# Supplementary material for: Investigating differential abundance methods in microbiome data: A benchmark study
Source: PLoS Comput Biol. 2022 Sep 8;18(9):e1010467. doi: 10.1371/journal.pcbi.1010467 (PMC9488820; doi:10.1371/journal.pcbi.1010467)
Supplement: S1 File — Section 1: Differences between benchmarking studies; Section 2: Differentially abundance methods; Section 3: Estimate of group A parameters; Section 4: Datasets; Section 5: Differentially abundant and differentially relative abundance scenarios; Section 6: Computational time; Section 7: Type of differentially abundant features simulated; Section 8: Results; Section 9: Comparison between ZIG and ZILG model in metagenomeSeq. (PDF) [file pcbi.1010467.s001.pdf]

Investigating differential abundance methods in microbiome data: a benchmark study

Marco Cappellato, Giacomo Baruzzo, Barbara Di Camillo

Supplementary File

1 Differences between benchmarking studies

Table A. Overview of the assessment metrics used in our and previous benchmark studies: False Positive Rate (FPR), False Discovery rate (FDR), Recall, Precision-Recall curve (PRcurve), partial Area Under PRcurve (pAUPR), Receiver Operating Characteristic (ROC) curve, area under ROC curve (AUC), partial AUC (pAUC), Runtime and Concordance.

	Metrics										
**Article**	**FPR**	**FDR**	**Recall**	**PRcurve**	**AUPR**	**pAUPR**	**ROC**	**AUC**	**pAUROC**	**Runtime**	**Concordance**
US	✓	✓	✓	✓	✓	✓				✓	
Lin et al.		✓	✓								
Calgaro et al.									✓	✓	
Hawinkel et al	✓	✓	✓				✓	✓			
Weiss et al.		✓	✓								
Khomich et al.	✓		✓								✓

Table B. Overview of the main covariates investigated in our and previous benchmark studies: Sample Size (SS), Percentage of DA taxa (PP), Sequencing depth (Seq. depth), relative abundance compensation (Compensation), Fold Change (FC), variability of taxa (Variability), all zero taxa treated as True Negative (All 0s as TN), threshold to avoid/allow low abundance DA taxa (Threshold), effect of sparsity parameter in zero-inflated model (Sparsity effect).

	Covariates								
**Article**	**SS**	**PP**	**Seq. depth**	**Compensation**	**FC**	**Variability**	**All 0s as TN**	**Threshold**	**Sparsity effect**
US	10, 25, 50, 100	5, 15, 20	8500, 17000, 34000, half/double, unbalance	yes	Vary in a parameteriz ed interval	✓	✓	✓	
Lin et al.		5, 15, 25							
Calgaro et al.	10, 20, 40	10, 50		yes/no	2,5				✓
Hawinkel et al	5, 25, 100			yes/no	1.5, 3, 5				
Weiss et al.	5, 25, 100		3000, 50000	yes/no	1.25, 2.5, 5, 10, 20				
Khomich et al.		2, 50			[1.6-2], [3-3.16]				

2 Differentially abundance methods

In this benchmark study we focus on established and recent differential abundance (DA) methods developed specifically for microbiome analyses and on two methods developed for differential expression analysis of RNA-seq data, but routinely used also in 16S sequencing data analysis.

ALDEx2 [1] models the data as the probability of observing the counts considering the observed absolute abundances as a realisation of a multinomial Poisson process. More in detail, it uses Monte-Carlo drawing of observed absolute abundance of each sample from a Dirichlet distribution with uniform priors. In this way, observed absolute abundances are converted into estimates of true relative abundances. Sampling is repeated *k* times and, for each realisation, the centered log ratio (clr) transformation is applied [2]. A statistical test (such as ANOVA, Welch's t-test or Wilcoxon rank sum test) is applied for each realisation of the dataset with correction for multiple testing, such as the Benjamini-Hochberg (BH) procedure. Finally, p-values and q-values are averaged across the *k* runs to assess statistical significance.

Similarly to ALDEx2, eBay [3] models observed absolute abundances using a Bayesian approach according to a multinomial distribution with Dirichlet prior for the taxa true relative abundances. Posterior Bayesian estimation for the true relative abundances is thus obtained as a weighted average of the vector of observed relative abundances and the mean of the prior distribution, thus eliminating the zeros from the dataset:

$$E(X'_{ij}|Y_j, \alpha)=\frac{L_j}{L_j+\alpha_+}Y'_{ij}+\frac{\alpha_+}{L_j+\alpha_+}\Psi_i \tag{1}$$

where L_j is the total reads number in sample j, Y'_{ij} is the observed relative abundance (i.e. observed absolute abundances divided by L_j), α_+ is the sum of α_i across the p features and Ψ_i is α_i divided by α_+. In practice, the hyper-parameters α_i are unknown; the authors propose to use either a uniform prior ($\alpha_i=\frac{1}{p}$ for each i) or, if a phylogenetic tree is available, a phylogeny-aware prior. After this step, the true relative abundances X'_{ij} are still in the Simplex space [2], by definition of proportions. The authors exploit the clr transformation to overcome the sum constraint and project data into Euclidean space, where Wilcoxon rank sum test and t-test can be applied to identify differentially abundant taxa.

Recently, Martin et al. [4] designed a novel framework, implemented in corncob R package, based on the beta-binomial model to perform differential abundance analysis specifically on microbiome data. The proposed beta-binomial regression framework allows taking into account the overdispersion observed in taxon's abundances, thus providing a new insight in differential variability testing. This new type of analysis is motivated by the hypothesis that a bacterial community perturbation could also induce an increase in variability. Basically, the corncob hierarchical model consists of two parts: a binomial distribution that models the observed absolute abundances given the sequencing depth and the true relative abundances; a beta distribution describing the latent true relative abundances parameters of the previously binomial process. The estimation of the model parameters is obtained through maximum-likelihood using the trust region optimisation algorithm [5,6]. Finally, the authors use several standard hypothesis testing procedures such as likelihood-ratio or Wald tests.

Recently, Mallick et al. [7] develop MaAsLin2, a differential abundance framework based on generalized linear and mixed models that allows to identify multivariable associations in meta-omics datasets. In brief, the user can test multiple covariates and repeated measures choosing different preprocessing steps such as filtering, normalisation or data transformation. In addition, metadata and microbial features are processed for missing values, unknown data values, and outliers. We adopt the default MaAsLin2 implementation, i.e. log-transformed linear model on Total Sum Scaling (TSS)-normalised data, since the authors shows that this setting it's appropriate for most analyses. However, it should be noted that the tool supports other statistical models including the Negative Binomial, Compound Poisson, Zero-inflated Negative Binomial, and also several normalisation/transformation approaches. Therefore, it could be used to assess differences in relative or absolute abundances depending on the adopted combination of input data, normalisation and transformation. When input data are count and TSS normalisation is used, MaAsLin2 is inherently modelling relative abundances, since TSS turns the data into proportions.

ALDEx2, eBay, corncob and MaAsLin2 are the only methods included in the comparison that define a DA feature between groups, when there is a difference in the mean true relative abundance. The remaining methods, on the other hand, focus on the difference in terms of mean true absolute abundance by modelling the observed counts.

One of the first approaches developed for metagenomic data is metagenomeSeq [8]. The underlying model assumed is the zero inflated Gaussian (ZIG) model, where a mixture of a point mass at zero and a Gaussian distribution are used to fit the data. The probability that an observed zero is due to the absence of the taxonomic feature in the microbial community rather than due to undersampling is estimated by modelling the mixture parameters as a binomial process. An expectation-maximization algorithm is then used to approximate maximum-likelihood estimates of the model parameters. Finally, the framework allows to estimate the fold change (FC) in mean true absolute abundances between groups and its standard error. The FC parameter and its standard error are employed to build a moderated t-statistic [9] that is used to test differentially abundant features between groups. The authors of metagenomeSeq modified their procedure reparametrizing the zero-inflation model in order to fit a zero-inflated model for each specific taxon separately. The authors recommend using zero-inflated Log-Gaussian (ZILG) mixture model for DA analysis.

In ANCOM, Mandal et al. [10] hypothesise that mean log true absolute abundance of two taxa in the population does not differ and that the mean log true absolute abundance of all taxa in the population is not directly proportional to the same constant between two groups or experimental conditions. In these hypotheses, the authors demonstrate that it is possible to verify the difference between true absolute abundances using a framework based on the log ratios between taxa. In particular, for each taxon i, $i = 1, 2, \ldots, p$, ANCOM calculates the log ratios between its observed absolute abundances and the counts of each other taxon in the same sample. A unit constant is added to all taxa to avoid computing logarithms for zero values. It then gives as input the obtained vectors to an ANOVA test comparing the groups of interest. Although ANCOM is testing a total of $p \frac{p-1}{2}$ distinct hypotheses, authors suggest correcting each feature independently considering *p-1* test. At the end, for each taxon a statistic is obtained, the so-called w-statistic, which counts the number of times the test has given a significant result across the (*p-1*) tests performed for the taxon. Finally, a taxon is considered DA if its w-statistic is above a percentile threshold (default 0.70) with respect to the empirical distribution of the w-statistics across all dataset. In this study we used a second version of the tool, ANCOM-II [11] that uses clr or alr transformation corrected by the mean within each observed group under the assumption that all specimens within an experimental group are a random sample from a common population of specimens. In addition, ANCOM-II transforms the datasets before applying the statistical tests. Namely: i) taxa with structural zeros, i.e. taxa having all zeros (or almost all zeros) in one group/condition but not in the

other, are considered DA and removed from the dataset; ii) outlier zeros are treated as missing values; iii) the other zeros are imputed as 1. To identify the outlier zeros, ANCOM-II fits a mixture of two gaussian distributions to the normalised data and declares the two distributions as distant if the 97.5th percentile of the first distribution does not overlap with the 2.5th percentile of the second distribution, and one distribution is "*h* % heavier" than other in terms of number of samples, for some pre-specified *h*. If distributions are well separated, zeros falling in the left one are considered outliers.

Recently, ANCOM-BC [12] further overcomes the bias introduced by differences in the sampling fractions across samples within the same group. The sampling fraction c_j is defined as the ratio between the expected value of the observed absolute abundance for the *i*-th taxon in the *j*-th subject and its true absolute abundance. This ratio c_j is assumed constant for each taxon in the same sample *j*. The framework proposed by the authors allows estimating the unobservable c_j, correcting the bias introduced by the possible extreme variation among samples. To do that, ANCOM-BC uses a linear model with a sample-specific offset term estimated from the observed absolute abundances to describe the log abundance of feature *i* in subject *j*. Basically, the offset is used to correct the bias induced by the sampling fraction, while the use of log ratios then takes into account the compositional nature of the data. As in ANCOM-II, the authors transform the dataset to take into account different types of zeros.

As previously mentioned, we decide to include in the comparison also two tools widely used in RNA-seq field, i.e. edgeR [13] and DESeq2 [14], which assume a negative binomial distribution of the data. Observed absolute abundances are normalised with different methods, such as Trimmed Mean of the M-values (TMM) in edgeR and relative log expression (RLE) in DESeq2, to account for library size variations. The estimation of the dispersion parameter is also conducted differently. In edgeR, taxon-wise dispersions are achieved through conditional maximum-likelihood, exploiting an empirical Bayesian approach [15] to shrink similar estimates towards a common value. On the other hand, DESeq2 fits a single dispersion trend from maximum likelihood taxon-wise estimates and shrinks dispersions towards the values predicted from the dispersion curve using an empirical Bayesian procedure. Finally, different statistical tests can be used for DA taxa detection, such as likelihood-ratio test in edgeR or Wald test in DESeq2.

In Table 1 in the main text, we classify each method as addressing relative or absolute abundances based on the underlying definition of DA taxa adopted and the preprocessing adopted. DESeq2, edgeR and metagenomeSeq apply normalisation in order to overcome bias introduced by different microbial loads and sequencing depths across samples. Although these methods use different statistical models, in principle normalisation should correct the above biased in order to be able to estimate Fold Changes between conditions without compositional bias. Therefore, we indicated them as addressing absolute differential abundance.

The authors of ANCOM and ANCOM-BC demonstrate that their models allow to explicitly test the differences in terms of true absolute abundance [10,12].

On the other hand, although corncob models directly observed absolute abundance, its approach aim at modelling the expected true relative abundance of each taxon and how it is linked to covariates in the data. For this reason, we classified this method as addressing relative differential abundance.

Similarly to ALDEx2, eBay models observed absolute abundances using a Bayesian approach. The authors formulate a Posterior Bayesian estimation for the true relative abundances. In both methods the estimated values are transformed with clr transformation in order to project relative data from Simplex to Euclidean space. The transformation is necessary to then carry out the statistical test correctly. According to the definition of clr, the differences between two or more groups are evaluated in relation to the geometric mean abundance. Consequently, these were classified as addressing relative differential abundance with respect to geometric mean.

As previously said, since we set TSS as a normalisation approach in MaAsLin2, we characterise this method as addressing relative differential abundance.

Another important aspect arises from Table 1 in the main text. Only few methods implement some strategies to consider taxon-specific biases, but none of these strategies are included in the default setting (so not included in our comparison). Specifically, in edgeR and DESeq2 packages the user can enter feature-specific correction factors in the model to account for feature-specific technical biases (e.g. GC content and gene length) [16,17].

Since the methods differ according to the definition of feature differentially abundant (i.e. relative or absolute), two types of baselines with different normalisation approaches are exploited: a naïve approach such as average

library size (AVG) and clr transformation. Basically, AVG scales the observed absolute abundances of each subject/sample to the average library size observed in the dataset. Instead, clr is a compositional transformation that divides each entry of the sample/subject observed absolute abundances by the geometric mean. We use the "clr" function of the R compositions [18] package, which calculates the geometric mean on the non-null counts. The transformation treats the data as relative information with respect to geometric mean and aims to remove the sum constraint which is independent of the scale on which the data is found. Consequently, applying Wilcox test after AVG can be considered the baseline for DA methods that consider true absolute abundances changes, while clr for methods that focus on differences in terms of true relative abundances with respect to geometric mean. After normalising the data, the test was applied taxa-wise between the two experimental groups in our benchmark framework, using the R core function "wilcox.test". Finally, the resulting p-values were corrected through the BH procedure using the R core function "p.adjust".

3 Estimate of group A parameters

We exploit metaSPARSim [19] for the estimation of the vector μ in condition A. In particular, the "estimate_intensity" function is used. The intensity μ^A is computed as mean of normalised counts for each feature. We normalise data with average library size (AVG) normalisation, i.e. scaling the abundances with respect to the average sequencing depth.

During the estimation of the variability parameter ϕ metaSPARSim assumes that the variance of the i-th feature count can be described from the following equation

$$var(Y_i)=\lambda_i^A(1+\lambda_i^A \cdot \phi_i^A) \quad (2)$$

where λ_i^A represents the mean expected count value for taxa i in the observed group A, whereas ϕ_i^A is the dispersion parameter. This model is often used to describe sequencing count data variability [13,14] and, despite being a raw estimate of OTU variability, it allowed metaSPARSim simulated count table to resemble the characteristics observed in real datasets. Here we use a raw estimate of the variability parameter φ_i^A using the inverse formula:

$$\varphi_i^A \sim \phi_i^A = \frac{\sigma^2(Y_i) - \underline{\mu_i^A}}{\underline{\mu_i^A}^2} \tag{3}$$

where $\underline{\mu_i^A}$ is the observed sample mean of normalised counts in group A and $\sigma^2(Y_i)$ is the group A variance calculated using an estimator robust to the outliers, as the square of the mean absolute deviation (MAD):

$$\frac{1}{SS^A}\sum_{j=1}^{SS^A}(Y_{ij}\text{-}median(Y_i))^2 \tag{4}$$

In Equation (4), SS^A is the sample size of the condition A and Y_i is the counts vector of the i-th feature across all SS^A measurements. In order to avoid possible negative dispersion values, i.e. when $\sigma^2(Y_i) < \underline{\mu_i^A}$, we exploit the linear interpolation of the values in the (μ^A, φ^A) plane. Linear interpolation allows to assign variability values as close as possible to those already observed in the data that have similar average abundances. In this way, the mean-dispersion relationship mimics the trend of the other features observed in real data.

As regards the last parameter to be provided as input to the simulator, i.e. the sequencing depth vector L, we exploit the "estimate_library_size" function provided in metaSPARSim R package to obtain SS^A values of sequencing depth. In case the number of samples to simulate is higher than SS^A, first the L vector is divided into N bins of length 1000. Then, one of the N bins is selected based on the frequency of each bin. Finally, the new sequencing depth value is sampled uniformly within the bounds of the selected bin. This procedure is repeated for all new sequencing depth values up to the number of samples required. The choice of bin length is based on the observed frequency of sequencing depth values in the three datasets.

4 Datasets

The first dataset was obtained in the context of the Human Microbiome Project (HMP) [20,21] and, among the microbial communities that characterise the oral cavity, we extracted the one related to the teeth site. The second dataset comes from a study conducted by He et al. [22] on the effect of the type of feeding in infants. The count table and metadata are available in the QIITA database (https://qiita.ucsd.edu/study/description/12021#). We focussed on the breast-fed (BF) reference group by

choosing the "6 month" time point. The third dataset comes from The Inflammatory Bowel Disease Multi'omics Database (IBDMD) [23] a study conducted as part of the Integrative Human Microbiome Project (HMP2 or iHMP) to characterise gut microbial ecosystem in the context of Inflammatory Bowel Disease (IBD). Data are publicly available from the project reference site (https://ibdmdb.org/). We extracted abundance profiles related to the control condition of healthy subjects (nonIBD).

BF and nonIBD subjects' profile with sequencing depth between 5000 and 25000 are retained. In addition, taxa with zero values across all the samples considered in our analysis were filtered out.

Since tooth, BF and nonIBD are human associated niches, we add in our benchmarking other two datasets: namely "AnimalGut" and "Soil" (see Table C). The first dataset was obtained from the preset available in metaSPARSim R package [19]. Instead, the second dataset comes from a study conducted by Yurgel et al. [24] on the differences between bacterial and eukaryotic soil communities associated with natural and managed habitats of wild blueberry. We download already preprocessed data from Nearing et al. [25]. Again, these two datasets are subjected to the same filtering on the sequencing depth and zero features described above.

Table C. Overview of the datasets used to estimate the parameters of condition A.

**Original Dataset**	**Sequencing Platform**	**16S rDNA region**	**Type of samples**	**Condition**	**Number of samples/subjects**	**Mean sequencing depth**
metaSPARSim preset	Illumina HiSeq2500	V3-4	chicken gut microbiota	X4.5_Pos	5	~170000
Yurgel et al.	Illumina MiSeq	V6-8	blueberry habitats	Soil	39	~11000

5 Differentially abundant and differentially relative abundance scenarios

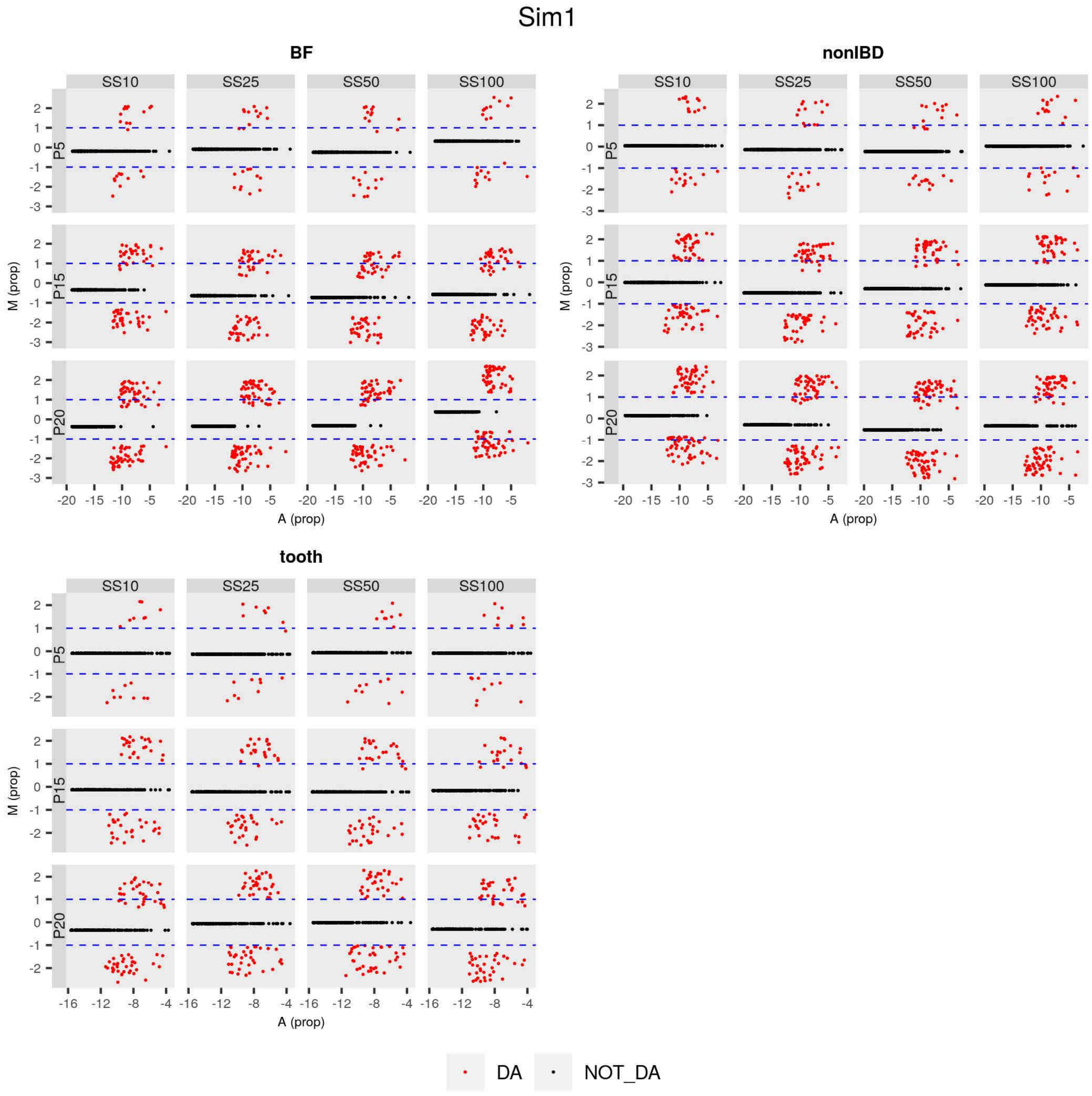

Figure 1. Simulation 1. MVA plot on true relative abundances (proportions) for each dataset considered in the comparison in the main scenario with DA features simulated. In each box corresponding to the dataset, different percentages (P) of the simulated DA features are on rows, while different sample size (SS) values are on columns. Features simulated as DA are in red, while as NOT DA in black. Dotted blue lines represent Fold Change (FC) values of 2 and 1/2 (1 and -1 in log-scale, respectively).

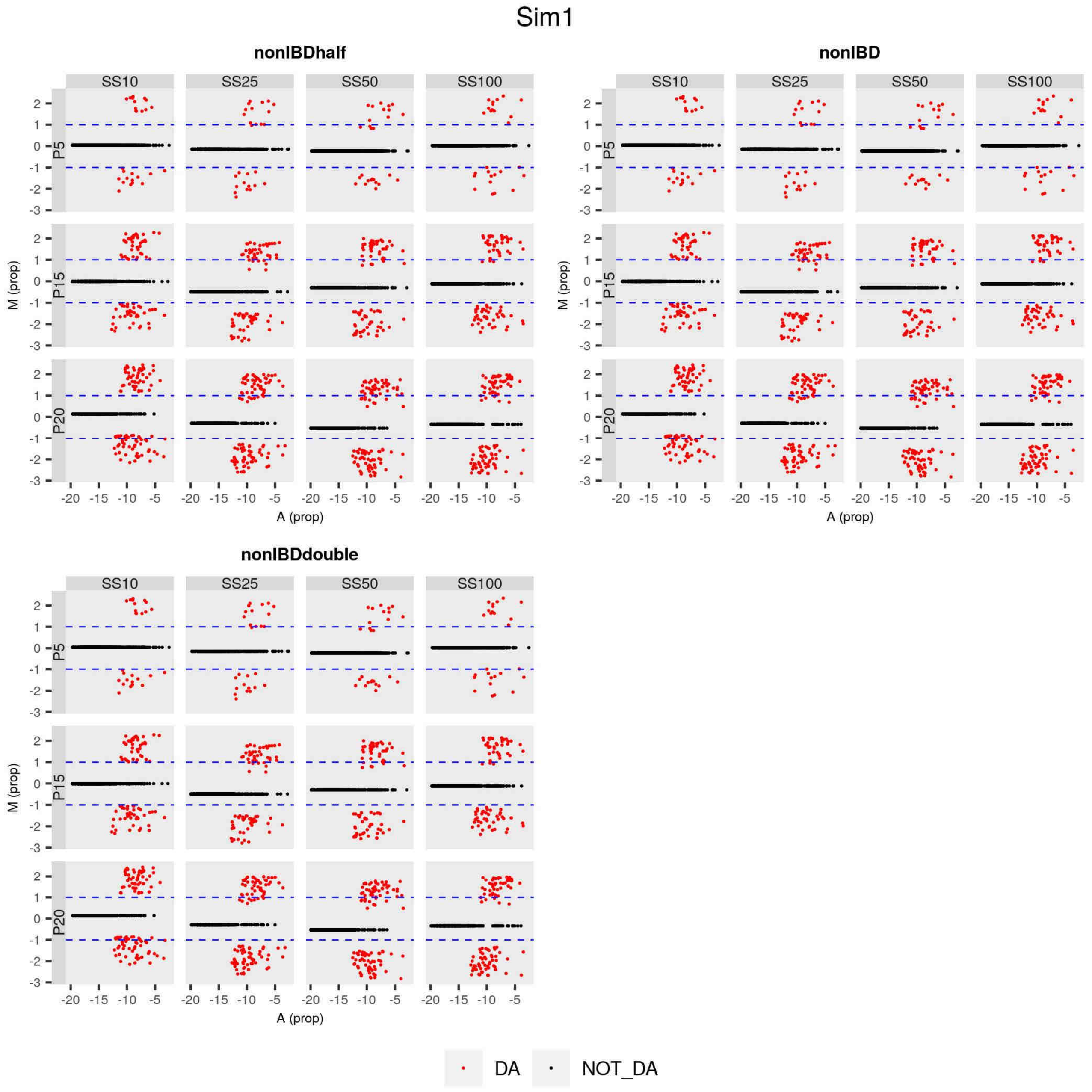

Figure 2. Simulation 1. MVA plot on relative abundances (proportions) for nonIBD dataset in the scenario with half, original and double sequencing depth. In each box corresponding to the dataset, different percentages (P) of the simulated DA features are on rows, while different sample size (SS) values are on columns. Features simulated as DA are in red, while as NOT DA in black. Dotted blue lines represent Fold Change (FC) values of 2 and 1/2 (1 and -1 in log-scale, respectively).

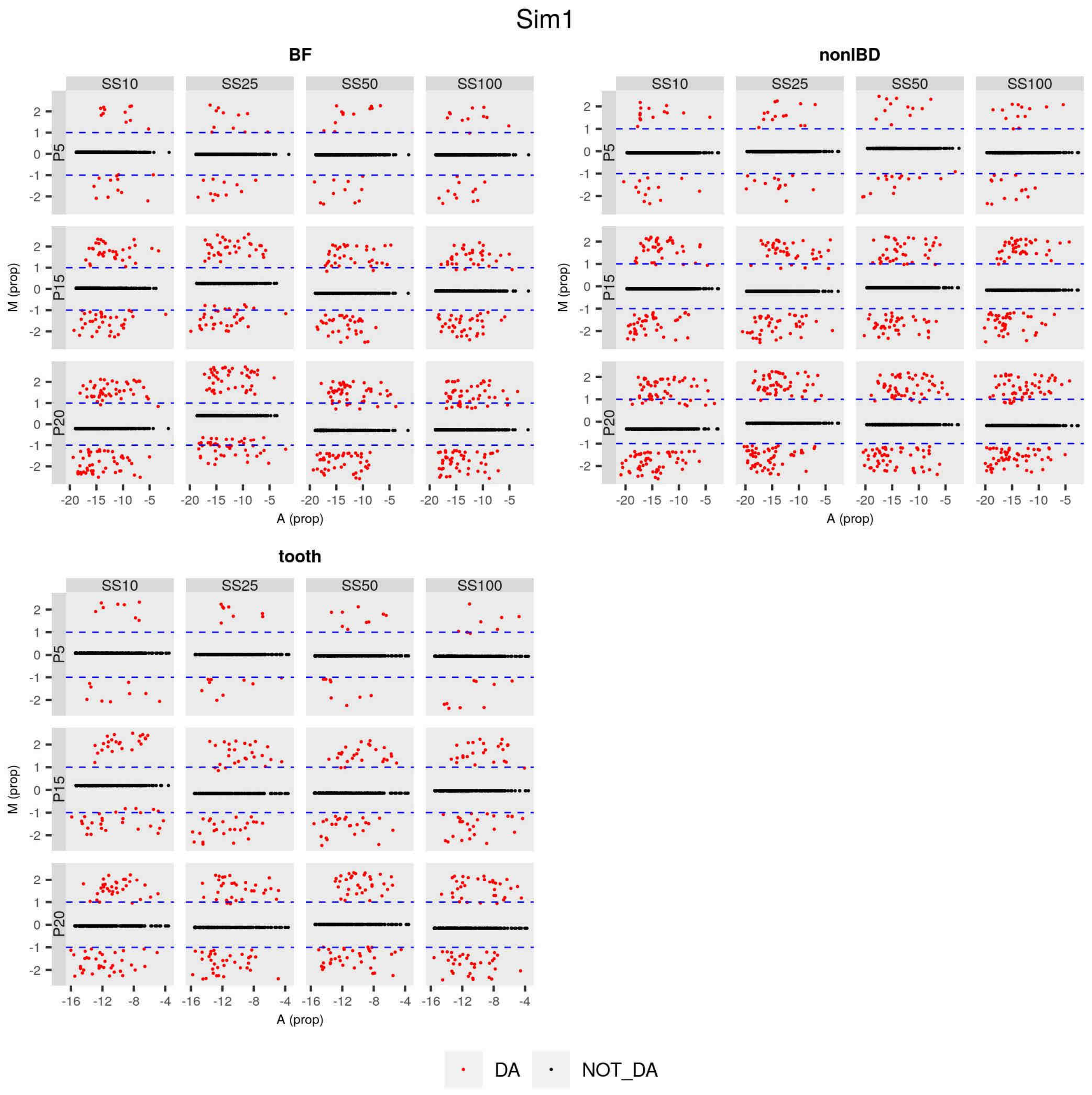

Figure 3. Simulation 1. MVA plot on relative abundances (proportions) for each dataset considered in the comparison in the scenario with DA features simulated and θ=*0*. In each box corresponding to the dataset different percentages (P) of the simulated DA features are on rows, while different sample size (SS) values are on columns. Features simulated as DA are in red, while as NOT DA in black. Dotted blue lines represent Fold Change (FC) values of 2 and 1/2 (1 and -1 in log-scale, respectively).

6 Computational time

The results of the comparison about the computational times are shown in Figures 4-5.

Corncob computational time is highly dependent on the increase in the sample size, followed by ANCOM, eBay which also shows a greater variance throughout different runs and ALDEx2. All other methods, on the other hand, are robust to the variation of SS by maintaining a constant low average time in the different configurations.

Although a ranking is visible between the methods, in this simulation setting and considering the sizes of the input features, computational times do not represent a particular obstacle, since the longer average execution time is around 4 minutes. In addition, it should be noted that ALDEx2 and DESeq2 allow the user to set the number of cores for parallel execution, thus further decreasing the computational complexity.

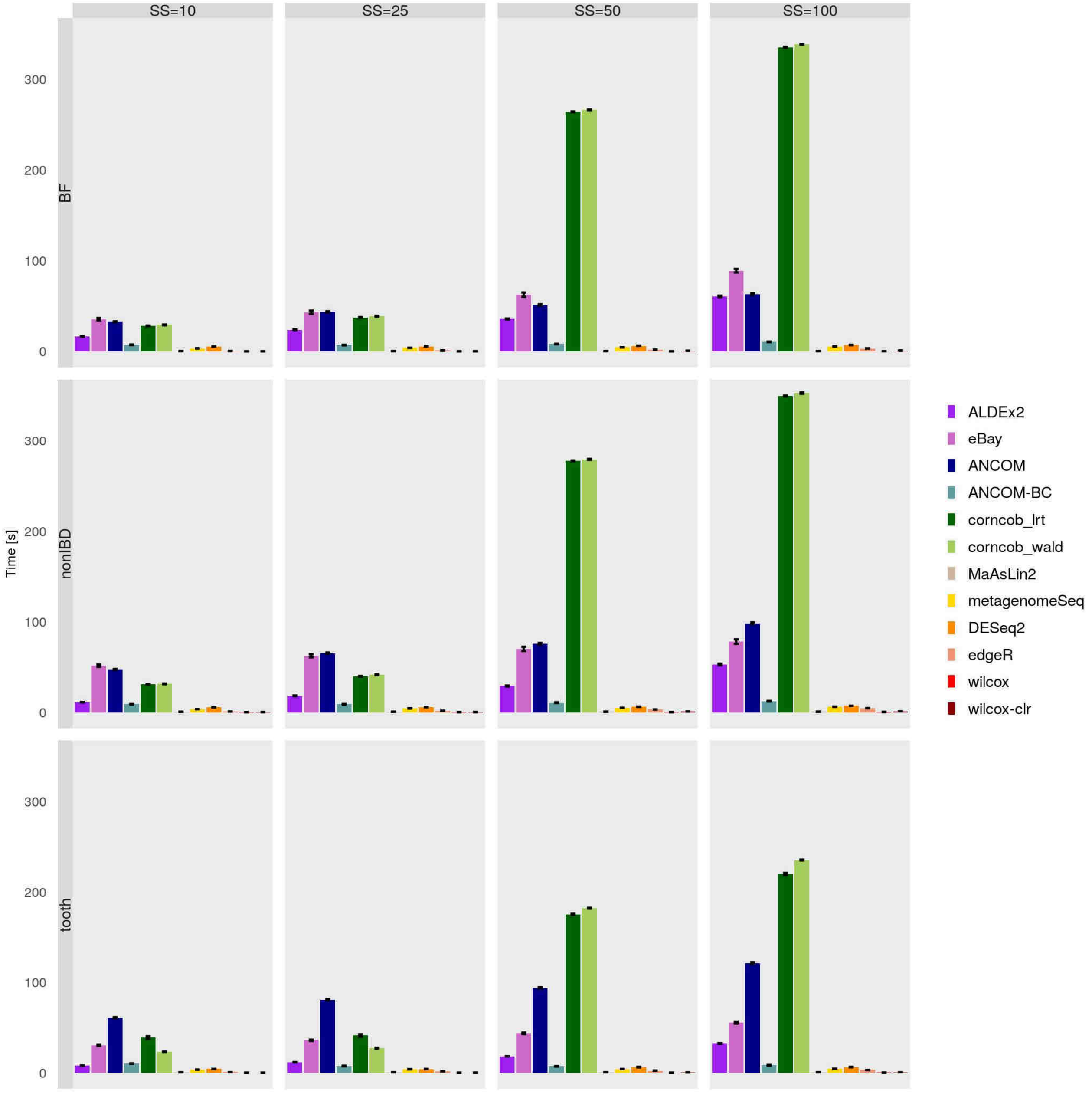

Figure 4. Running time in the main scenario with simulated DA features. In each set of boxes, different datasets are on rows, while different sample size (SS) values are on columns. The runtime values are averaged over the 50 simulations and the bars show the standard error.

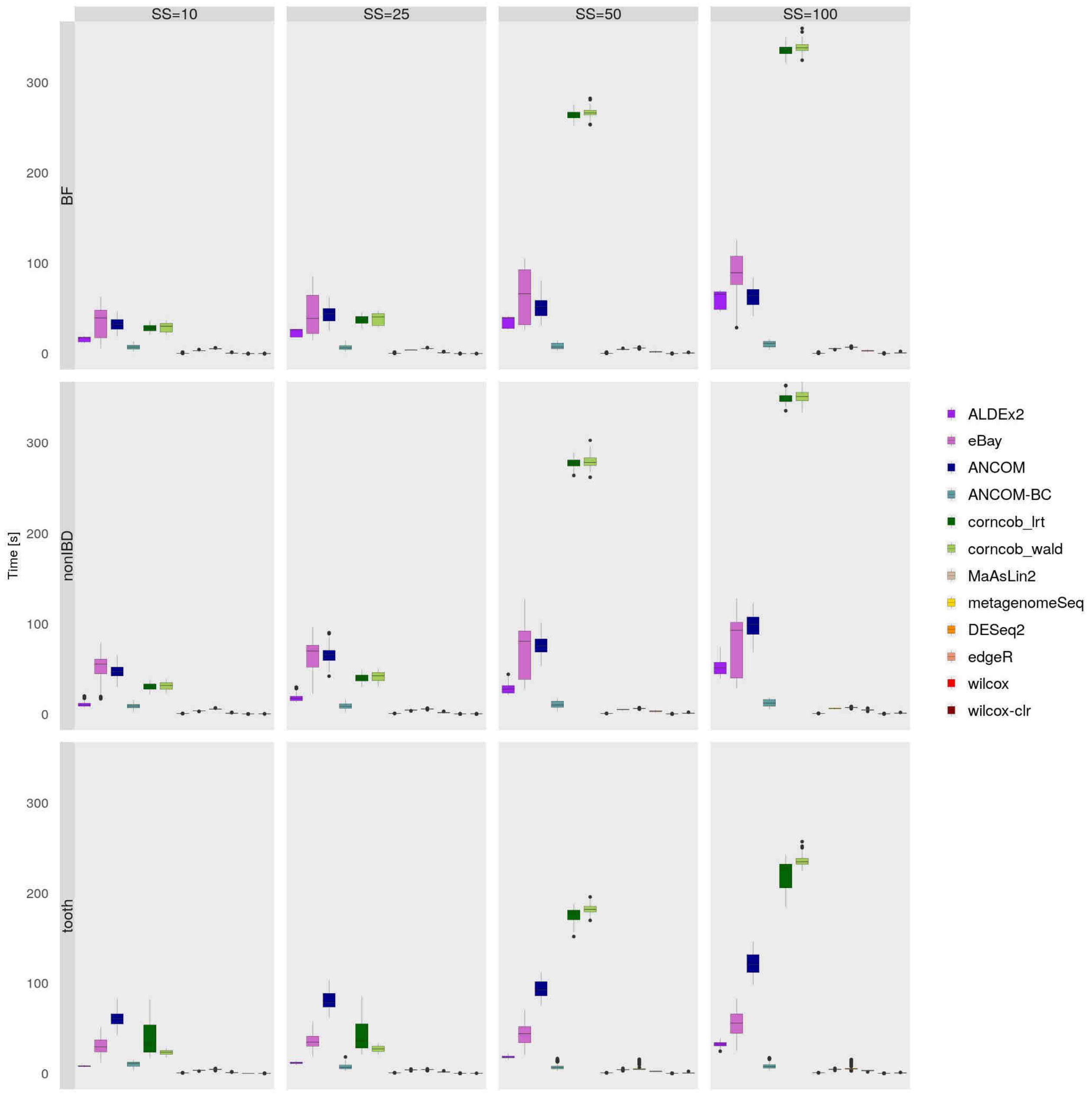

Figure 5: Boxplot of running time distribution across the 50 simulations of the main scenario with simulated DA features. In each set of boxes, different datasets are on rows, while different sample size (SS) values are on columns.

7 Type of differentially abundant features simulated

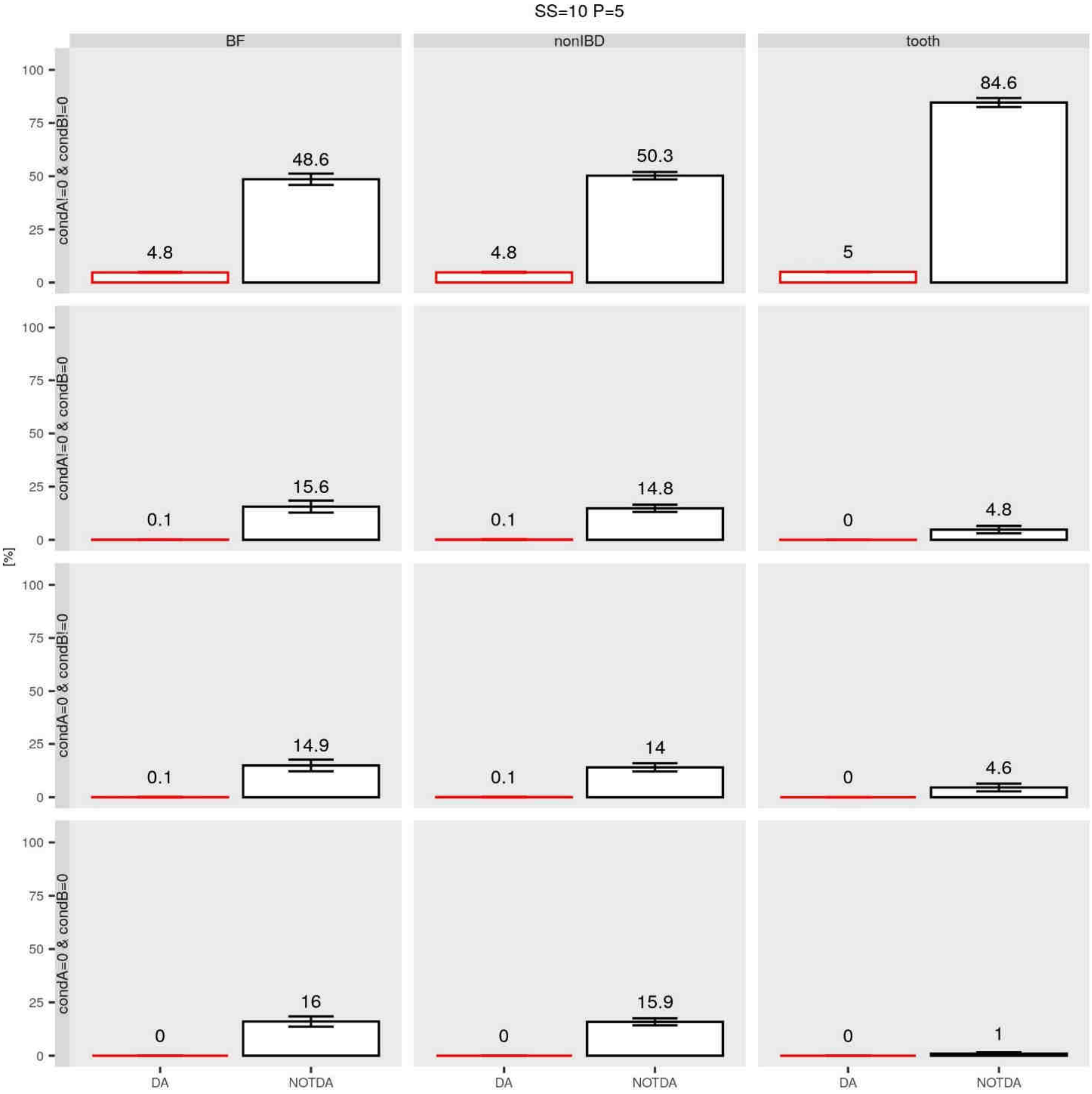

Figure 6: Percentages of features simulated as DA and NOT DA in the main scenario with simulated DA features in relation to the following cases: both conditions not null (condA!=0 & condB!=0), only condition B all null (condA!=0 & condB=0), only condition A all null (condA=0 & condB!=0), both conditions all null (condA=0 & condB=0). The percentages shown are averaged between the 50 simulations obtained by setting the sample size (SS) to 10 and with a percentage of inserted DA features (P) of 5%.

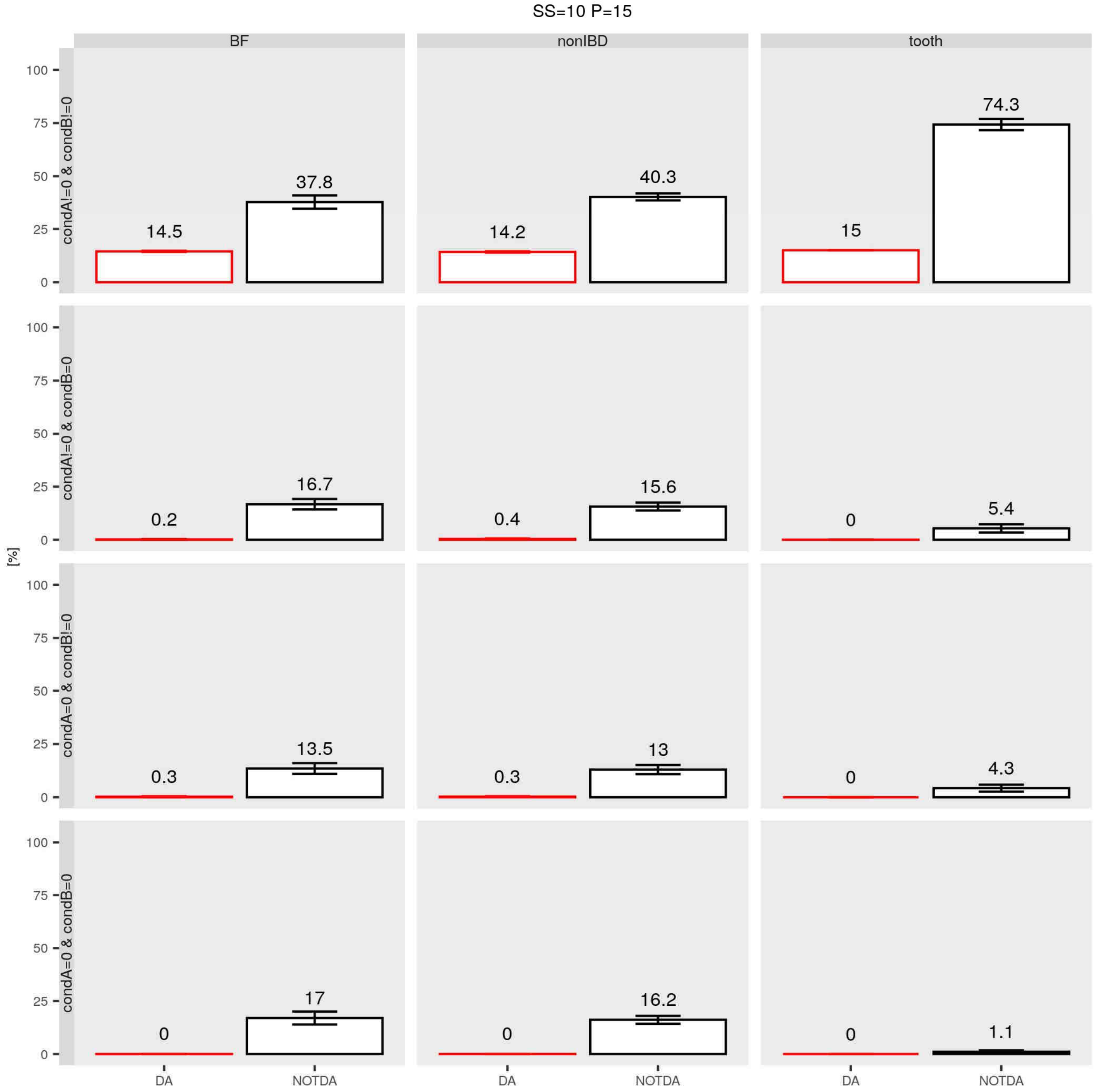

Figure 7: Percentages of features simulated as DA and NOT DA in the main scenario with simulated DA features in relation to the following cases: both conditions not null (condA!=0 & condB!=0), only condition B all null (condA!=0 & condB=0), only condition A all null (condA=0 & condB!=0), both conditions all null (condA=0 & condB=0). The percentages shown are averaged between the 50 simulations obtained by setting the sample size (SS) to 10 and with a percentage of inserted DA features (P) of 15%.

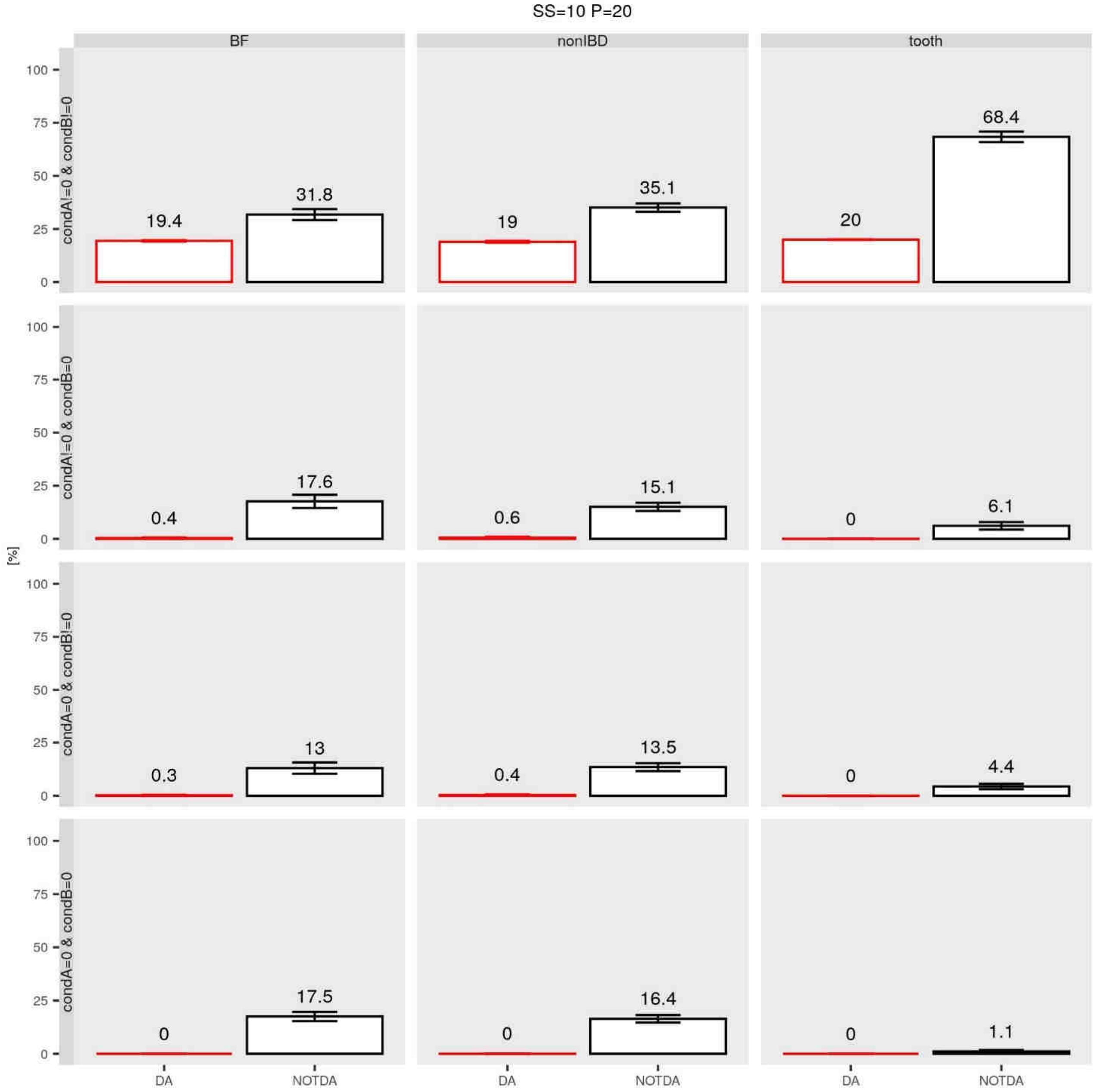

Figure 8: Percentages of features simulated as DA and NOT DA in the main scenario with simulated DA features in relation to the following cases: both conditions not null (condA!=0 & condB!=0), only condition B all null (condA!=0 & condB=0), only condition A all null (condA=0 & condB!=0), both conditions all null (condA=0 & condB=0). The percentages shown are averaged between the 50 simulations obtained by setting the sample size (SS) to 10 and with a percentage of inserted DA features (P) of 20%.

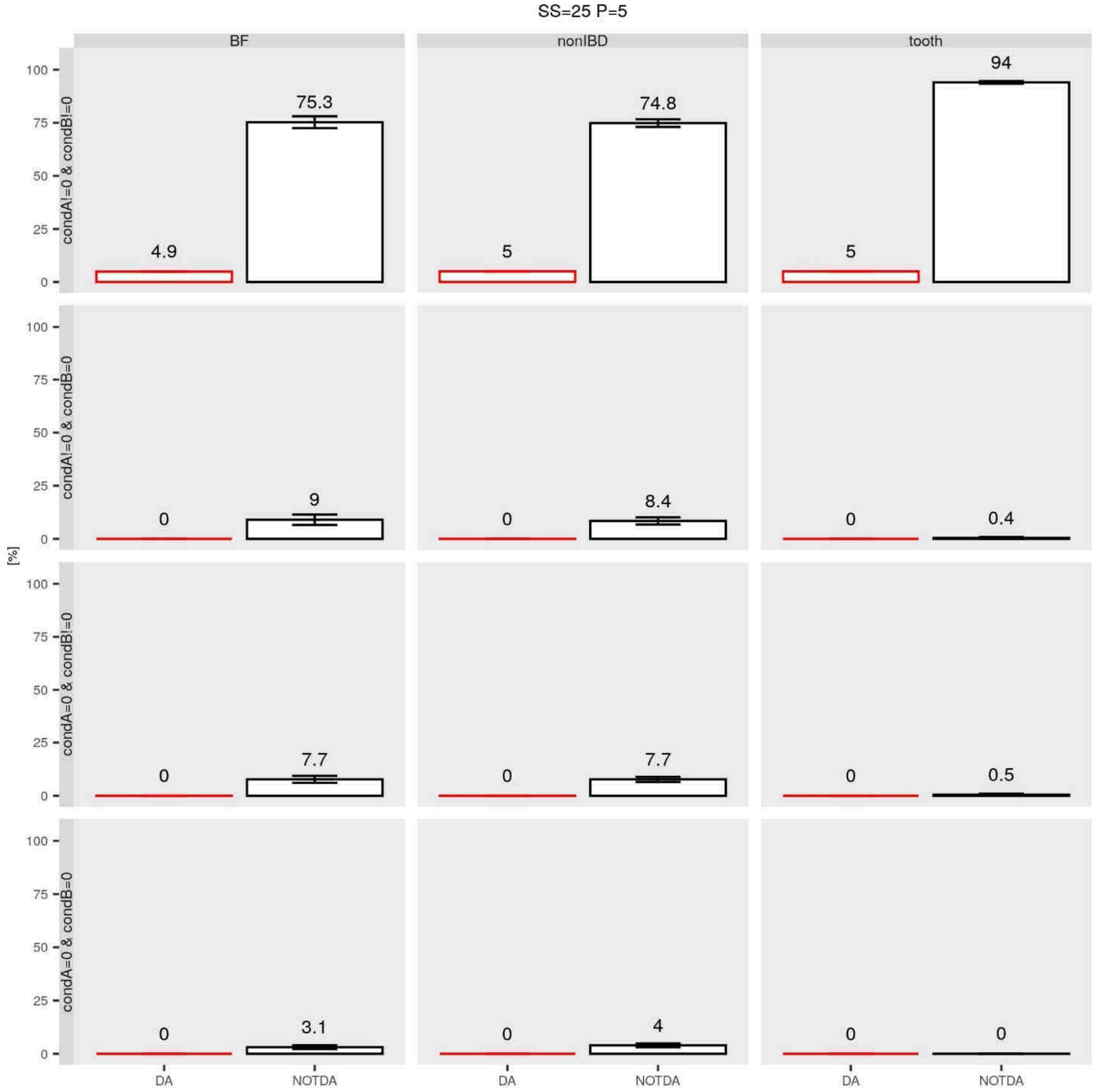

Figure 9: Percentages of features simulated as DA and NOT DA in the main scenario with simulated DA features in relation to the following cases: both conditions not null (condA!=0 & condB!=0), only condition B all null (condA!=0 & condB=0), only condition A all null (condA=0 & condB!=0), both conditions all null (condA=0 & condB=0). The percentages shown are averaged between the 50 simulations obtained by setting the sample size (SS) to 25 and with a percentage of inserted DA features (P) of 5%.

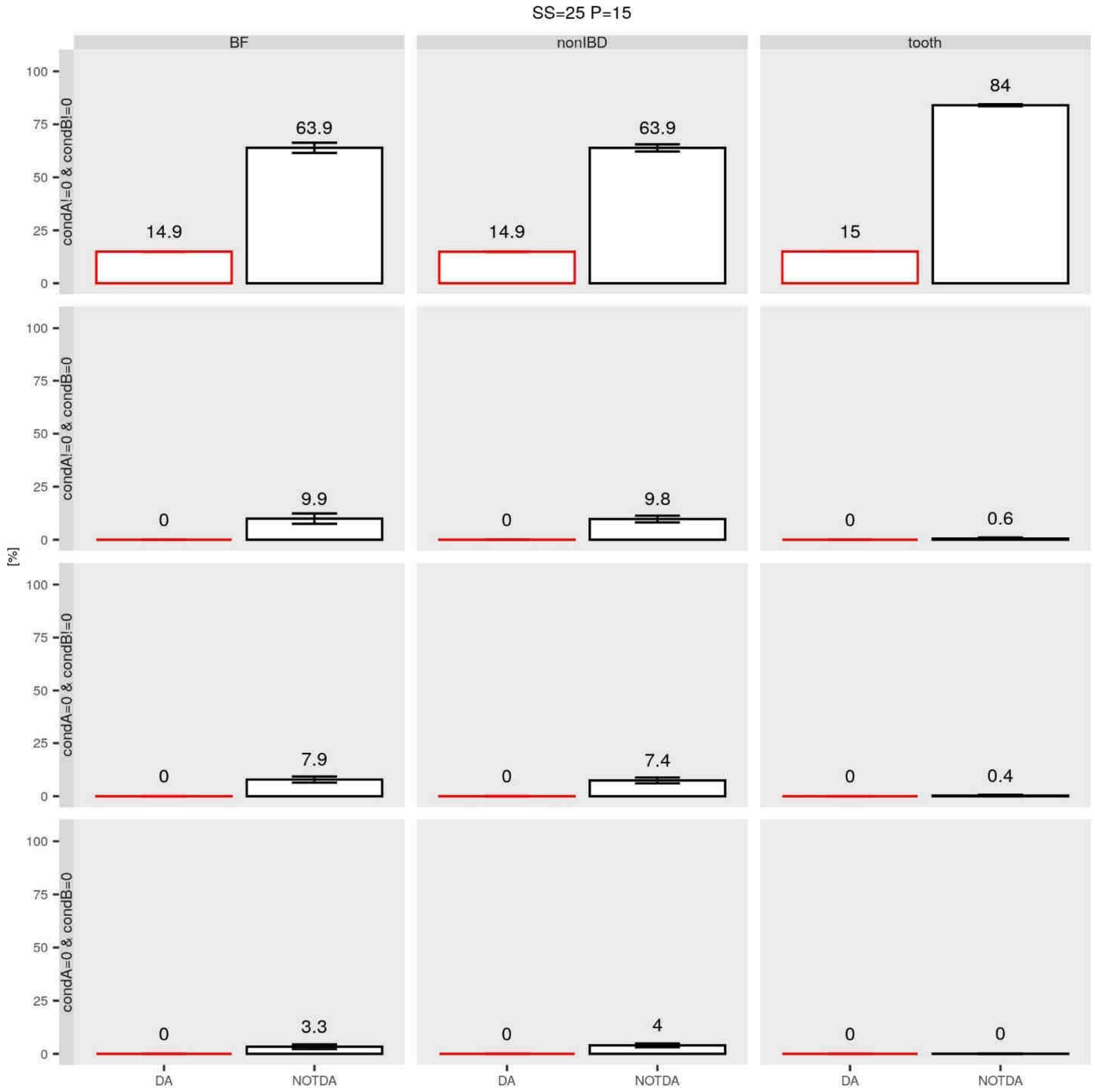

Figure 10: Percentages of features simulated as DA and NOT DA in the main scenario with simulated DA features in relation to the following cases: both conditions not null (condA!=0 & condB!=0), only condition B all null (condA!=0 & condB=0), only condition A all null (condA=0 & condB!=0), both conditions all null (condA=0 & condB=0). The percentages shown are averaged between the 50 simulations obtained by setting the sample size (SS) to 25 and with a percentage of inserted DA features (P) of 15%.

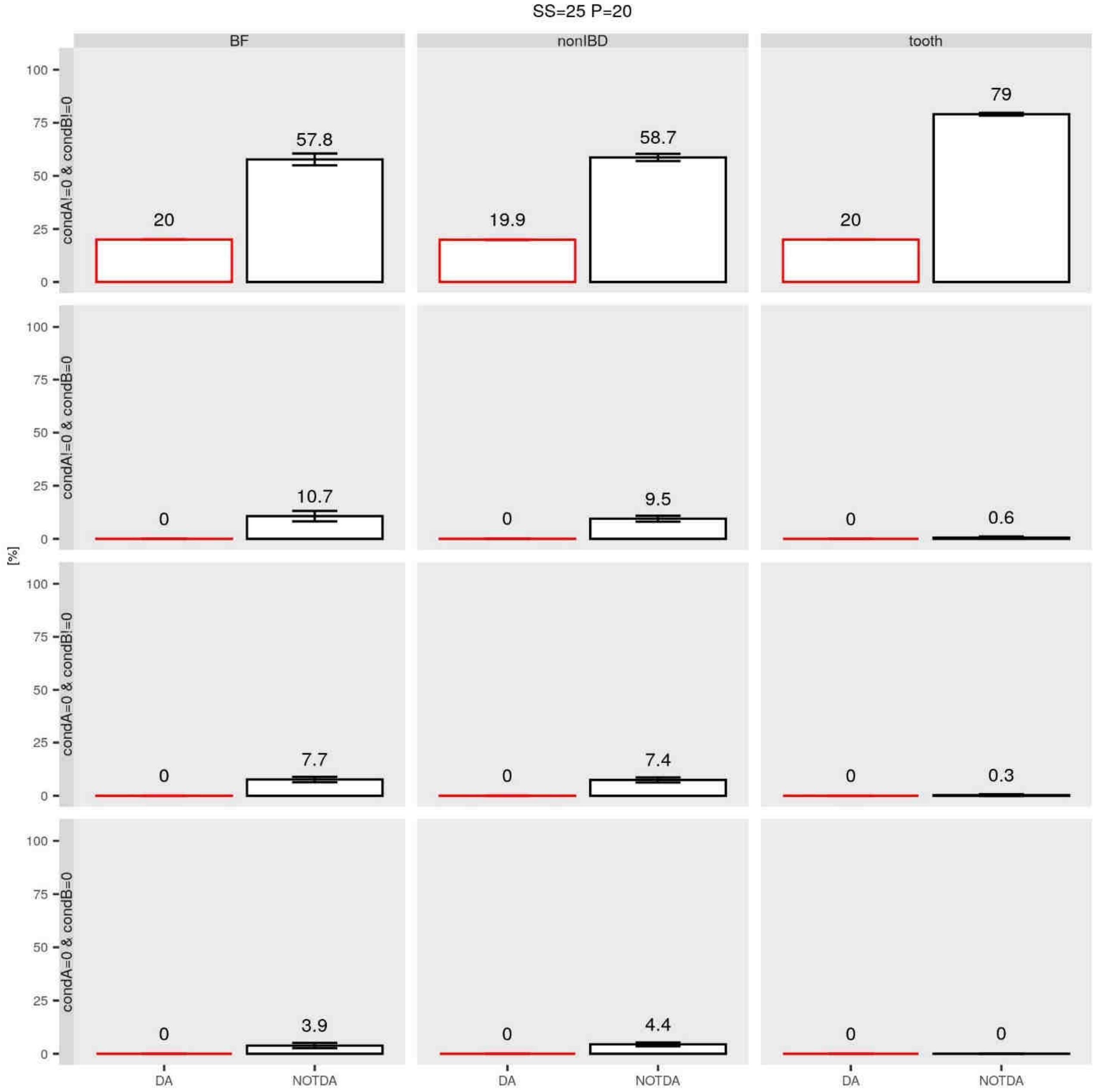

Figure 11: Percentages of features simulated as DA and NOT DA in the main scenario with simulated DA features in relation to the following cases: both conditions not null (condA!=0 & condB!=0), only condition B all null (condA!=0 & condB=0), only condition A all null (condA=0 & condB!=0), both conditions all null (condA=0 & condB=0). The percentages shown are averaged between the 50 simulations obtained by setting the sample size (SS) to 25 and with a percentage of inserted DA features (P) of 20%.

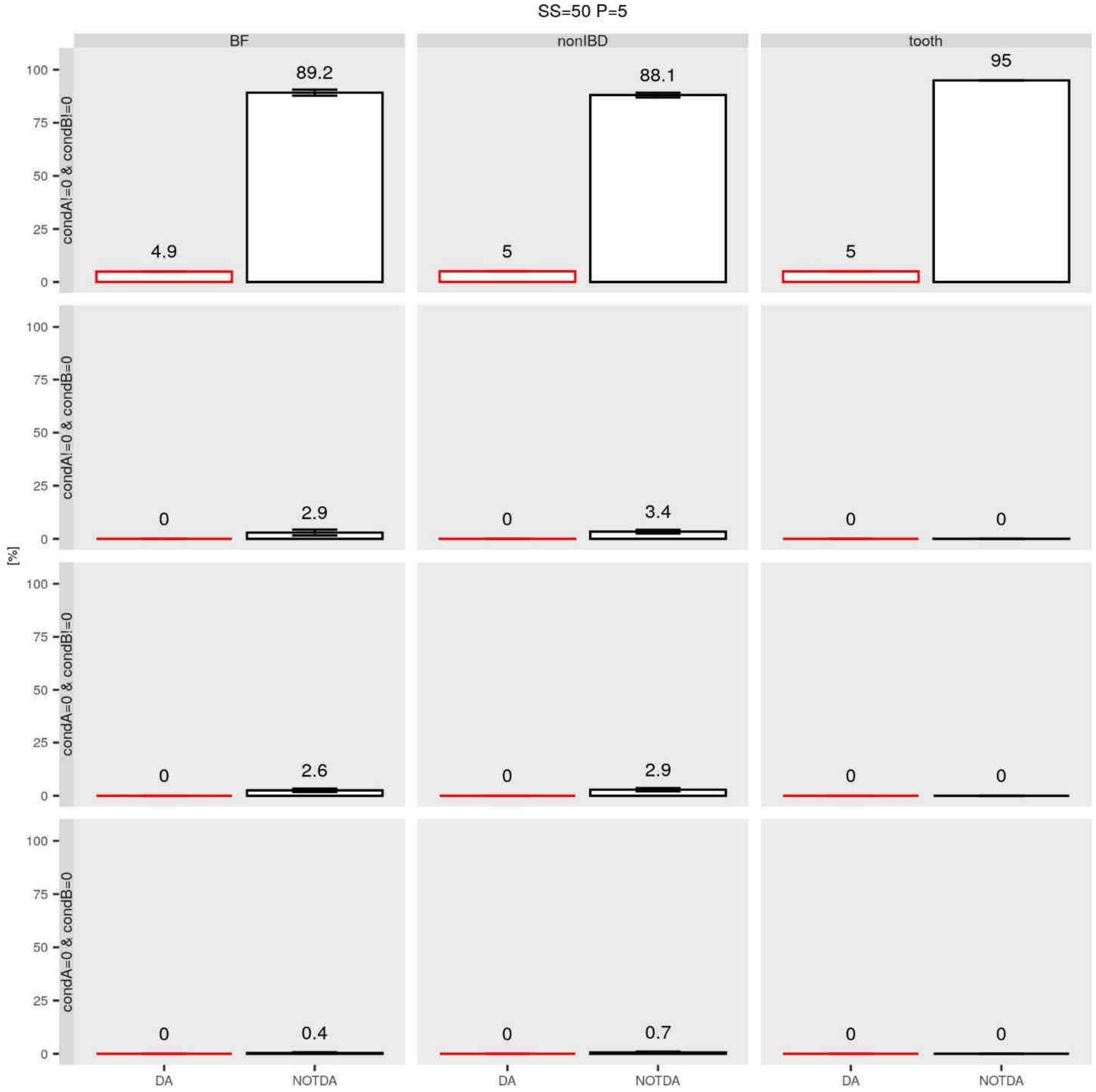

Figure 12: Percentages of features simulated as DA and NOT DA in the main scenario with simulated DA features in relation to the following cases: both conditions not null (condA!=0 & condB!=0), only condition B all null (condA!=0 & condB=0), only condition A all null (condA=0 & condB!=0), both conditions all null (condA=0 & condB=0). The percentages shown are averaged between the 50 simulations obtained by setting the sample size (SS) to 50 and with a percentage of inserted DA features (P) of 5%.

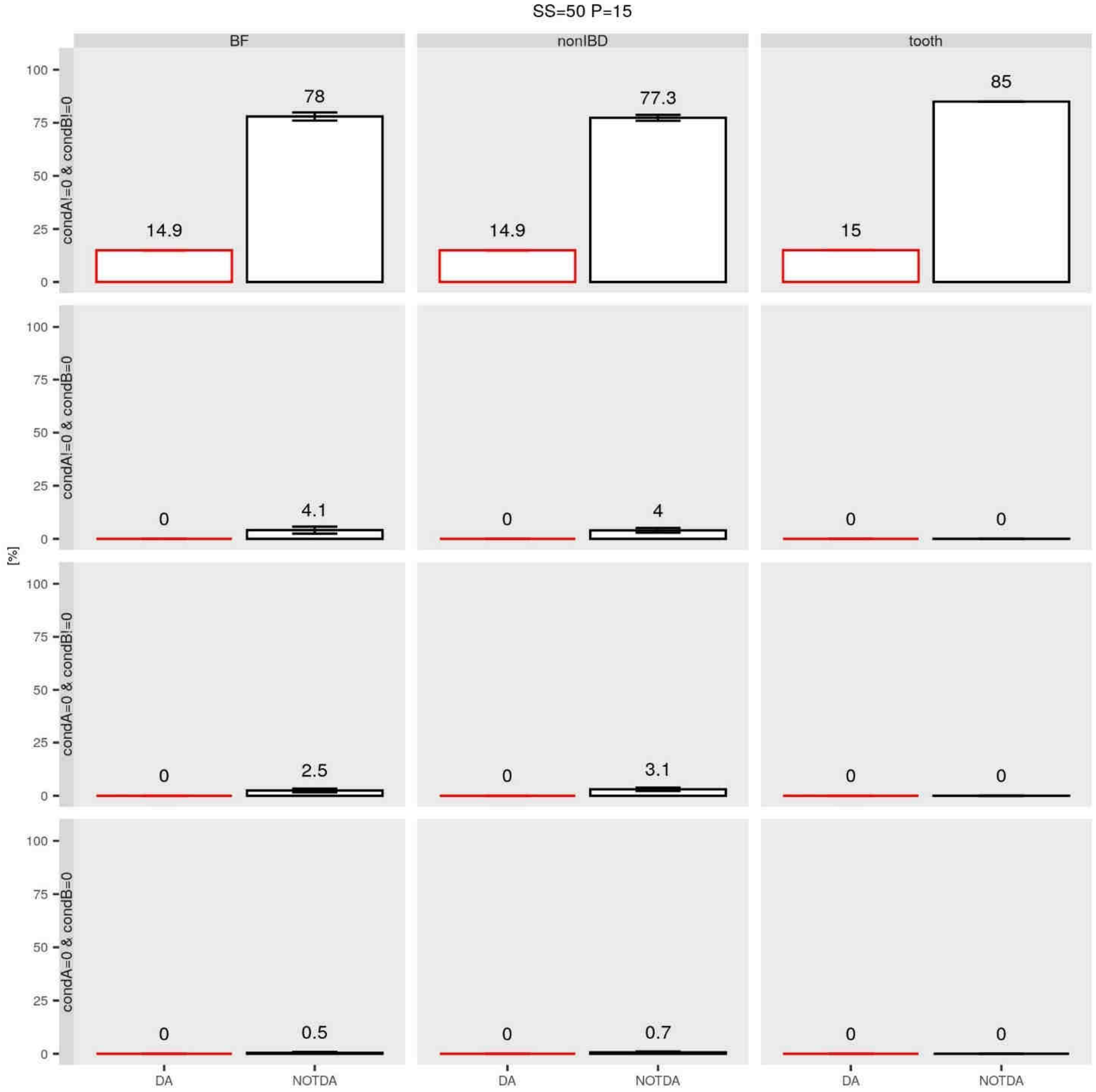

Figure 13: Percentages of features simulated as DA and NOT DA in the main scenario with simulated DA features in relation to the following cases: both conditions not null (condA!=0 & condB!=0), only condition B all null (condA!=0 & condB=0), only condition A all null (condA=0 & condB!=0), both conditions all null (condA=0 & condB=0). The percentages shown are averaged between the 50 simulations obtained by setting the sample size (SS) to 50 and with a percentage of inserted DA features (P) of 15%.

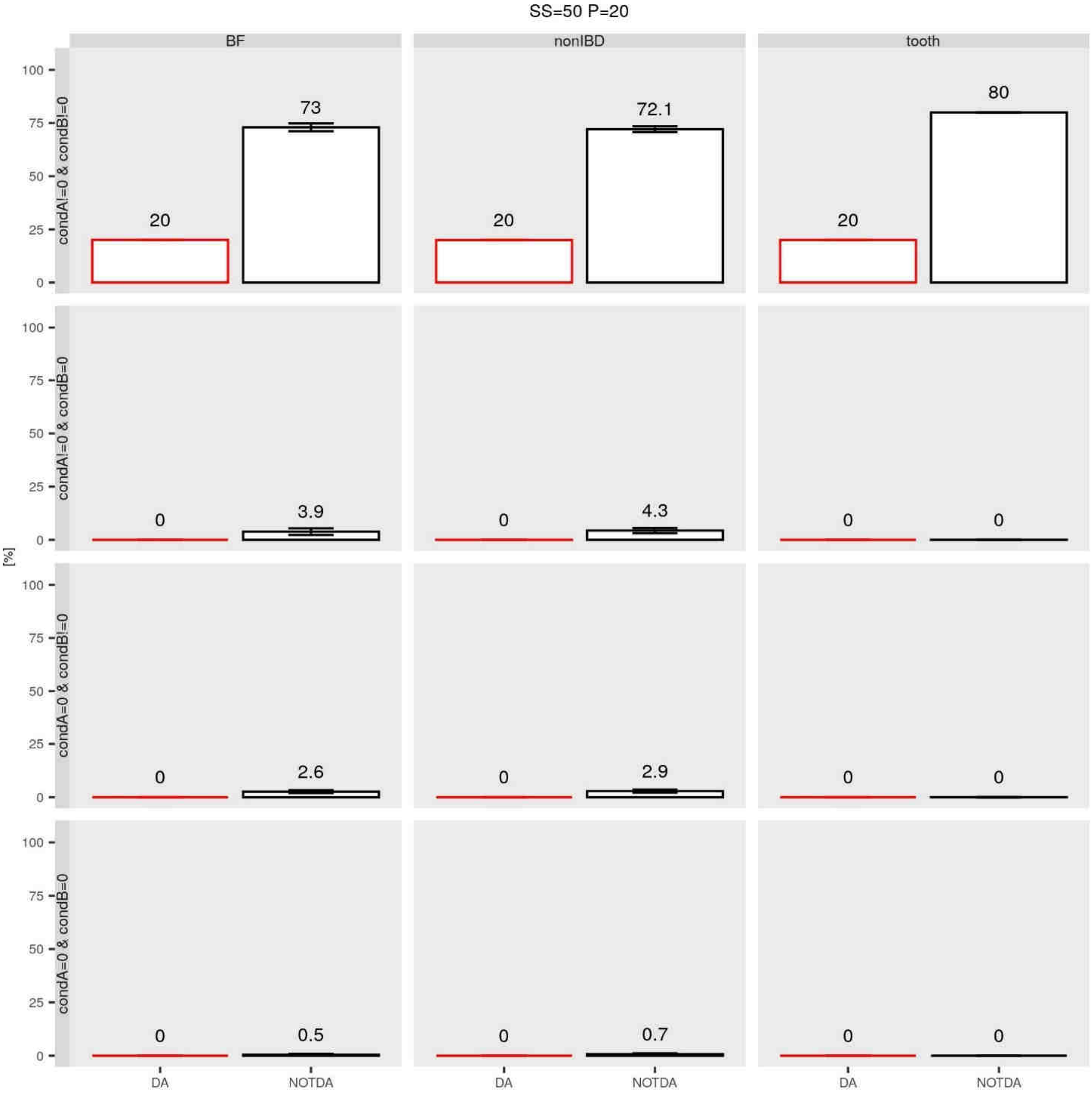

Figure 14: Percentages of features simulated as DA and NOT DA in the main scenario with simulated DA features in relation to the following cases: both conditions not null (condA!=0 & condB!=0), only condition B all null (condA!=0 & condB=0), only condition A all null (condA=0 & condB!=0), both conditions all null (condA=0 & condB=0). The percentages shown are averaged between the 50 simulations obtained by setting the sample size (SS) to 50 and with a percentage of inserted DA features (P) of 20%.

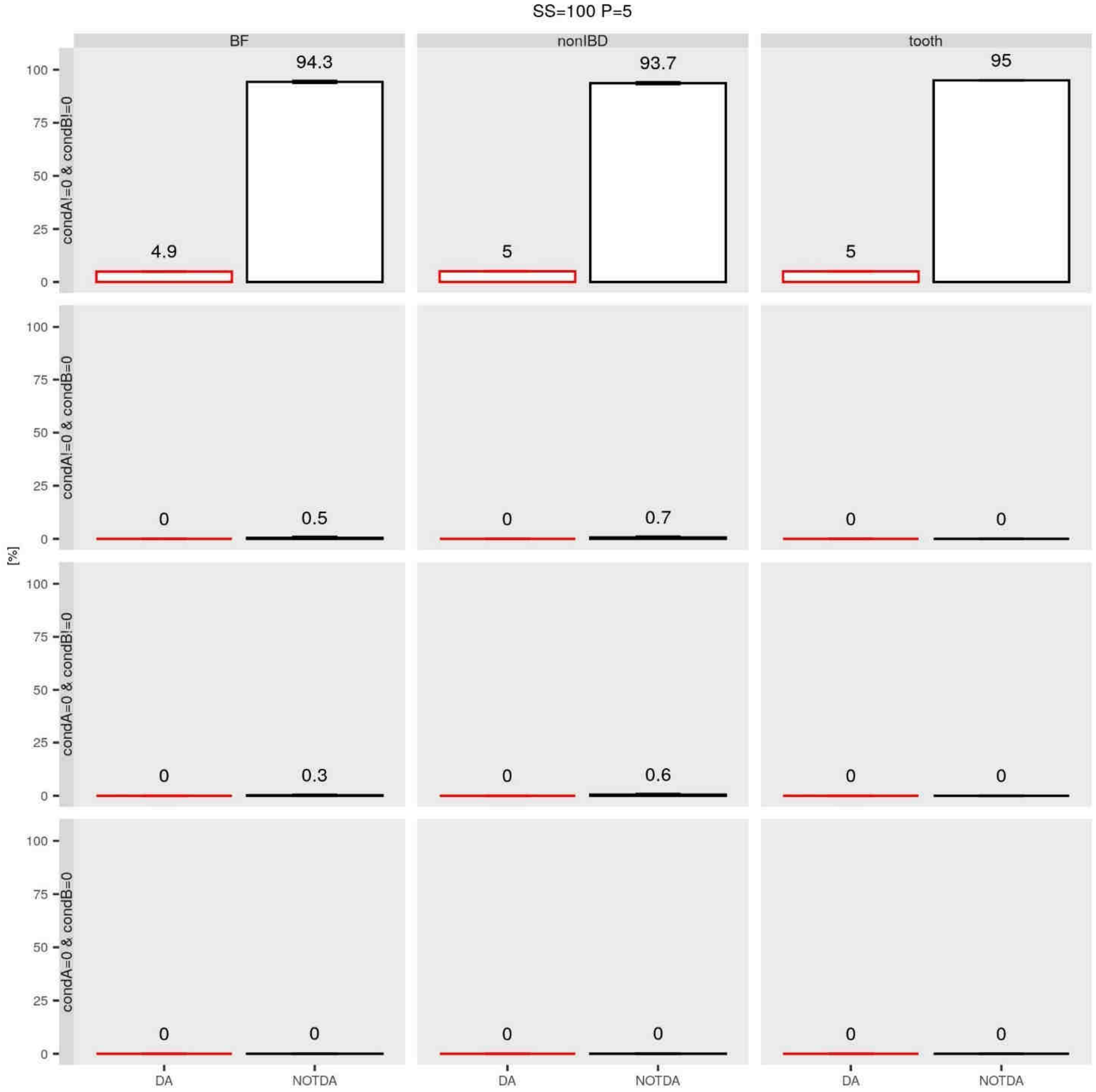

Figure 15: Percentages of features simulated as DA and NOT DA in the main scenario with simulated DA features in relation to the following cases: both conditions not null (condA!=0 & condB!=0), only condition B all null (condA!=0 & condB=0), only condition A all null (condA=0 & condB!=0), both conditions all null (condA=0 & condB=0). The percentages shown are averaged between the 50 simulations obtained by setting the sample size (SS) to 100 and with a percentage of inserted DA features (P) of 5%.

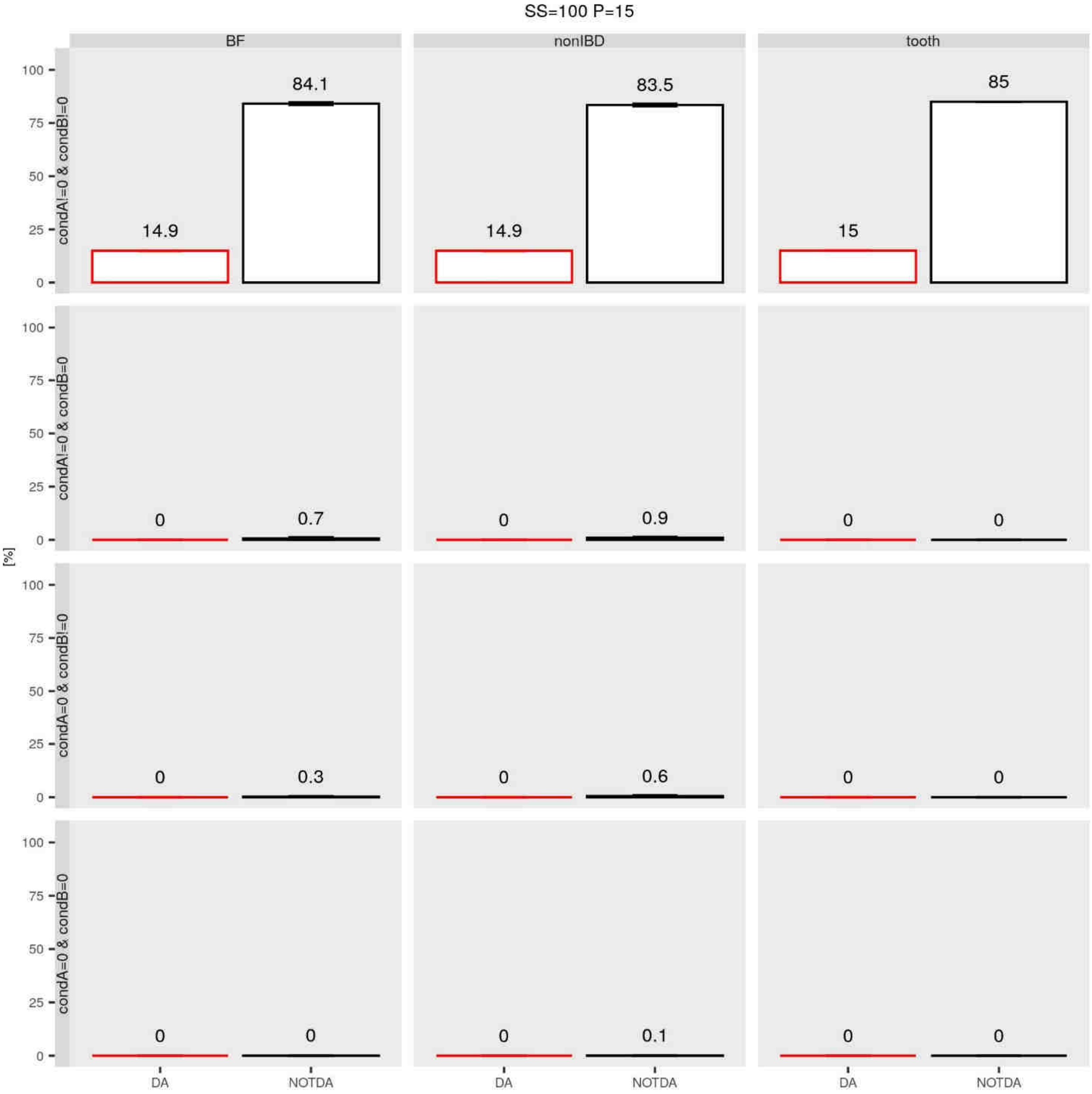

Figure 16: Percentages of features simulated as DA and NOT DA in the main scenario with simulated DA features in relation to the following cases: both conditions not null (condA!=0 & condB!=0), only condition B all null (condA!=0 & condB=0), only condition A all null (condA=0 & condB!=0), both conditions all null (condA=0 & condB=0). The percentages shown are averaged between the 50 simulations obtained by setting the sample size (SS) to 100 and with a percentage of inserted DA features (P) of 15%.

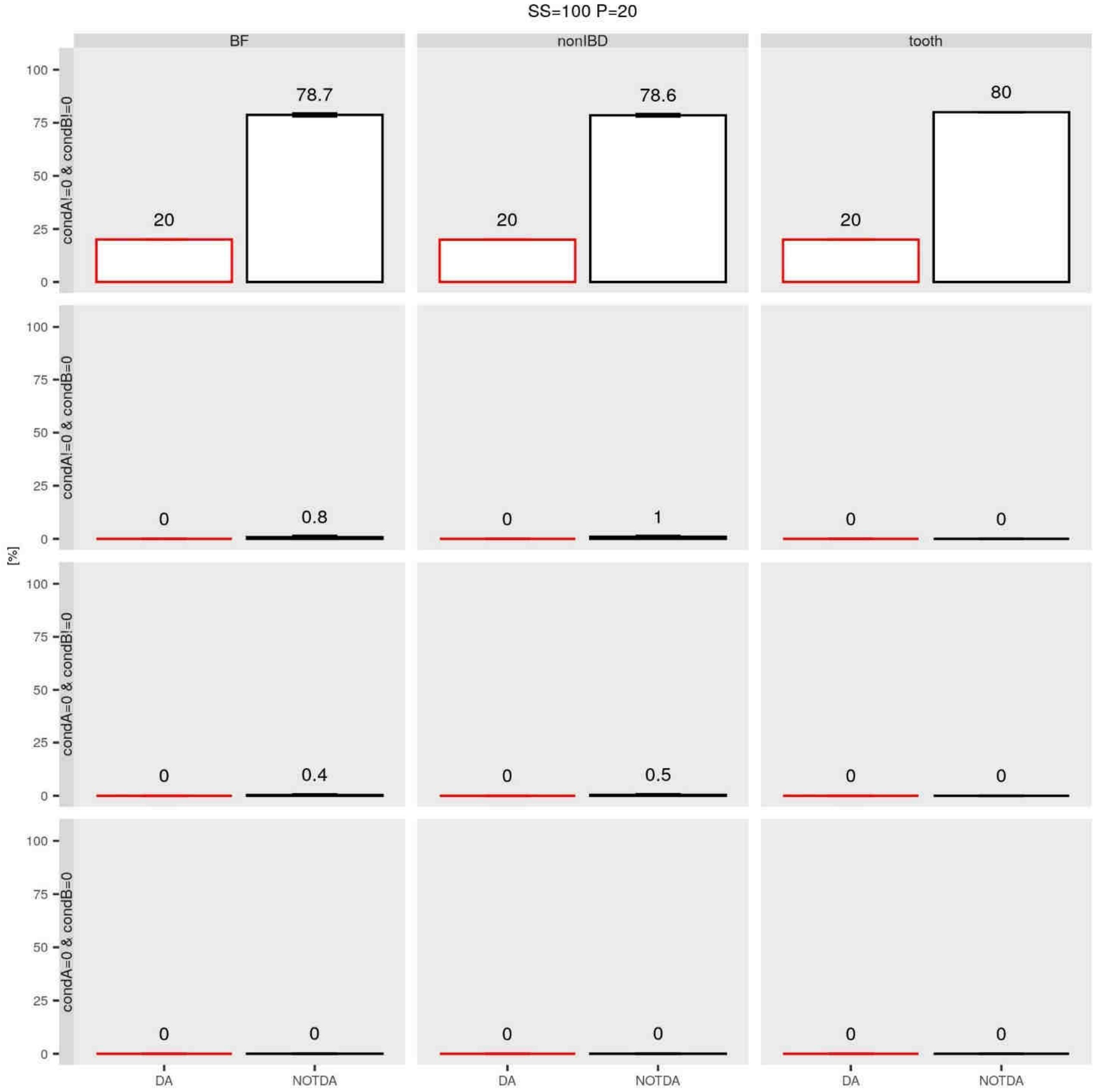

Figure 17: Percentages of features simulated as DA and NOT DA in the main scenario with simulated DA features in relation to the following cases: both conditions not null (condA!=0 & condB!=0), only condition B all null (condA!=0 & condB=0), only condition A all null (condA=0 & condB!=0), both conditions all null (condA=0 & condB=0). The percentages shown are averaged between the 50 simulations obtained by setting the sample size (SS) to 100 and with a percentage of inserted DA features (P) of 20%.

8 Results

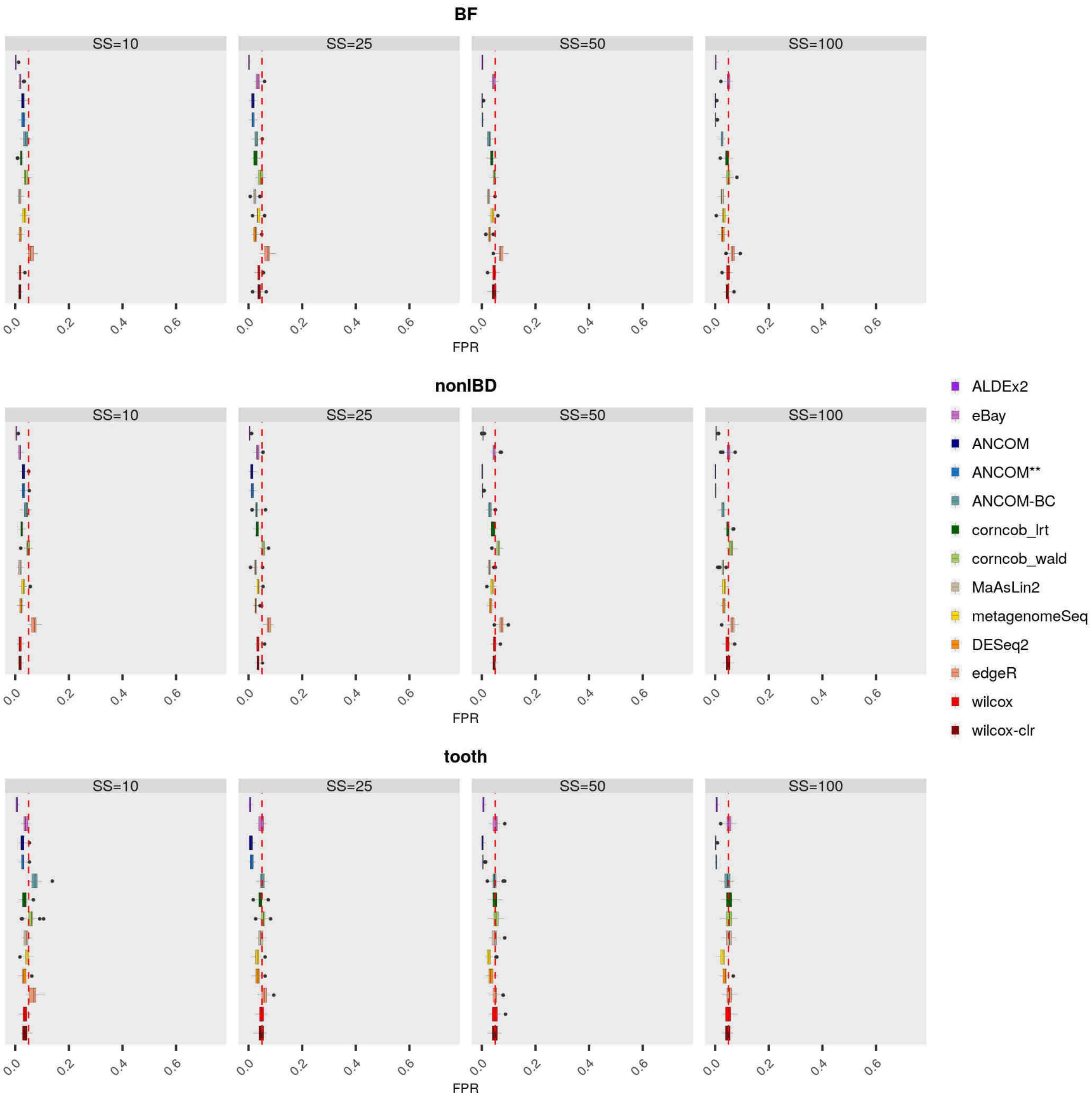

Figure 18. Boxplot of the False Positive Rate (FPR) distribution across the 50 simulations of each differential abundance method for each dataset considered in the comparison in the scenario without simulated DA features. In each set of boxes corresponding to the dataset, tools are on rows, while different sample size (SS) values are on columns. The ANCOM** label refers to the method run without performing the underlying FDR adjustment.

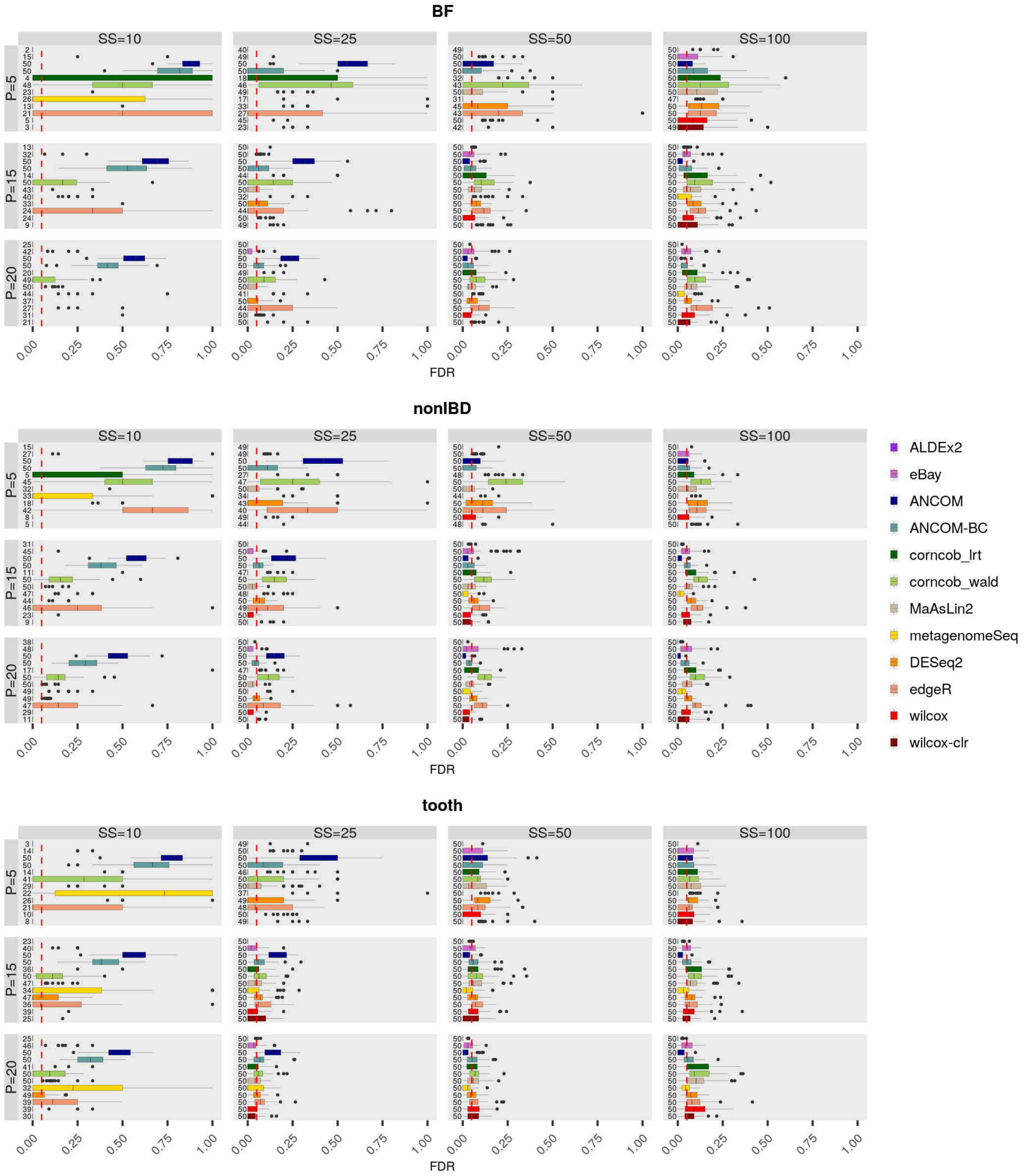

Figure 19. Boxplot of the False Discovery Rate (FDR) distribution across the 50 simulations of each differential abundance method for each dataset considered in the comparison in the main scenario with simulated DA features. In each set of boxes corresponding to the dataset, different percentages (P) of simulated DA features are on rows, while different sample size (SS) values are on columns. The number of runs that provide a defined value of FDR is shown on the left of each boxplot.

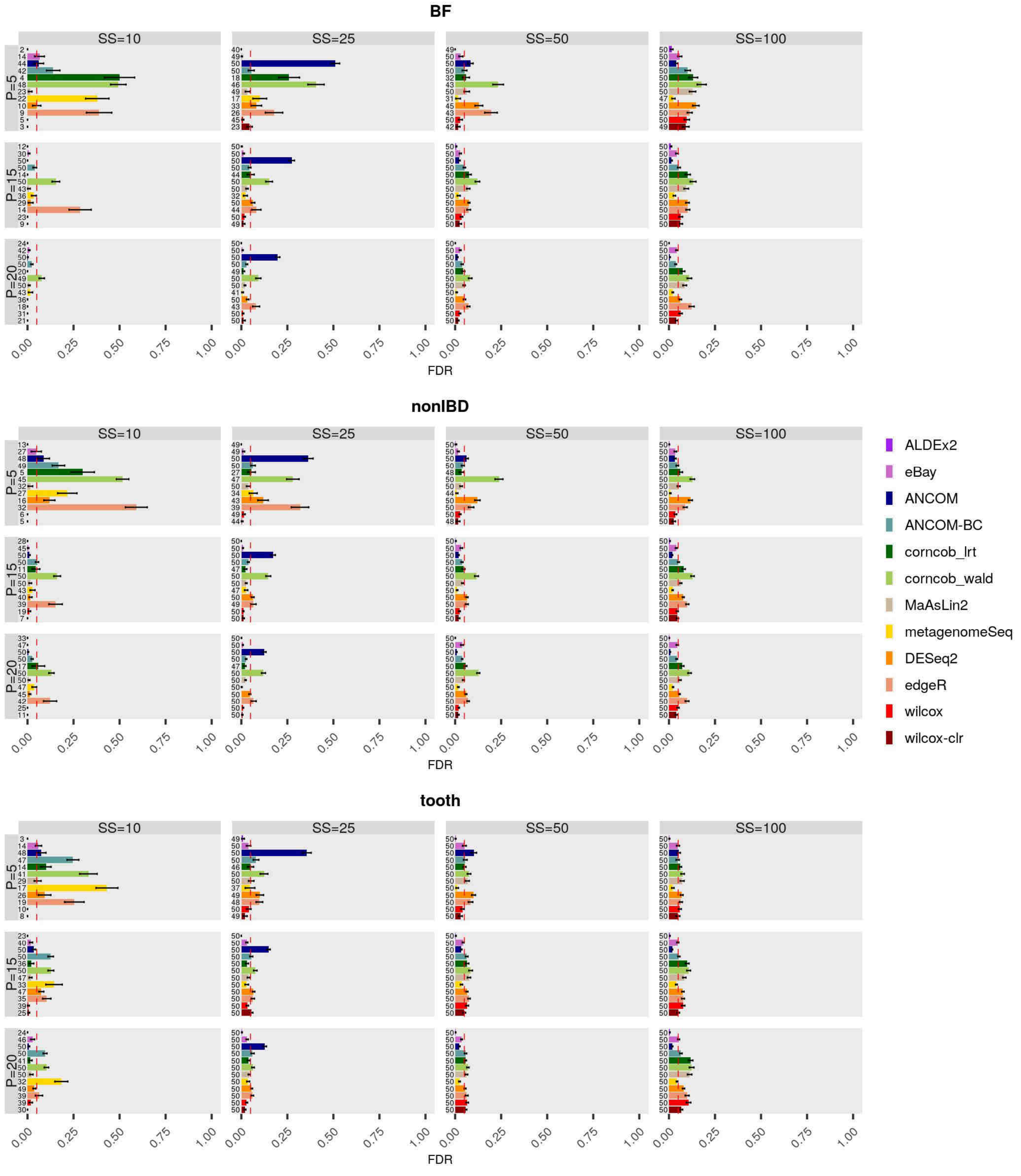

Figure 20. False Discovery Rate (FDR) of each differential abundance method for each dataset considered in the comparison in the scenario considering structural zeros as TN. In each set of boxes corresponding to the dataset, different percentages (P) of simulated DA features are on rows, while different sample size (SS) values are on columns. The FDR values are averaged over the 50 simulations and the bars show the standard error. The number of runs that provide a defined value of FDR is shown at the beginning of the bars.

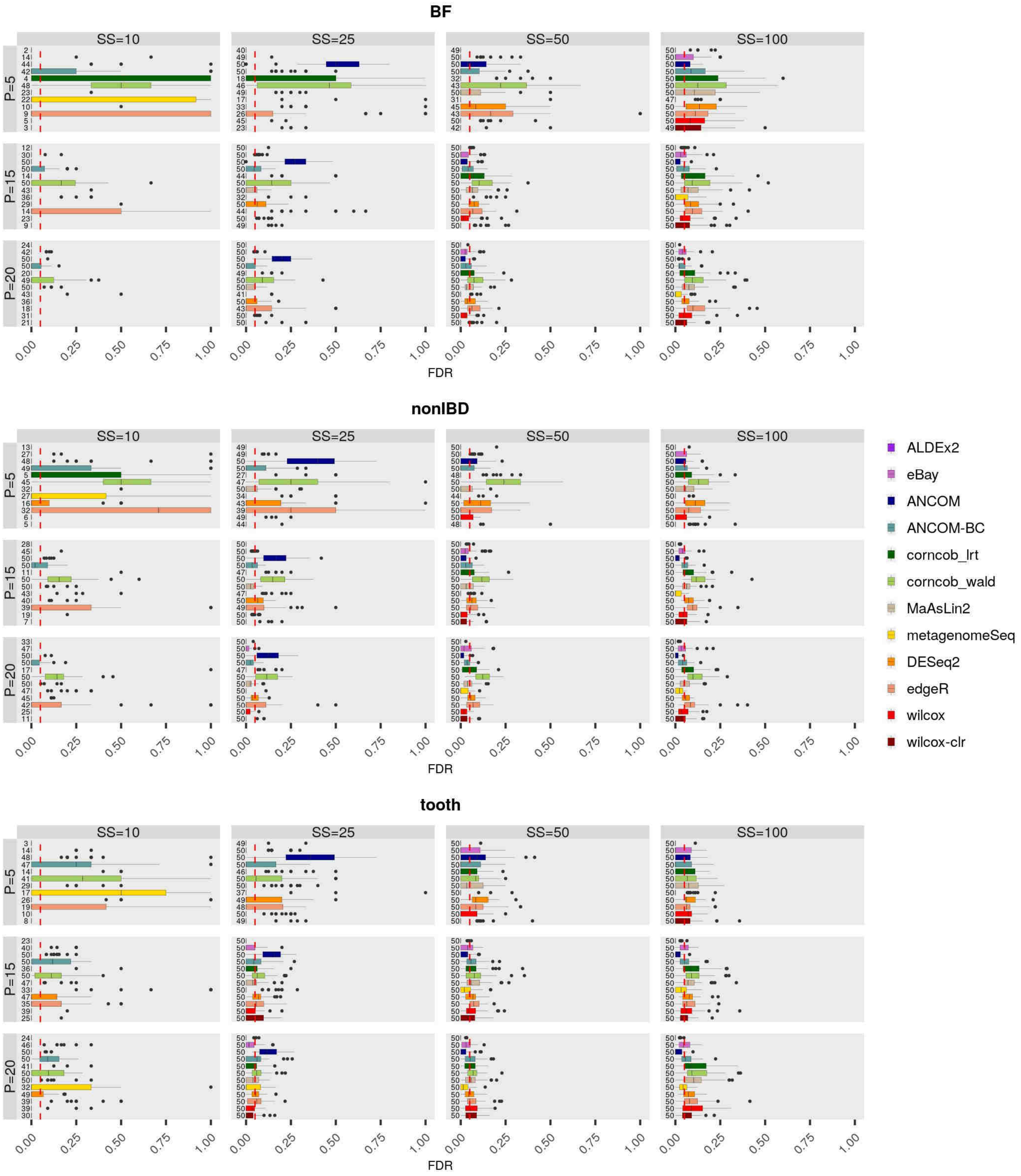

Figure 21. Boxplot of the False Discovery Rate (FDR) distribution across the 50 simulations of each differential abundance method for each dataset considered in the comparison in the scenario considering structural zeros as TN. In each set of boxes corresponding to the dataset, different percentages (P) of simulated DA features are on rows, while different sample size (SS) values are on columns. The number of runs that provide a defined value of FDR is shown on the left of each boxplot.

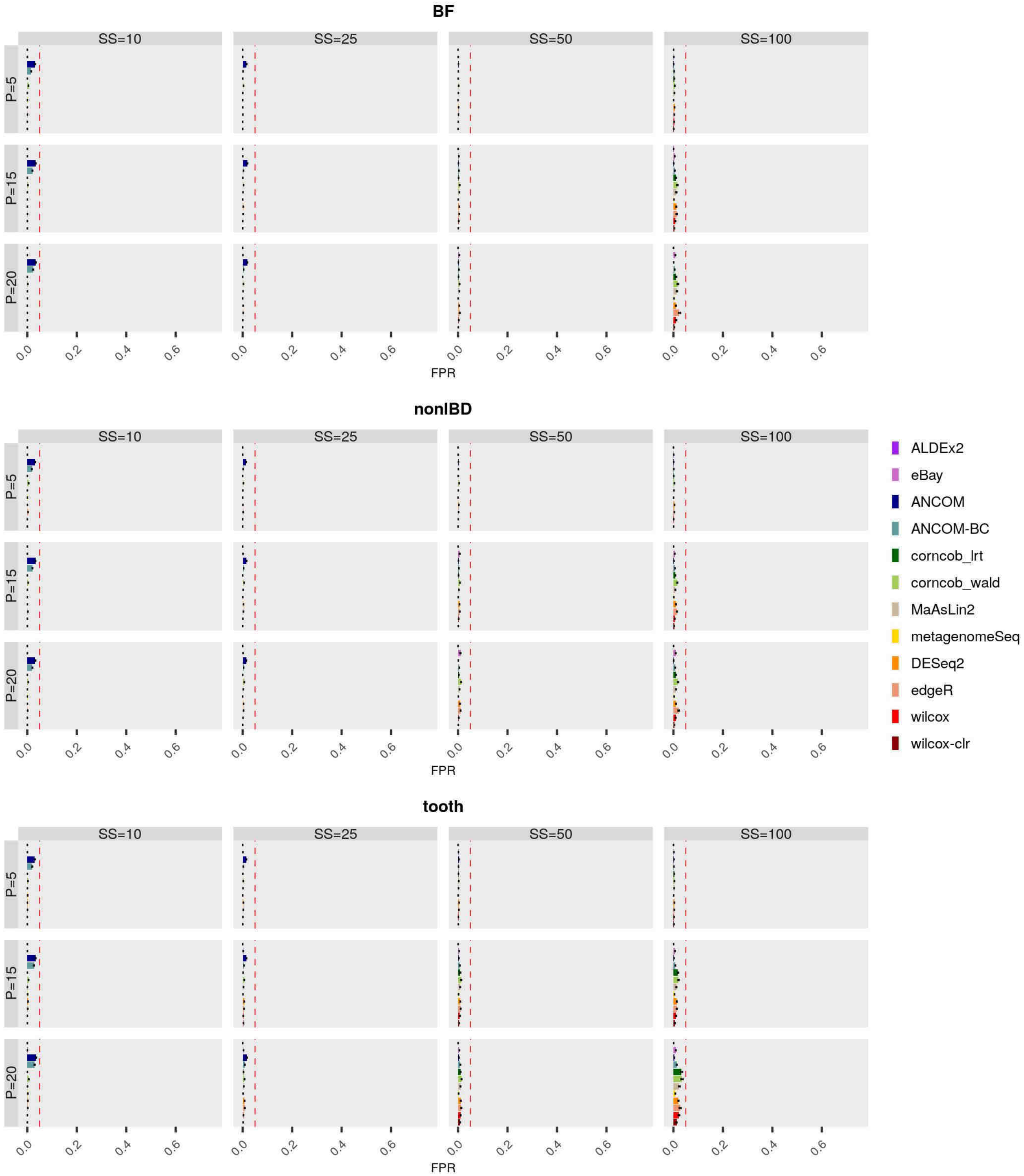

Figure 22. False Positive Rate (FPR) of each differential abundance method for each dataset considered in the comparison in the main scenario with simulated DA features. In each set of boxes corresponding to the dataset, different percentages (P) of DA features a are on rows, while different sample size (SS) values are on columns. The FPR values are averaged over the 50 simulations and the bars show the standard error.

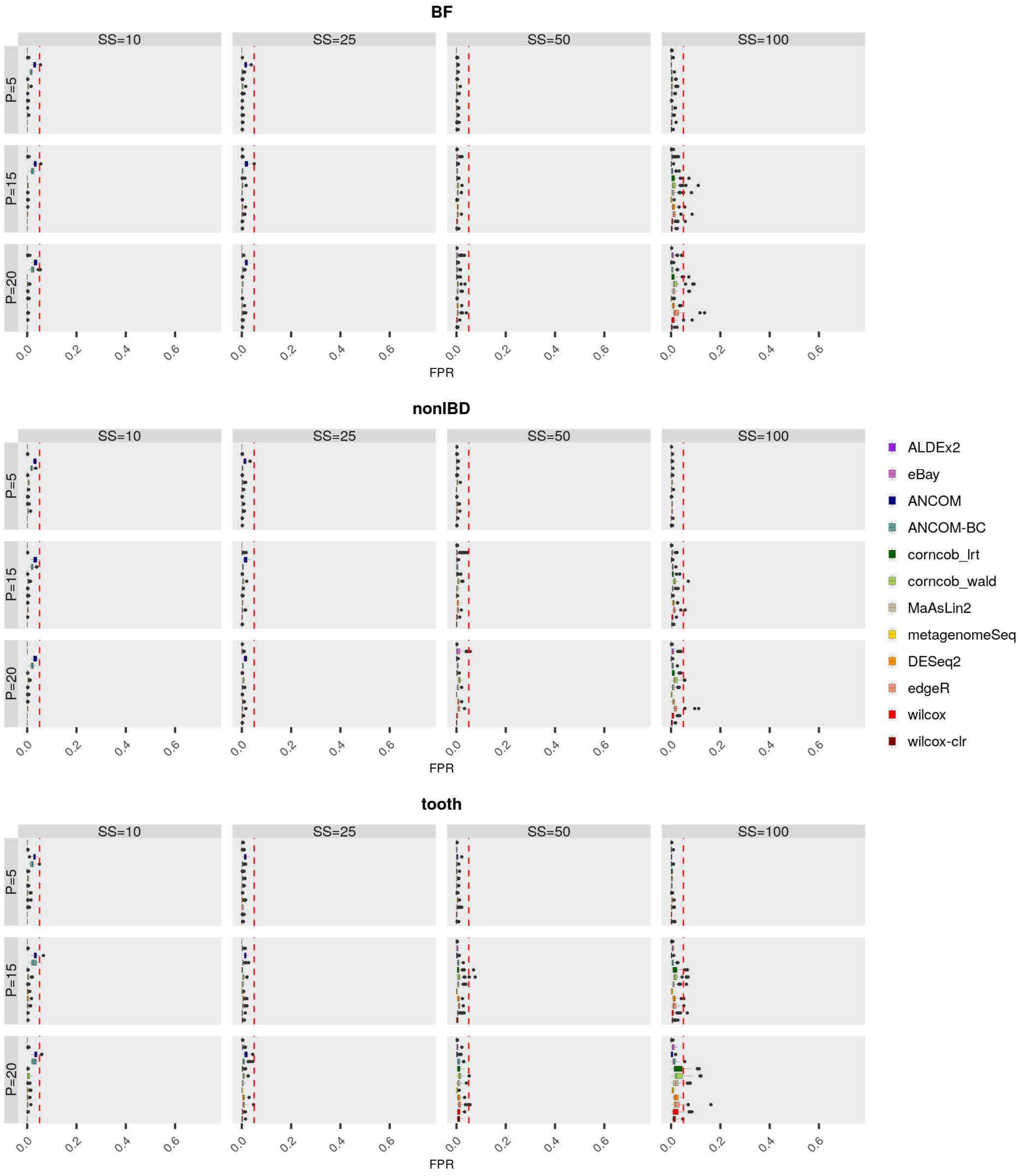

Figure 23. Boxplot of the False Positive Rate (FPR) distribution across the 50 simulations of each differential abundance method for each dataset considered in the comparison in the main scenario with simulated DA features. In each set of boxes corresponding to the dataset, different percentages (P) of simulated DA features are on rows, while different sample size (SS) values are on columns.

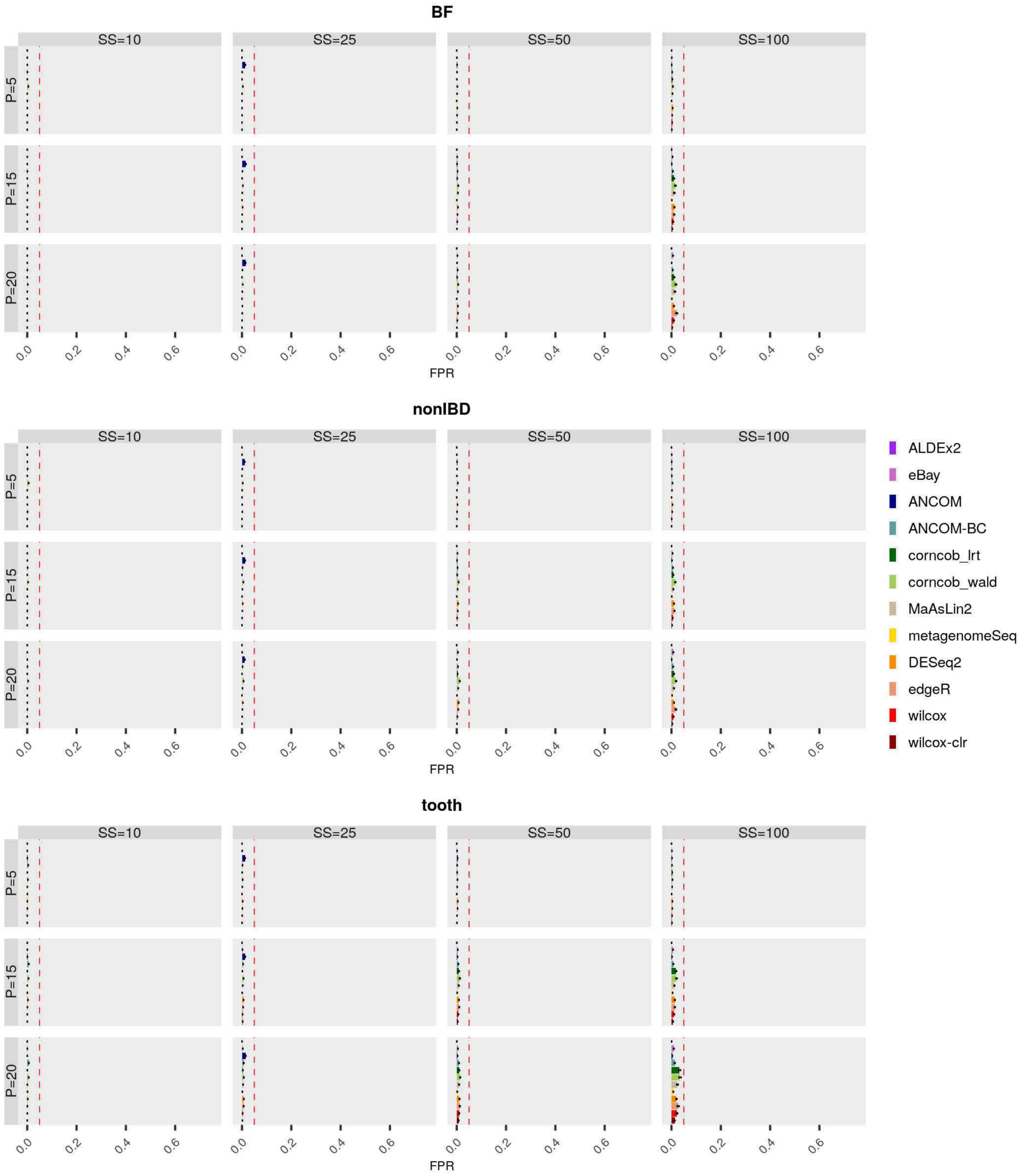

Figure 24. False Positive Rate (FPR) of each differential abundance method for each dataset considered in the comparison in the scenario considering structural zeros as TN. In each set of boxes corresponding to the dataset, different percentages (P) of simulated DA features are on rows, while different sample size (SS) values are on columns. The FPR values are averaged over the 50 simulations and the bars show the standard error.

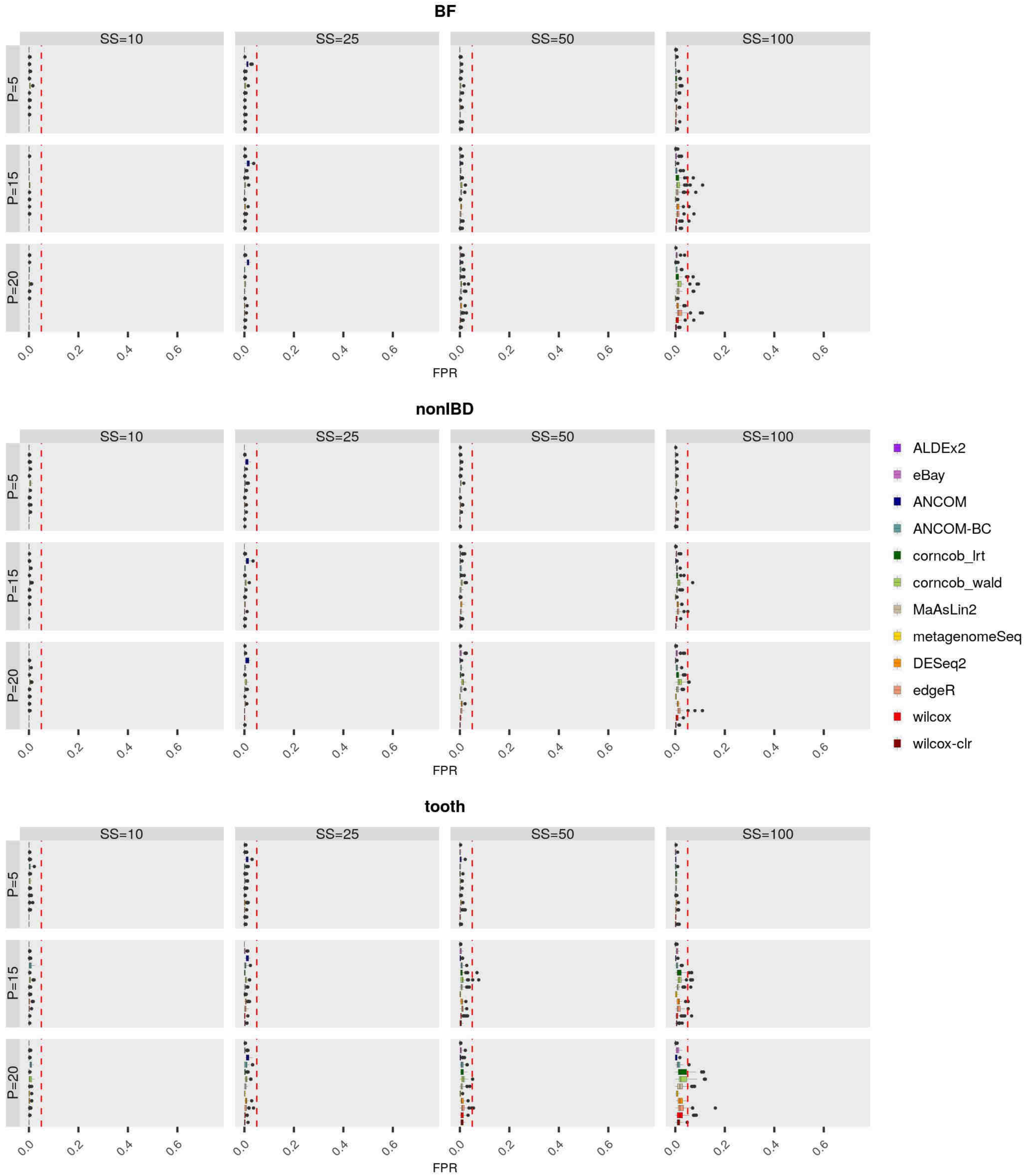

Figure 25. Boxplot of the False Positive Rate (FPR) distribution across the 50 simulations of each differential abundance method for each dataset considered in the comparison in the scenario considering structural zeros as TN. In each set of boxes corresponding to the dataset, different percentages (P) of simulated DA features are on rows, while different sample size (SS) values are on columns.

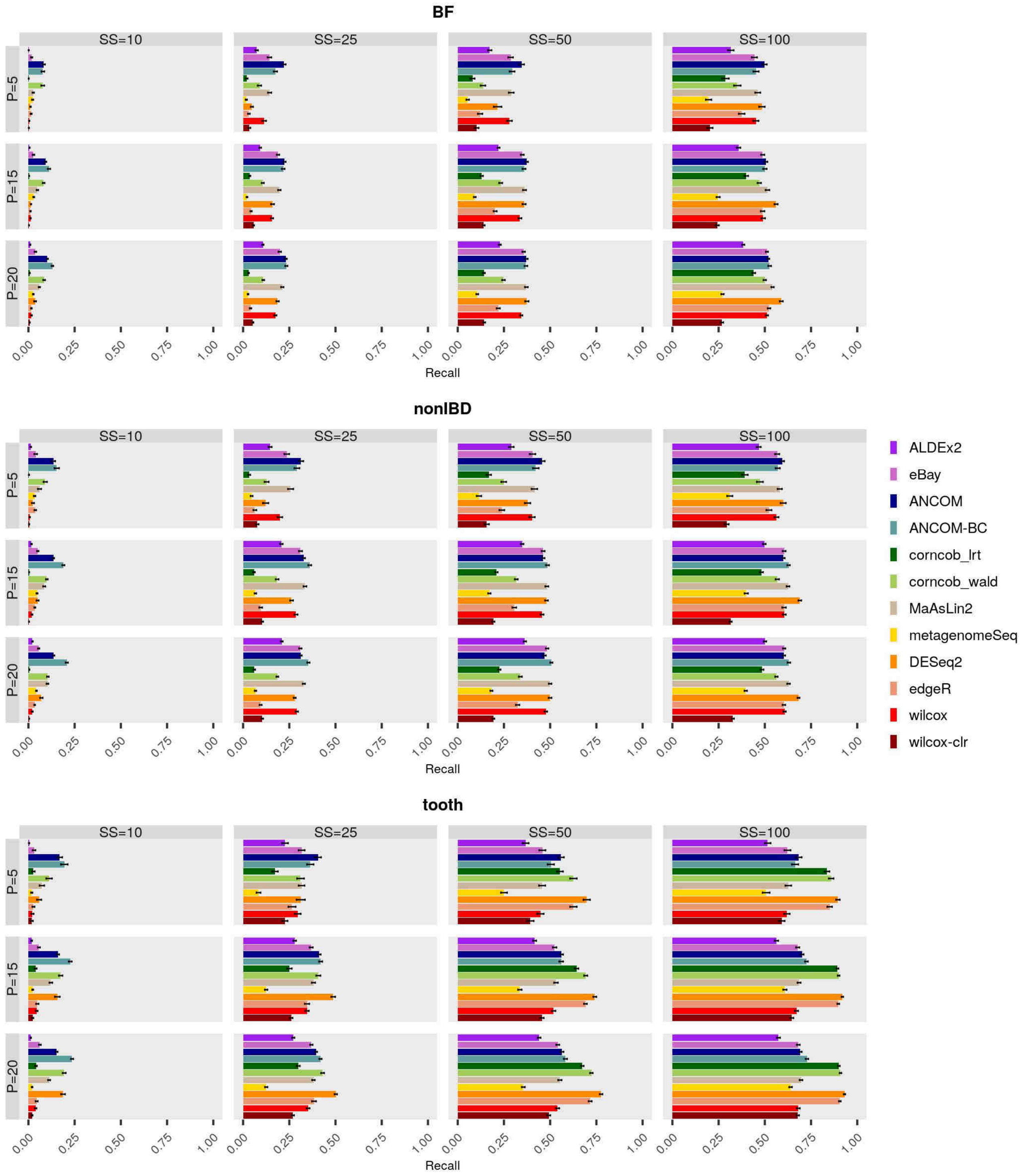

Figure 26. Recall of each differential abundance method for each dataset considered in the comparison in the main scenario with simulated DA features. In each set of boxes corresponding to the dataset, different percentages (P) of simulated DA features are on rows, while different sample size (SS) values are on columns. The recall values are averaged over the 50 simulations and the bars show the standard error.

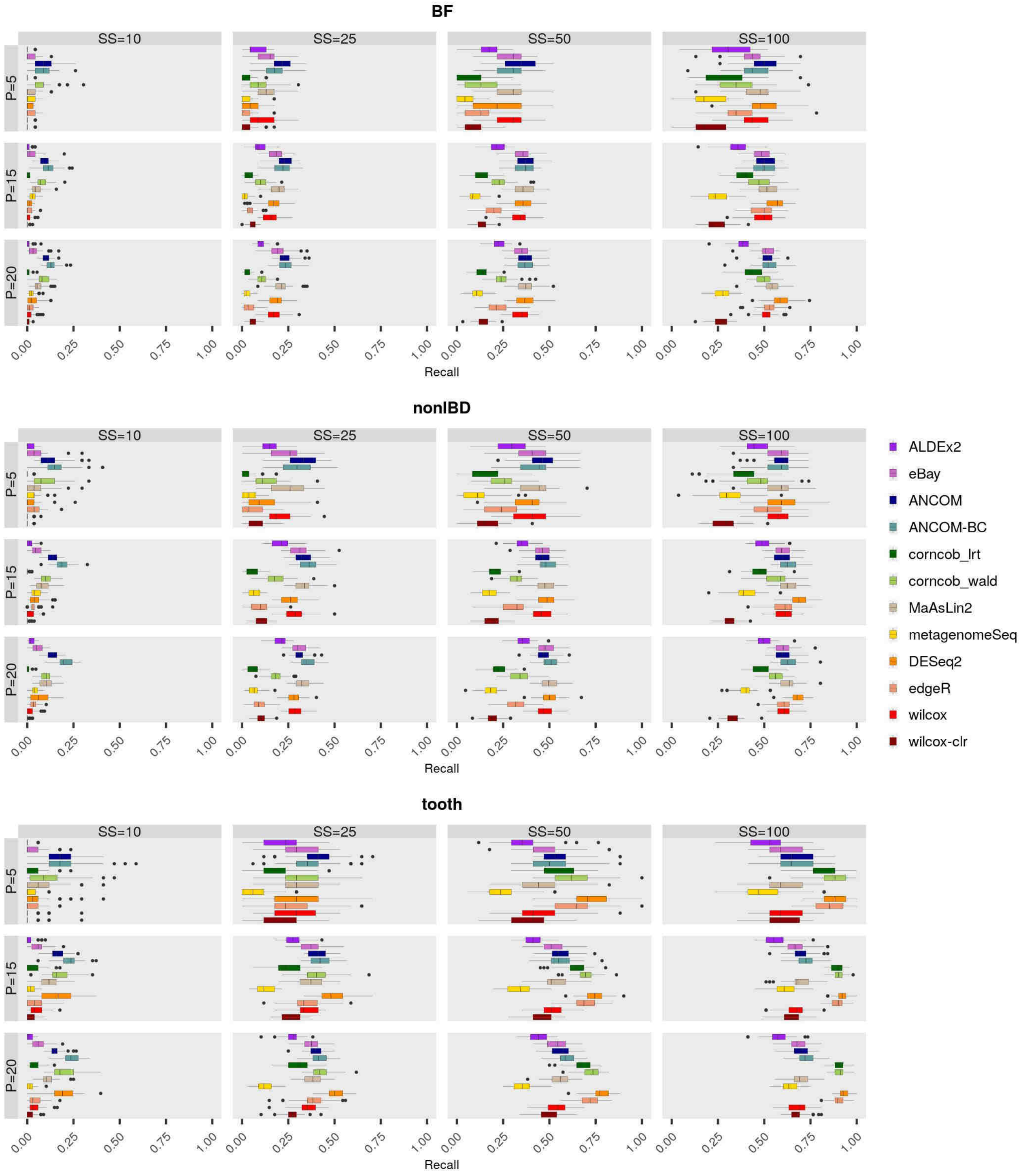

Figure 27. Boxplot of recall distribution across the 50 simulations of each differential abundance method for each dataset considered in the comparison in the main scenario with simulated DA features. In each set of boxes corresponding to the dataset, different percentages (P) of simulated DA features are on rows, while different sample size (SS) values are on columns.

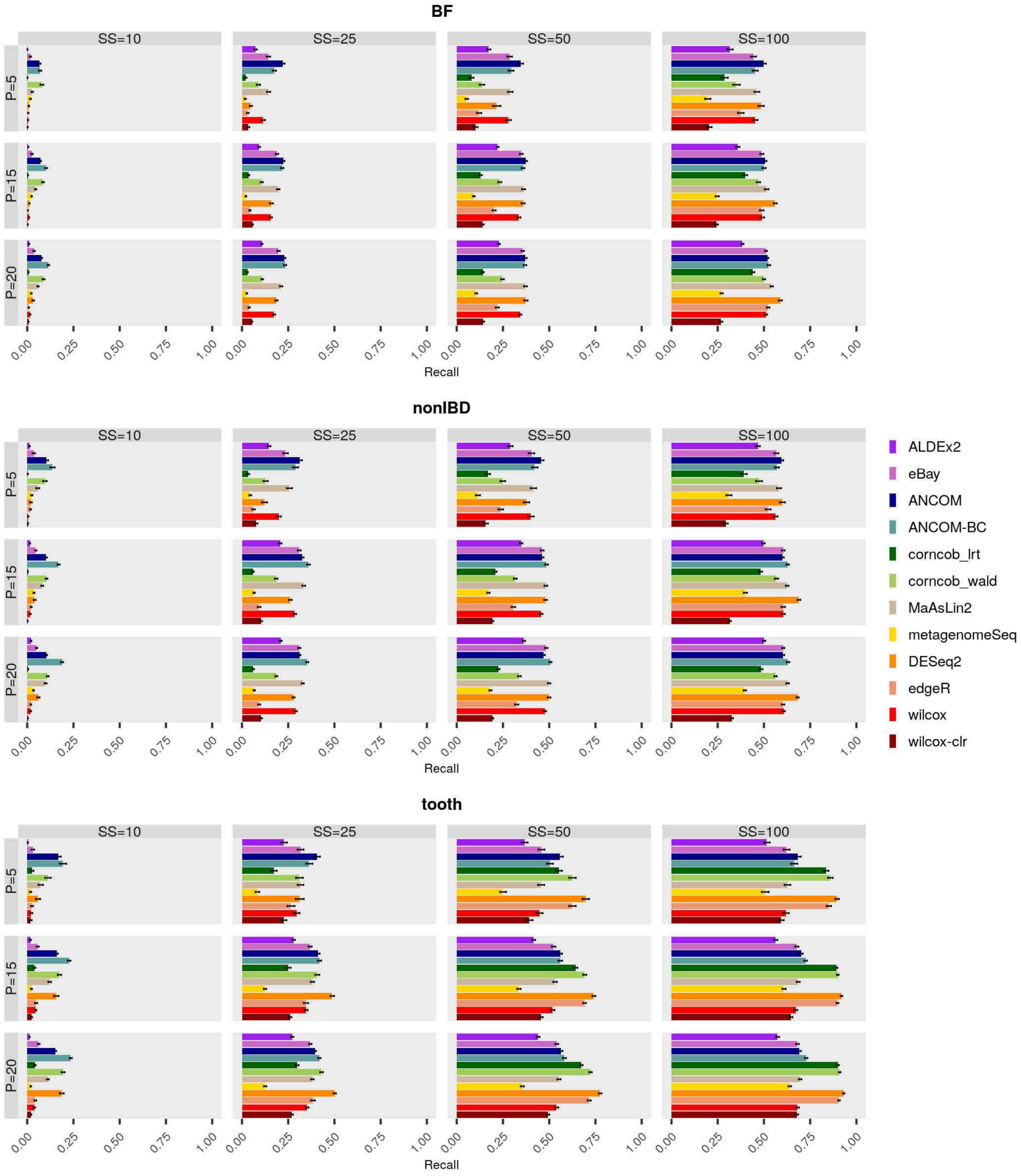

Figure 28. Recall of each differential abundance method for each dataset considered in the comparison in the scenario considering structural zeros as TN. In each set of boxes corresponding to the dataset, different percentages (P) of simulated DA features are on rows, while different sample size (SS) values are on columns. The recall values are averaged over the 50 simulations and the bars show the standard error.

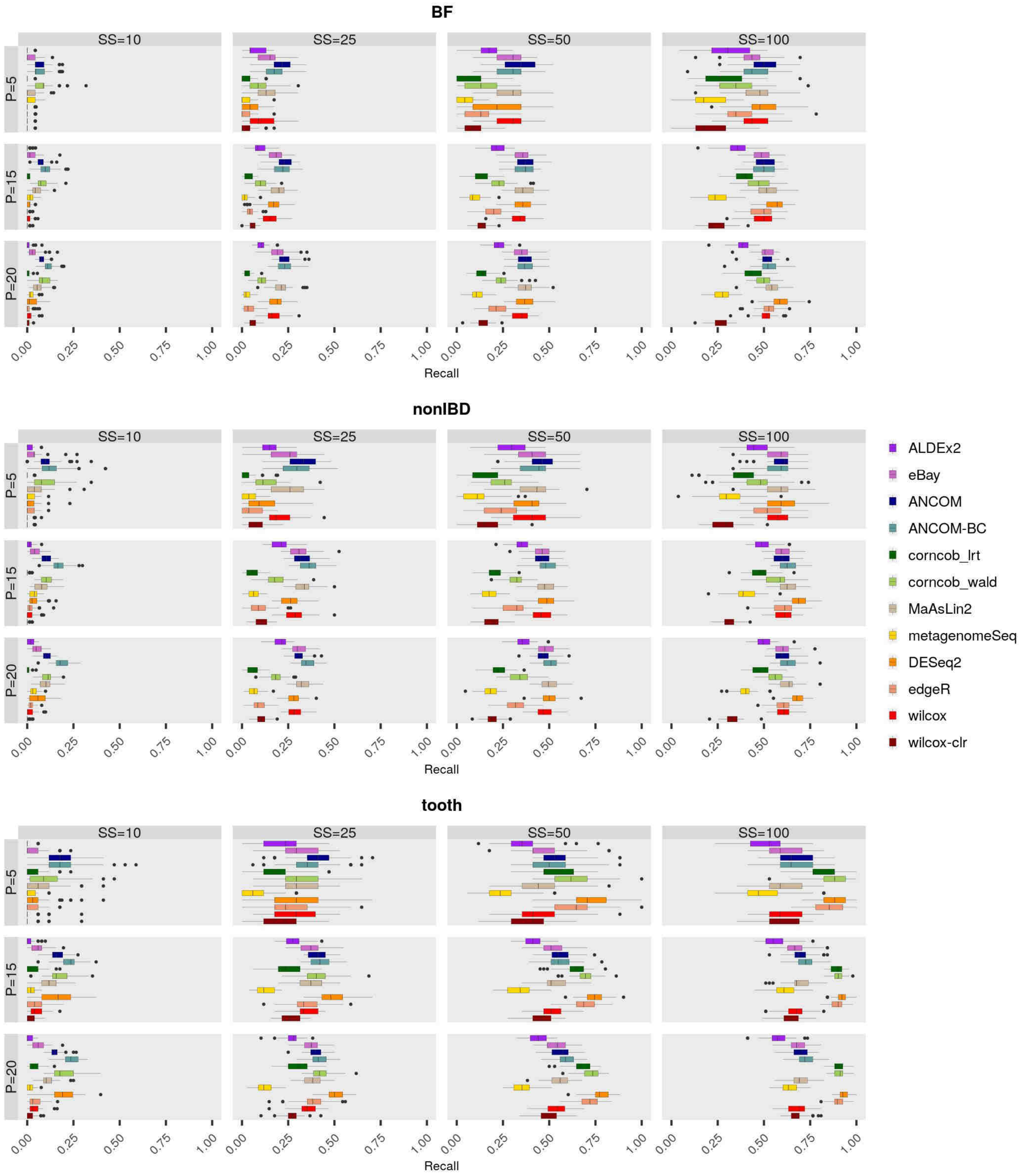

Figure 29. Boxplot of recall distribution across the 50 simulations of each differential abundance method for each dataset considered in the comparison in the scenario considering structural zeros as TN. In each set of boxes corresponding to the dataset, different percentages (P) of simulated DA features are on rows, while different sample size (SS) values are on columns.

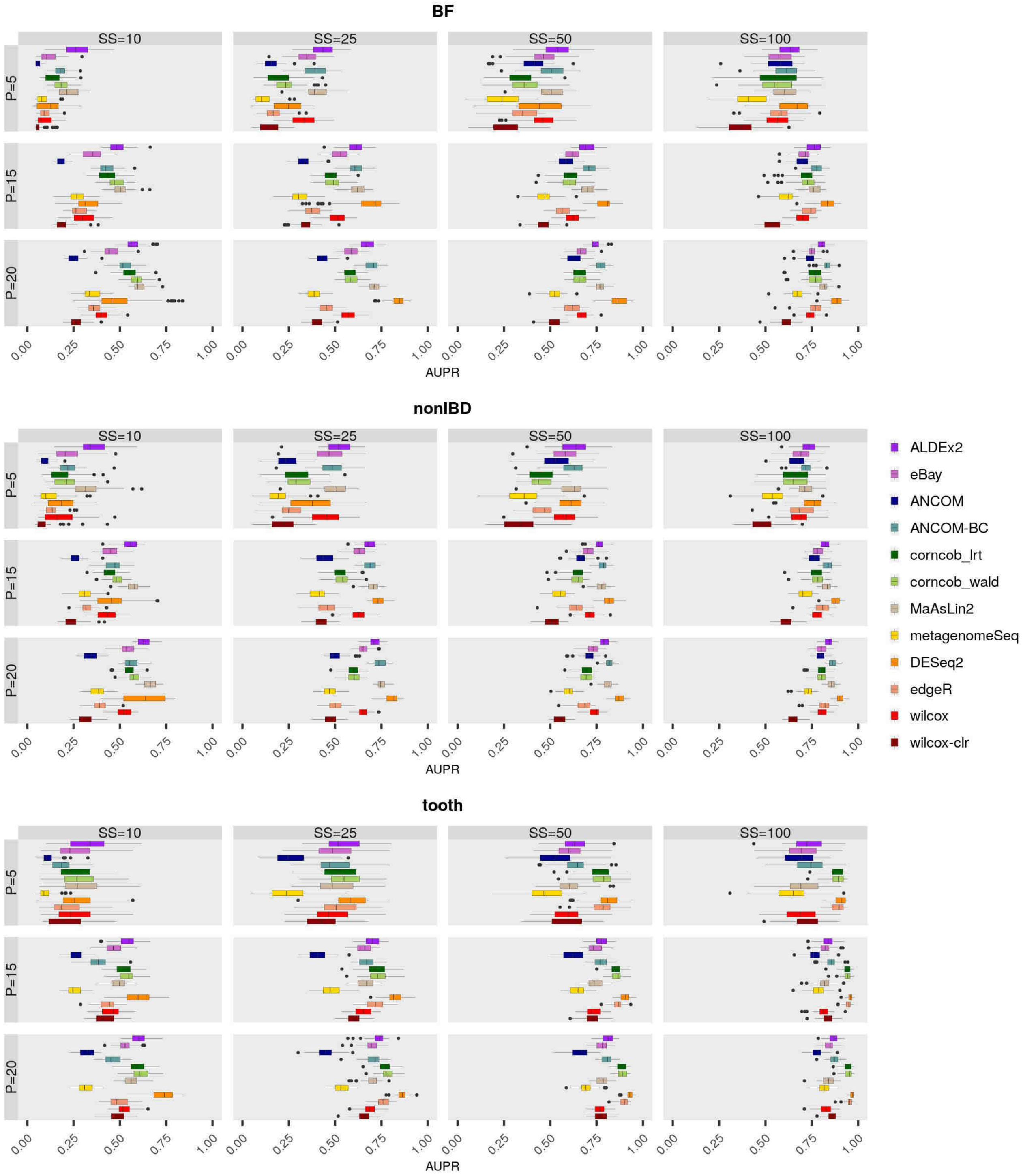

Figure 30. Boxplot of the Area Under Precision-Recall curves (AUPR) of each differential abundance method for each dataset considered in the comparison in the main scenario with simulated DA features. In each set of boxes corresponding to the dataset, different percentages (P) of simulated DA features are on rows, while different sample size (SS) values are on columns.

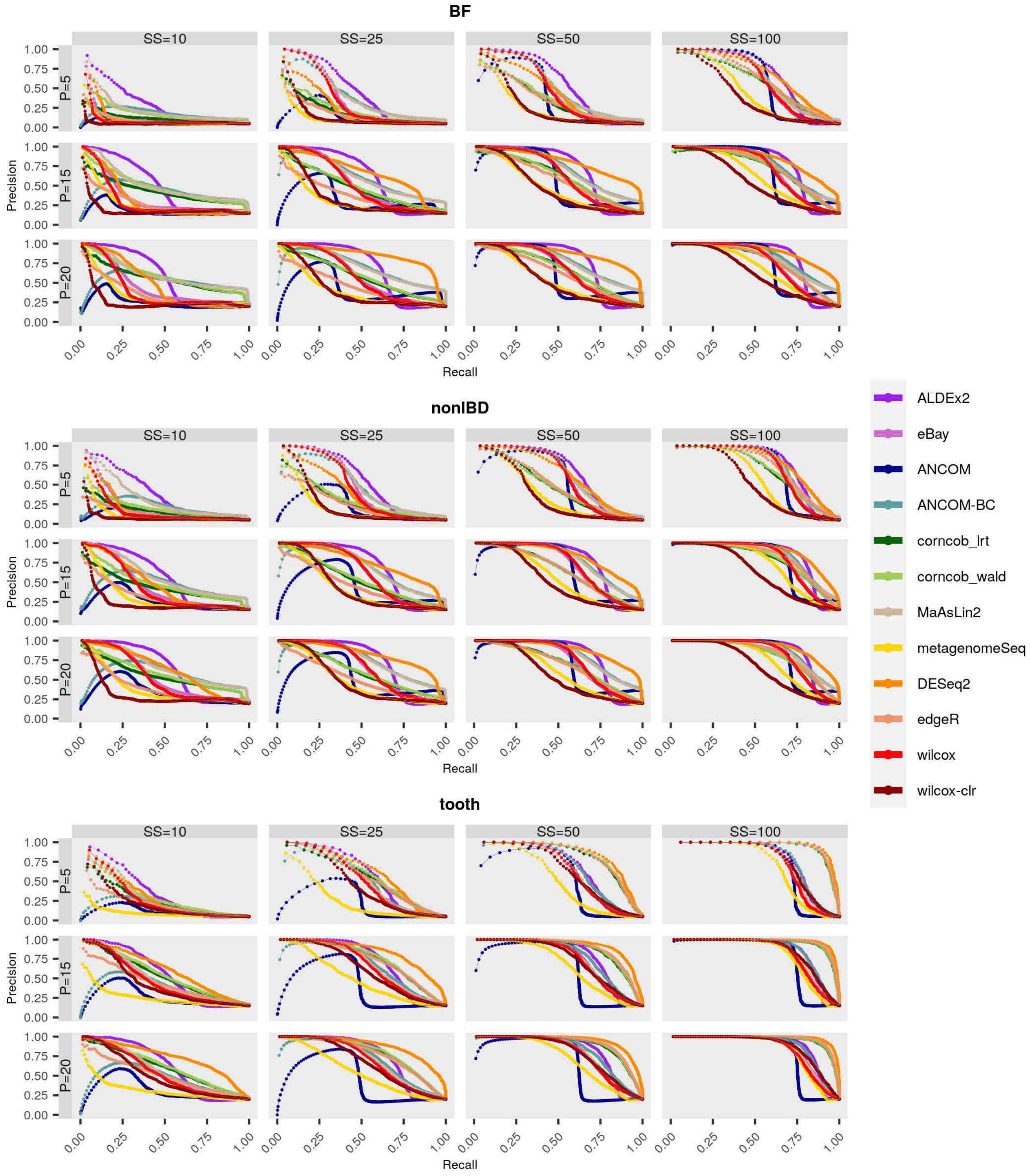

Figure 31. Mean Precision-Recall curves of each differential abundance method for each dataset considered in the comparison in the main scenario with simulated DA features. In each set of boxes corresponding to the dataset, different percentages (P) of simulated DA features are on rows, while different sample size (SS) values are on columns. The mean pr-curve is defined as the average of the *i*-th precision value and the *i*-th recall value over the 50 simulations.

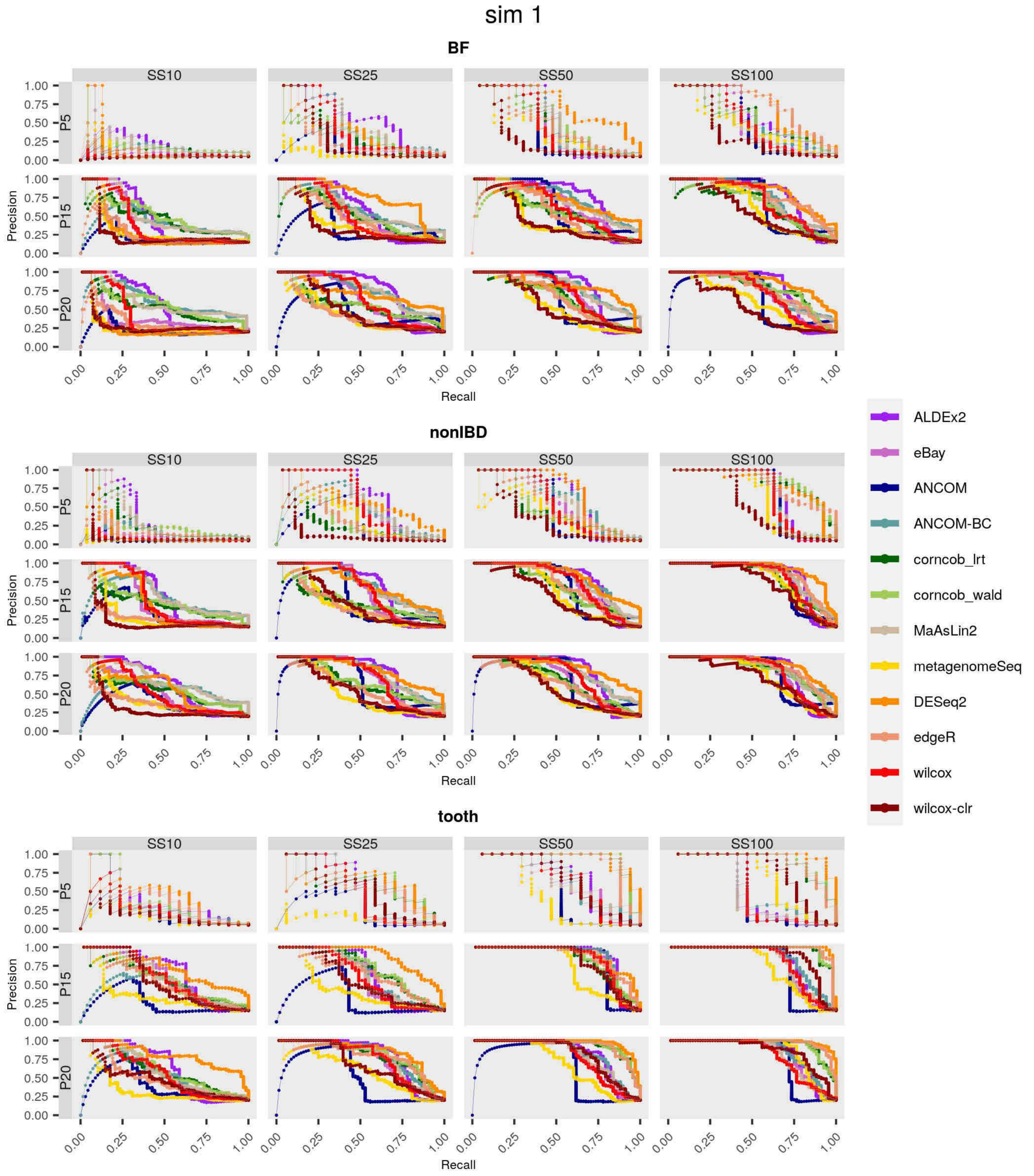

Figure 32. Simulation 1. Precision-Recall curves of each differential abundance method for each dataset considered in the comparison in the main scenario with simulated DA features. In each set of boxes corresponding to the dataset, different percentages (P) of simulated DA features are on rows, while different sample size (SS) values are on columns.

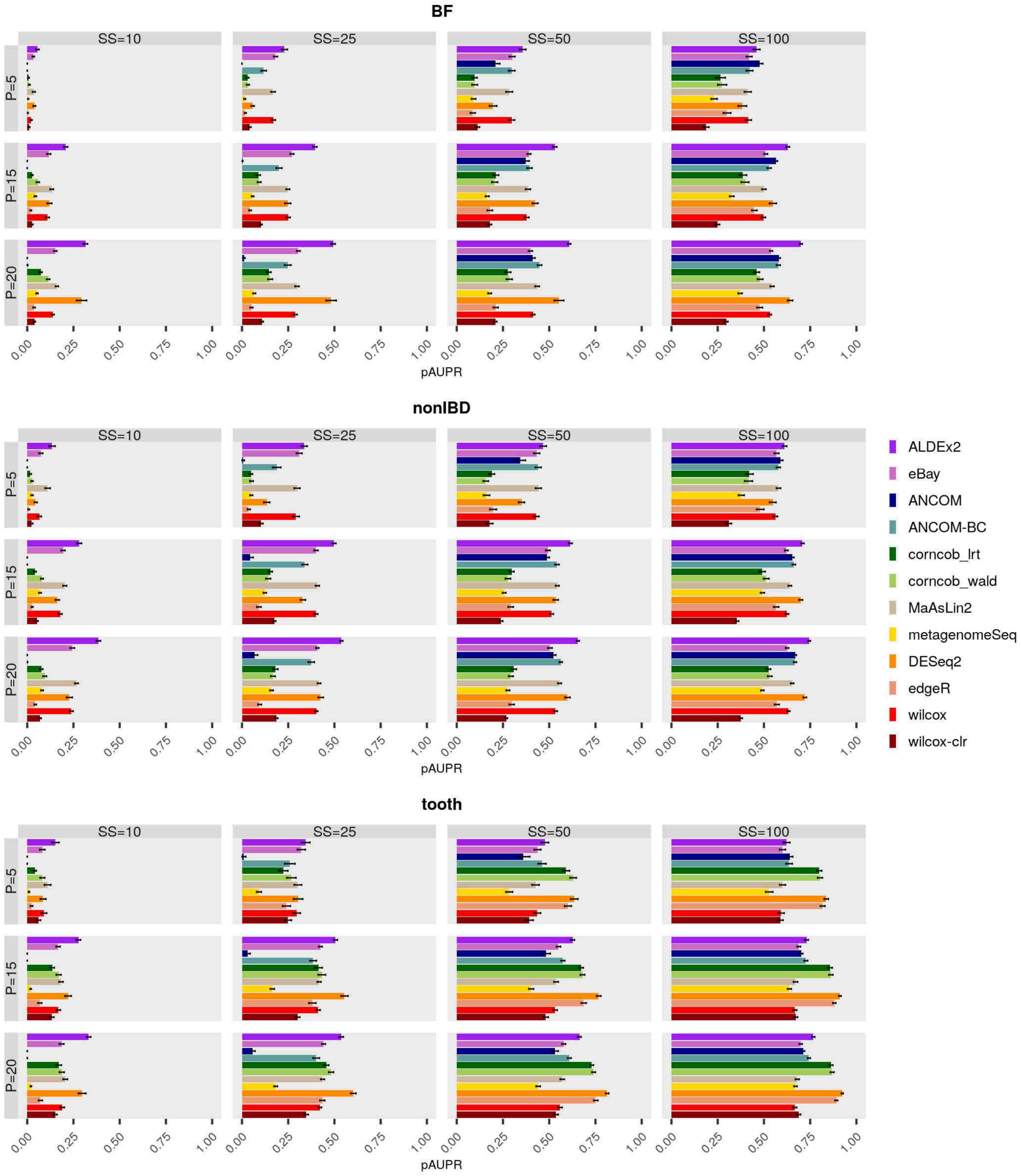

Figure 33. Partial Area Under Precision-Recall curves (pAUPR) of each differential abundance method for each dataset considered in the comparison in the main scenario with simulated DA features. In each set of boxes corresponding to the dataset, different percentages (P) of simulated DA features are on rows, while different sample size (SS) values are on columns. The pAUPR values are averaged over the 50 simulations and the bars show the standard error.

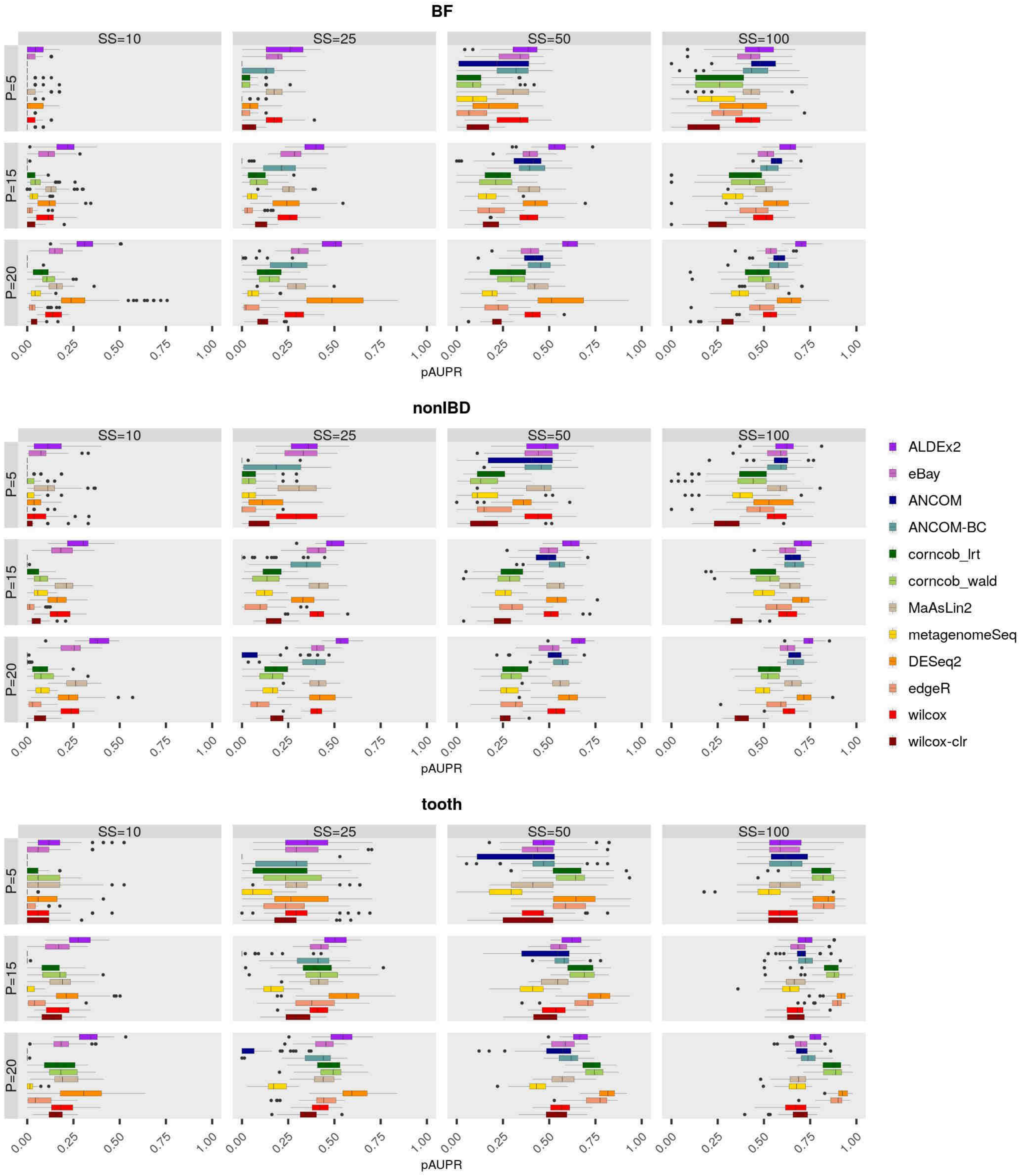

Figure 34. Boxplot of the partial Area Under Precision-Recall curves (pAUPR) of each differential abundance method for each dataset considered in the comparison in the main scenario with simulated DA features. In each set of boxes corresponding to the dataset, different percentages (P) of simulated DA features are on rows, while different sample size (SS) values are on columns.

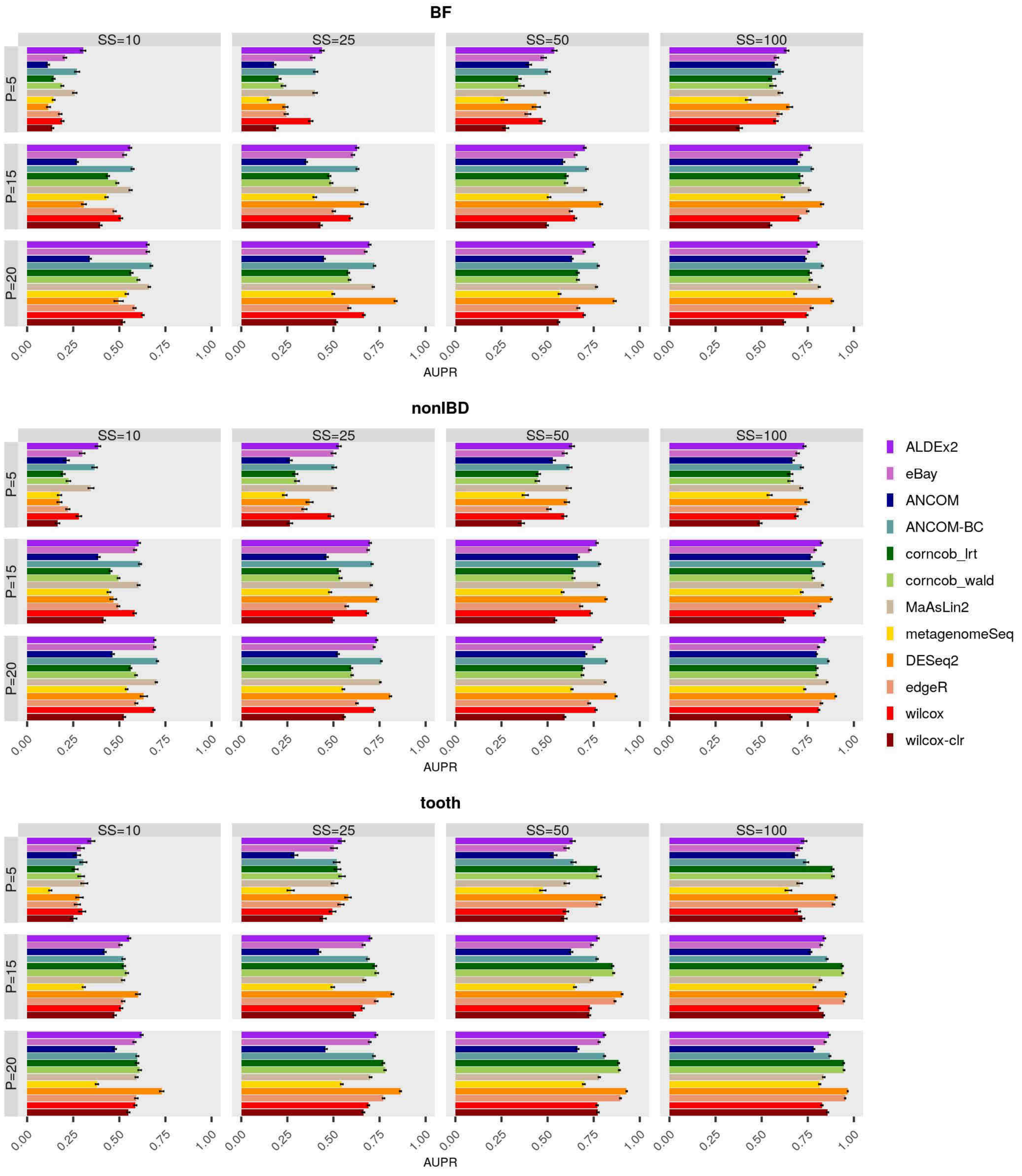

Figure 35. Area Under Precision-Recall curves (AUPR) of each differential abundance method for each dataset considered in the comparison in the scenario considering structural zeros as TN. In each set of boxes corresponding to the dataset, different percentages (P) of simulated DA features are on rows, while different sample size (SS) values are on columns. The AUPR values are averaged over the 50 simulations and the bars show the standard error.

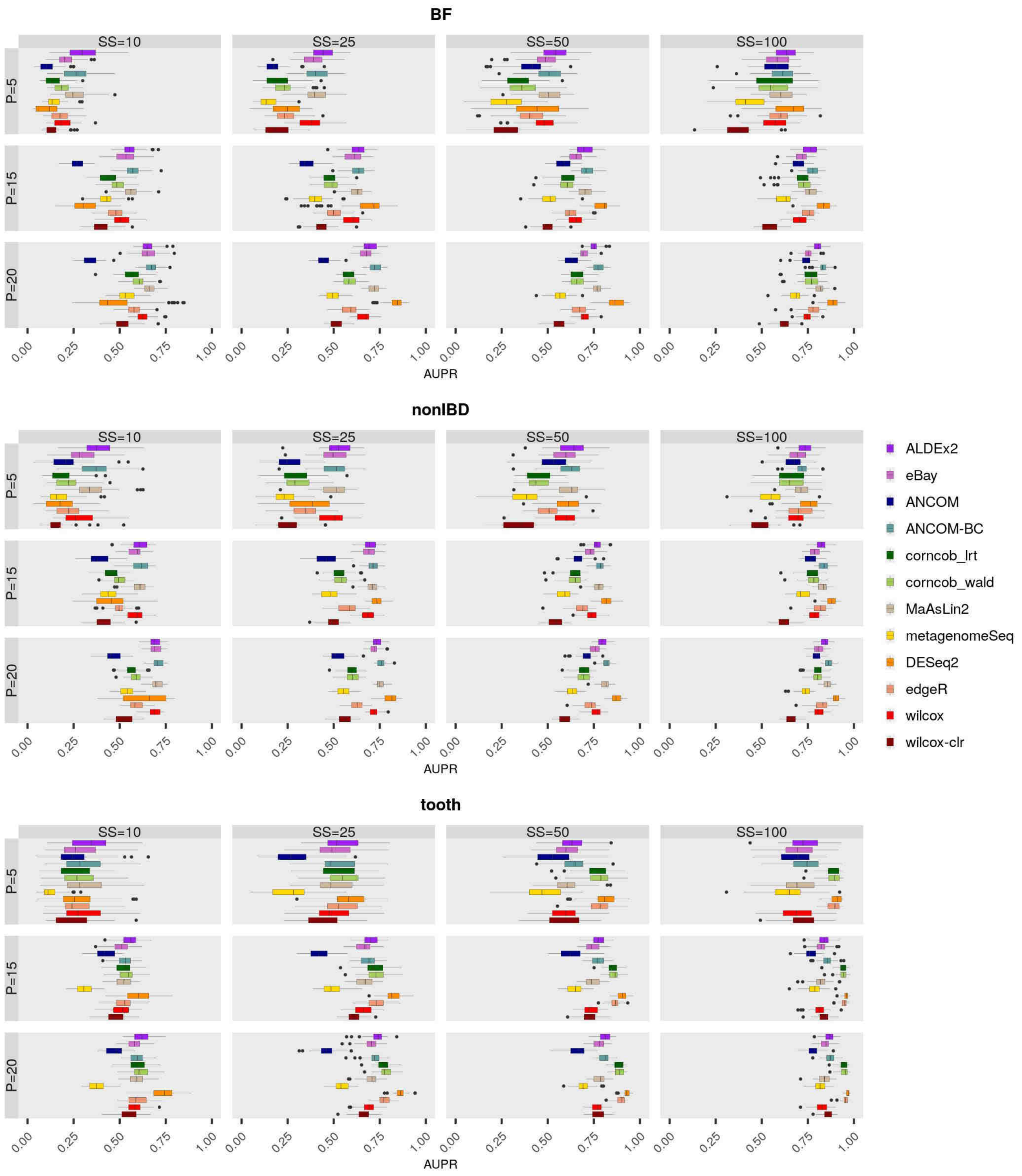

Figure 36. Boxplot of the Area Under Precision-Recall curves (AUPR) of each differential abundance method for each dataset considered in the comparison in the scenario considering structural zeros as TN. In each set of boxes corresponding to the dataset, different percentages (P) of simulated DA features are on rows, while different sample size (SS) values are on columns.

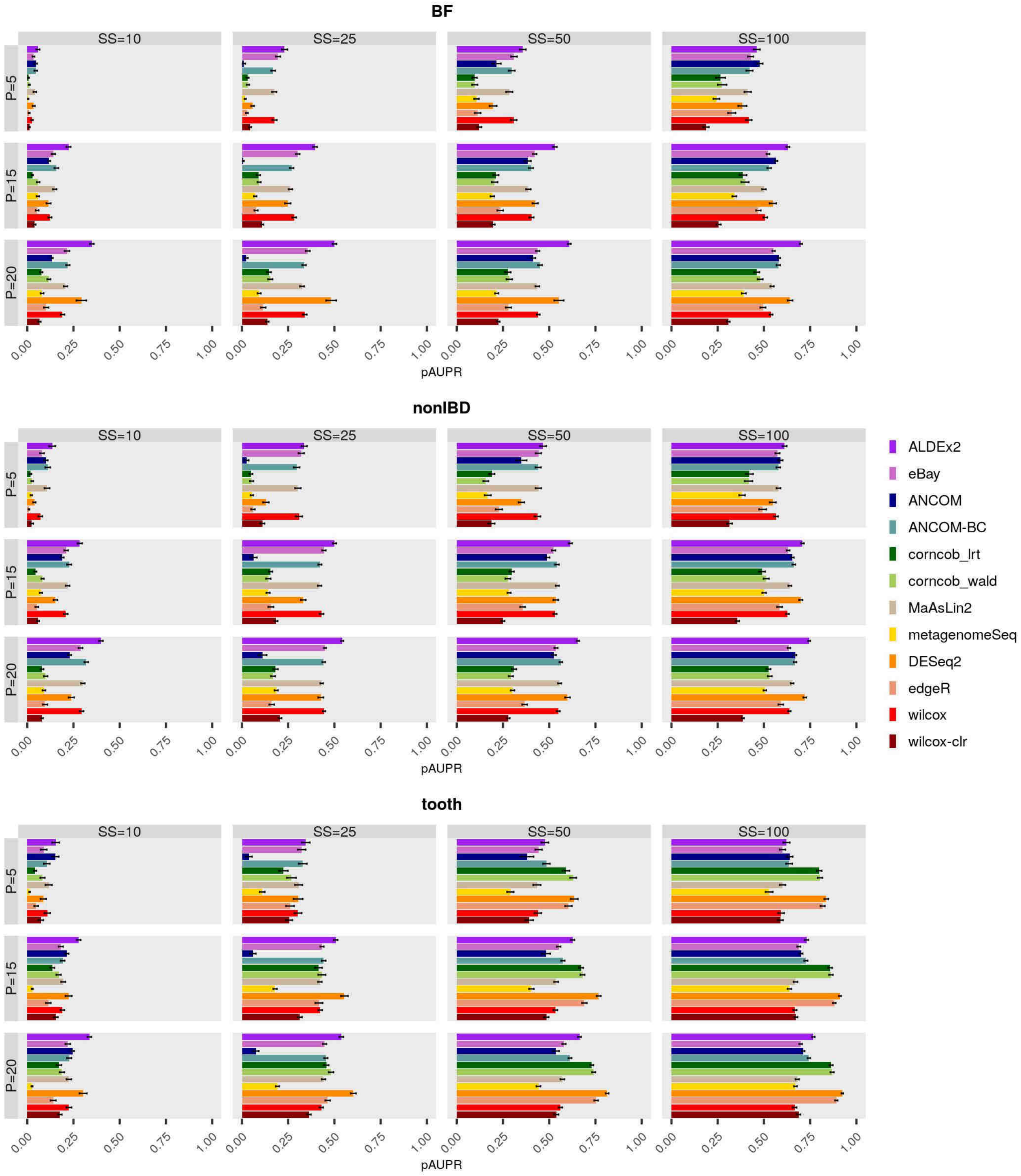

Figure 37. Partial Area Under Precision-Recall curves (pAUPR) of each differential abundance method for each dataset considered in the comparison in the scenario considering structural zeros as TN. In each set of boxes corresponding to the dataset, different percentages (P) of simulated DA features are on rows, while different sample size (SS) values are on columns. The pAUPR values are averaged over the 50 simulations and the bars show the standard error.

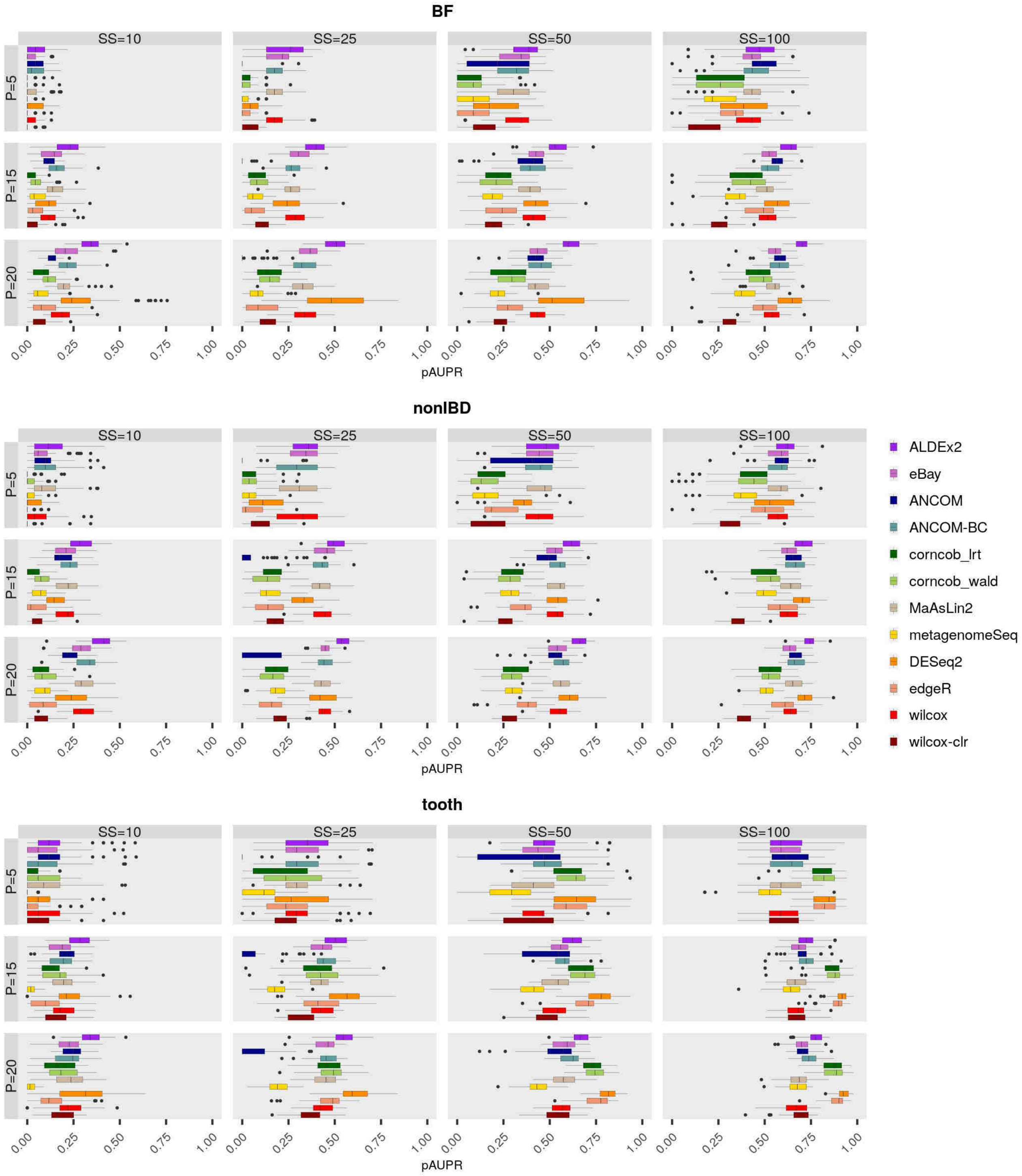

Figure 38. Boxplot of the partial Area Under Precision-Recall curves (pAUPR) of each differential abundance method for each dataset considered in the comparison in the scenario considering structural zeros as TN. In each set of boxes corresponding to the dataset, different percentages (P) of simulated DA features are on rows, while different sample size (SS) values are on columns.

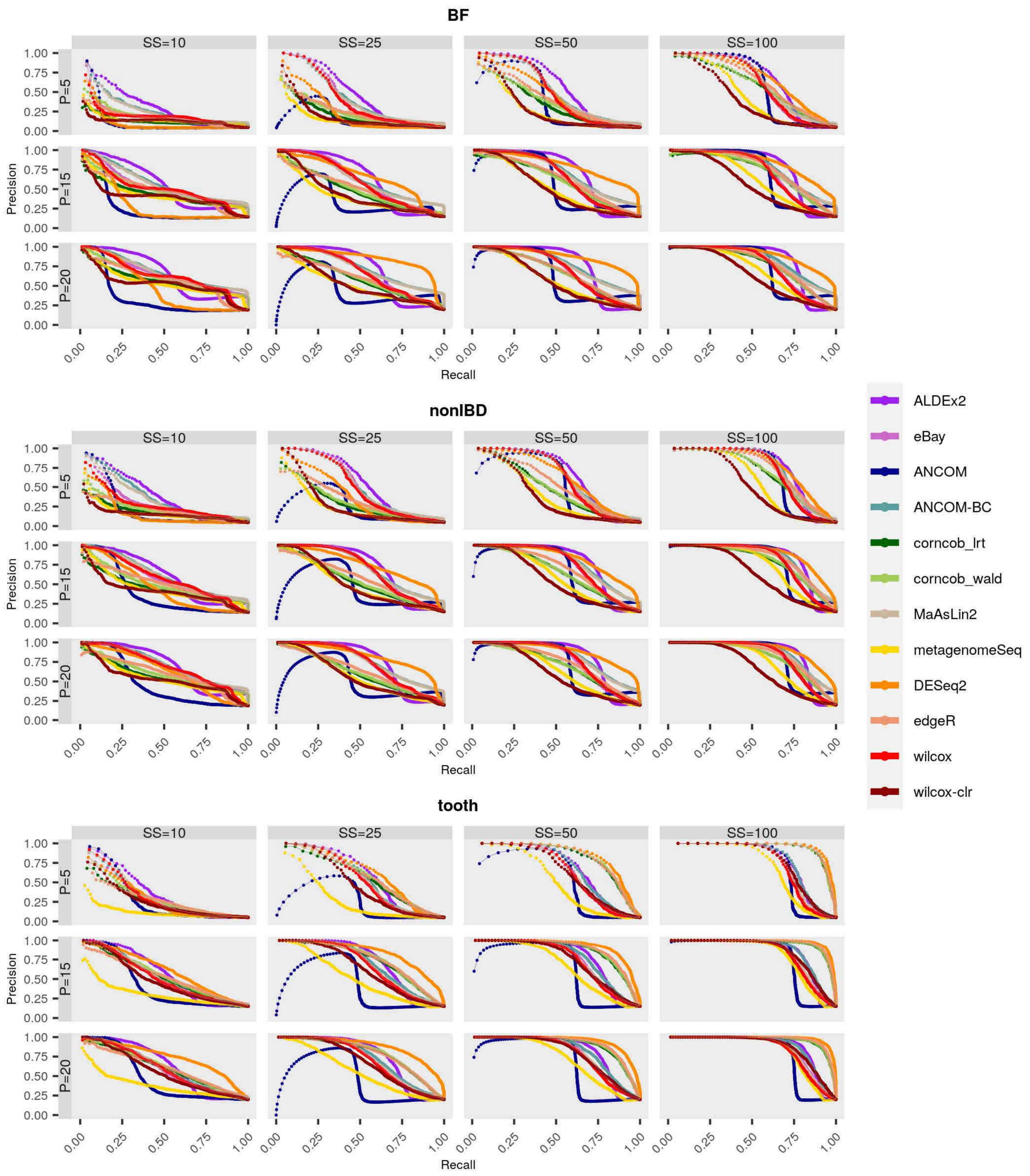

Figure 39. Mean Precision-Recall curves of each differential abundance method for each dataset considered in the comparison in the scenario considering structural zeros as TN. In each set of boxes corresponding to the dataset, different percentages (P) of simulated DA features are on rows, while different sample size (SS) values are on columns. The mean pr-curve is defined as the average of the *i*-th precision value and the *i*-th recall value over the 50 simulations.

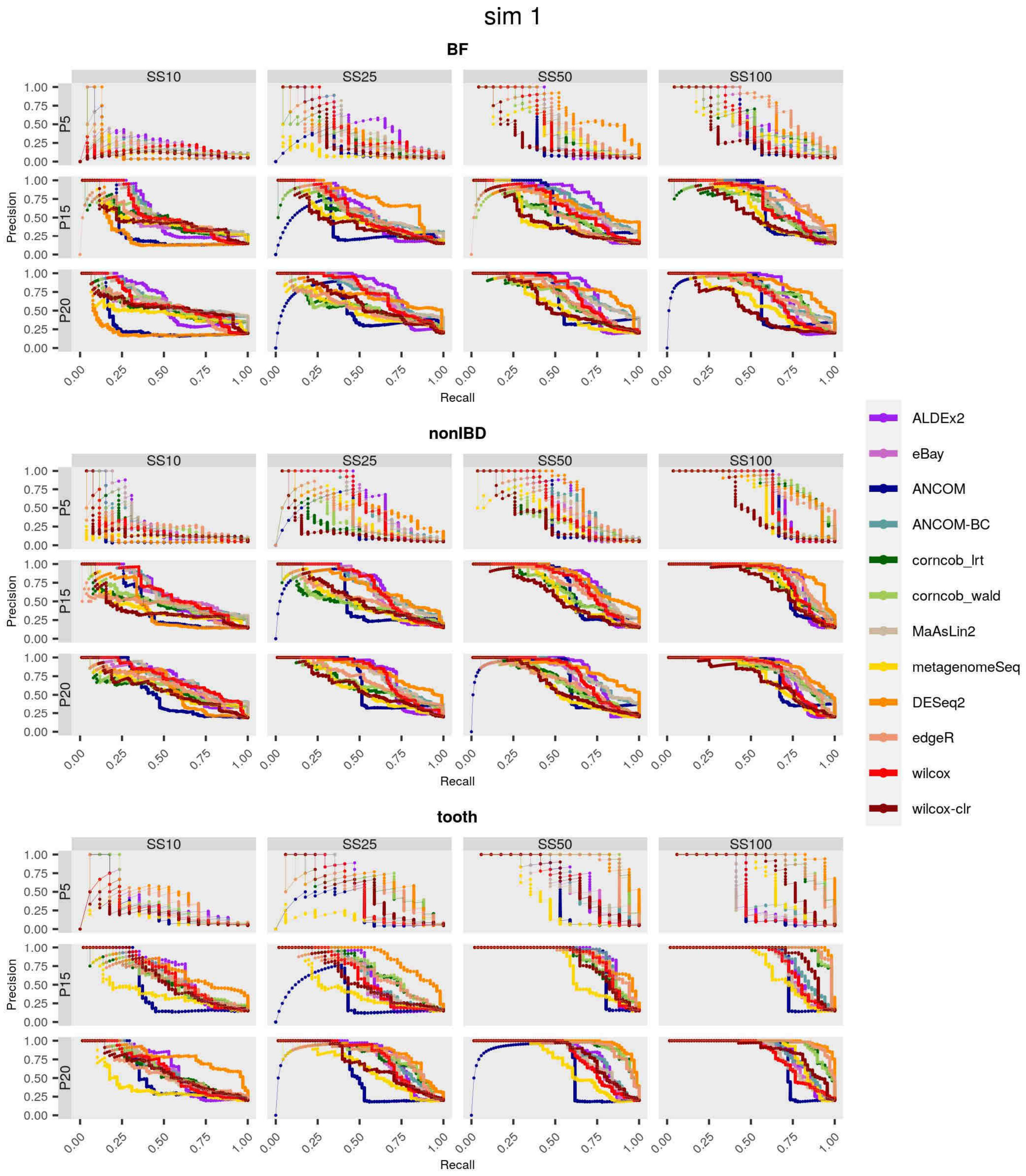

Figure 40. Simulation 1. Precision-Recall curves of each differential abundance method for each dataset considered in the comparison in the scenario considering structural zeros as TN. In each set of boxes corresponding to the dataset, different percentages (P) of simulated DA features are on rows, while different sample size (SS) values are on columns.

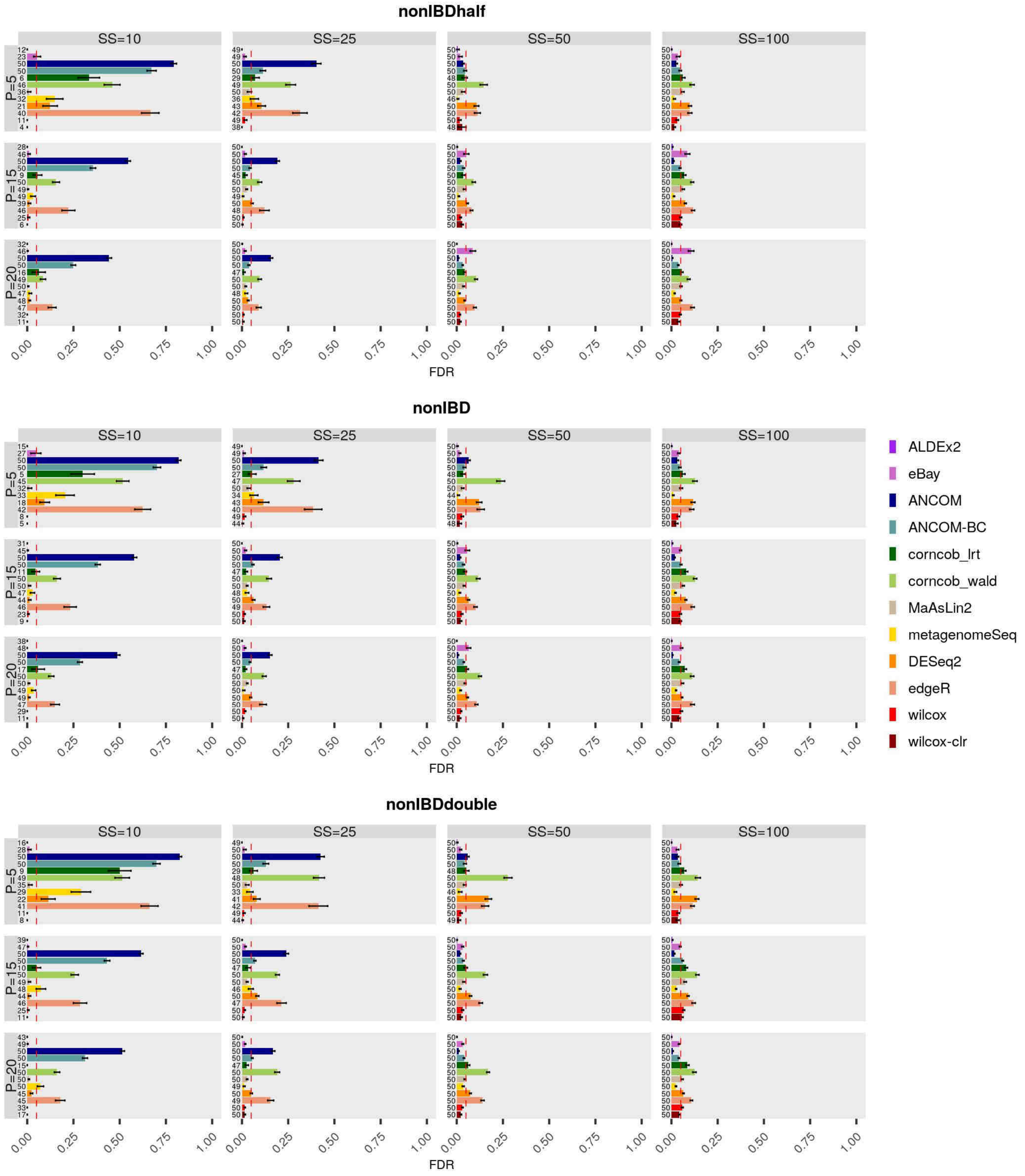

Figure 41. False Discovery Rate (FDR) of each differential abundance method for nonIBD dataset in the scenario with half, original and double sequencing depth. In each set of boxes corresponding to the dataset, different percentages (P) of simulated DA features are on rows, while different sample size (SS) values are on columns. The FDR values are averaged over the 50 simulations and the bars show the standard error. The number of runs that provide a defined value of FDR is shown at the beginning of the bars.

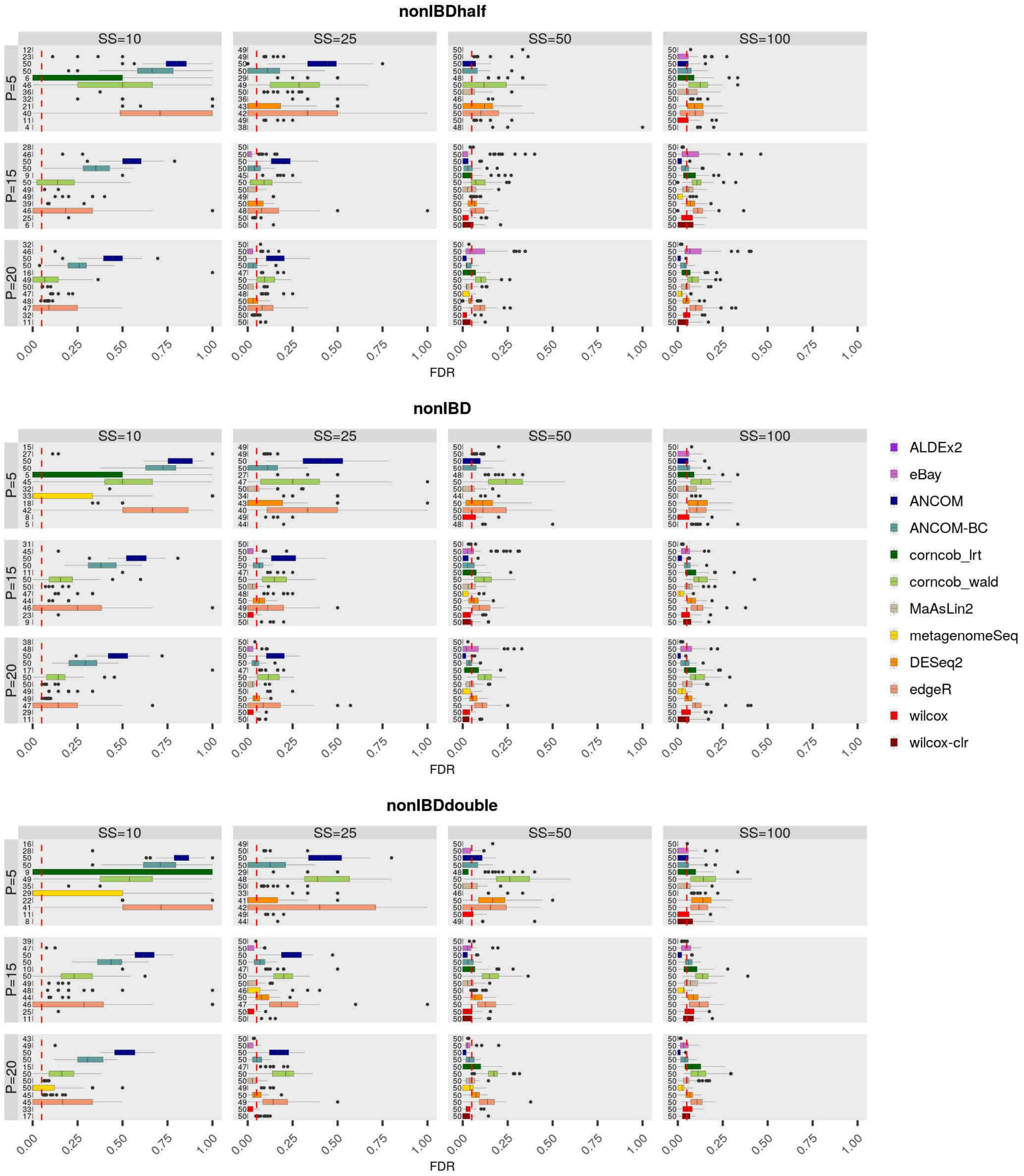

Figure 42. Boxplot of the False Discovery Rate (FDR) distribution across the 50 simulations of each differential abundance method for nonIBD dataset in the scenario with half, original and double sequencing depth. In each set of boxes corresponding to the dataset, different percentages (P) of simulated DA features are on rows, while different sample size (SS) values are on columns. The number of runs that provide a defined value of FDR is shown on the left of each boxplot.

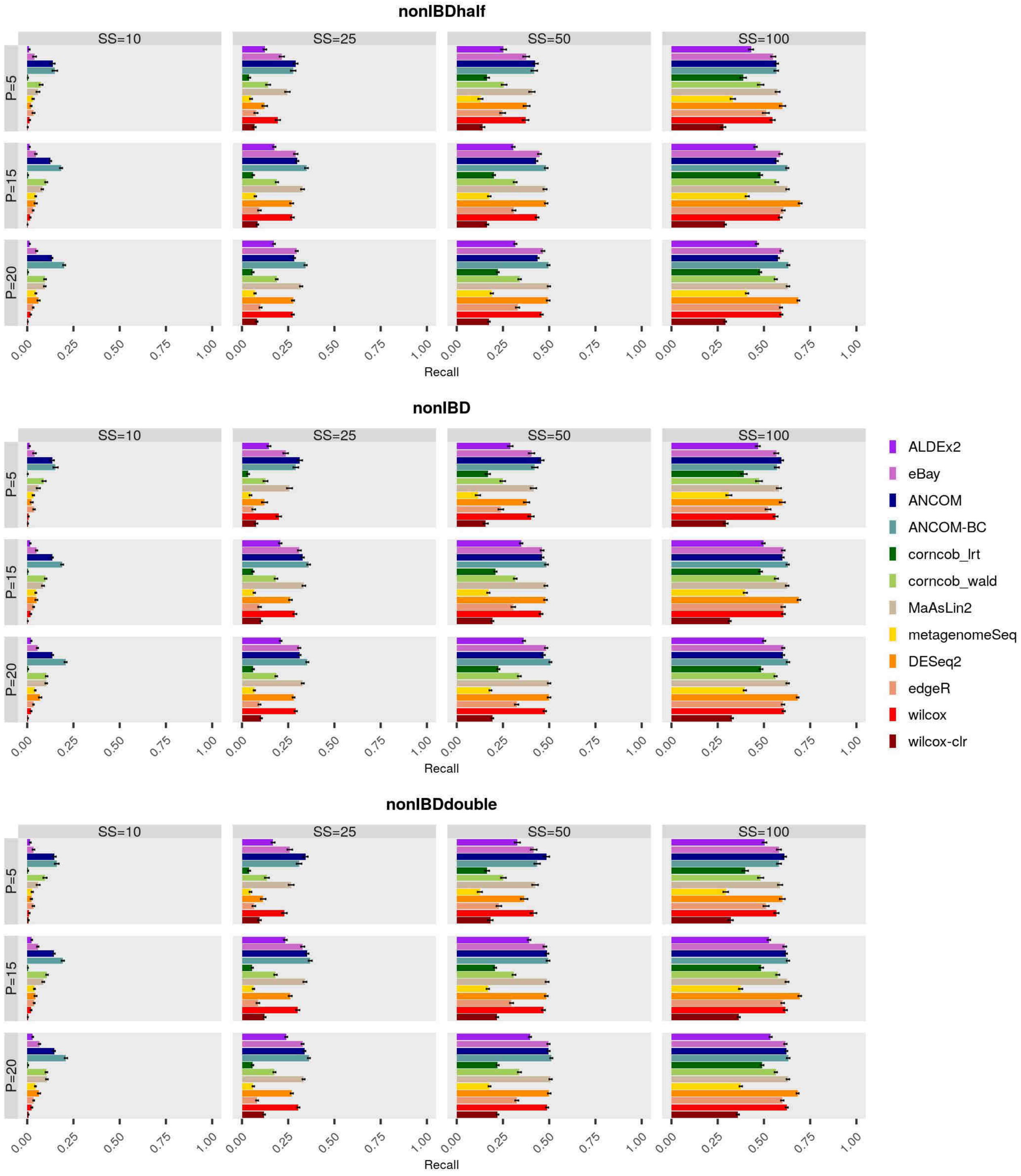

Figure 43. Recall of each differential abundance method for nonIBD dataset in the scenario with half, original and double sequencing depth. In each set of boxes corresponding to the dataset, different percentages of simulated DA features are on rows, while different sample size (SS) values are on columns. The recall values are averaged over the 50 simulations and the bars show the standard error.

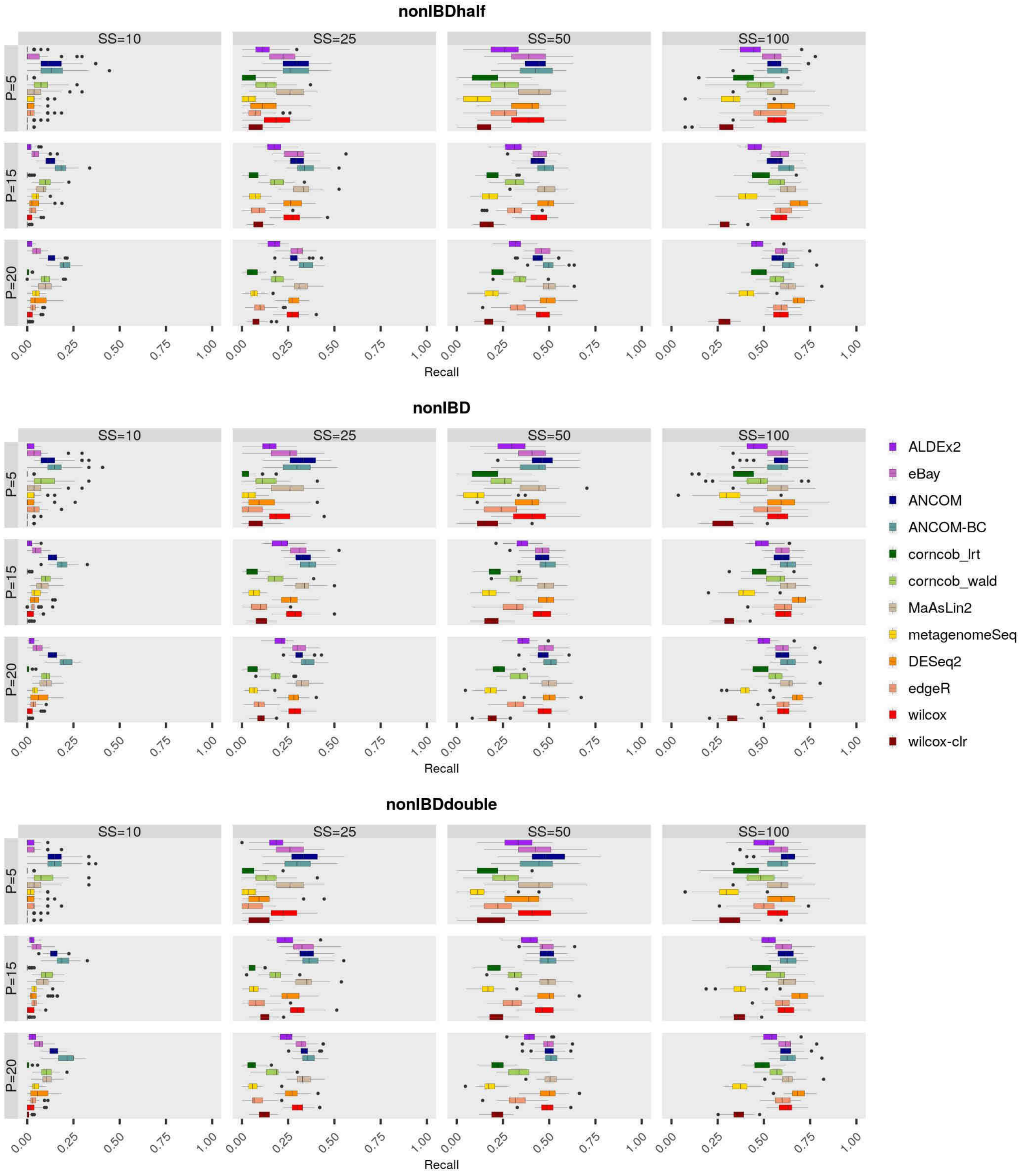

Figure 44. Boxplot of recall distribution across the 50 simulations of each differential abundance method for nonIBD dataset in the scenario with half, original and double sequencing depth. In each set of boxes corresponding to the dataset, different percentages (P) of simulated DA features are on rows, while different sample size (SS) values are on columns.

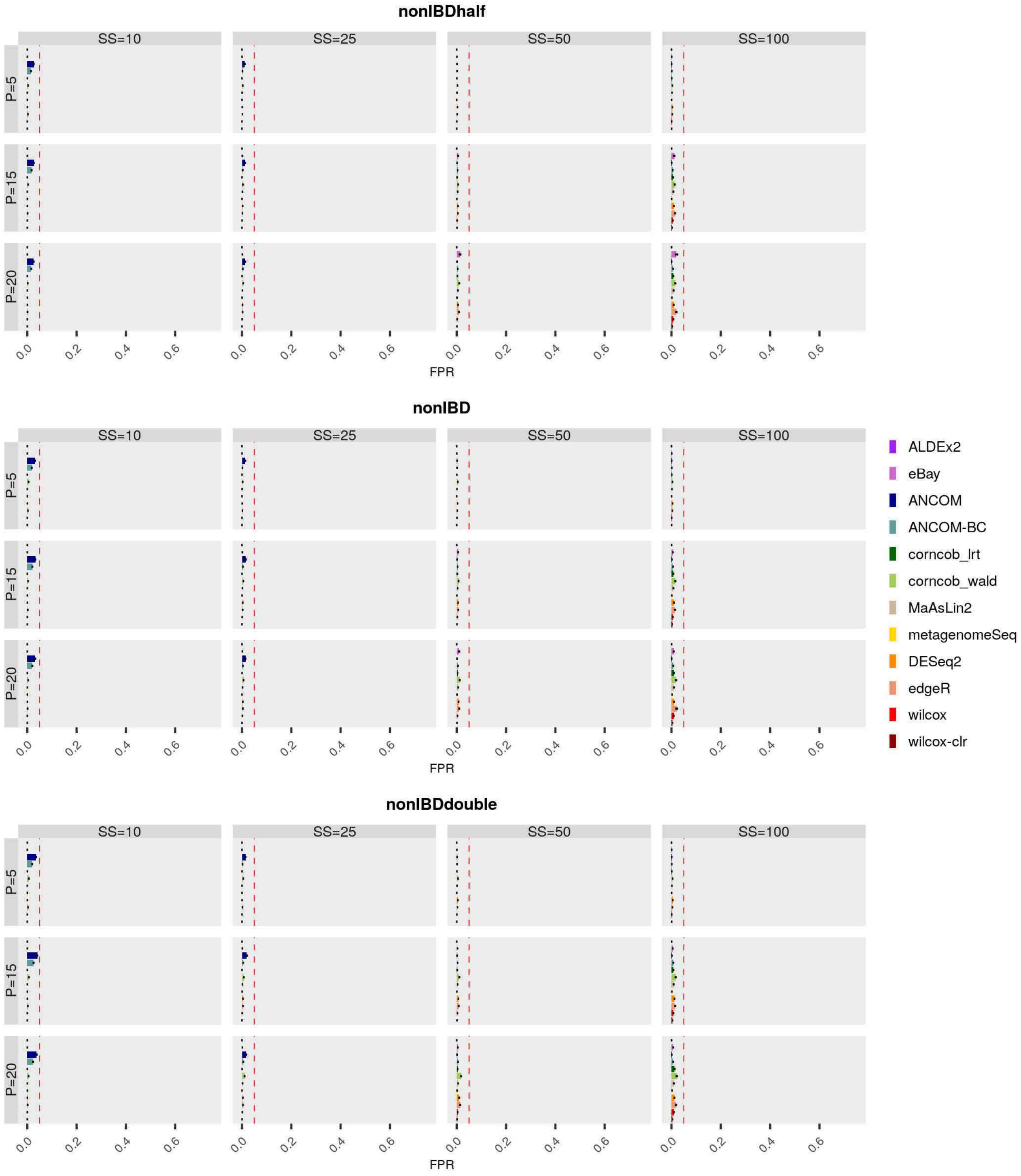

Figure 45. False Positive Rate (FPR) of each differential abundance method for nonIBD dataset in the scenario with half, original and double sequencing depth. In each set of boxes corresponding to the dataset, different percentages (P) of simulated DA features are on rows, while different sample size (SS) values are on columns. The FPR values are averaged over the 50 simulations and the bars show the standard error.

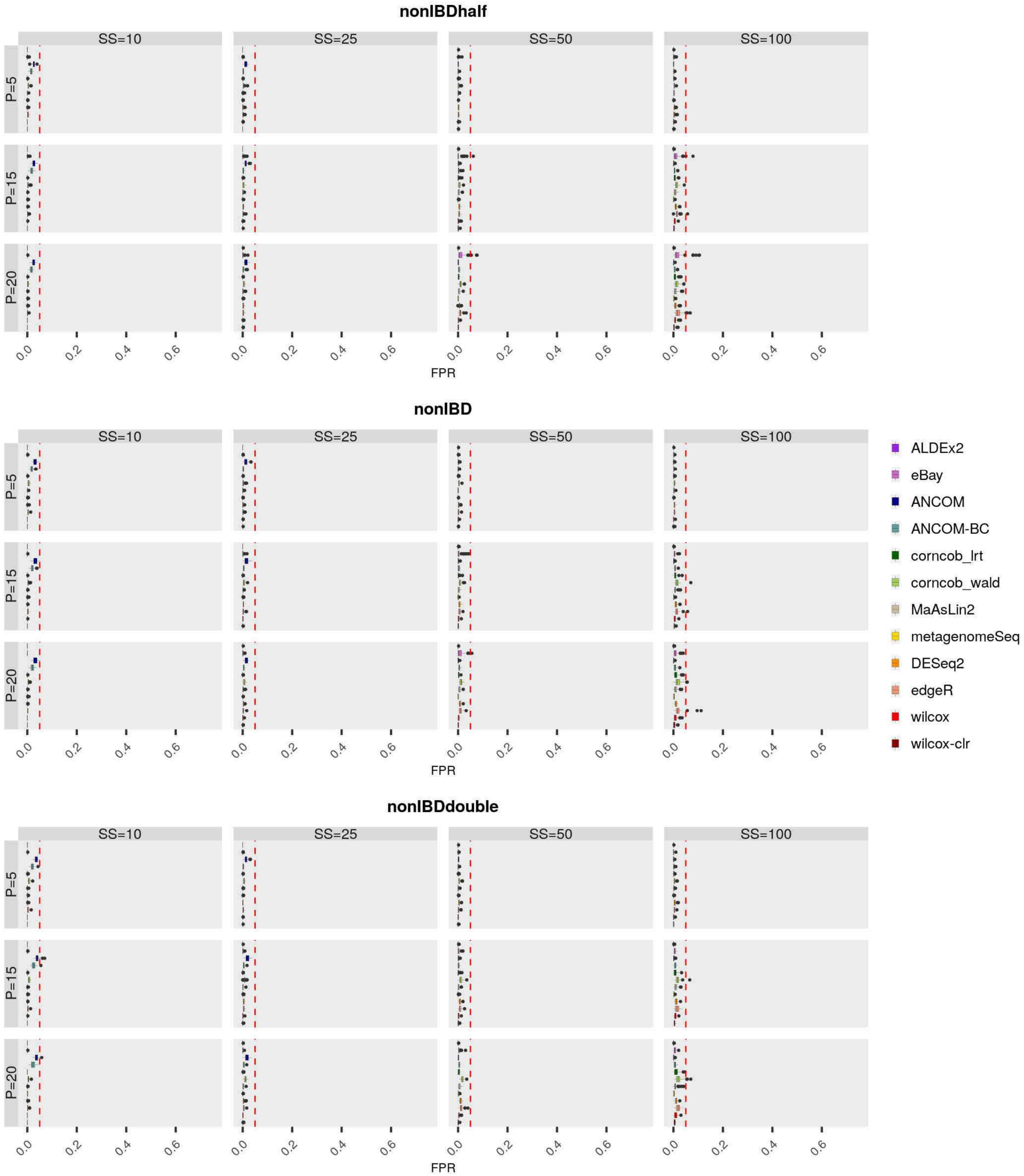

Figure 46. Boxplot of the False Positive Rate (FPR) distribution across the 50 simulations of each differential abundance method for nonIBD dataset in the scenario with half, original and double sequencing depth. In each set of boxes corresponding to the dataset, different percentages (P) of simulated DA features are on rows, while different sample size (SS) values are on columns.

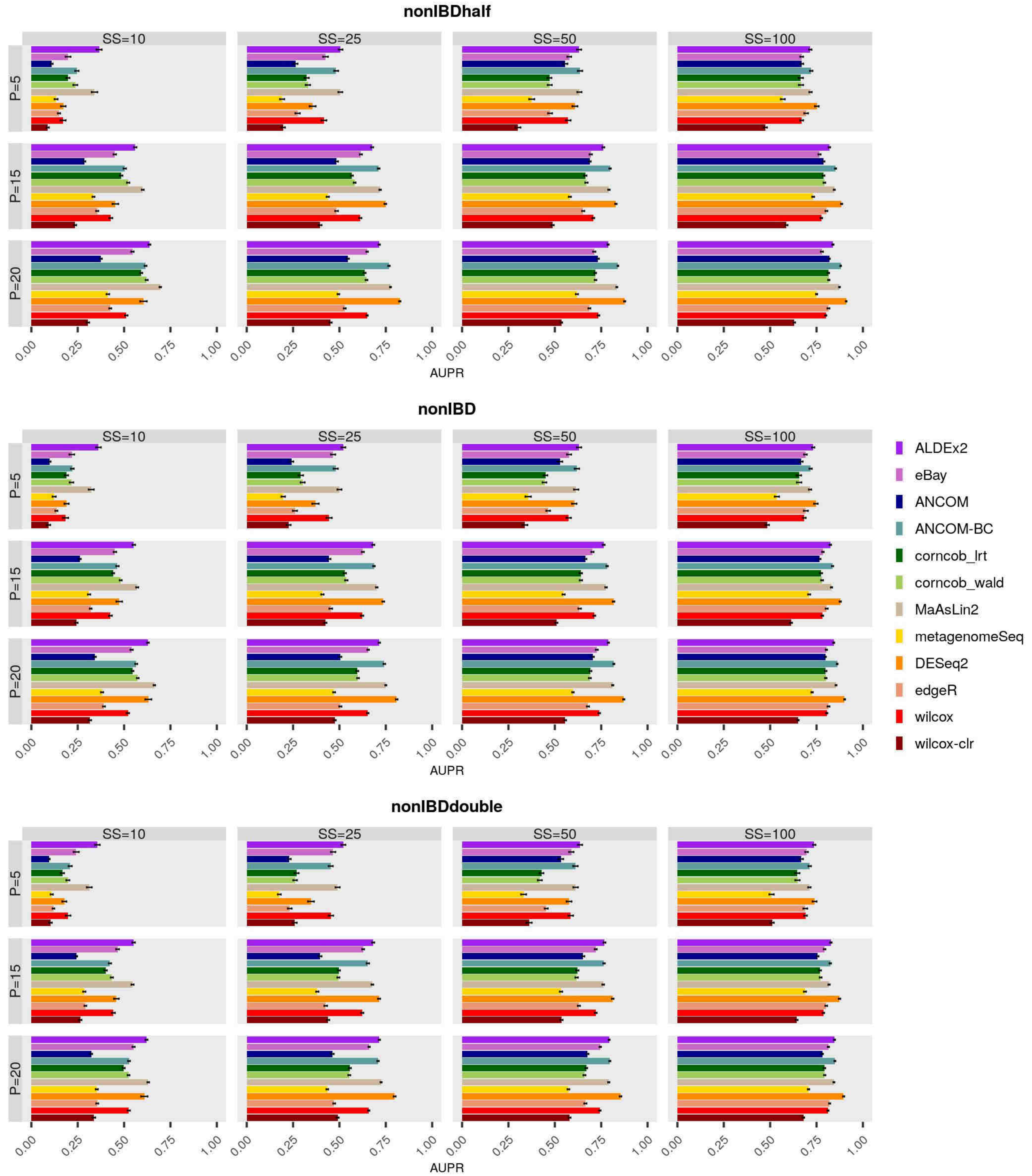

Figure 47. Area Under Precision-Recall curves (AUPR) of each differential abundance method for nonIBD dataset in the scenario with half, original and double sequencing depth. In each set of boxes corresponding to the dataset, different percentages (P) of simulated DA features are on rows, while different sample size (SS) values are on columns. The AUPR values are averaged over the 50 simulations and the bars show the standard error.

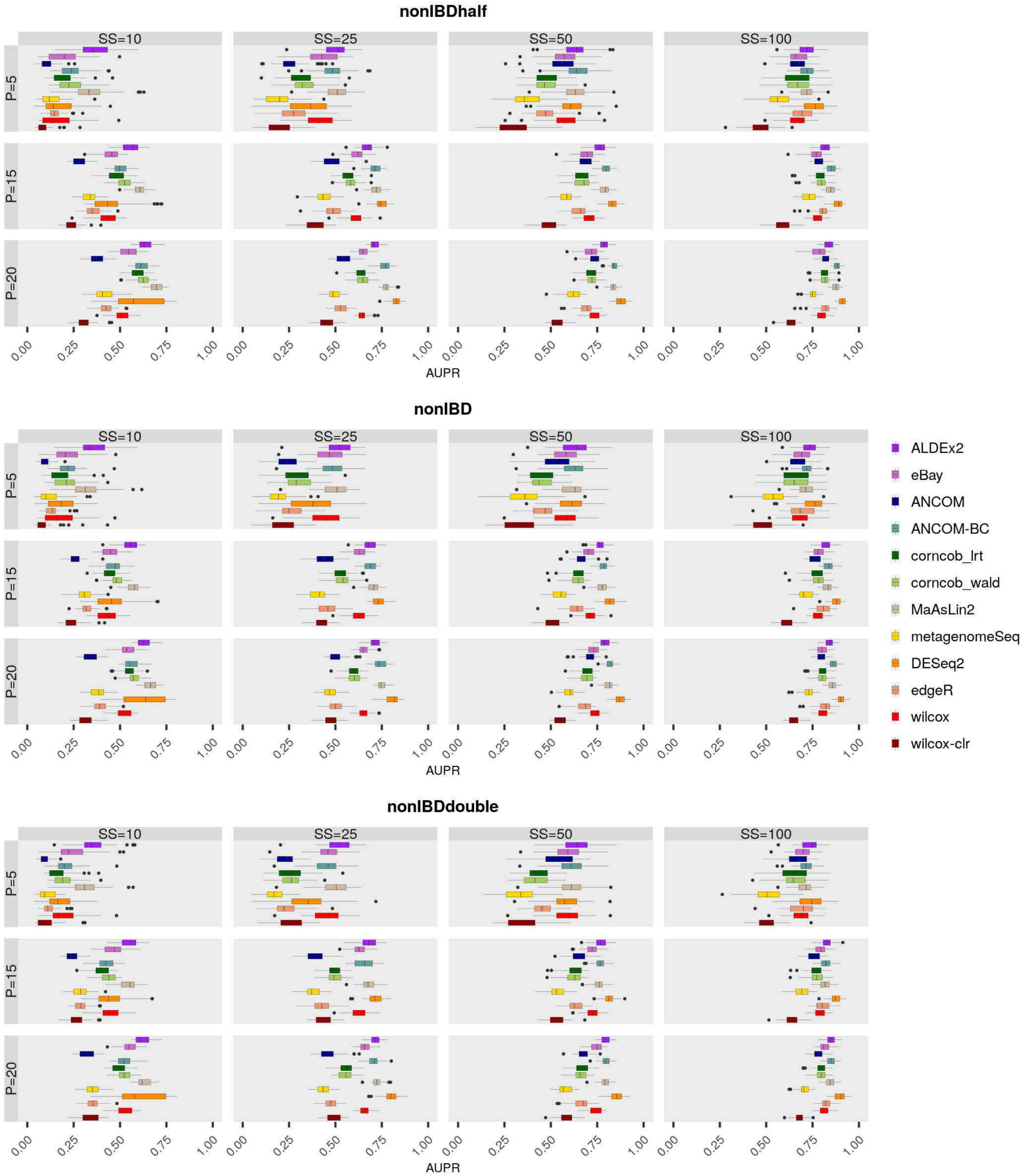

Figure 48. Boxplot of the Area Under Precision-Recall curves (AUPR) of each differential abundance method for nonIBD dataset in the scenario with half, original and double sequencing depth. In each set of boxes corresponding to the dataset, different percentages (P) of simulated DA features are on rows, while different sample size (SS) values are on columns.

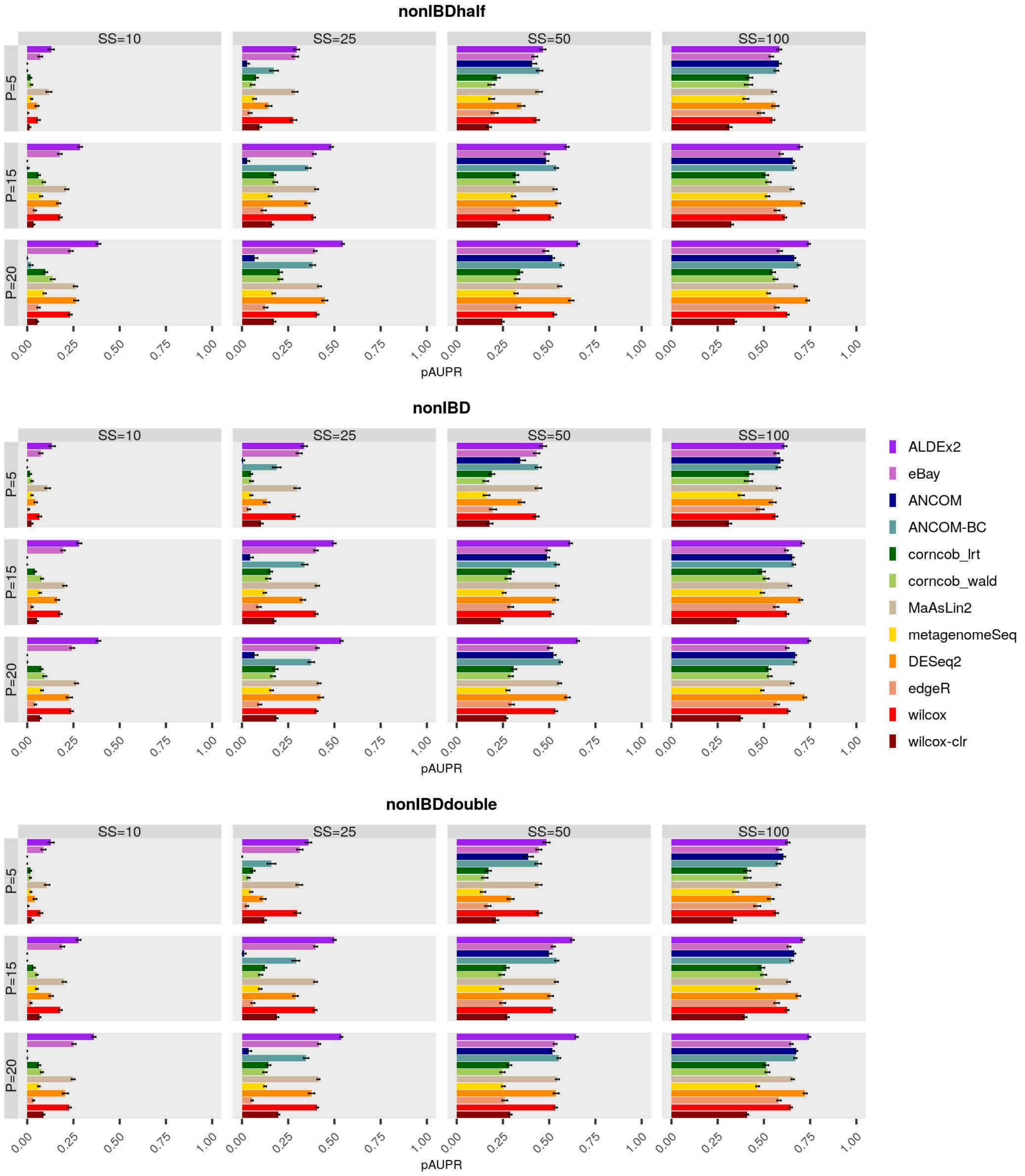

Figure 49. Partial Area Under Precision-Recall curves (pAUPR) of each differential abundance method for nonIBD dataset in the scenario with half, original and double sequencing depth. In each set of boxes corresponding to the dataset, different percentages (P) of simulated DA features are on rows, while different sample size (SS) values are on columns. The pAUPR values are averaged over the 50 simulations and the bars show the standard error.

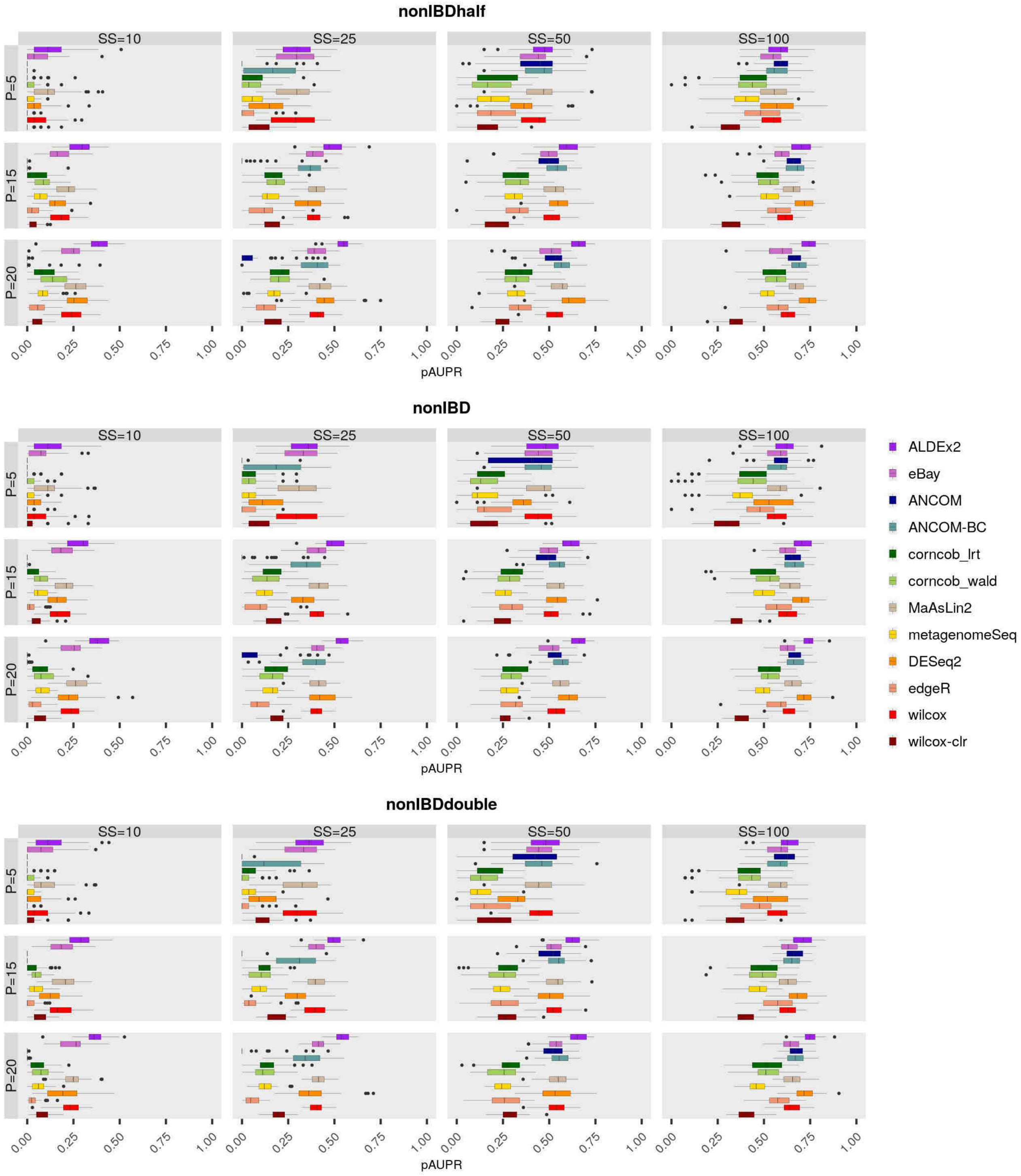

Figure 50. Boxplot of the partial Area Under Precision-Recall curves (pAUPR) of each differential abundance method for nonIBD dataset in the scenario with half, original and double sequencing depth. In each set of boxes corresponding to the dataset, different percentages (P) of simulated DA features are on rows, while different sample size (SS) values are on columns.

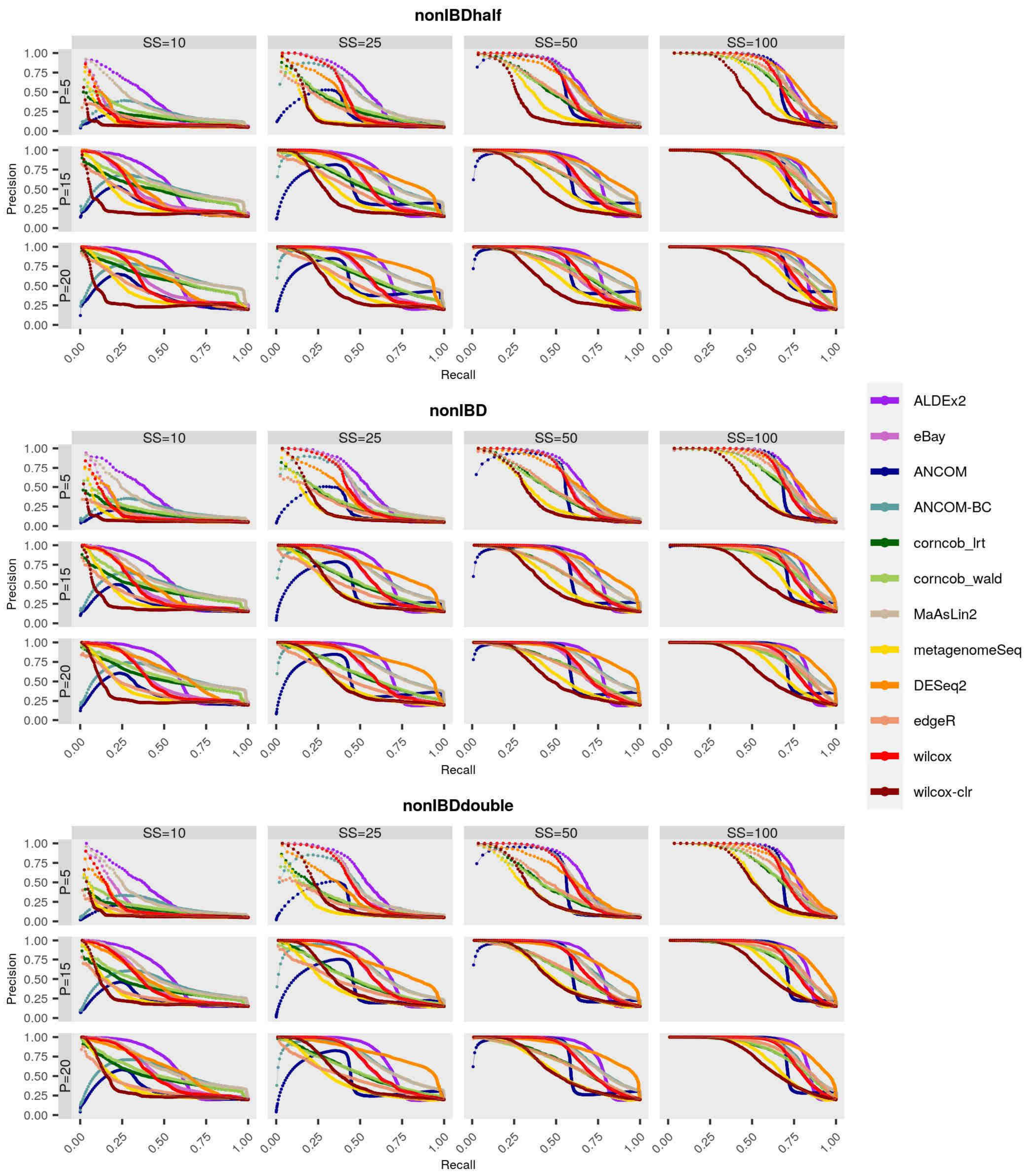

Figure 51. Mean Precision-Recall curves of each differential abundance method for nonIBD dataset in the scenario with half, original and double sequencing depth. In each set of boxes corresponding to the dataset, different percentages (P) of simulated DA features are on rows, while different sample size (SS) values are on columns. The mean pr-curve is defined as the average of the *i*-th precision value and the *i*-th recall value over the 50 simulations.

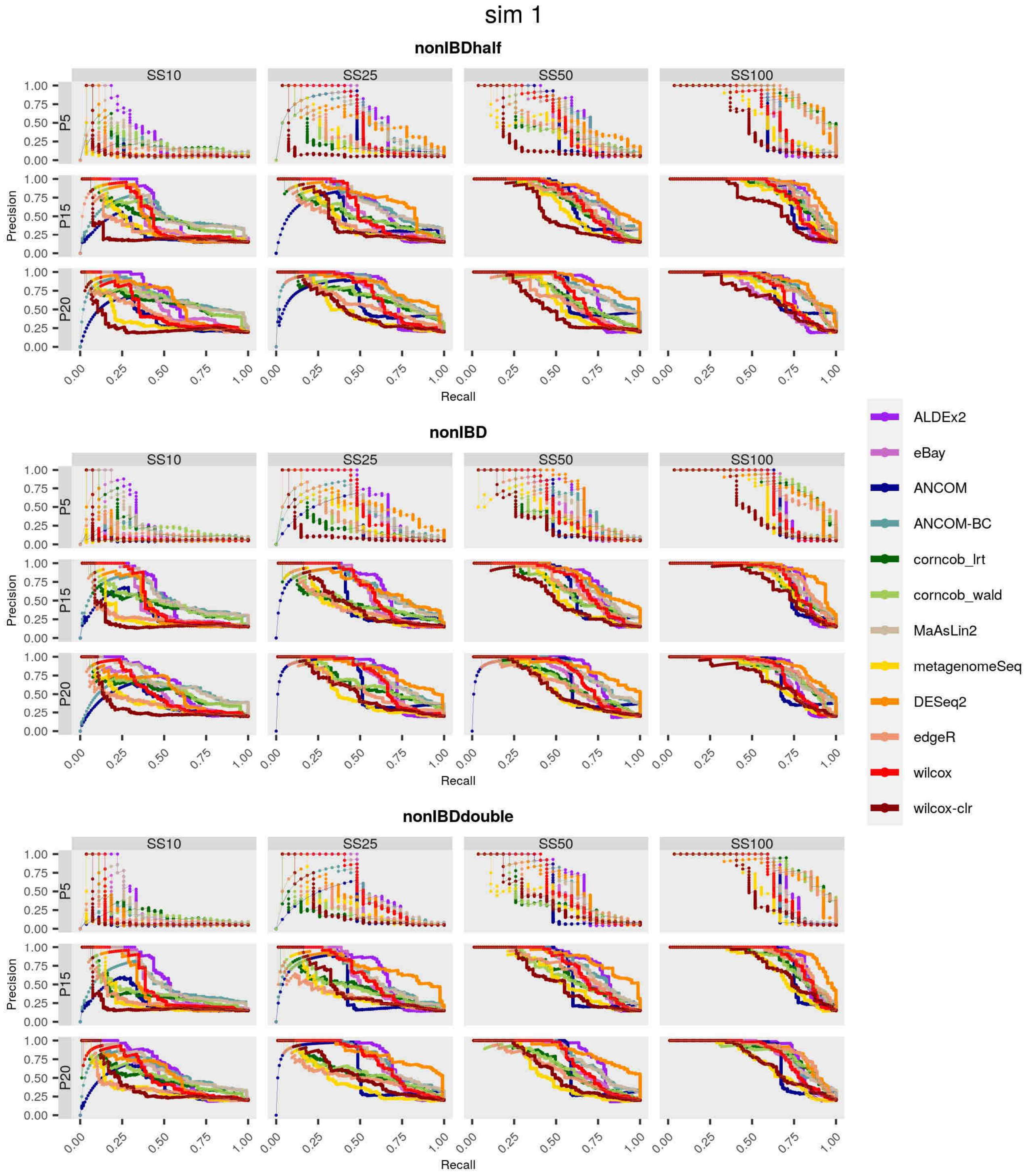

Figure 52. Simulation 1. Precision-Recall curves of each differential abundance method for nonIBD dataset in the scenario with half, original and double sequencing depth. In each set of boxes corresponding to the dataset, different percentages (P) of simulated DA features are on rows, while different sample size (SS) values are on columns.

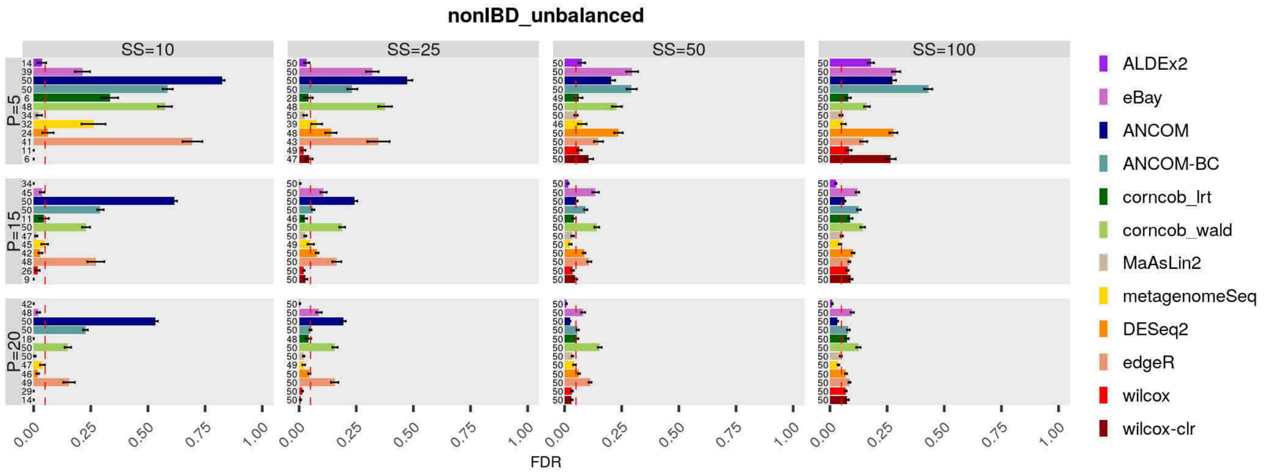

Figure 53. False Discovery Rate (FDR) of each differential abundance method for nonIBD dataset in the scenario with unbalanced sequencing depth between conditions. Different percentages (P) of simulated DA features are on rows, while different sample size (SS) values are on columns. The FDR values are averaged over the 50 simulations and the bars show the standard error. The number of runs that provide a defined value of FDR is shown at the beginning of the bars.

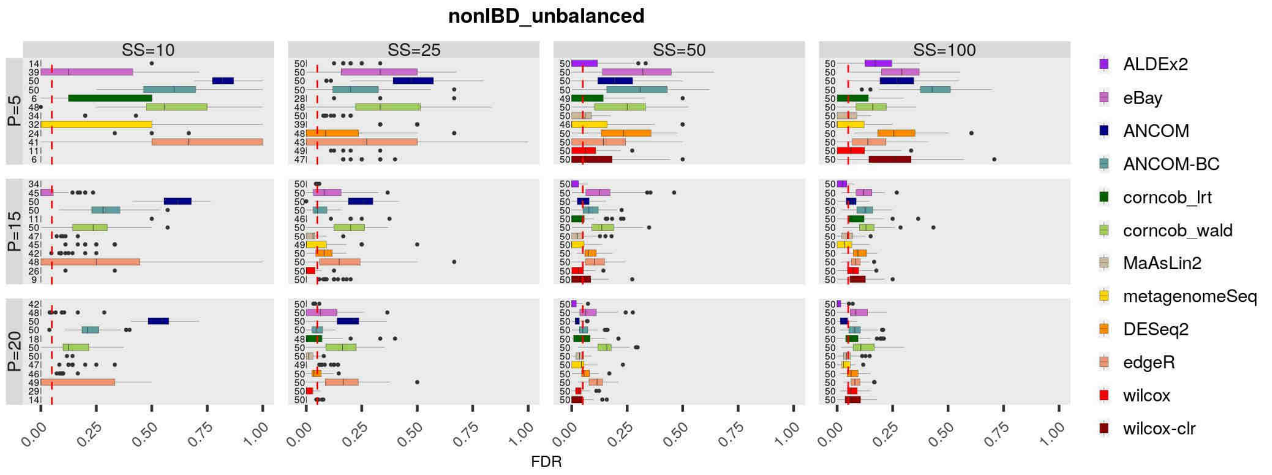

Figure 54. Boxplot of the False Discovery Rate (FDR) distribution across the 50 simulations of each differential abundance method for nonIBD dataset in the scenario with unbalanced sequencing depth between conditions. Different percentages (P) of simulated DA features are on rows, while different sample size (SS) values are on columns. The number of runs that provide a defined value of FDR is shown on the left of each boxplot.

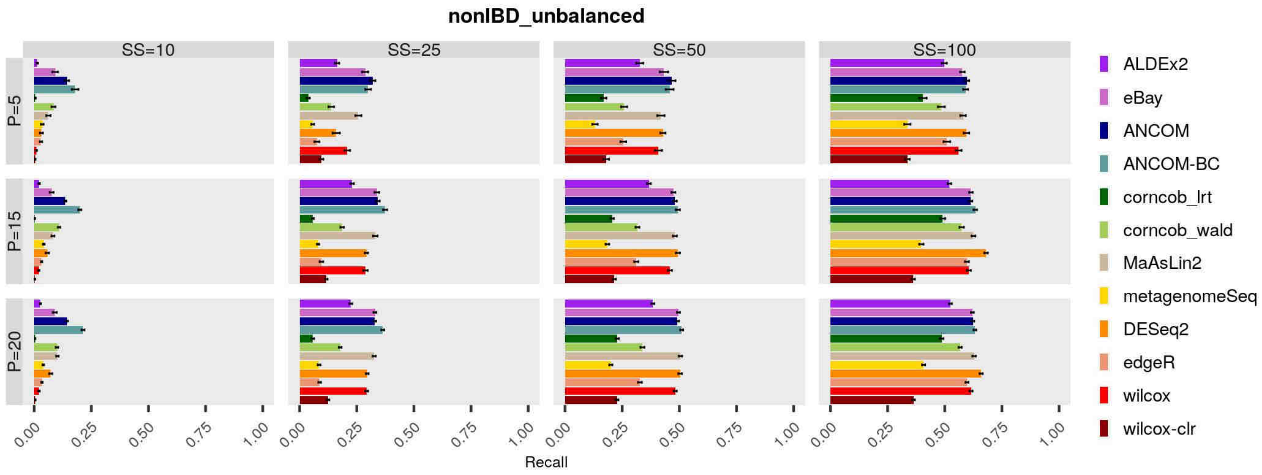

Figure 55. Recall of each differential abundance method for nonIBD dataset in the scenario with unbalanced sequencing depth between conditions. Different percentages (P) of simulated DA features are on rows, while different sample size (SS) values are on columns. The recall values are averaged over the 50 simulations and the bars show the standard error.

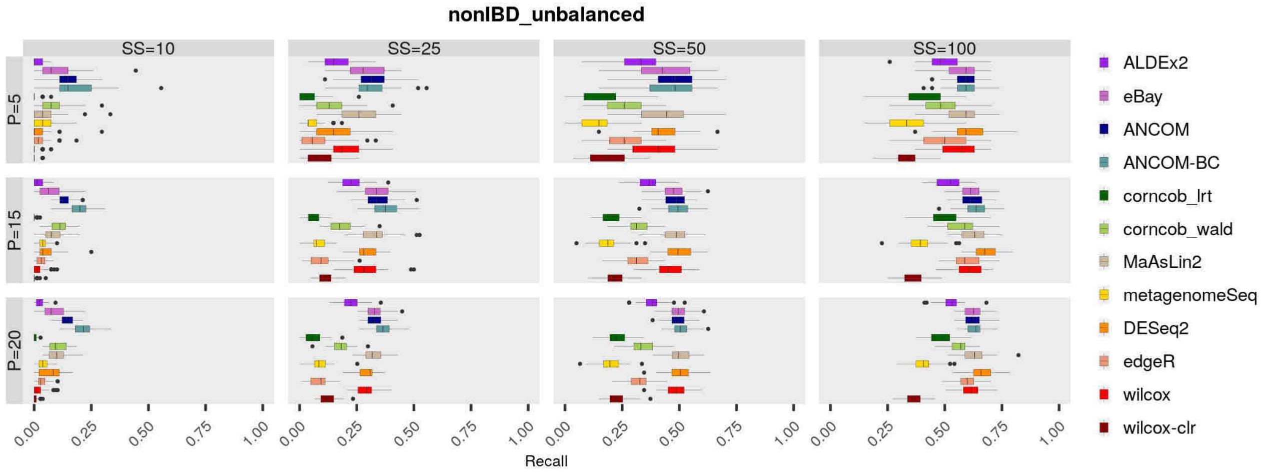

Figure 56. Boxplot of recall distribution across the 50 simulations of each differential abundance method for nonIBD dataset in the scenario with unbalanced sequencing depth between conditions. Different percentages (P) of simulated DA features are on rows, while different sample size (SS) values are on columns.

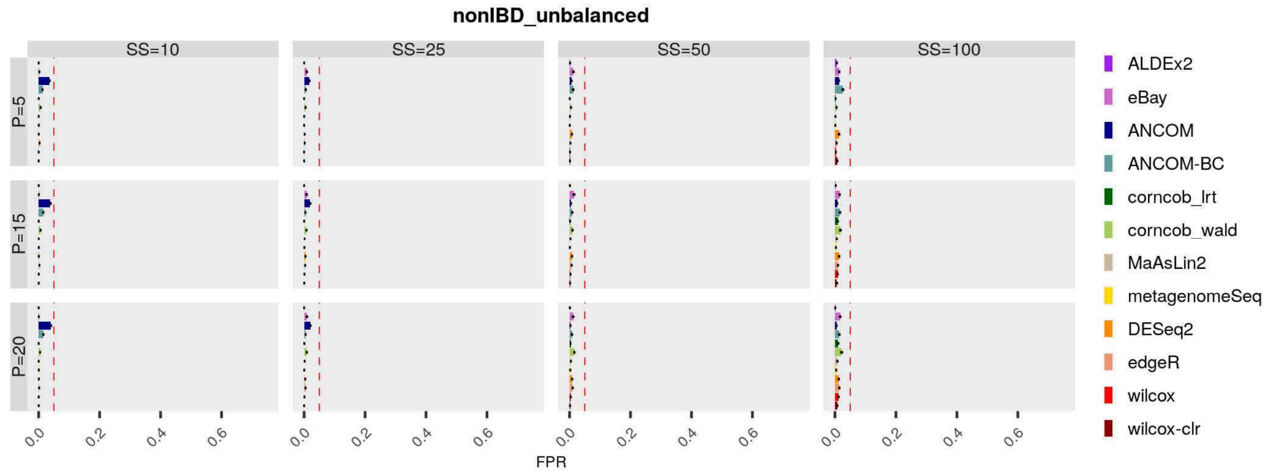

Figure 57. False Positive Rate (FPR) of each differential abundance method for nonIBD dataset in the scenario with unbalanced sequencing depth between conditions. Different percentages (P) of simulated DA features are on rows, while different sample size (SS) values are on columns. The FPR values are averaged over the 50 simulations and the bars show the standard error.

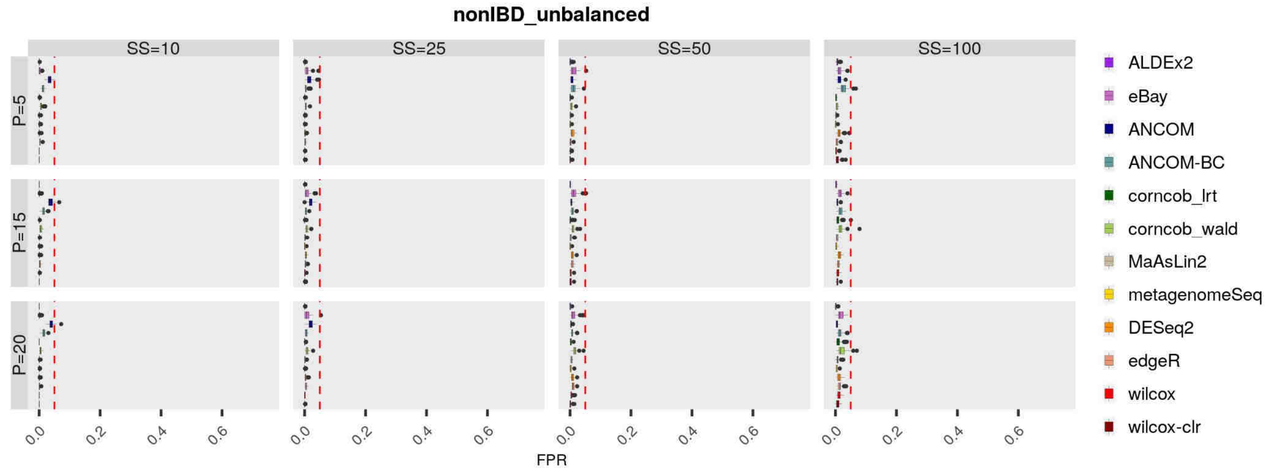

Figure 58. Boxplot of the False Positive Rate (FPR) distribution across the 50 simulations of each differential abundance method for nonIBD dataset in the scenario with unbalanced sequencing depth between conditions. Different percentages (P) of simulated DA features are on rows, while different sample size (SS) values are on columns.

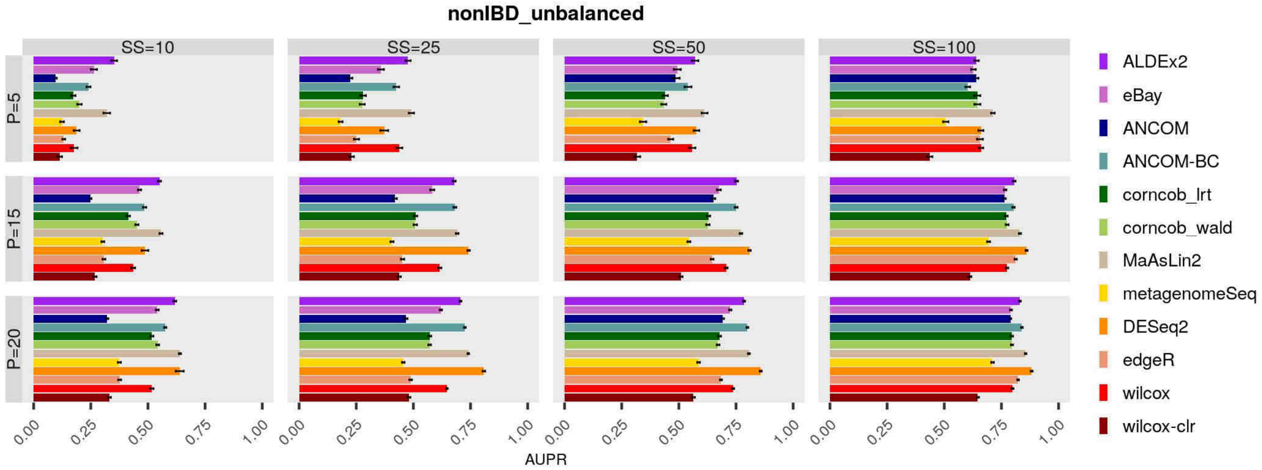

Figure 59. Area Under Precision-Recall curves (AUPR) of each differential abundance method for nonIBD dataset in the scenario with unbalanced sequencing depth between conditions. Different percentages (P) of simulated DA features are on rows, while different sample size (SS) values are on columns. The AUPR values are averaged over the 50 simulations and the bars show the standard error.

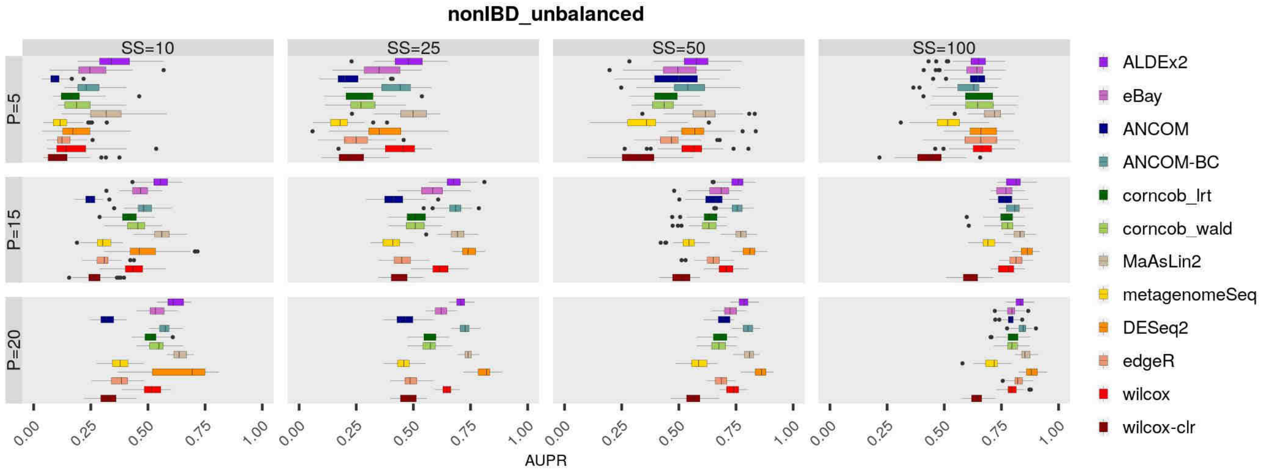

Figure 60. Boxplot of the Area Under Precision-Recall curves (AUPR) of each differential abundance method for nonIBD dataset in the scenario with unbalanced sequencing depth between conditions. Different percentages (P) of simulated DA features are on rows, while different sample size (SS) values are on columns.

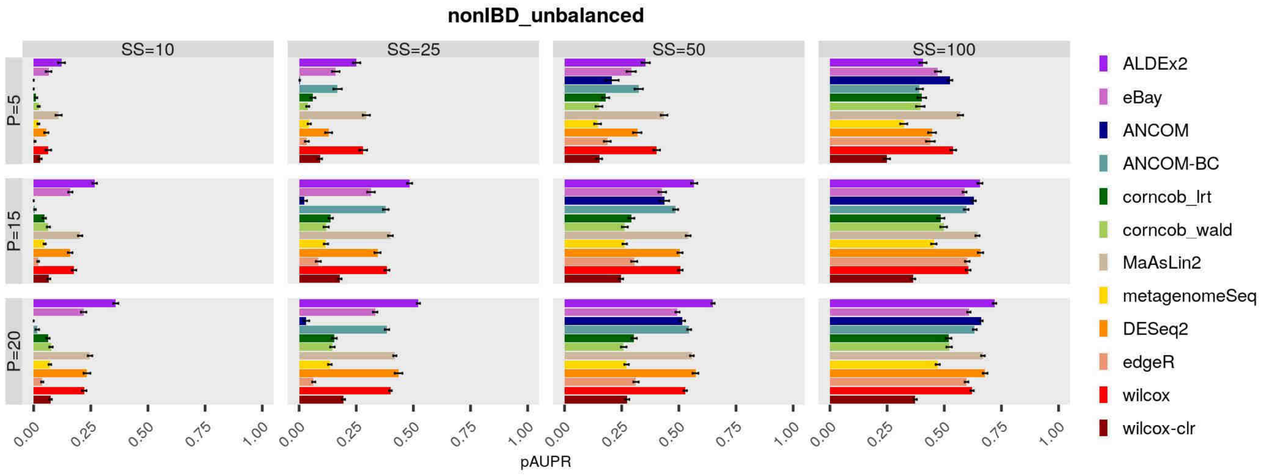

Figure 61. Partial Area Under Precision-Recall curves (pAUPR) of each differential abundance method for nonIBD dataset in the scenario with unbalanced sequencing depth between conditions. Different percentages (P) of simulated DA features are on rows, while different sample size (SS) values are on columns. The pAUPR values are averaged over the 50 simulations and the bars show the standard error.

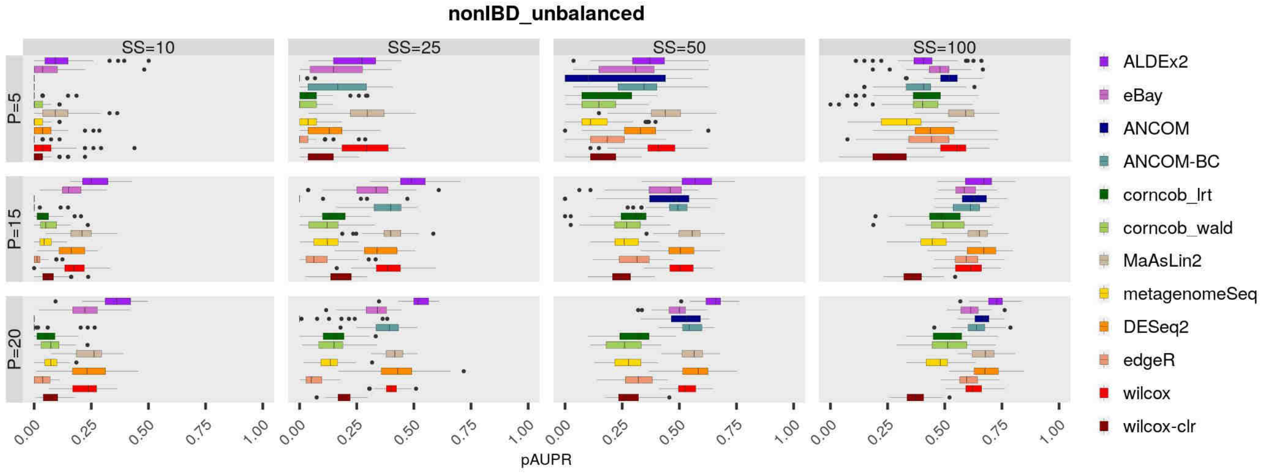

Figure 62. Boxplot of the partial Area Under Precision-Recall curves (pAUPR) of each differential abundance method for nonIBD dataset in the scenario with unbalanced sequencing depth between conditions. Different percentages (P) of simulated DA features are on rows, while different sample size (SS) values are on columns.

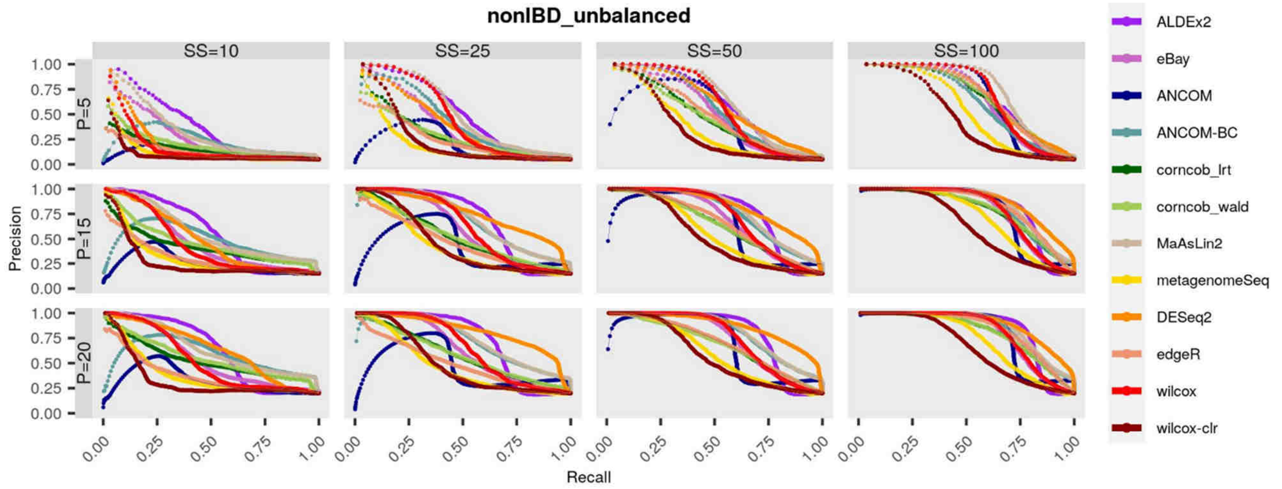

Figure 63. Mean Precision-Recall curves of each differential abundance method for nonIBD dataset in the scenario with unbalanced sequencing depth between conditions. Different percentages (P) of simulated DA features are on rows, while different sample size (SS) values are on columns. The mean pr-curve is defined as the average of the *i*-th precision value and the *i*-th recall value over the 50 simulations.

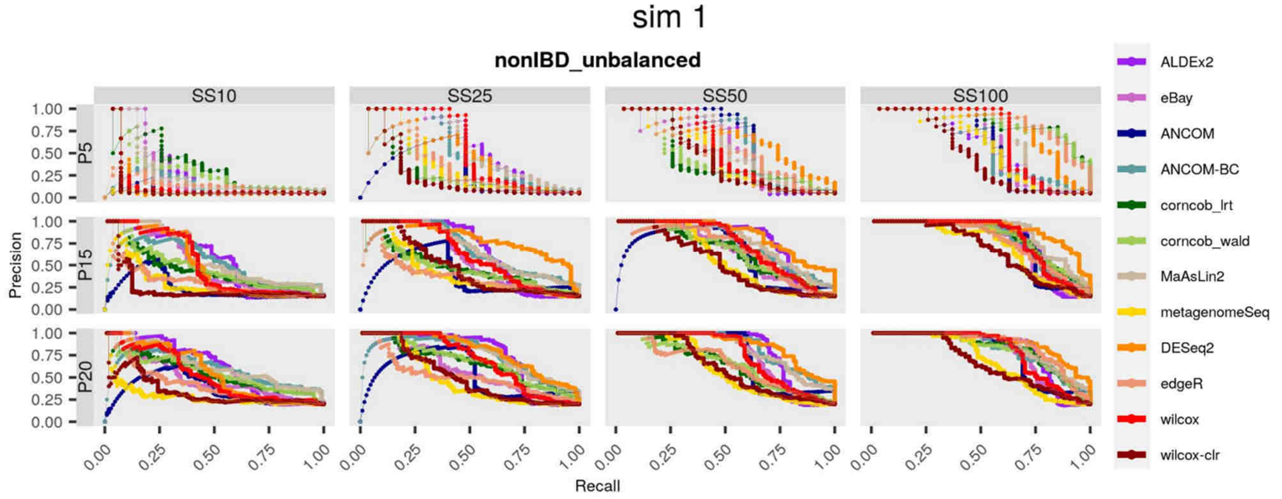

Figure 64. Simulation 1. Precision-Recall curves of each differential abundance method for nonIBD dataset in the scenario with unbalanced sequencing depth between conditions. Different percentages (P) of simulated DA features are on rows, while different sample size (SS) values are on columns.

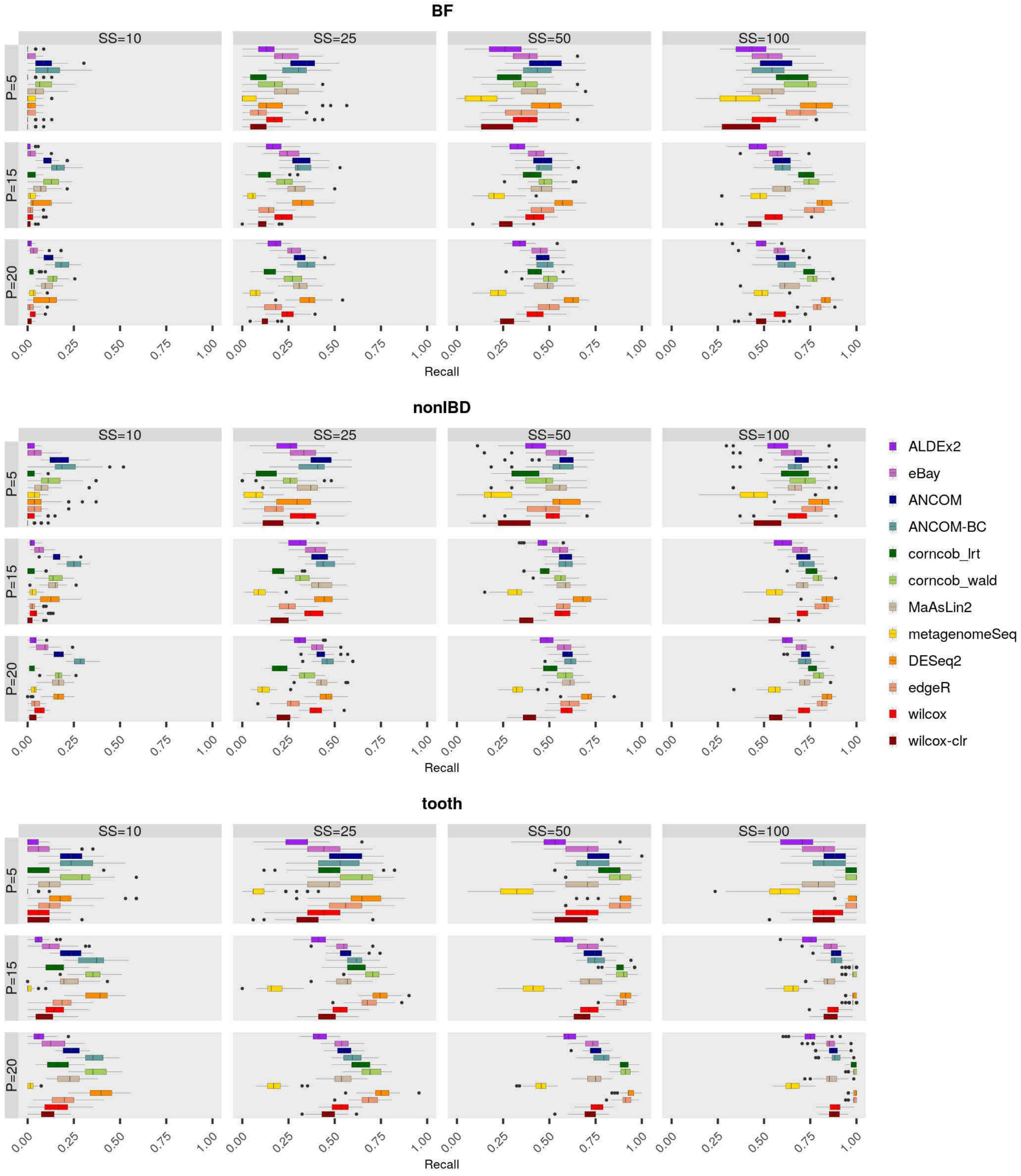

Figure 65. Boxplot of recall distribution across the 50 simulations of each differential abundance method for each dataset considered in the comparison in the scenario considering half variability in simulation. In each set of boxes corresponding to the dataset, different percentages (P) of simulated DA features are on rows, while different sample size (SS) values are on columns.

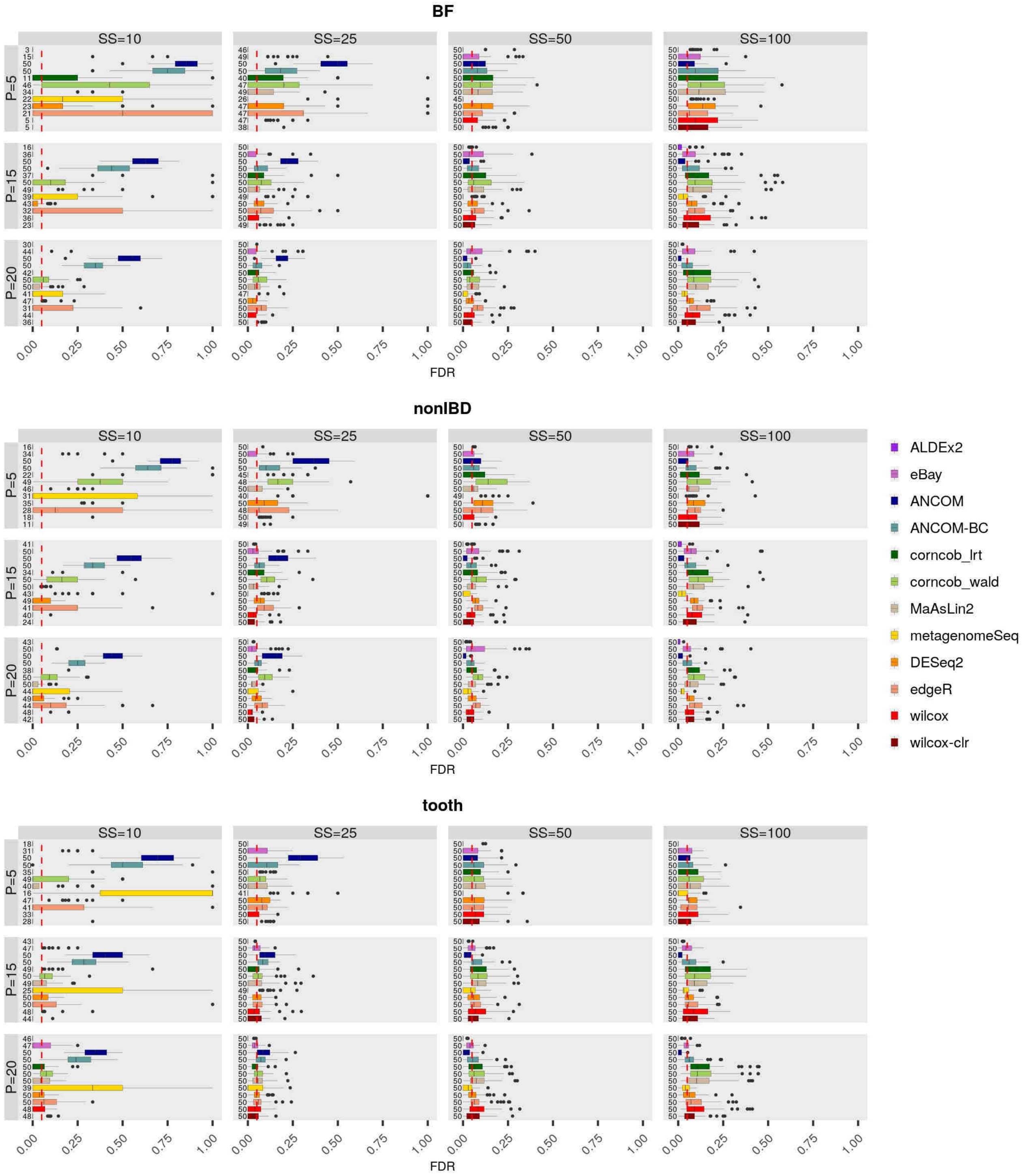

Figure 66. Boxplot of the False Discovery Rate (FDR) distribution across the 50 simulations of each differential abundance method for each dataset considered in the comparison in the scenario considering half variability in simulation. In each set of boxes corresponding to the dataset, different percentages (P) of simulated DA features are on rows, while different sample size (SS) values are on columns. The number of runs that provide a defined value of FDR is shown on the left of each boxplot.

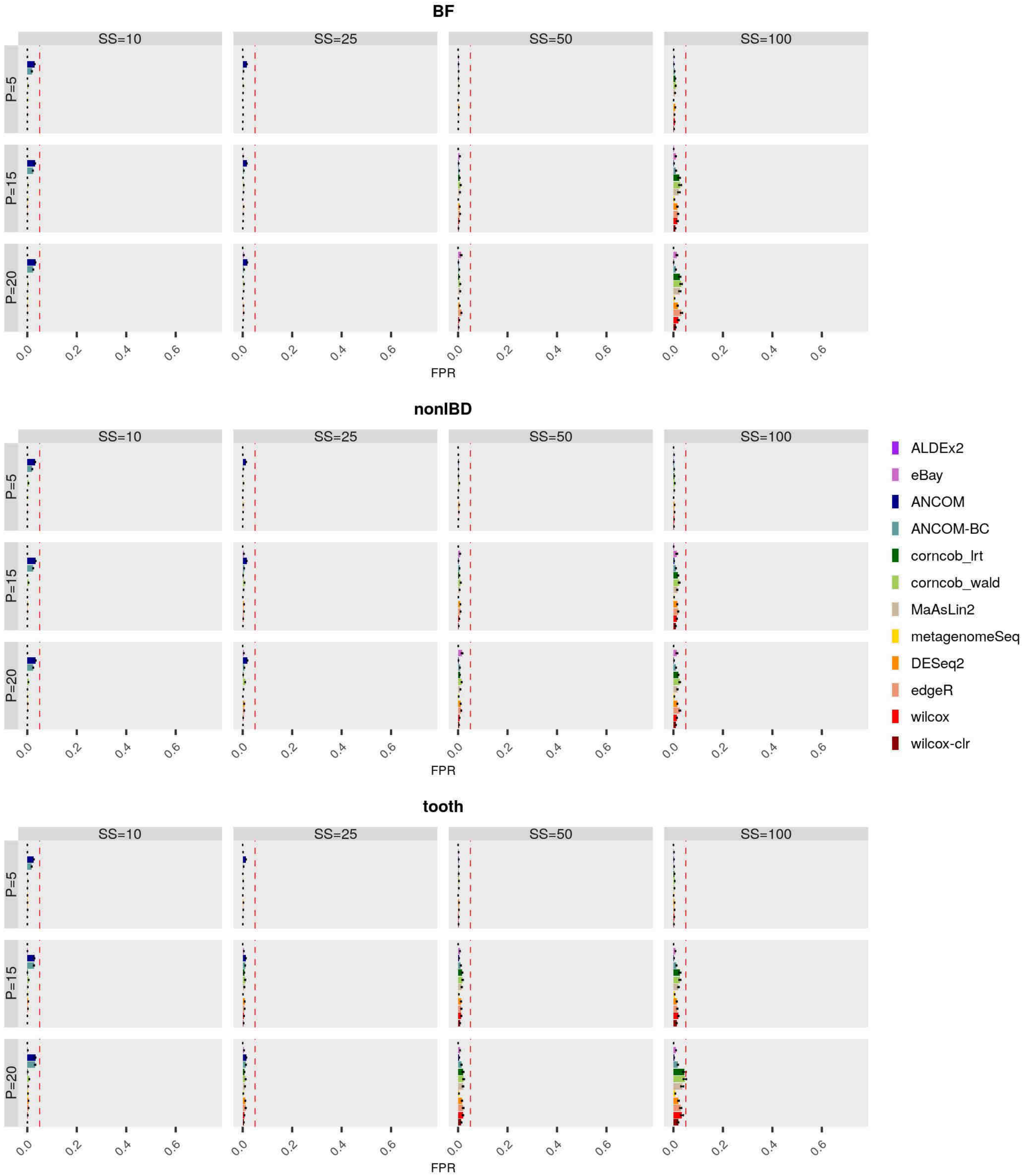

Figure 67. False Positive Rate (FPR) of each differential abundance method for each dataset considered in the comparison in the scenario considering half variability in simulation. In each set of boxes corresponding to the dataset, different percentages (P) of simulated DA features are on rows, while different sample size (SS) values are on columns. The FPR values are averaged over the 50 simulations and the bars show the standard error.

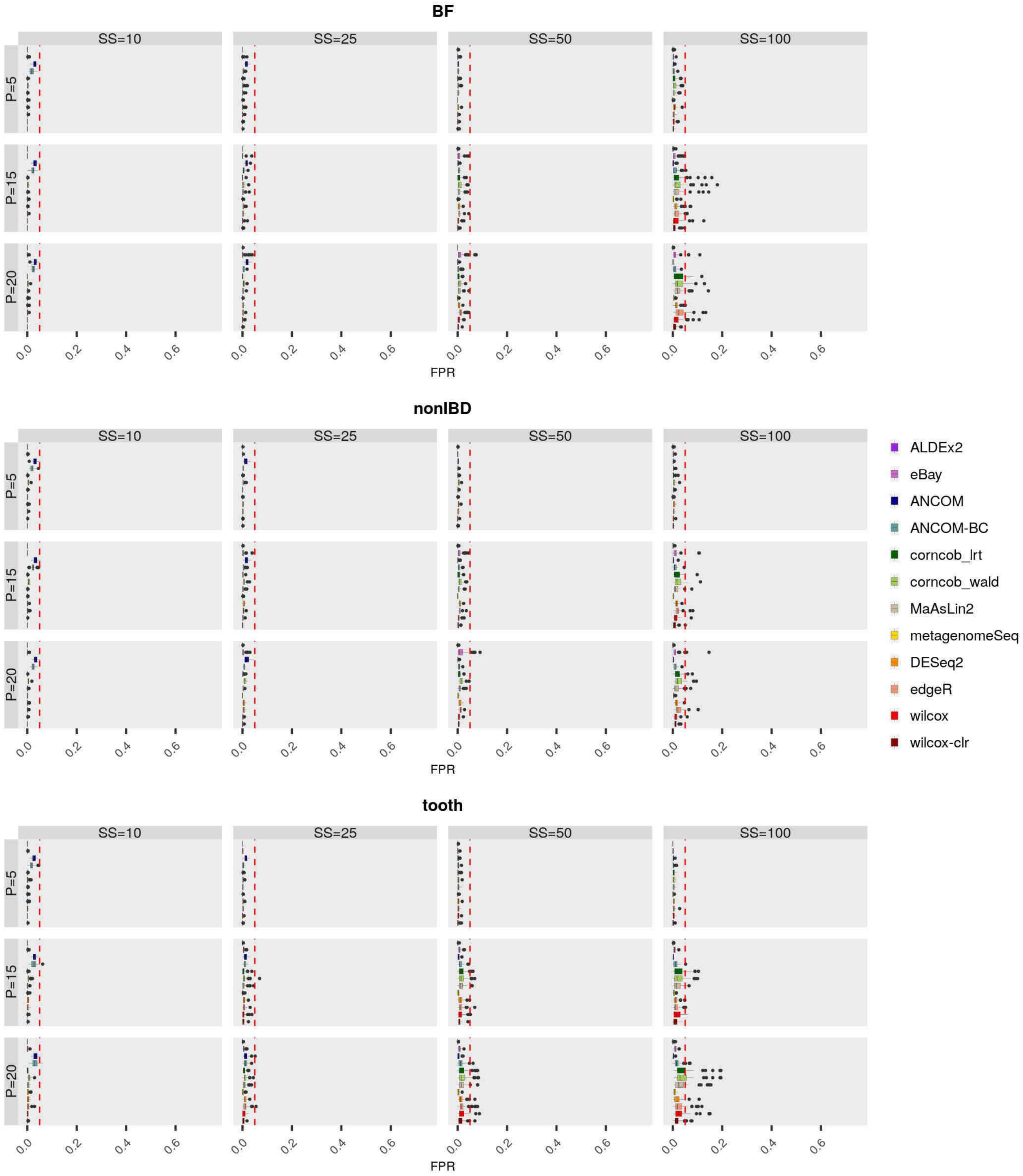

Figure 68. Boxplot of the False Positive Rate (FPR) distribution across the 50 simulations of each differential abundance method for each dataset considered in the comparison in the scenario considering half variability in simulation. In each set of boxes corresponding to the dataset, different percentages (P) of simulated DA features are on rows, while different sample size (SS) values are on columns.

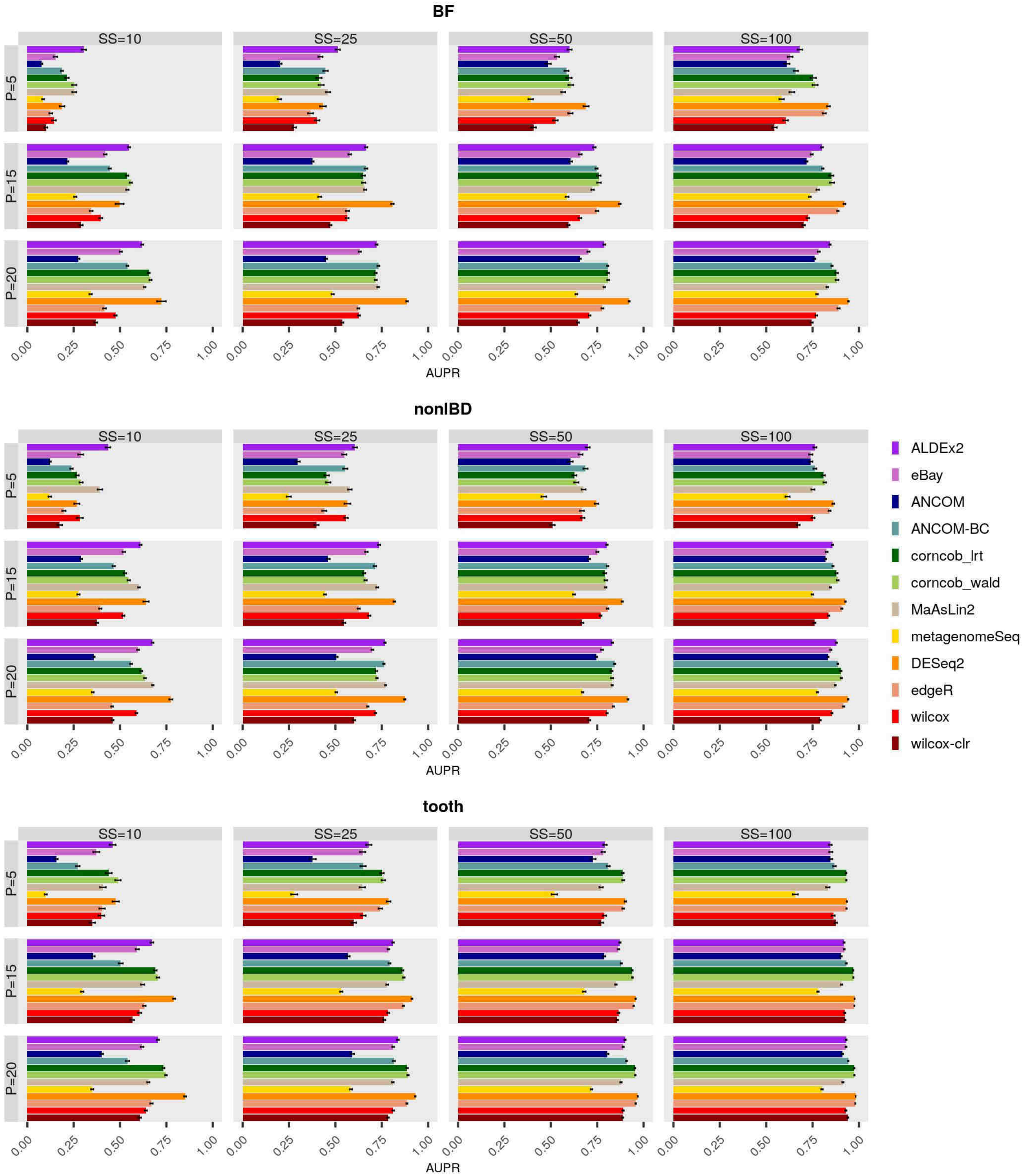

Figure 69. Area Under Precision-Recall curves (AUPR) of each differential abundance method for each dataset considered in the comparison in the scenario considering half variability in simulation. In each set of boxes corresponding to the dataset, different percentages of simulated DA features are on rows, while different sample size (SS) values are on columns. The AUPR values are averaged over the 50 simulations and the bars show the standard error.

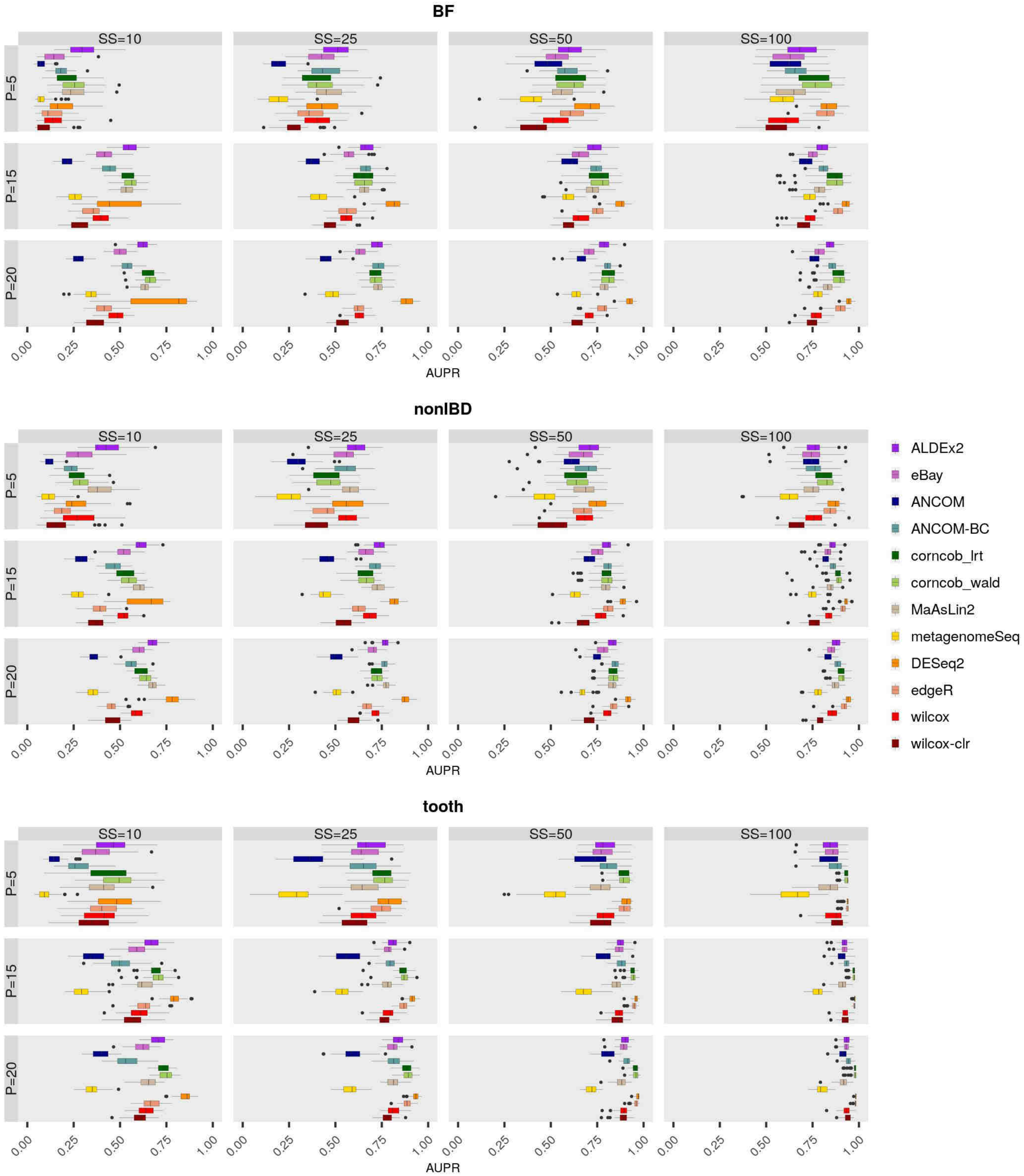

Figure 70. Boxplot of the Area Under Precision-Recall curves (AUPR) of each differential abundance method for each dataset considered in the comparison in the scenario considering half variability in simulation. In each set of boxes corresponding to the dataset, different percentages (P) of simulated DA features are on rows, while different sample size (SS) values are on columns.

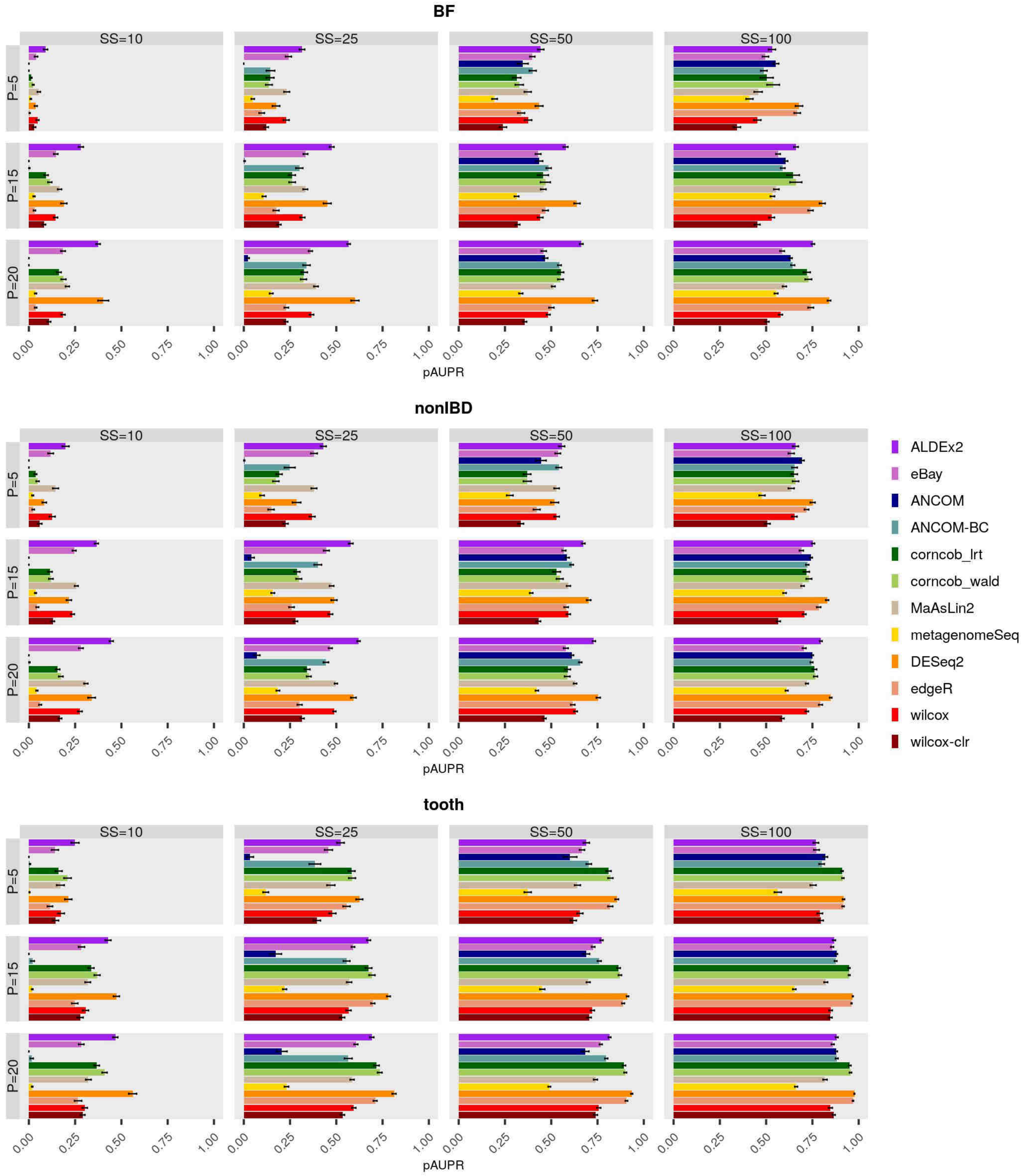

Figure 71. Partial Area Under Precision-Recall curves (pAUPR) of each differential abundance method for each dataset considered in the comparison in the scenario considering half variability in simulation. In each set of boxes corresponding to the dataset, different percentages (P) of simulated DA features are on rows, while different sample size (SS) values are on columns. The pAUPR values are averaged over the 50 simulations and the bars show the standard error.

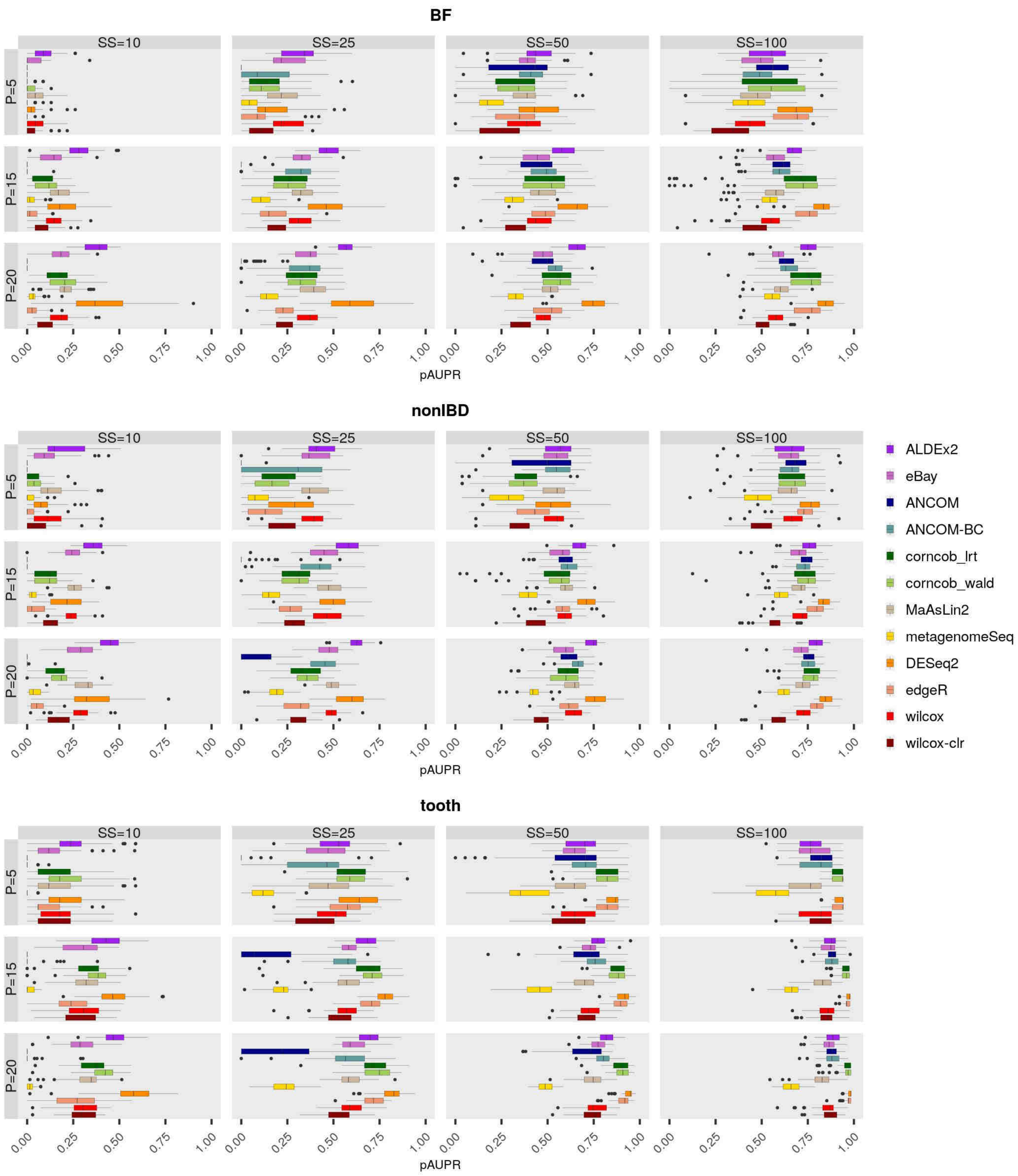

Figure 72. Boxplot of the partial Area Under Precision-Recall curves (pAUPR) of each differential abundance method for each dataset considered in the comparison in the scenario considering half variability in simulation. In each set of boxes corresponding to the dataset, different percentages (P) of simulated DA features are on rows, while different sample size (SS) values are on columns.

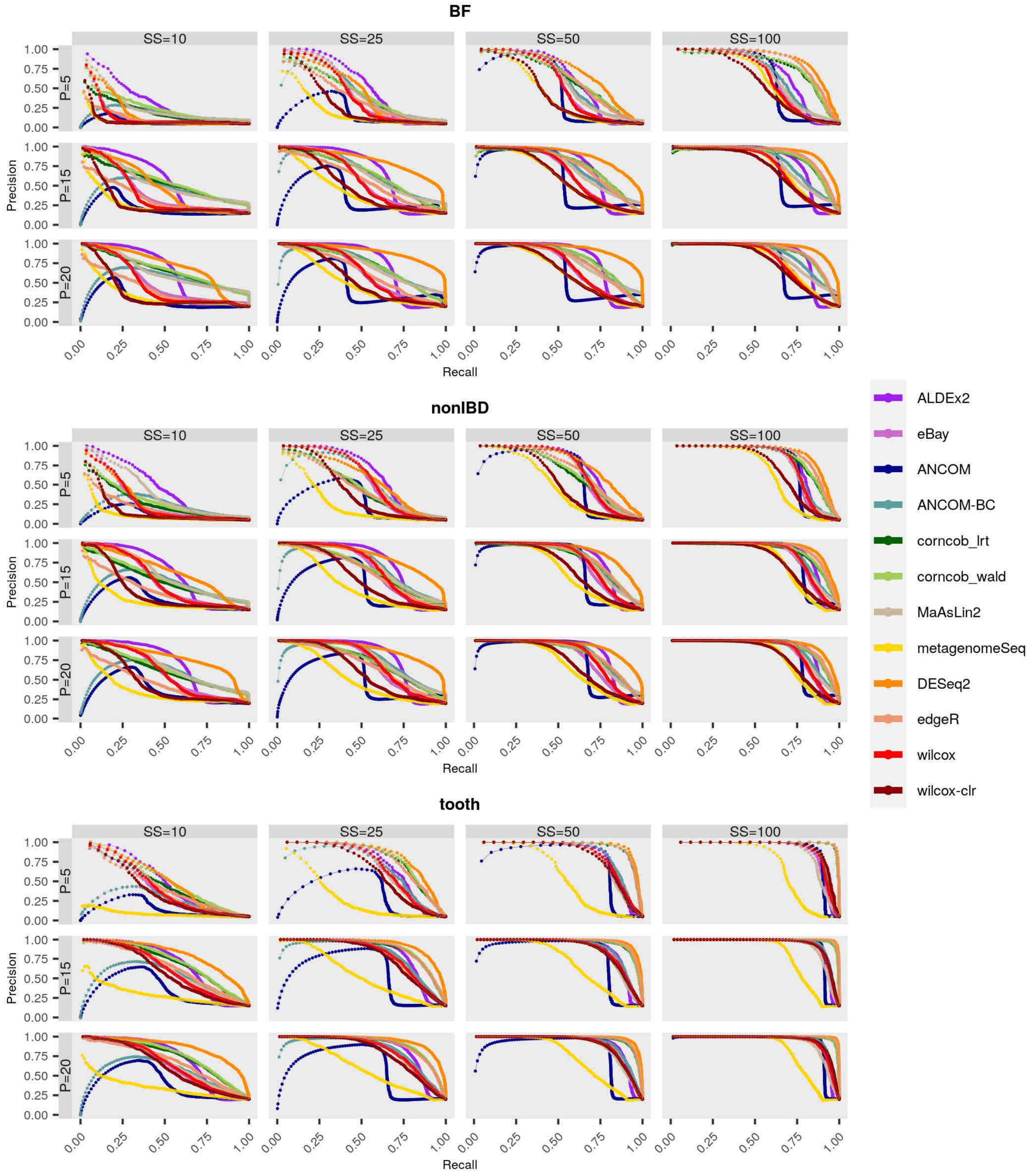

Figure 73. Mean Precision-Recall curves of each differential abundance method for each dataset considered in the comparison in the scenario considering half variability in simulation. In each set of boxes corresponding to the dataset, different percentages (P) of simulated DA features are on rows, while different sample size (SS) values are on columns. The mean pr-curve is defined as the average of the i-th precision value and the i-th recall value over the 50 simulations.

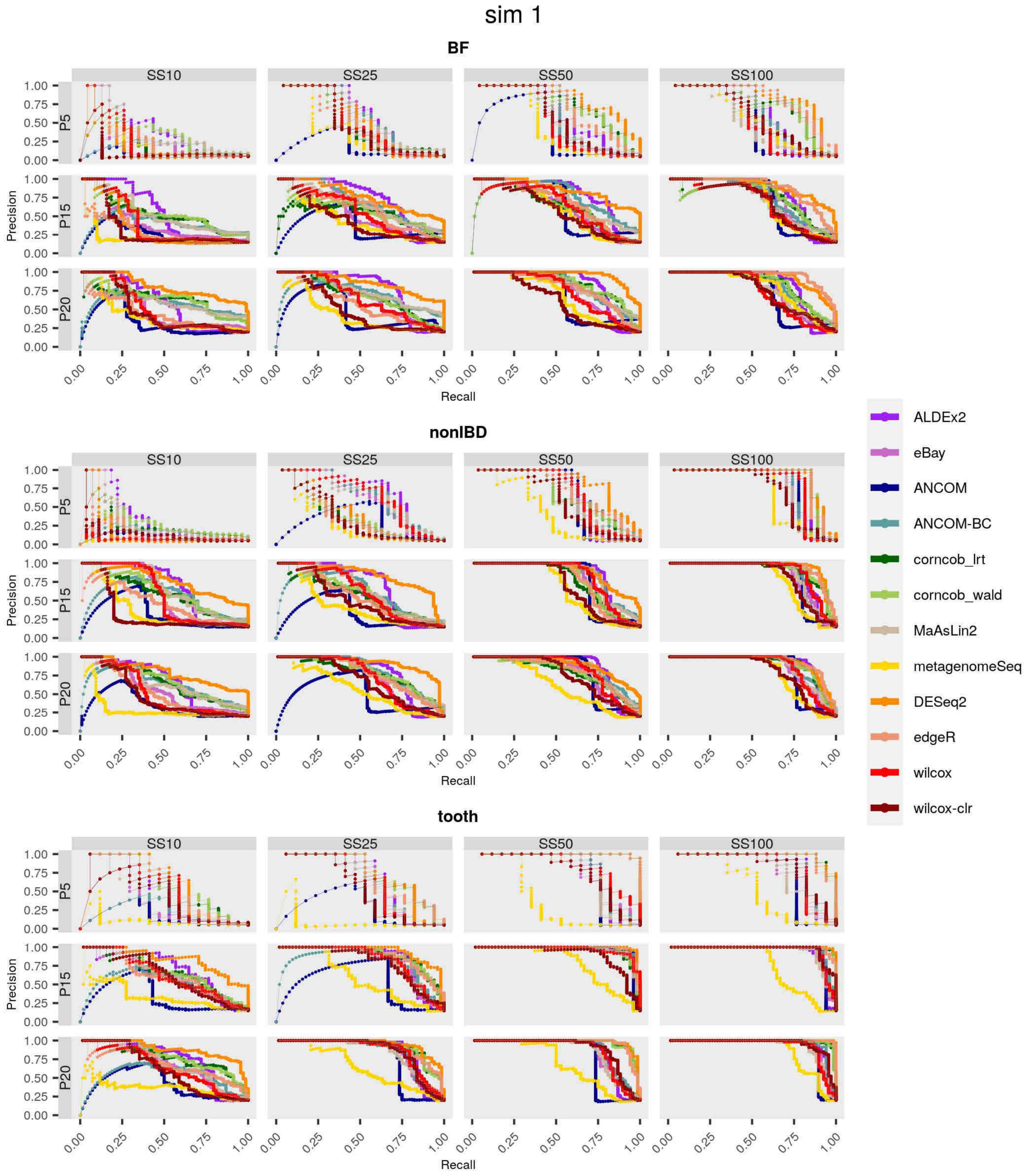

Figure 74. Simulation 1. Precision-Recall curves of each differential abundance method for each dataset considered in the comparison in the scenario considering half variability in simulation. In each set of boxes corresponding to the dataset, different percentages (P) of simulated DA features are on rows, while different sample size (SS) values are on columns.

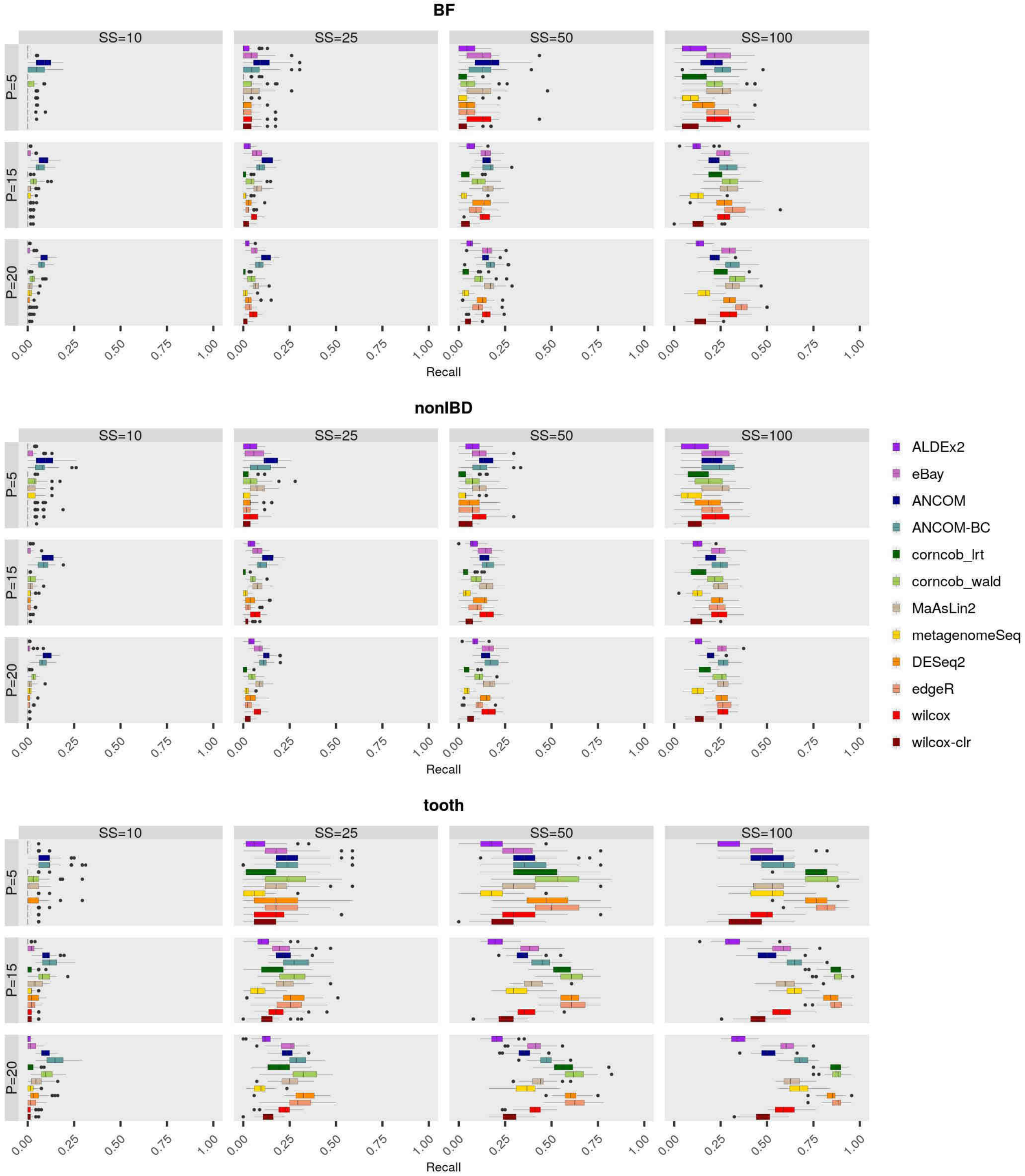

Figure 75. Boxplot of recall distribution across the 50 simulations of each differential abundance method for each dataset considered in the comparison in the scenario with simulated DA features and $\theta=0$. In each set of boxes corresponding to the dataset, different percentages (P) of simulated DA features are on rows, while different sample size (SS) values are on columns.

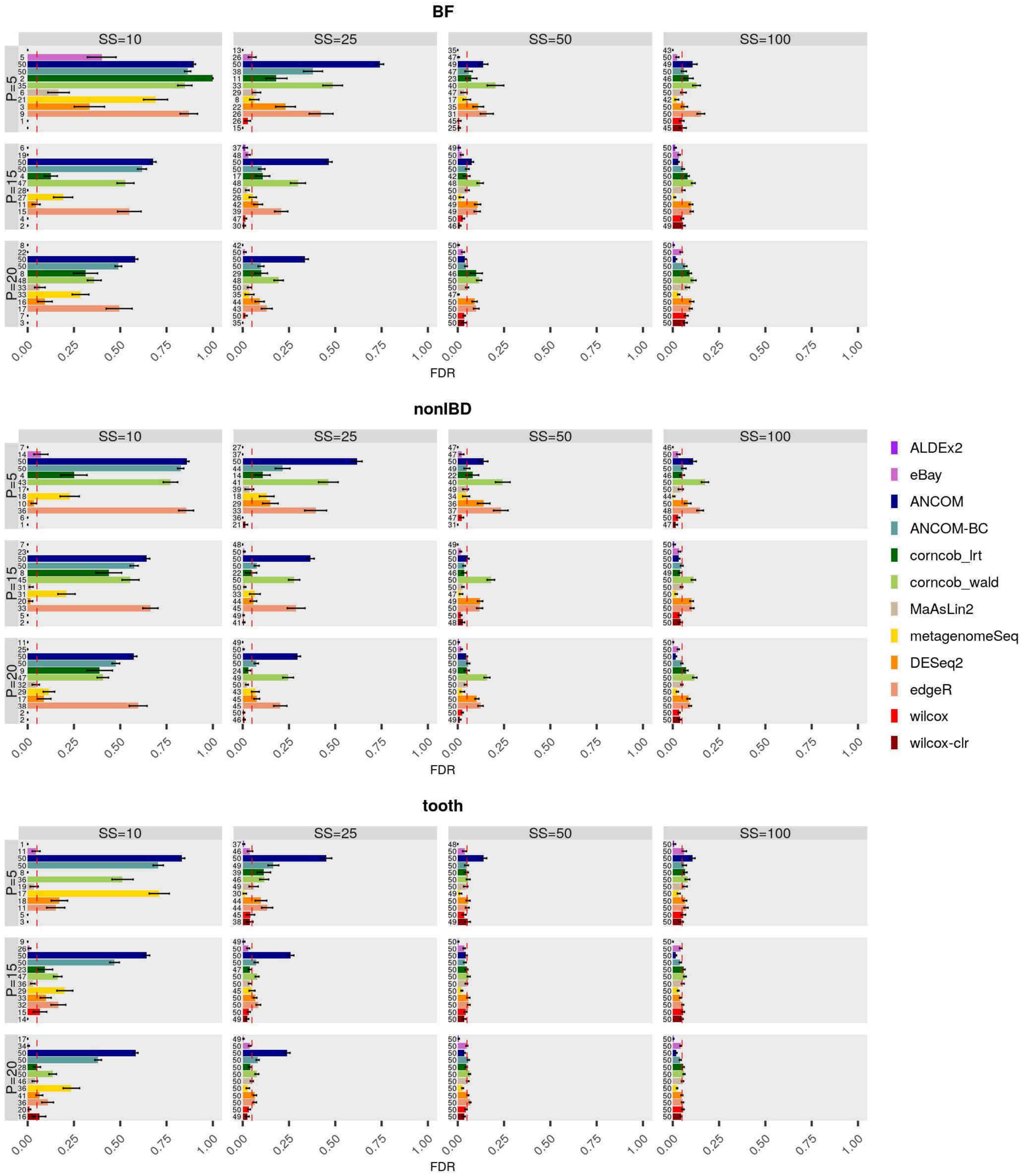

Figure 76. False Discovery Rate (FDR) of each differential abundance method for each dataset considered in the comparison in the scenario with simulated DA features and *θ=0*. In each set of boxes corresponding to the dataset, different percentages (P) of simulated DA features are on rows, while different sample size (SS) values are on columns. The FDR values are averaged over the 50 simulations and the bars show the standard error. The number of runs that provide a defined value of FDR is shown at the beginning of the bars.

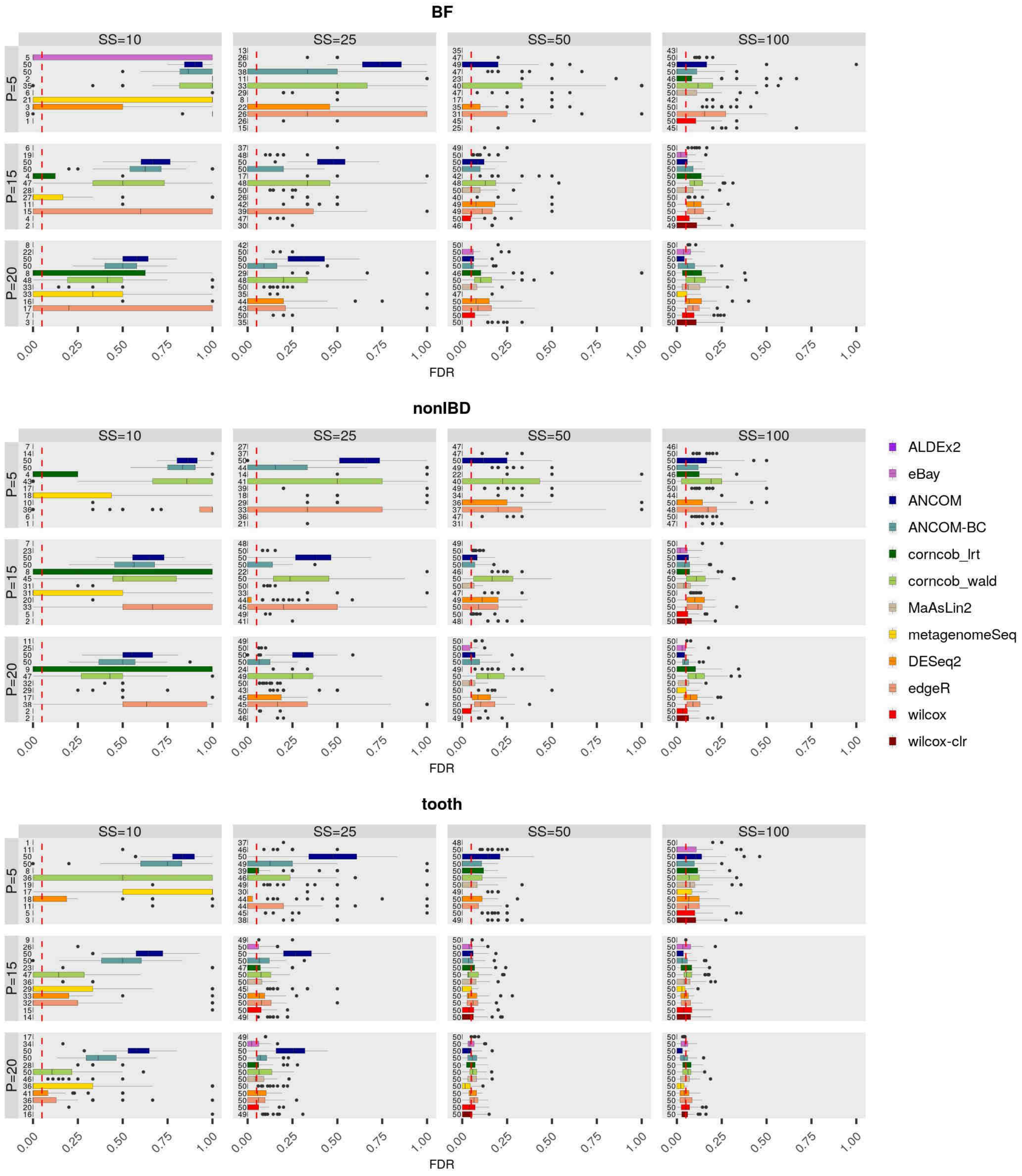

Figure 77. Boxplot of the False Discovery Rate (FDR) distribution across the 50 simulations of each differential abundance method for each dataset considered in the comparison in the scenario with simulated DA features and *θ=0*. In each set of boxes corresponding to the dataset, different percentages (P) of simulated DA features are on rows, while different sample size (SS) values are on columns. The number of runs that provide a defined value of FDR is shown on the left of each boxplot.

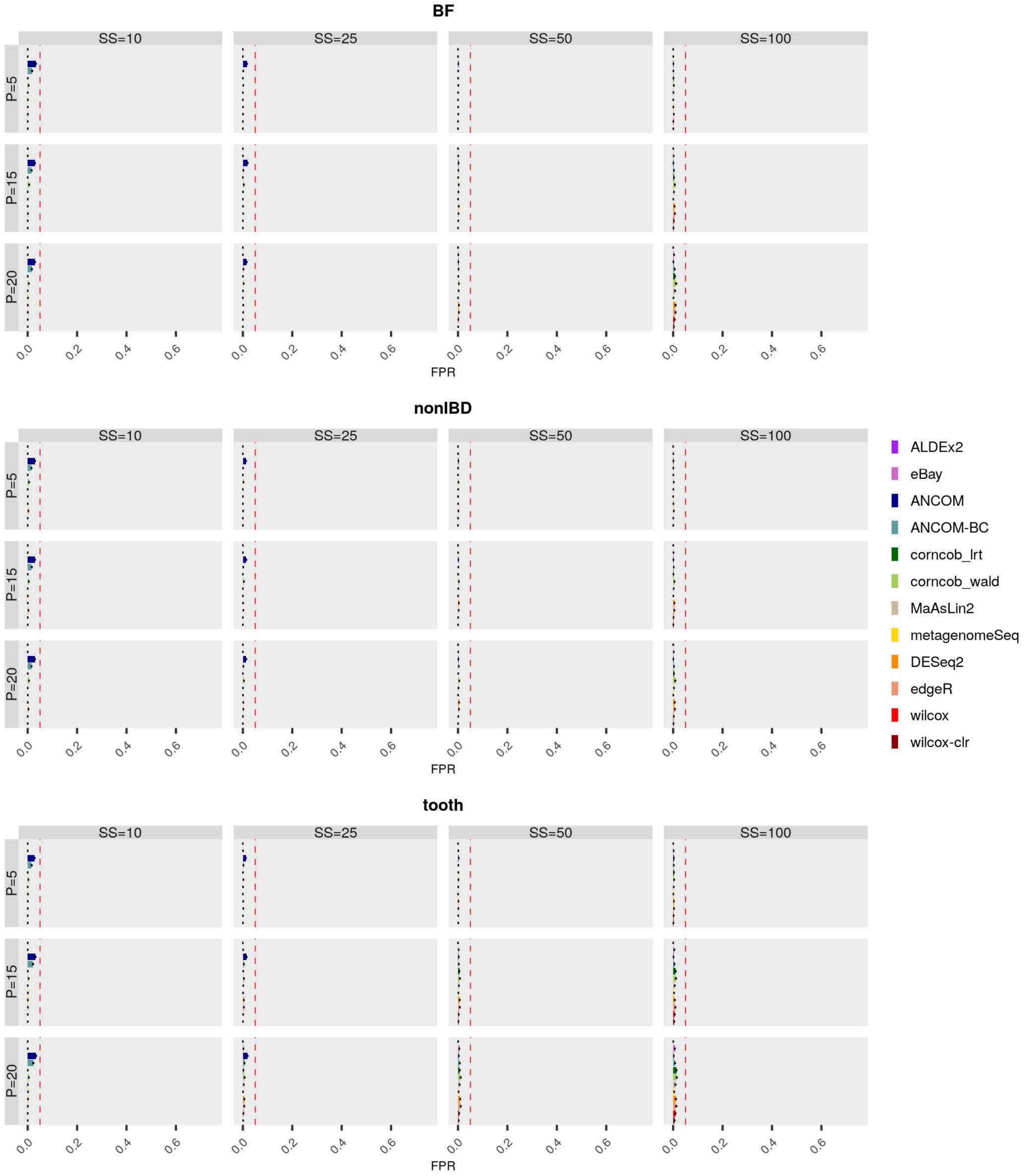

Figure 78. False Positive Rate (FPR) of each differential abundance method for each dataset considered in the comparison in the scenario with simulated DA features and *θ=0*. In each set of boxes corresponding to the dataset, different percentages (P) of simulated DA features are on rows, while different sample size (SS) values are on columns. The FPR values are averaged over the 50 simulations and the bars show the standard error.

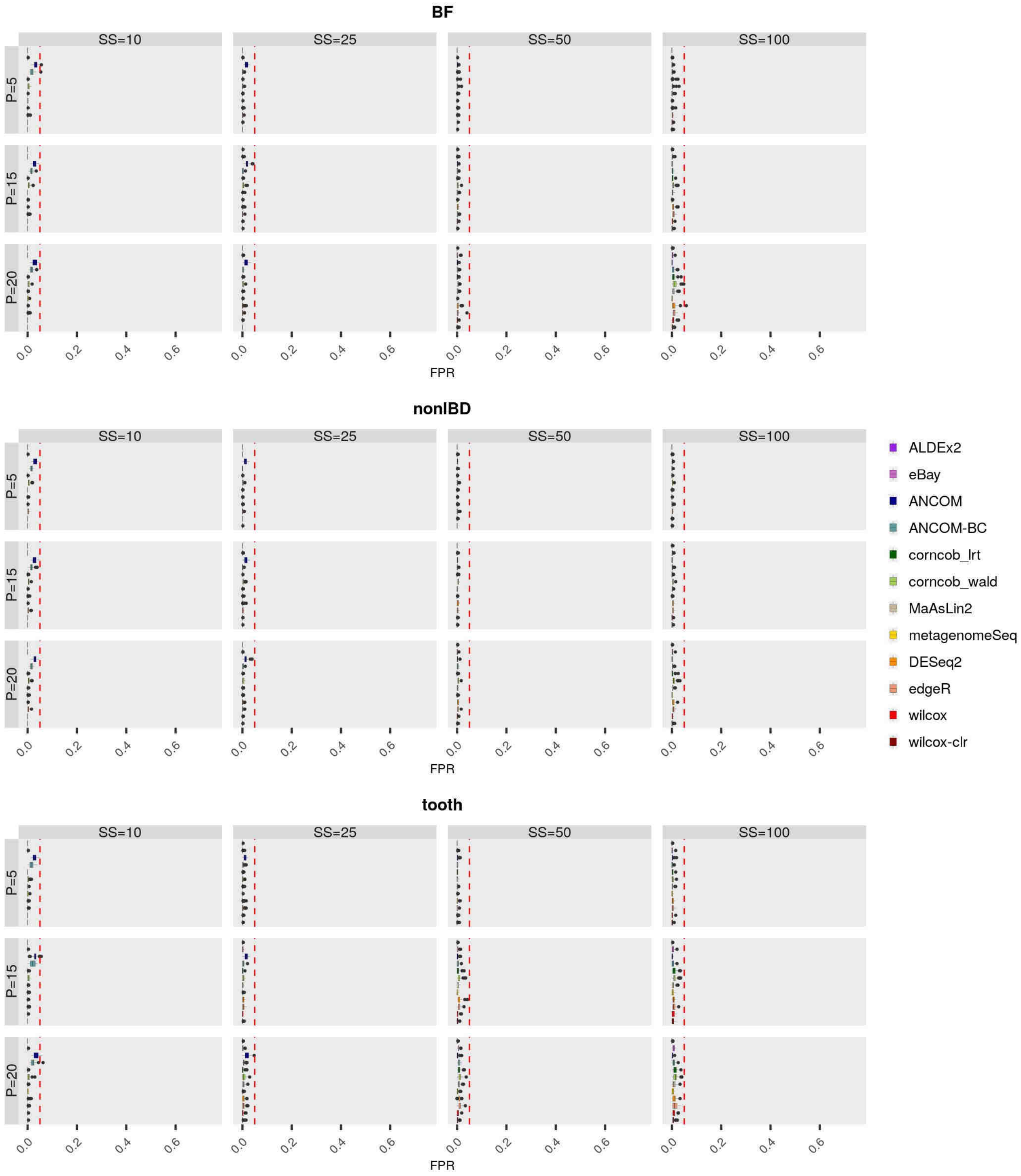

Figure 79. Boxplot of the False Positive Rate (FPR) distribution across the 50 simulations of each differential abundance method for each dataset considered in the comparison in the scenario with simulated DA features and $\theta=0$. In each set of boxes corresponding to the dataset, different percentages (P) of DA features are on rows, while different sample size (SS) values are on columns.

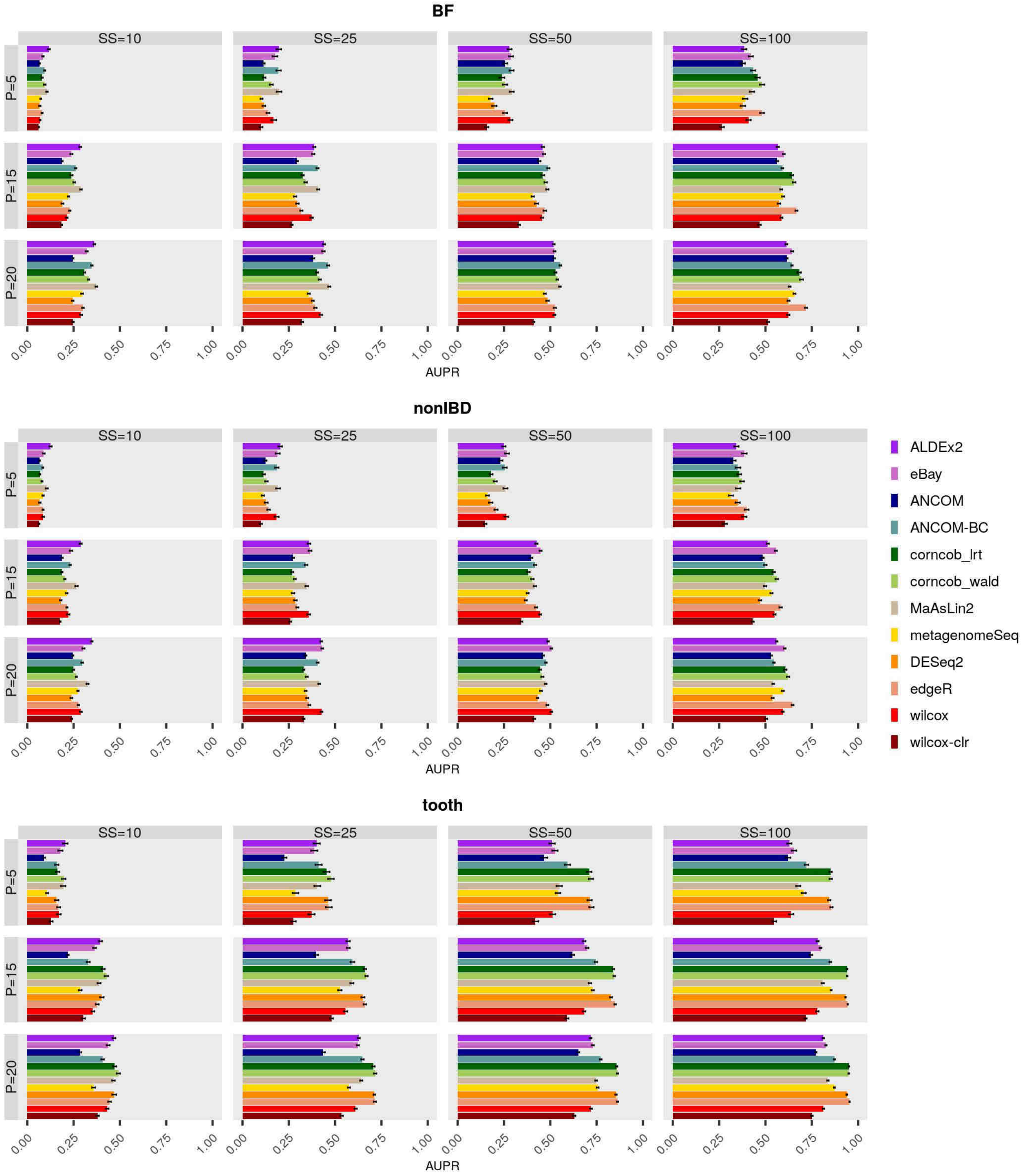

Figure 80. Area Under Precision-Recall curves (AUPR) of each differential abundance method for each dataset considered in the comparison in the scenario with simulated DA features and $\theta=0$. In each set of boxes corresponding to the dataset, different percentages (P) of simulated DA features are on rows, while different sample size (SS) values are on columns. The AUPR values are averaged over the 50 simulations and the bars show the standard error.

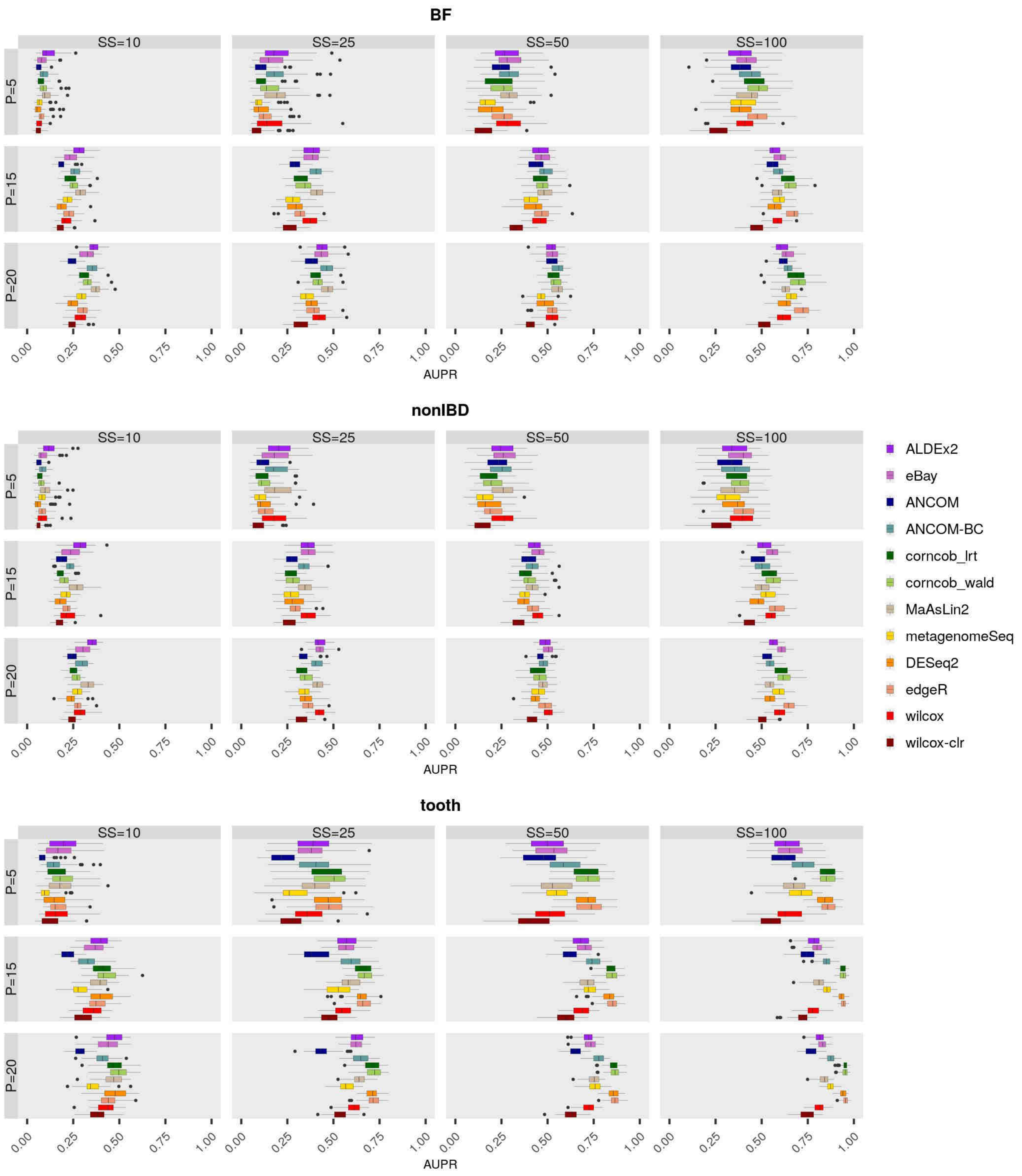

Figure 81. Boxplot of the Area Under Precision-Recall curves (AUPR) of each differential abundance method for each dataset considered in the comparison in the scenario with simulated DA features and $\theta=0$. In each set of boxes corresponding to the dataset, different percentages (P) of simulated DA features are on rows, while different sample size (SS) values are on columns.

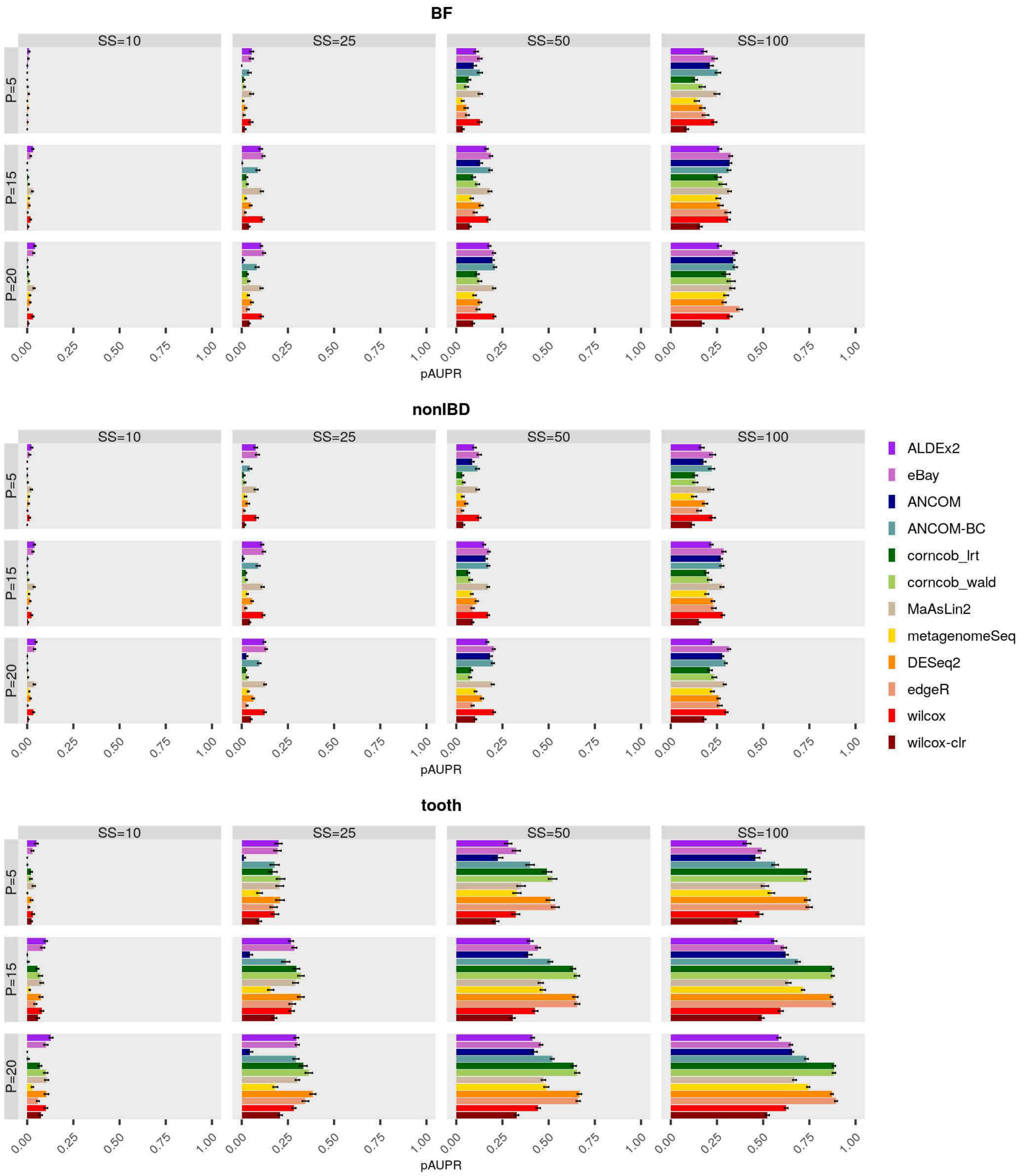

Figure 82. Partial Area Under Precision-Recall curves (pAUPR) of each differential abundance method for each dataset considered in the comparison in the scenario with simulated DA features and *θ=0*. In each set of boxes corresponding to the dataset, different percentages (P) of simulated DA features are on rows, while different sample size (SS) values are on columns. The pAUPR values are averaged over the 50 simulations and the bars show the standard error.

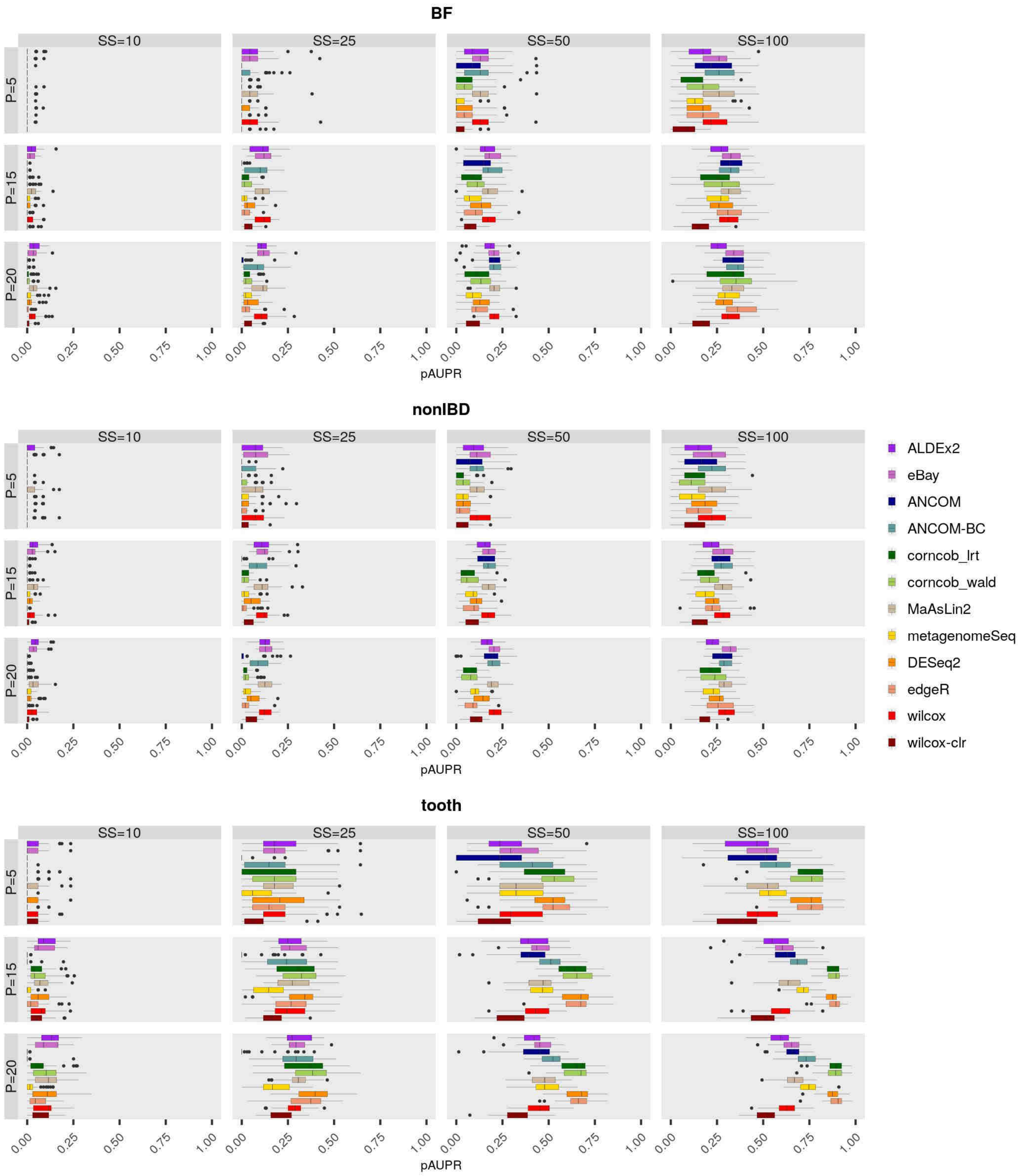

Figure 83. Boxplot of the partial Area Under Precision-Recall curves (pAUPR) of each differential abundance method for each dataset considered in the comparison in the scenario with simulated DA features and θ=0. In each set of boxes corresponding to the dataset, different percentages (P) of simulated DA features are on rows, while different sample size (SS) values are on columns.

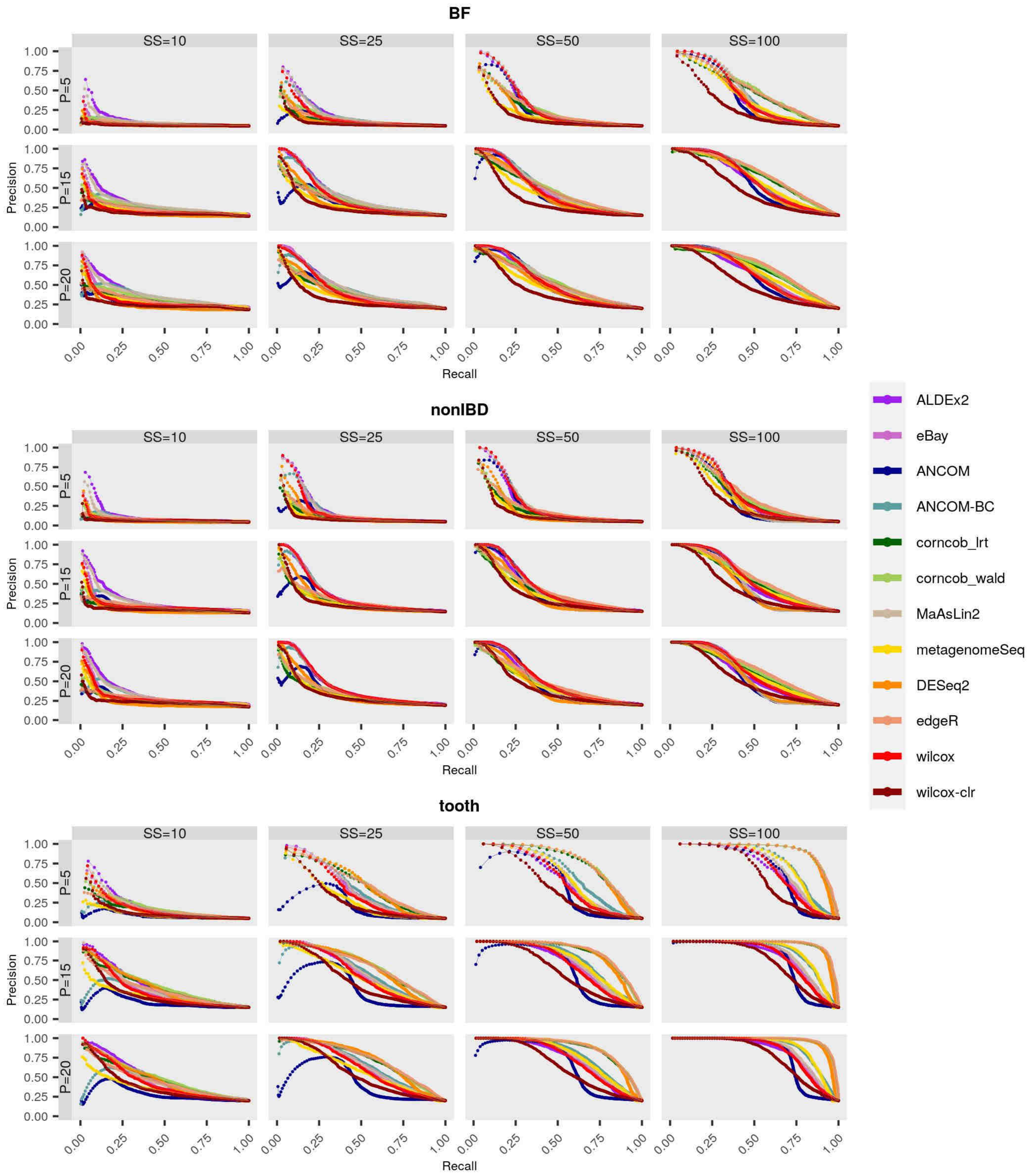

Figure 84. Mean Precision-Recall curves of each differential abundance method for each dataset considered in the comparison in the scenario with simulated DA features and *θ=0*. In each set of boxes corresponding to the dataset, different percentages (P) of simulated DA features are on rows, while different sample size (SS) values are on columns. The mean pr-curve is defined as the average of the i-th precision value and the i-th recall value over the 50 simulations.

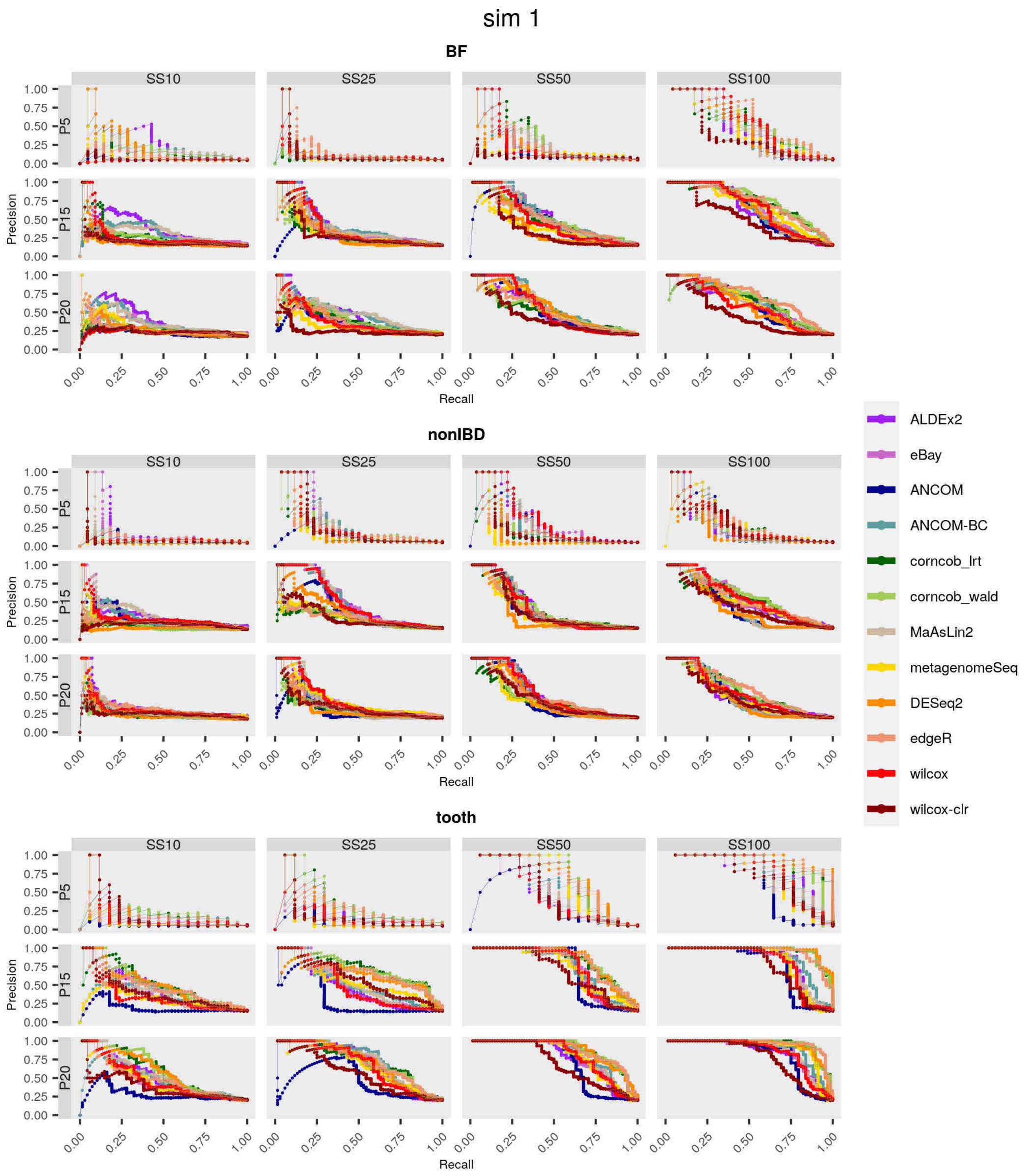

Figure 85. Simulation 1. Precision-Recall curves of each differential abundance method for each dataset considered in the comparison in the scenario with simulated DA features and $\theta=0$. In each set of boxes corresponding to the dataset, different percentages (P) of simulated DA features are on rows, while different sample size (SS) values are on columns.

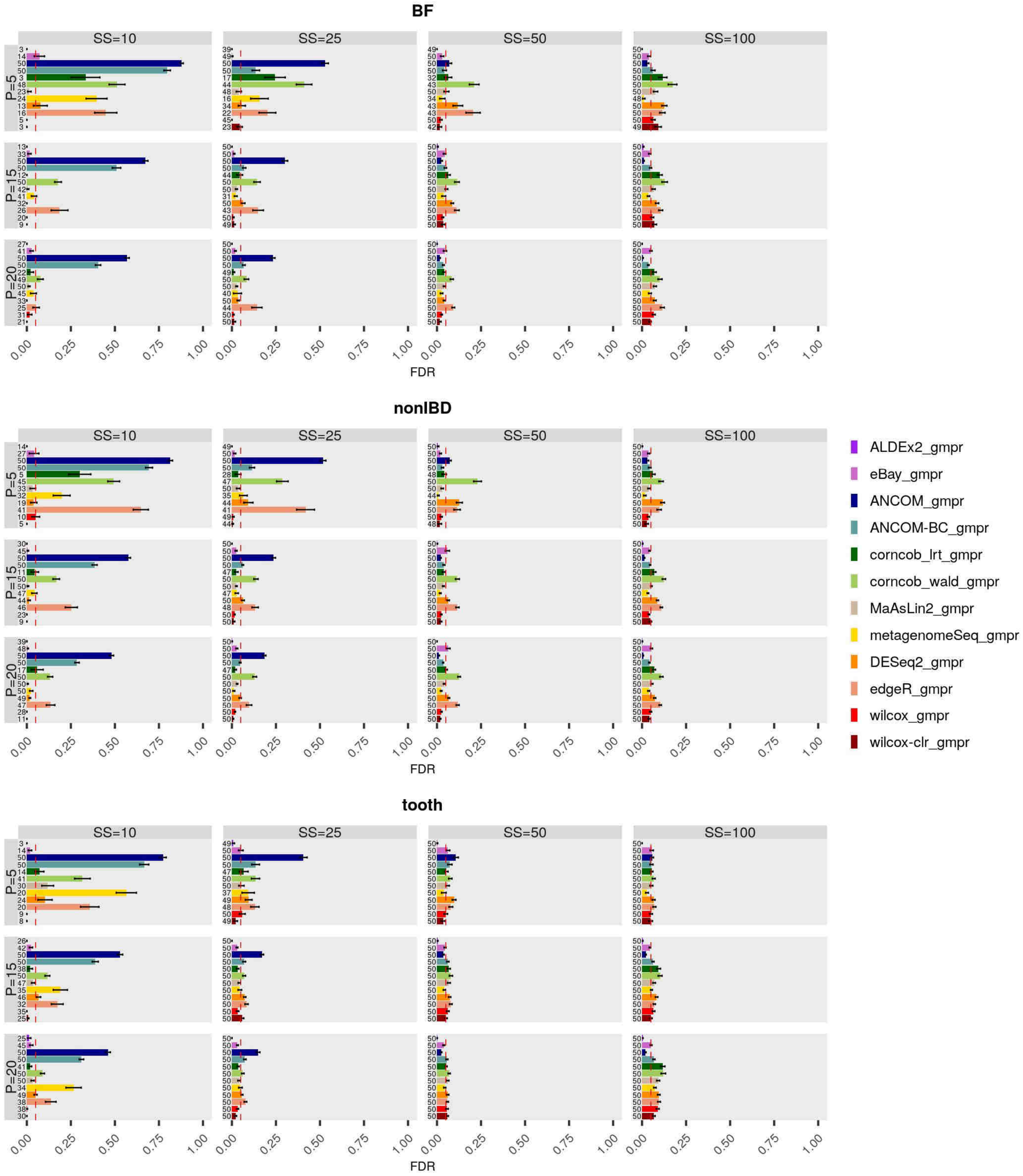

Figure 86. False Discovery Rate (FDR) of each differential abundance method normalised with GMPR for each dataset considered in the comparison in the main scenario with simulated DA features. In each set of boxes corresponding to the dataset, different percentages (P) of simulated DA features are on rows, while different sample size (SS) values are on columns. The FDR values are averaged over the 50 simulations and the bars show the standard error. The number of runs that provide a defined value of FDR is shown at the beginning of the bars.

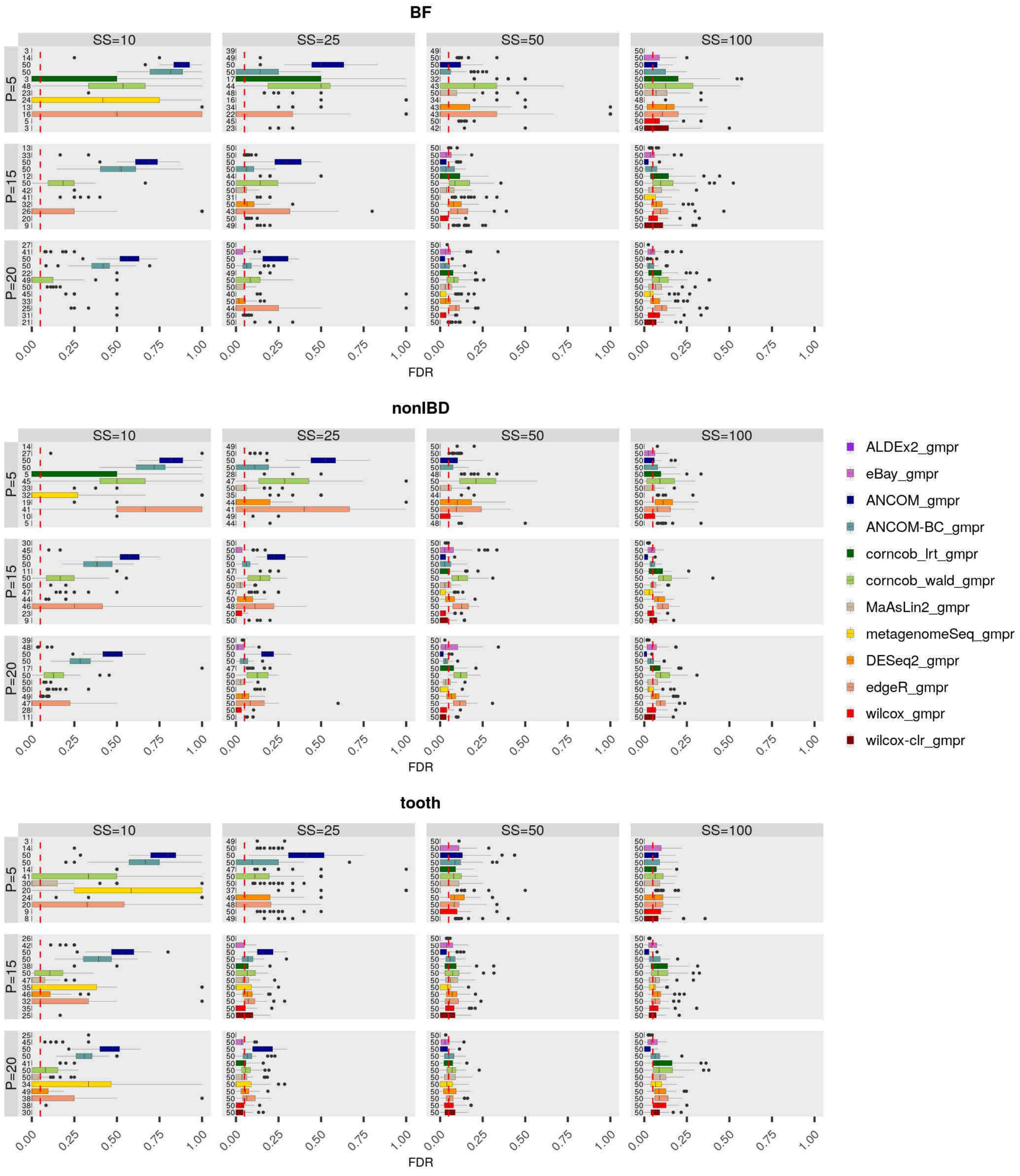

Figure 87. Boxplot of the False Discovery Rate (FDR) distribution across the 50 simulations of each differential abundance method normalised with GMPR for each dataset considered in the comparison in the main scenario with simulated DA features. In each set of boxes corresponding to the dataset, different percentages (P) of simulated DA features are on rows, while different sample size (SS) values are on columns. The number of runs that provide a defined value of FDR is shown on the left of each boxplot.

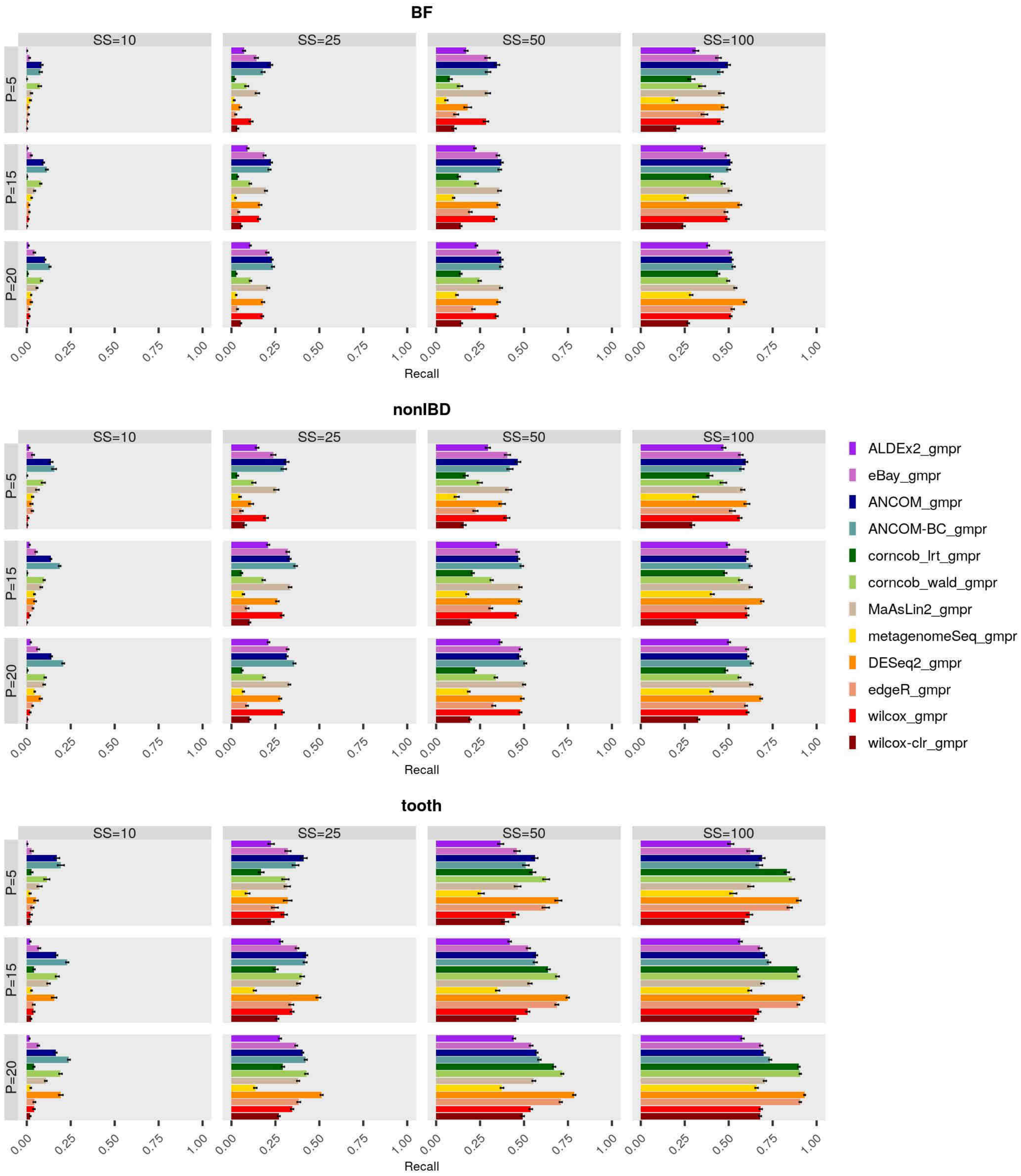

Figure 88. Recall of each differential abundance method normalised with GMPR for each dataset considered in the comparison in the main scenario with simulated DA features. In each set of boxes corresponding to the dataset, different percentages (P) of simulated DA features are on rows, while different sample size (SS) values are on columns. The recall values are averaged over the 50 simulations and the bars show the standard error.

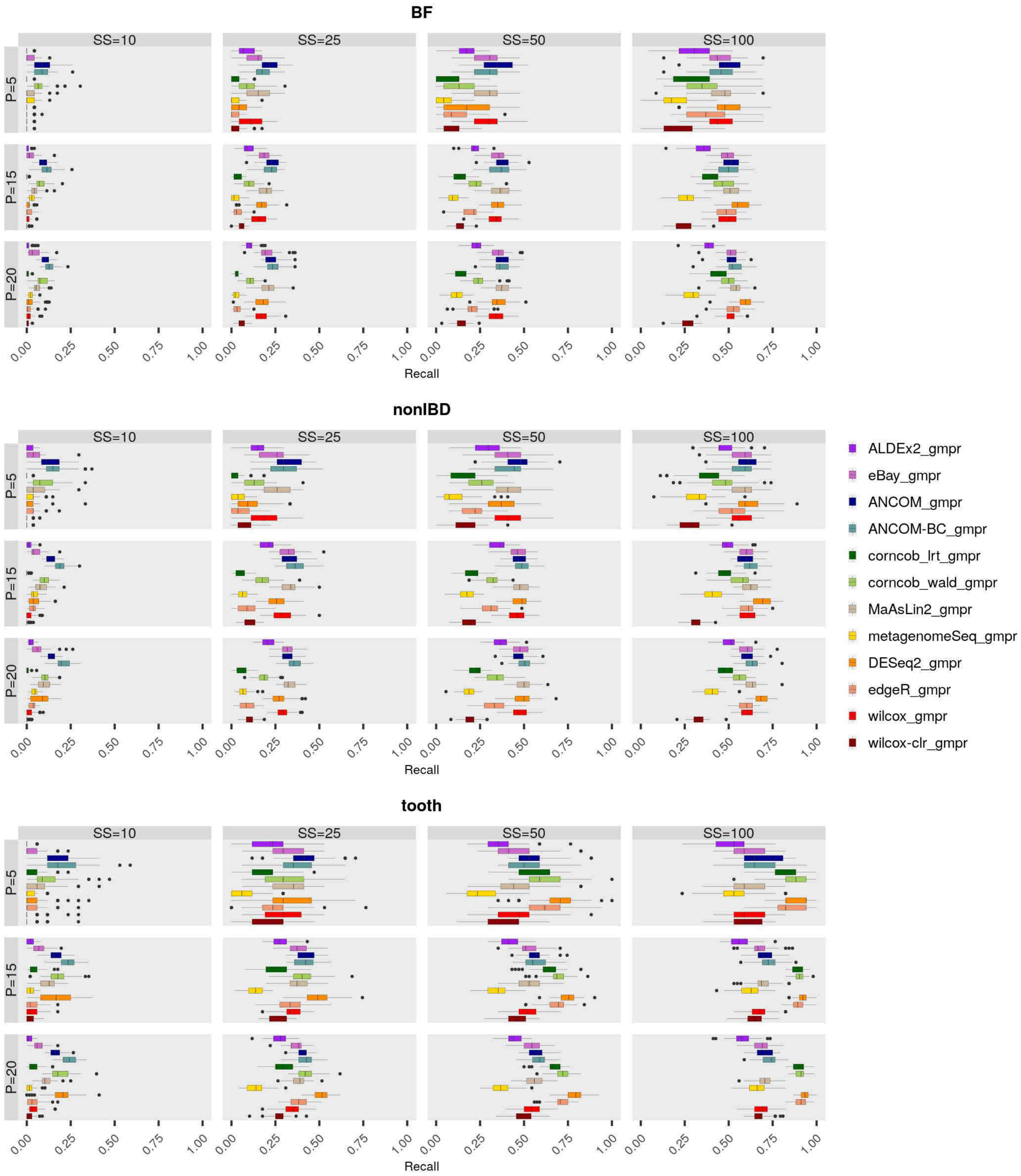

Figure 89. Boxplot of recall distribution across the 50 simulations of each differential abundance method normalised with GMPR for each dataset considered in the comparison in the main scenario with simulated DA features. In each set of boxes corresponding to the dataset, different percentages (P) of simulated DA features are on rows, while different sample size (SS) values are on columns.

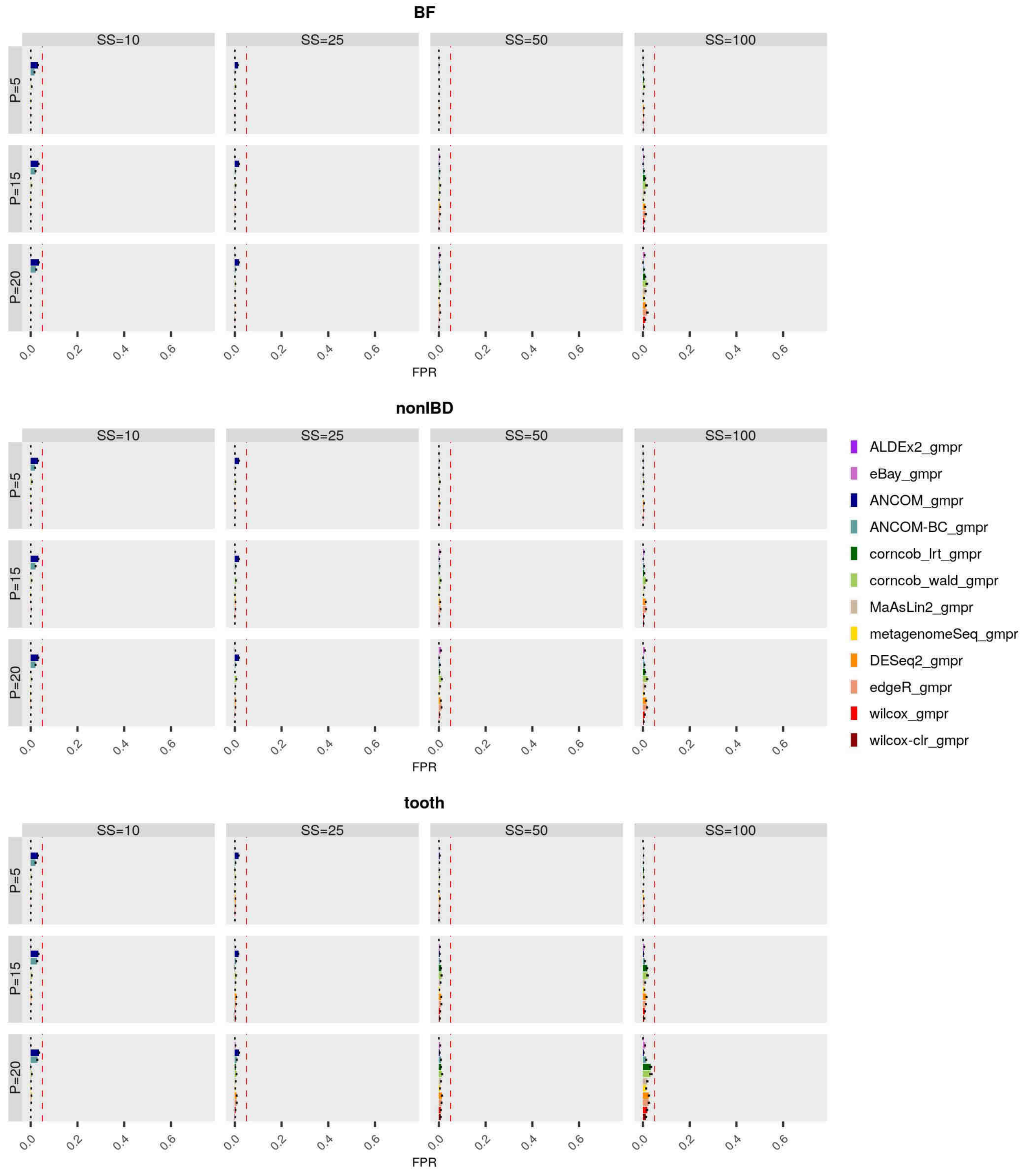

Figure 90. False Positive Rate (FPR) of each differential abundance method normalised with GMPR for each dataset considered in the comparison in the scenario without simulated DA features. In each set of boxes corresponding to the dataset, tools are on rows, while different sample size (SS) values are on columns. The FPR values are averaged over the 50 simulations and the bars show the standard error.

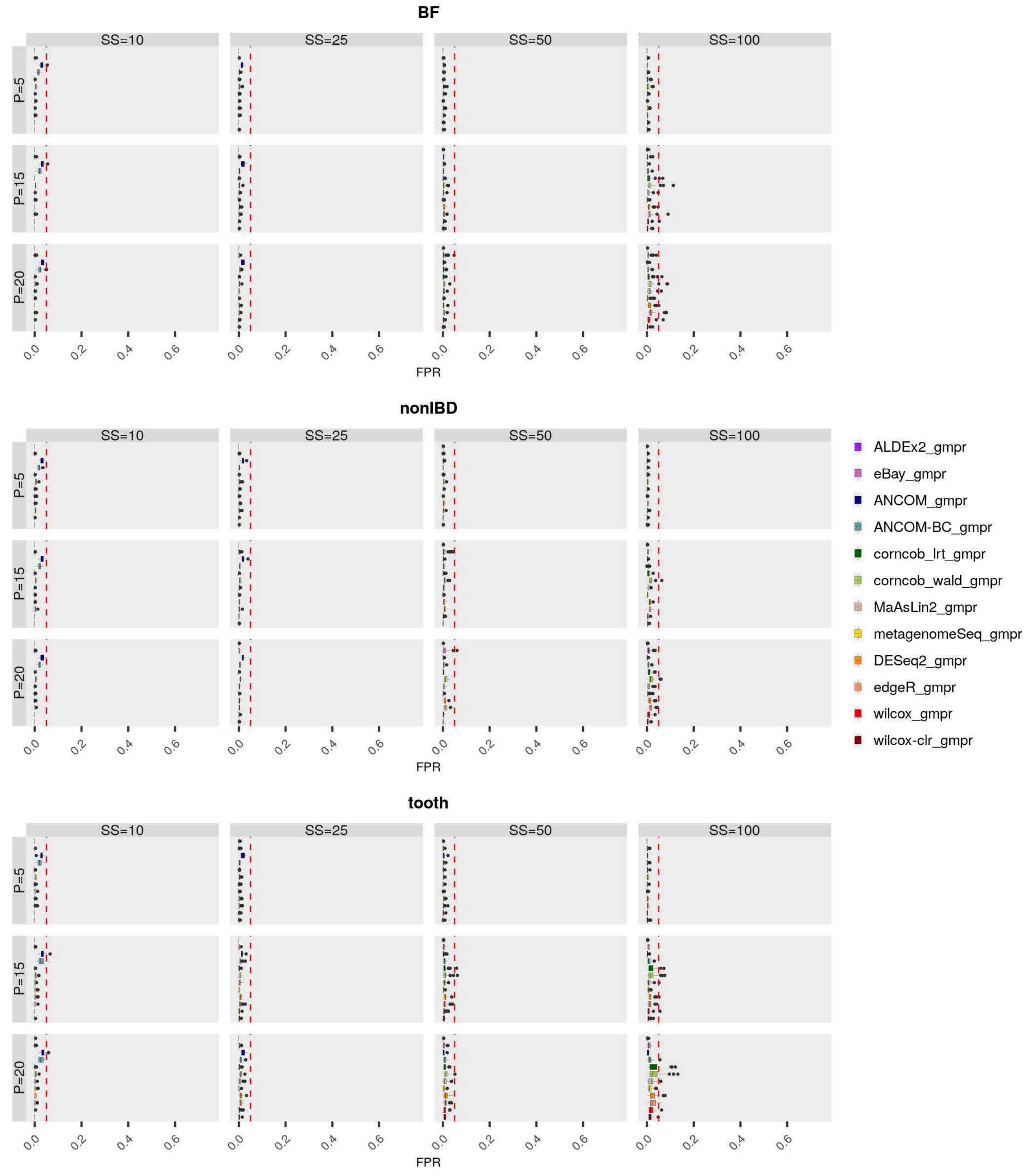

Figure 91. Boxplot of the False Positive Rate (FPR) distribution across the 50 simulations of each differential abundance method normalised with GMPR for each dataset considered in the comparison in the scenario without simulated DA features. In each set of boxes corresponding to the dataset, tools are on rows, while different sample size (SS) values are on columns.

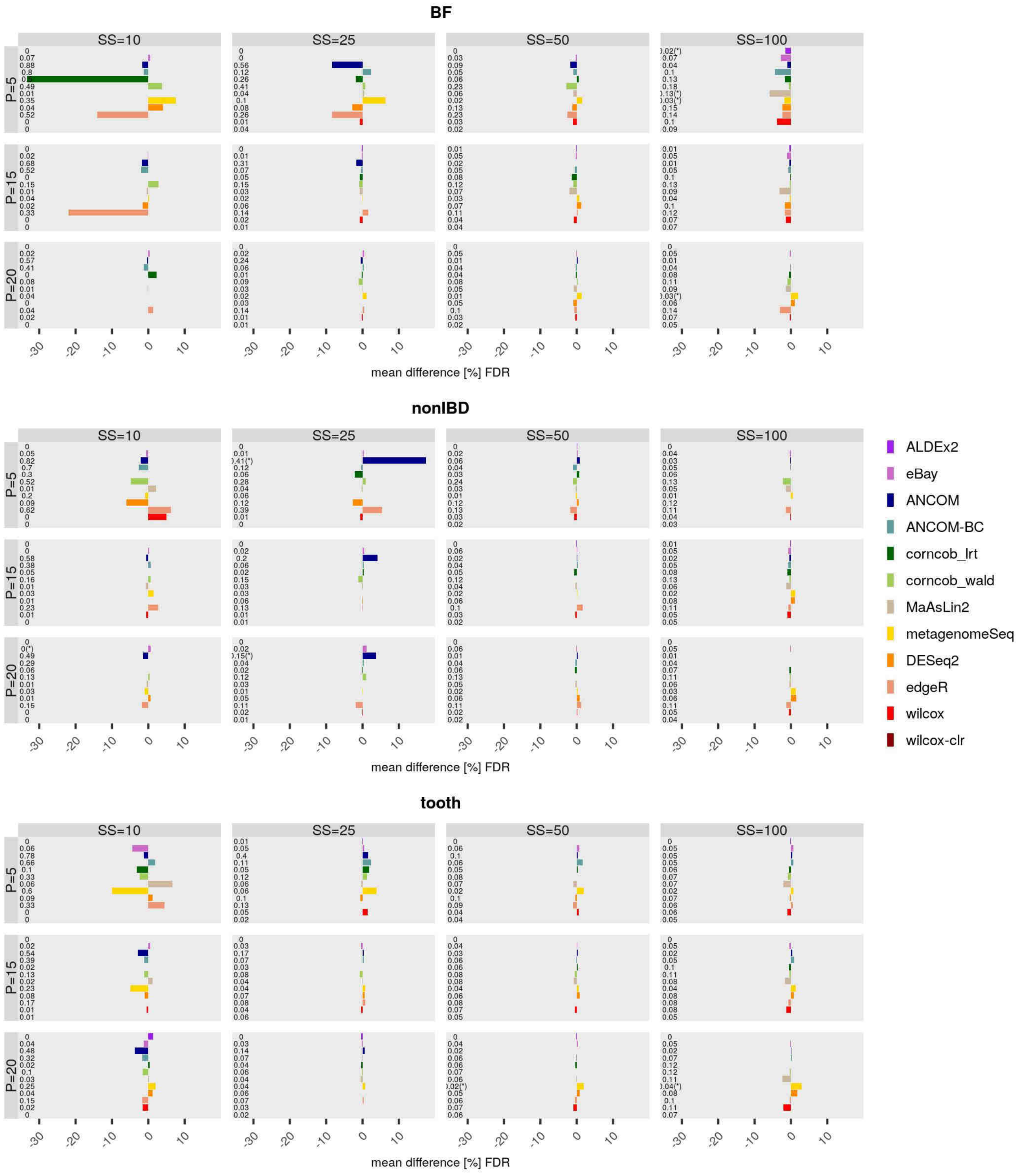

Figure 92. Mean FDR difference [%] between each differential abundance method and its GMPR normalised version for each dataset considered in the comparison in the scenario with simulated DA features. In each set of boxes corresponding to the dataset, different percentages (P) of simulated DA features are on rows, while different sample size (SS) values are on columns. Numbers at the beginning of each row correspond to the FDR values obtained with default normalization, while the symbol (*) identifies the Wilcoxon unpaired statistical test is significant.

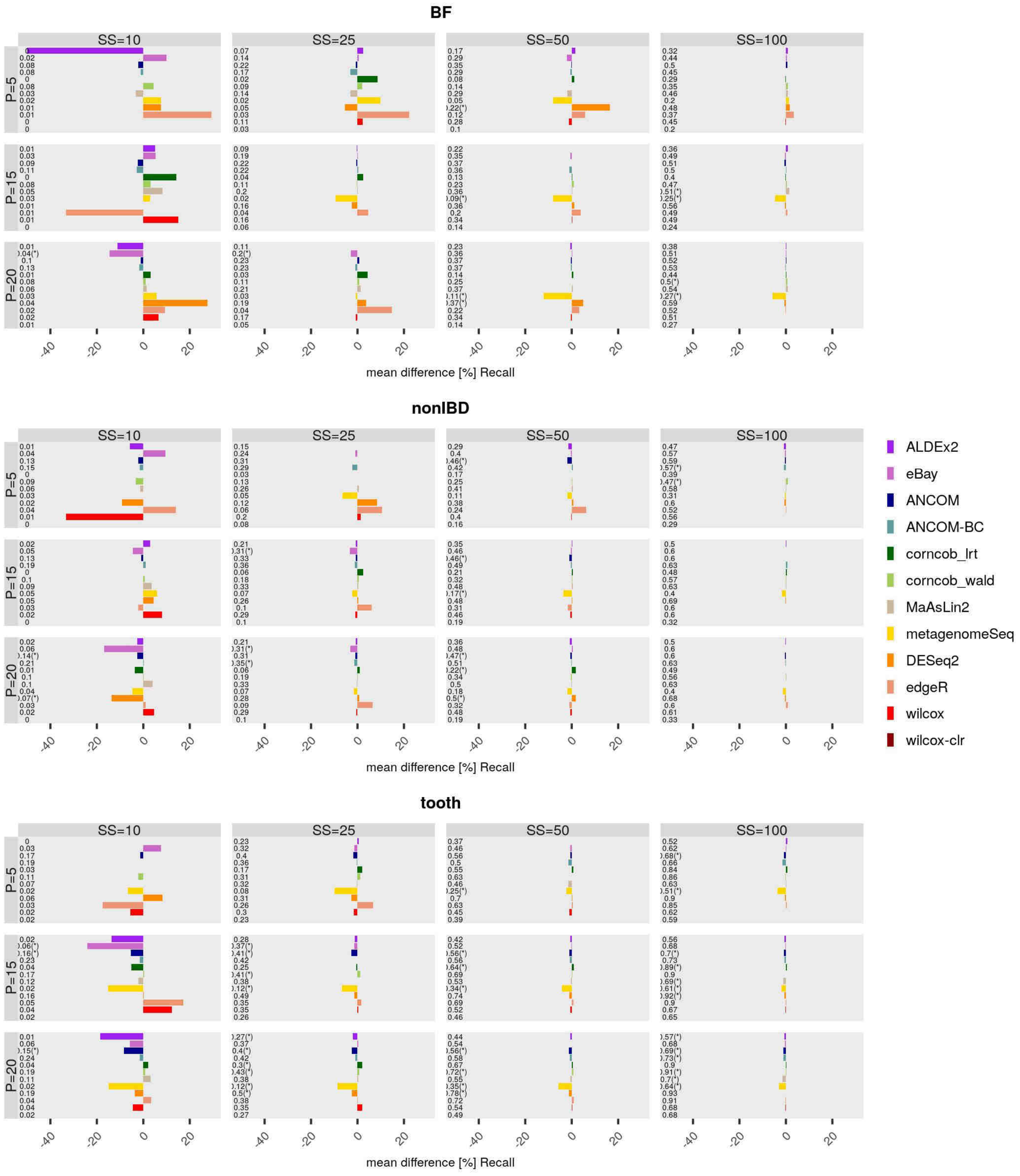

Figure 93. Mean Recall difference [%] between each differential abundance method and its GMPR normalised version for each dataset considered in the comparison in the scenario with simulated DA features. In each set of boxes corresponding to the dataset, different percentages (P) of simulated DA features are on rows, while different sample size (SS) values are on columns. Numbers at the beginning of each row correspond to the Recall values obtained with default normalization, while the symbol (*) identifies the Wilcoxon paired statistical test is significant.

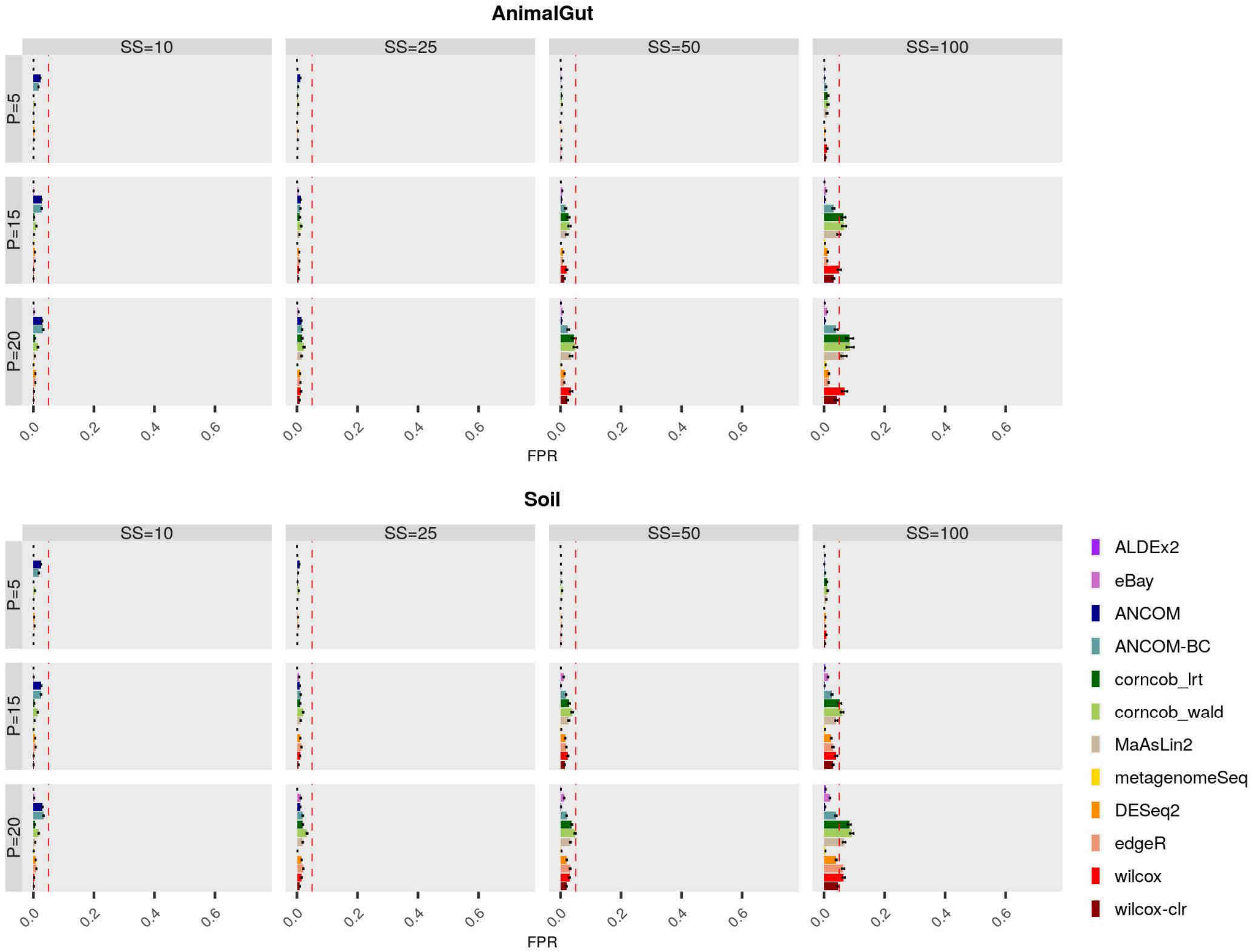

Figure 94. False Positive Rate (FPR) of each differential abundance method for AnimalGut and Soil dataset in the main scenario with simulated DA features. In each set of boxes corresponding to the dataset, tools are on rows, while different sample size (SS) values are on columns. The FPR values are averaged over the 50 simulations and the bars show the standard error.

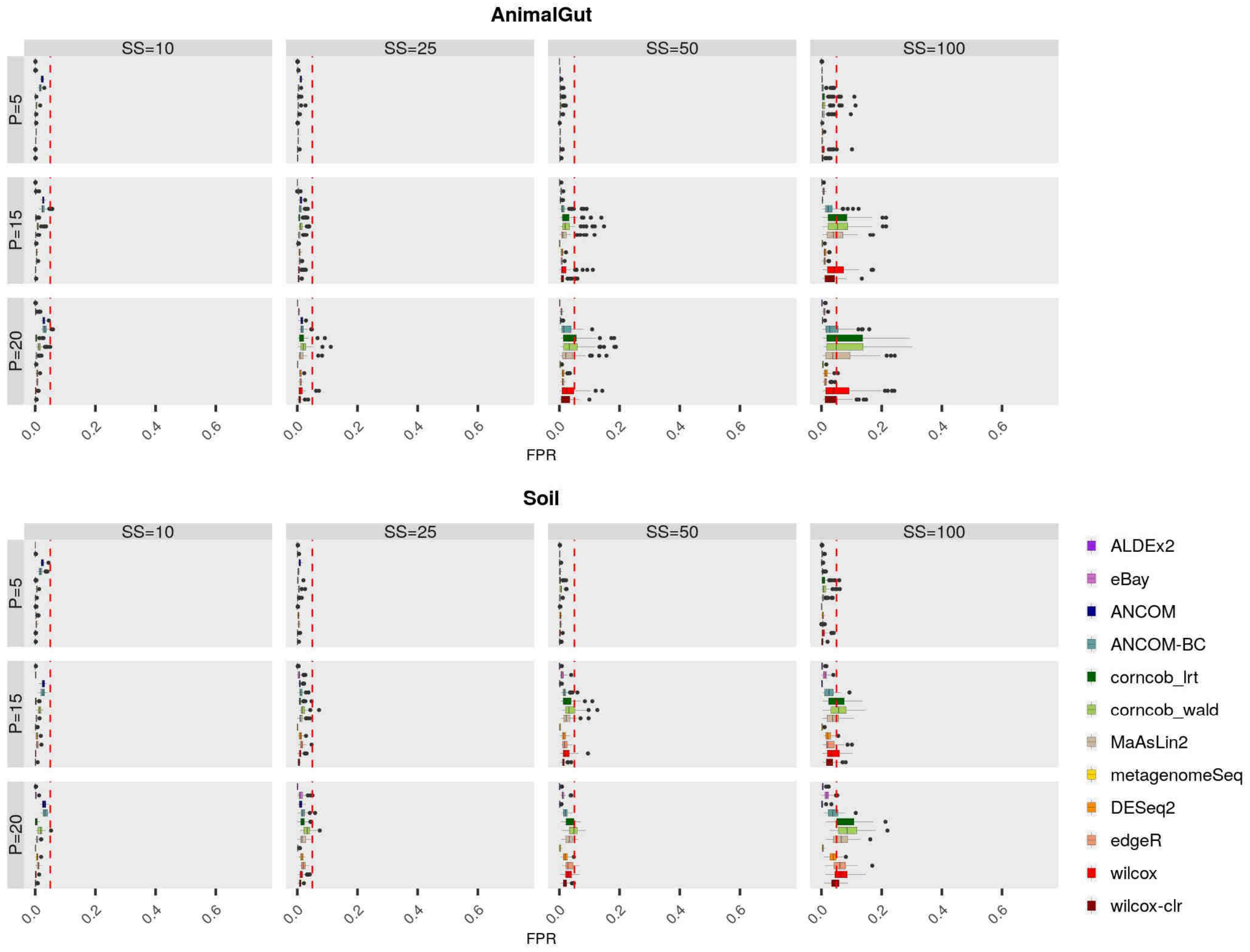

Figure 95. Boxplot of the False Positive Rate (FPR) distribution across the 50 simulations of each differential abundance method for AnimalGut and Soil dataset in the main scenario with simulated DA features. In each set of boxes corresponding to the dataset, tools are on rows, while different sample size (SS) values are on columns.

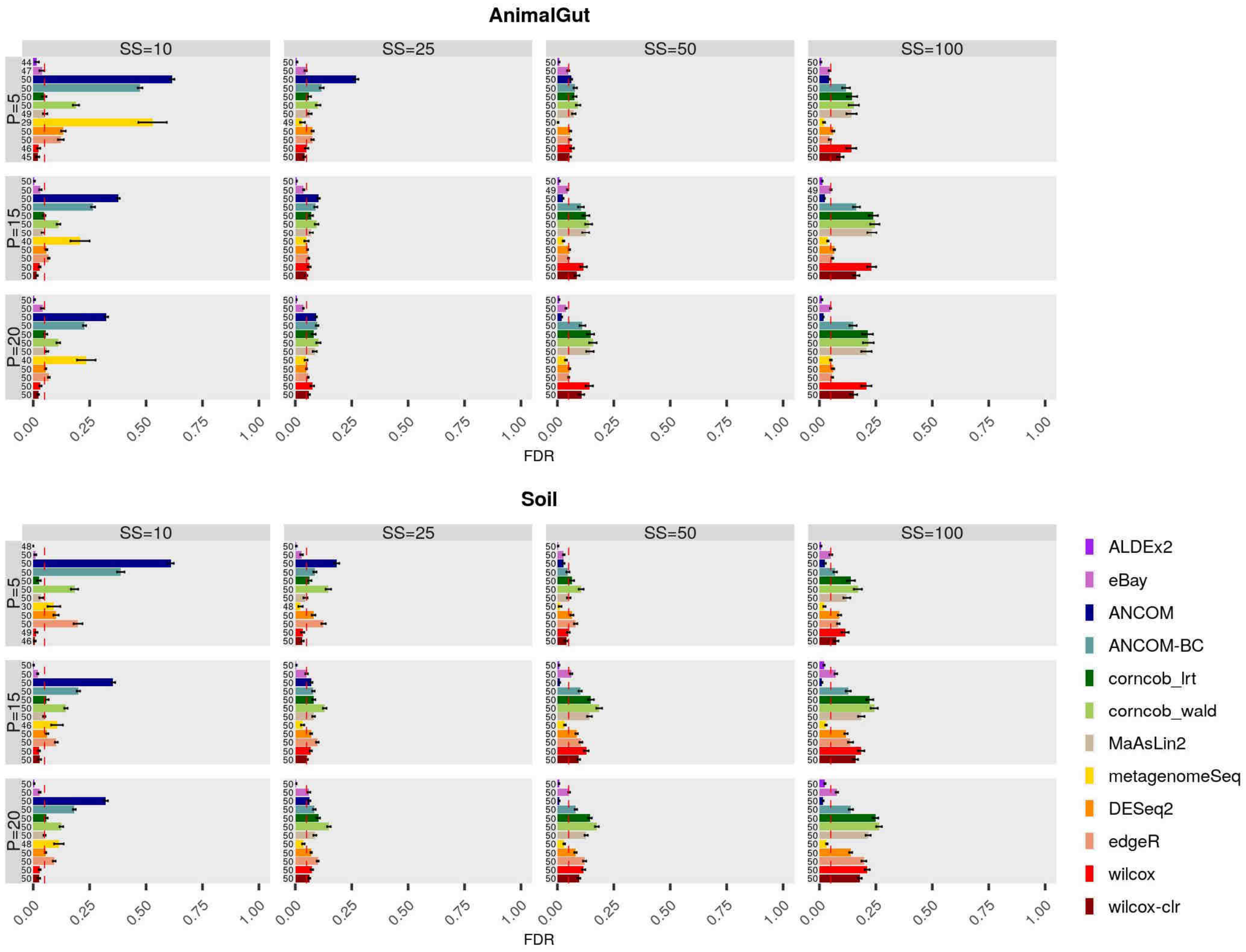

Figure 96. False Discovery Rate (FDR) of each differential abundance method for AnimalGut and Soil dataset in the main scenario with simulated DA features. In each set of boxes corresponding to the dataset, different percentages (P) of simulated DA features are on rows, while different sample size (SS) values are on columns. The FDR values are averaged over the 50 simulations and the bars show the standard error. The number of runs that provide a defined value of FDR is shown at the beginning of the bars.

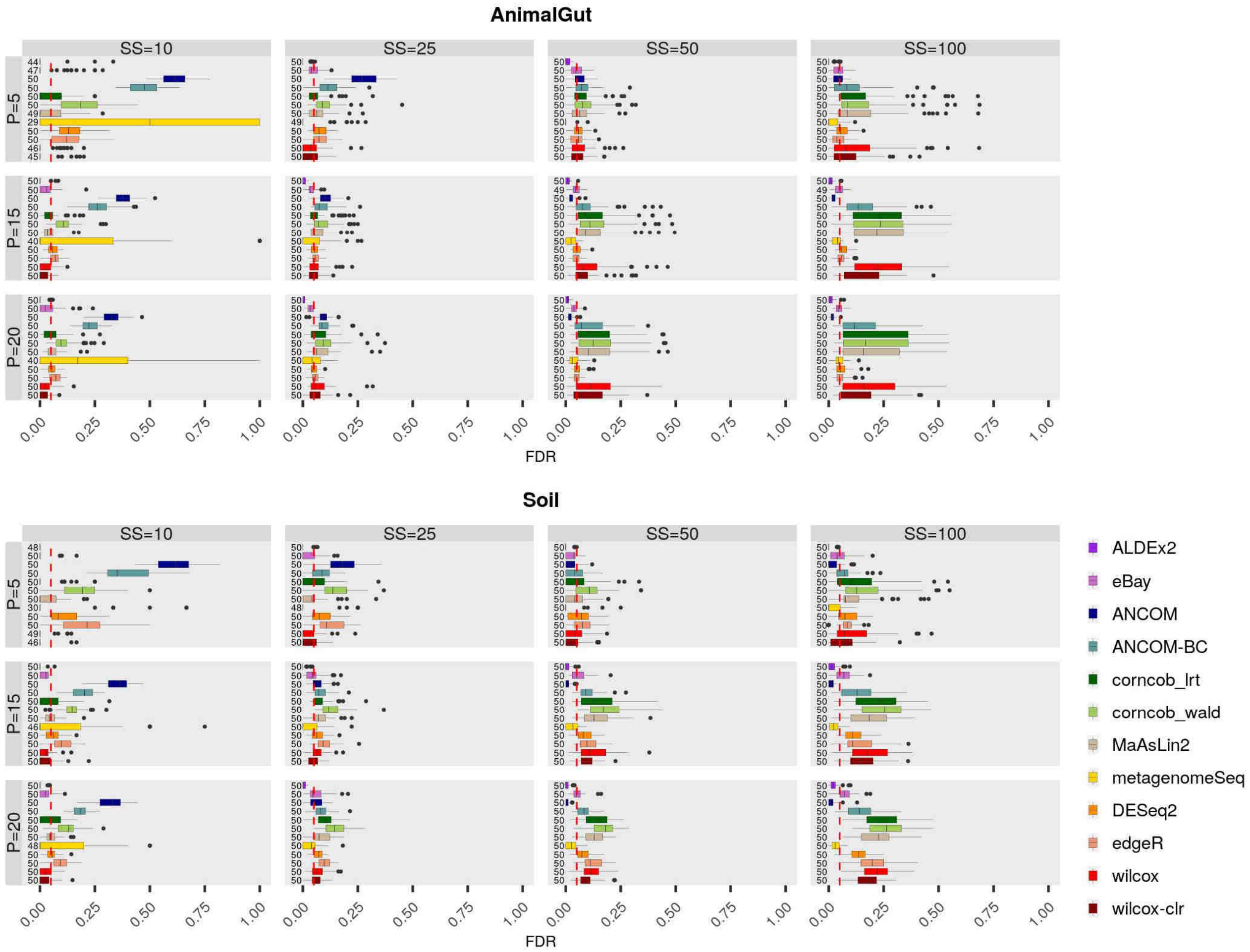

Figure 97. Boxplot of the False Discovery Rate (FDR) distribution across the 50 simulations of each differential abundance method for AnimalGut and Soil dataset in the main scenario with simulated DA features. In each set of boxes corresponding to the dataset, different percentages (P) of simulated DA features are on rows, while different sample size (SS) values are on columns. The number of runs that provide a defined value of FDR is shown on the left of each boxplot.

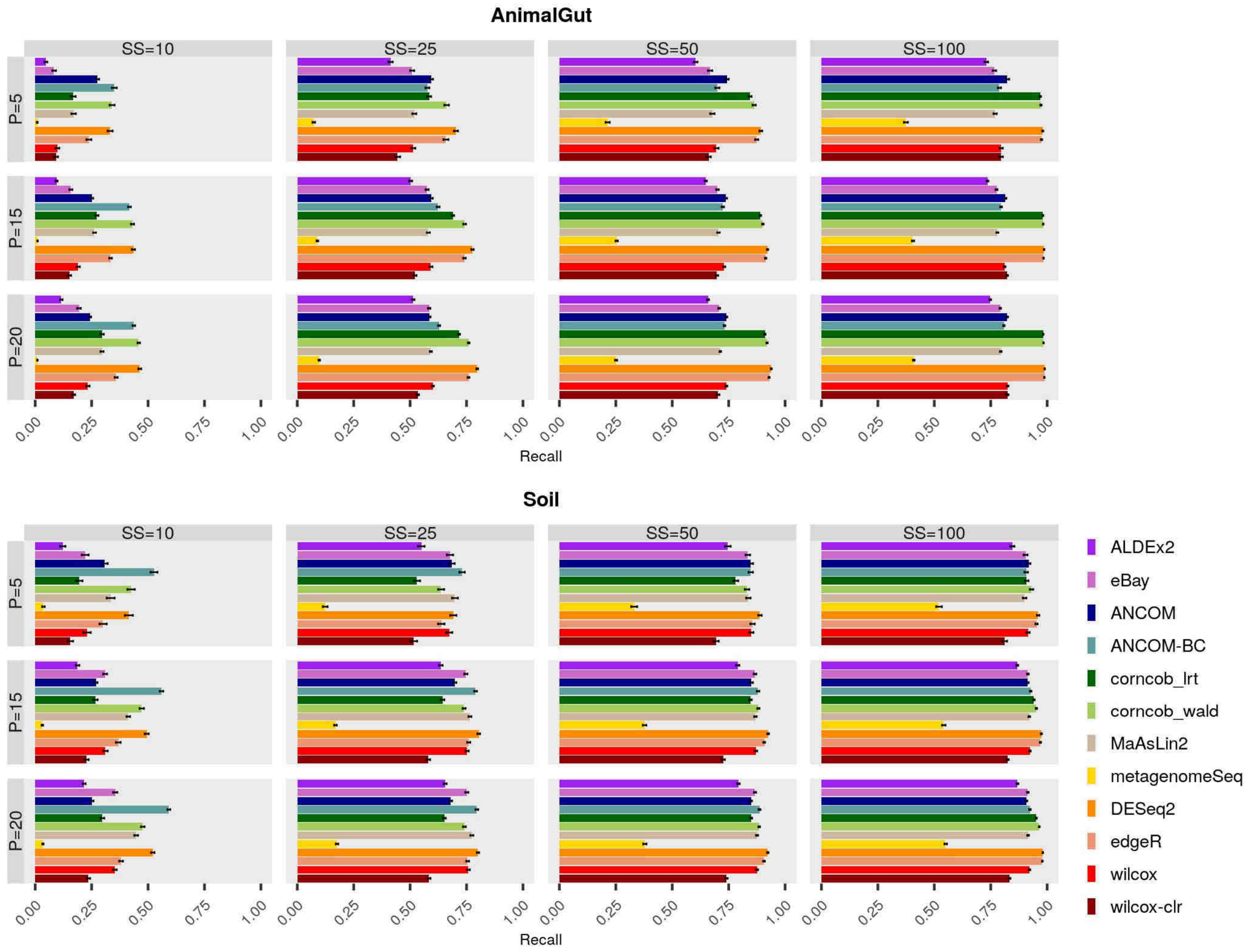

Figure 98. Recall of each differential abundance method for AnimalGut and Soil dataset in the main scenario with simulated DA features. In each set of boxes corresponding to the dataset, different percentages (P) of simulated DA features are on rows, while different sample size (SS) values are on columns. The recall values are averaged over the 50 simulations and the bars show the standard error.

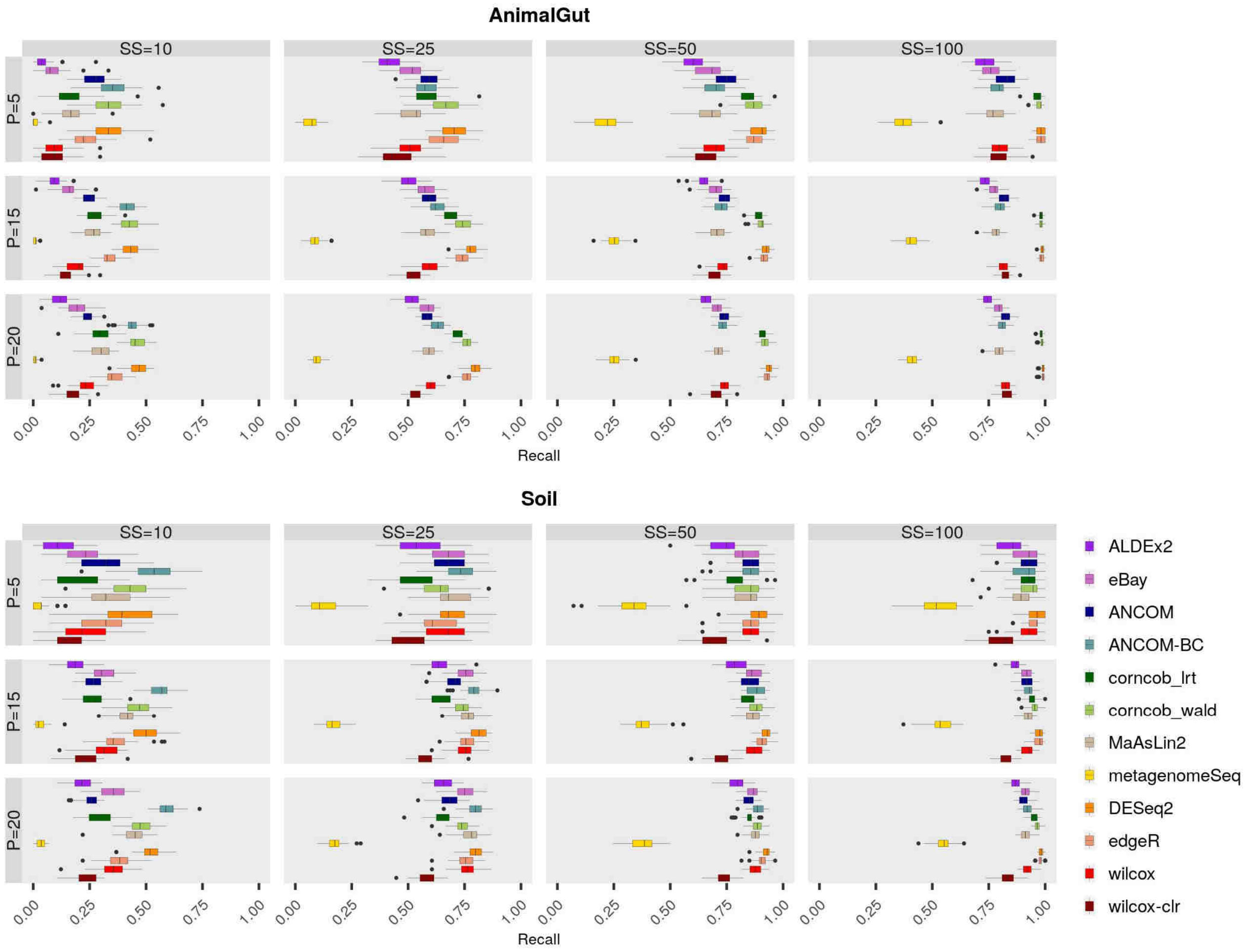

Figure 99. Boxplot of recall distribution across the 50 simulations of each differential abundance method for AnimalGut and Soil dataset in the main scenario with simulated DA features. In each set of boxes corresponding to the dataset, different percentages (P) of simulated DA features are on rows, while different sample size (SS) values are on columns.

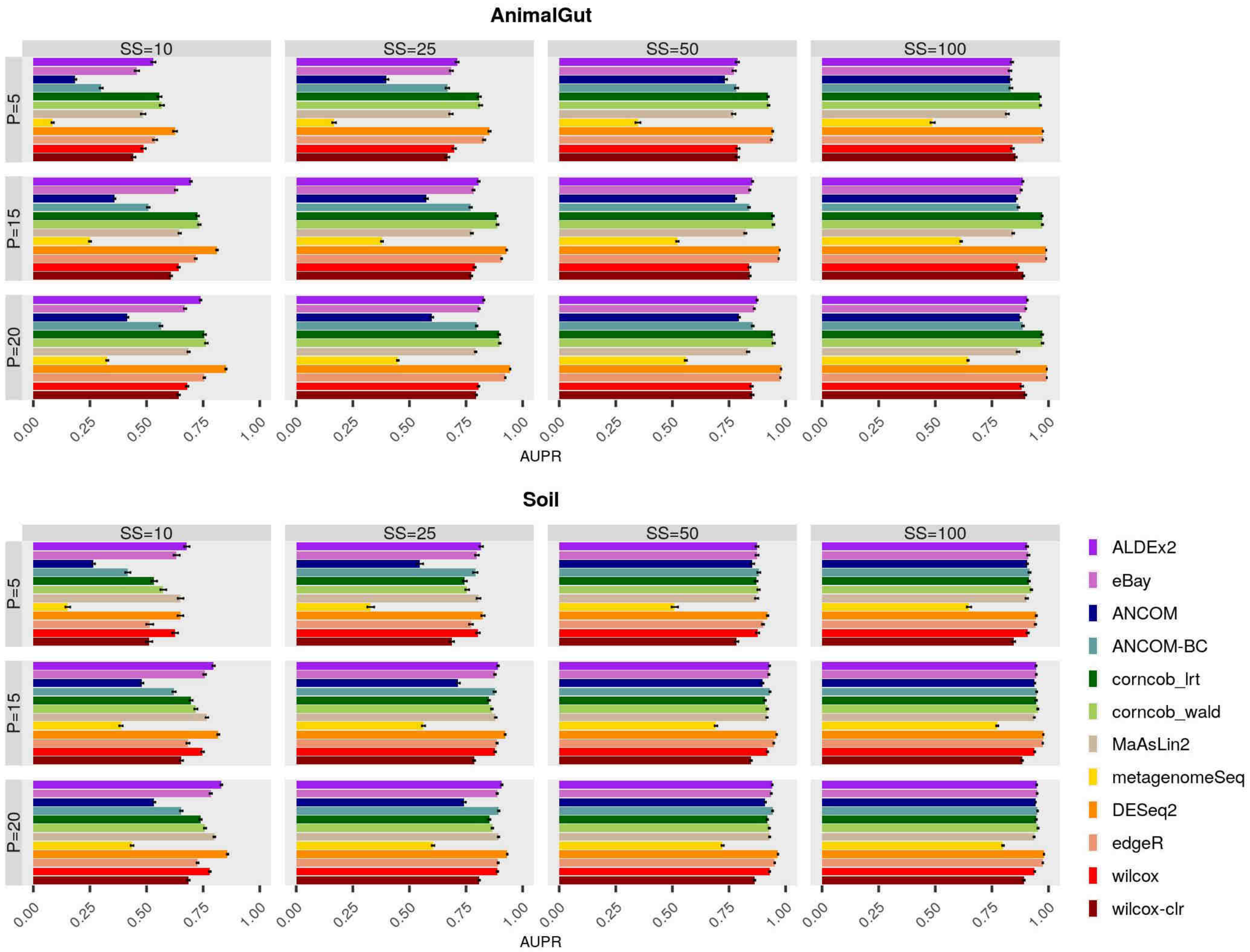

Figure 100. Area Under Precision-Recall curves (AUPR) of each differential abundance method for AnimalGut and Soil dataset in the main scenario with simulated DA features. In each set of boxes corresponding to the dataset, different percentages (P) of simulated DA features are on rows, while different sample size (SS) values are on columns. The AUPR values are averaged over the 50 simulations and the bars show the standard error.

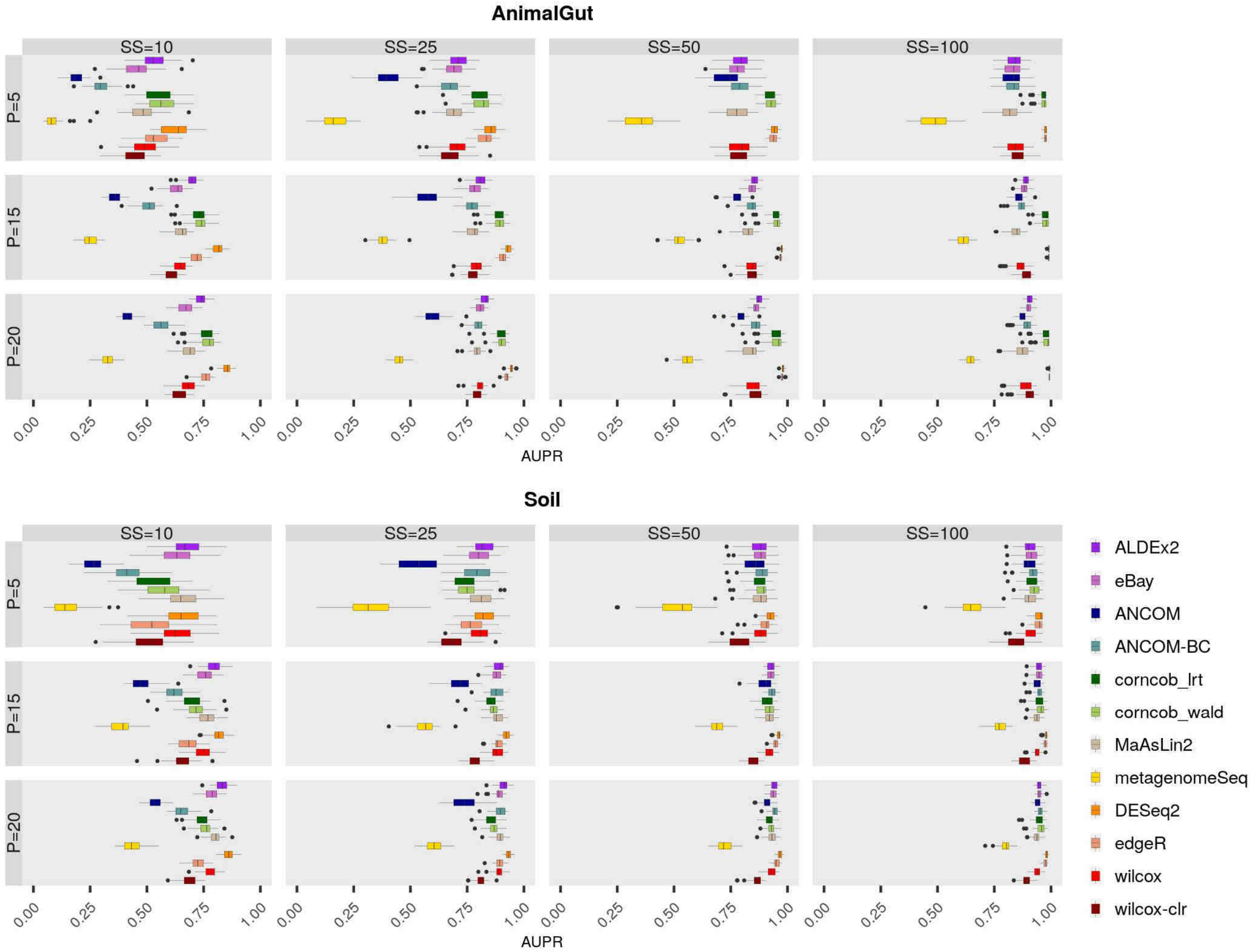

Figure 101. Boxplot of the Area Under Precision-Recall curves (AUPR) of each differential abundance method for AnimalGut and Soil dataset in the main scenario with simulated DA features. In each set of boxes corresponding to the dataset, different percentages (P) of simulated DA features are on rows, while different sample size (SS) values are on columns.

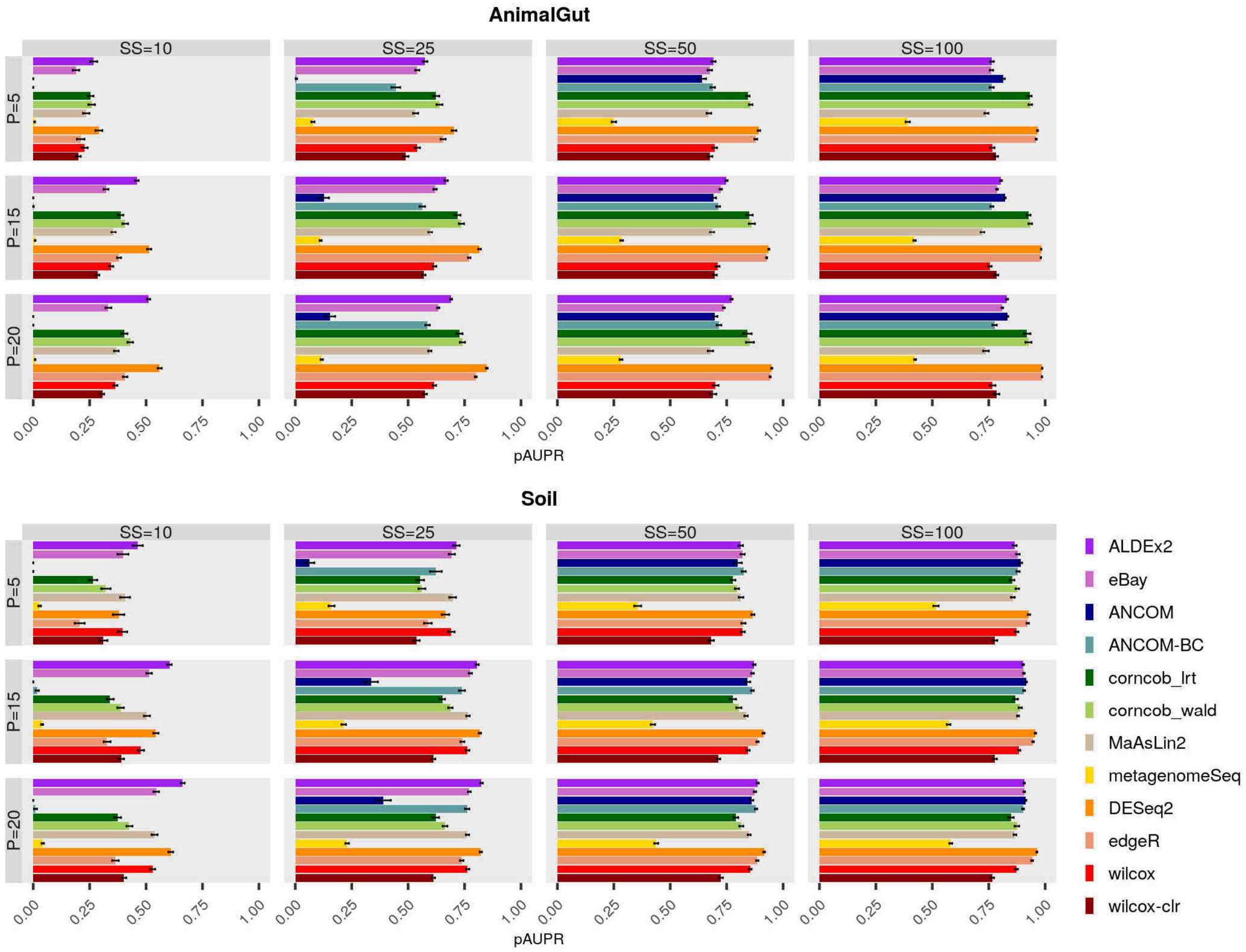

Figure 102. Partial Area Under Precision-Recall curves (pAUPR) of each differential abundance method for AnimalGut and Soil dataset in the main scenario with simulated DA features. In each set of boxes corresponding to the dataset, different percentages (P) of simulated DA features are on rows, while different sample size (SS) values are on columns. The AUPR values are averaged over the 50 simulations and the bars show the standard error.

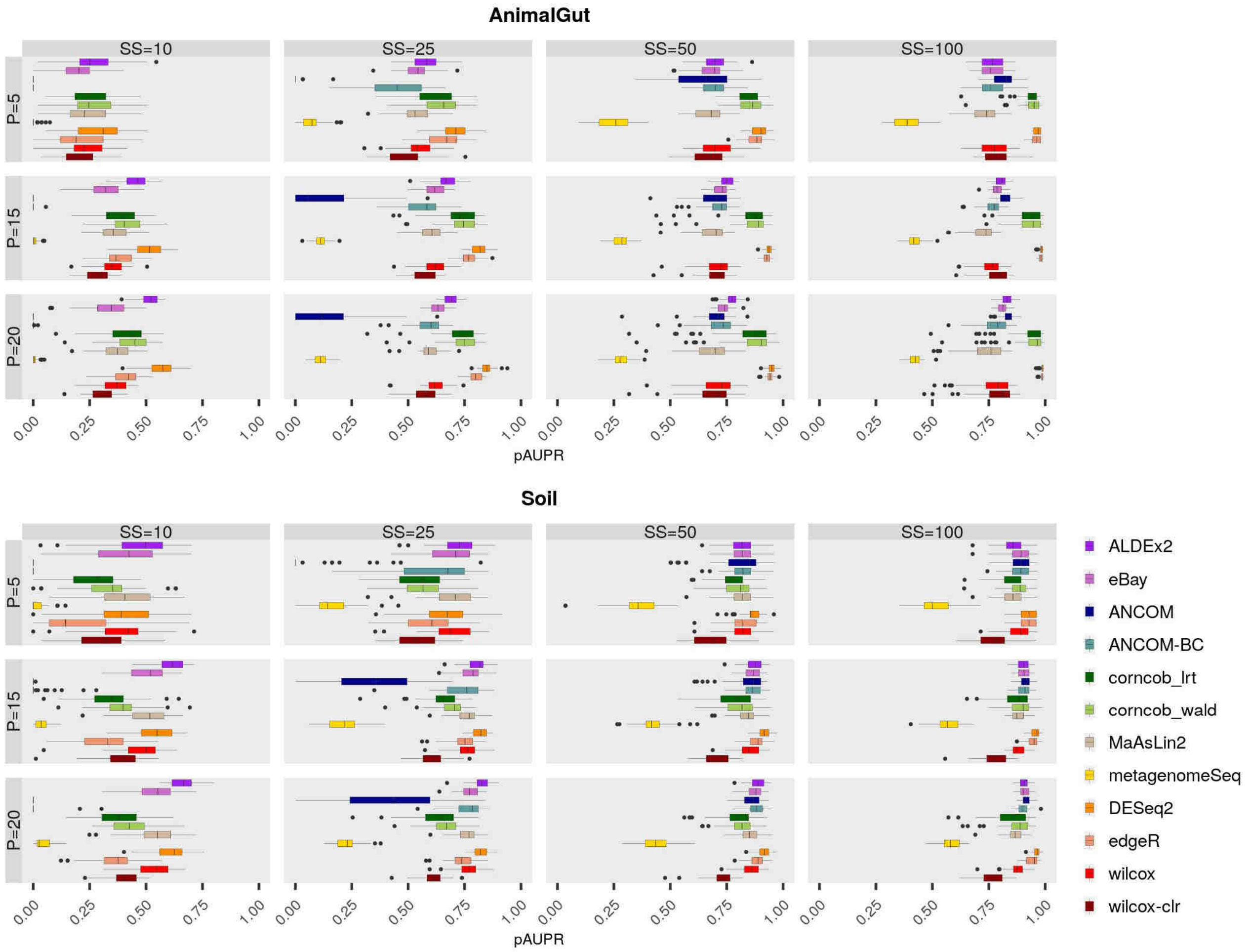

Figure 103. Boxplot of the partial Area Under Precision-Recall curves (pAUPR) of each differential abundance method for AnimalGut and Soil dataset in the main scenario with simulated DA features. In each set of boxes corresponding to the dataset, different percentages (P) of simulated DA features are on rows, while different sample size (SS) values are on columns.

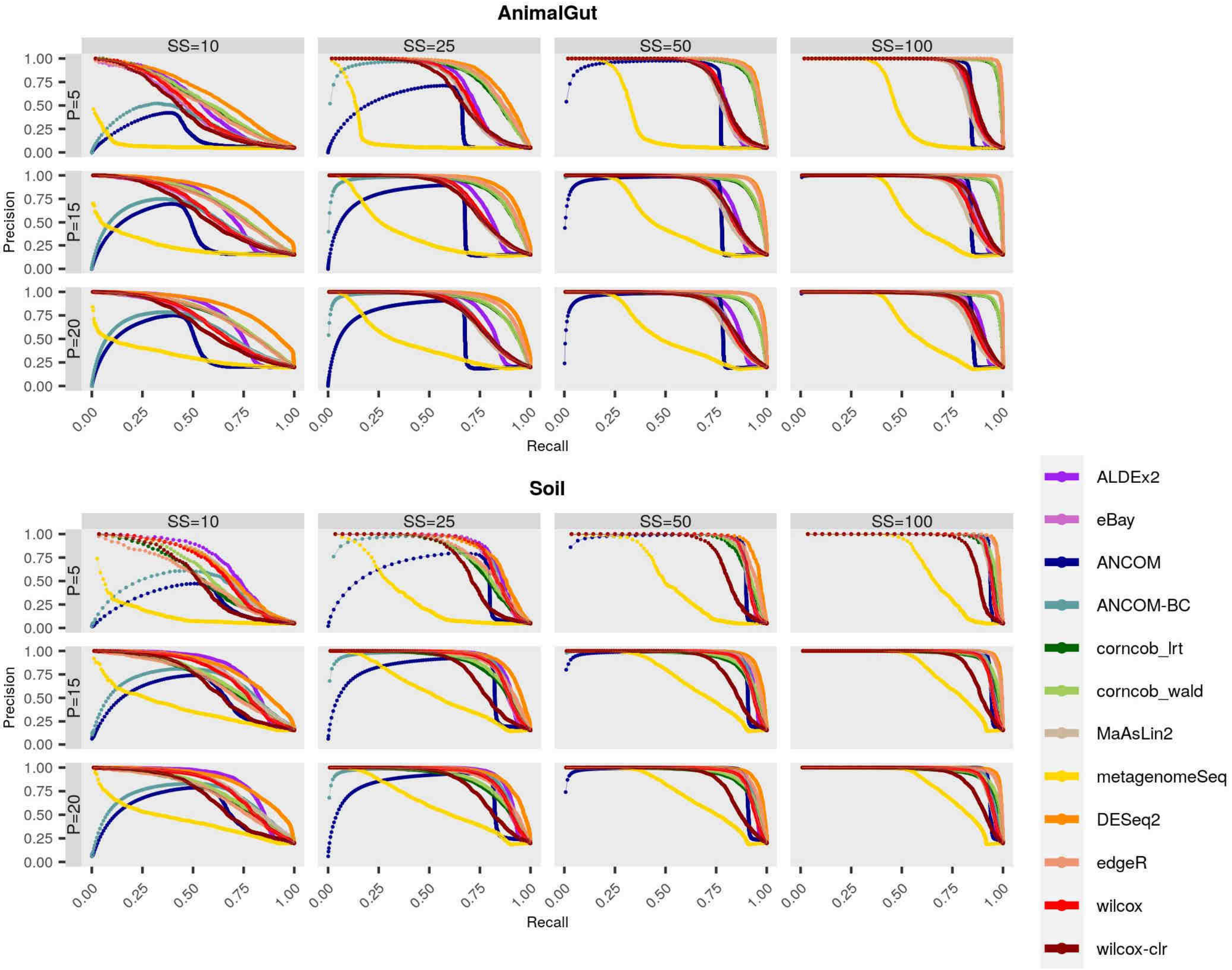

Figure 104. Mean Precision-Recall curves of each differential abundance method for AnimalGut and Soil dataset in the comparison in the main scenario with simulated DA features. In each set of boxes corresponding to the dataset, different percentages (P) of simulated DA features are on rows, while different sample size (SS) values are on columns. The mean pr-curve is defined as the average of the *i*-th precision value and the *i*-th recall value over the 50 simulations.

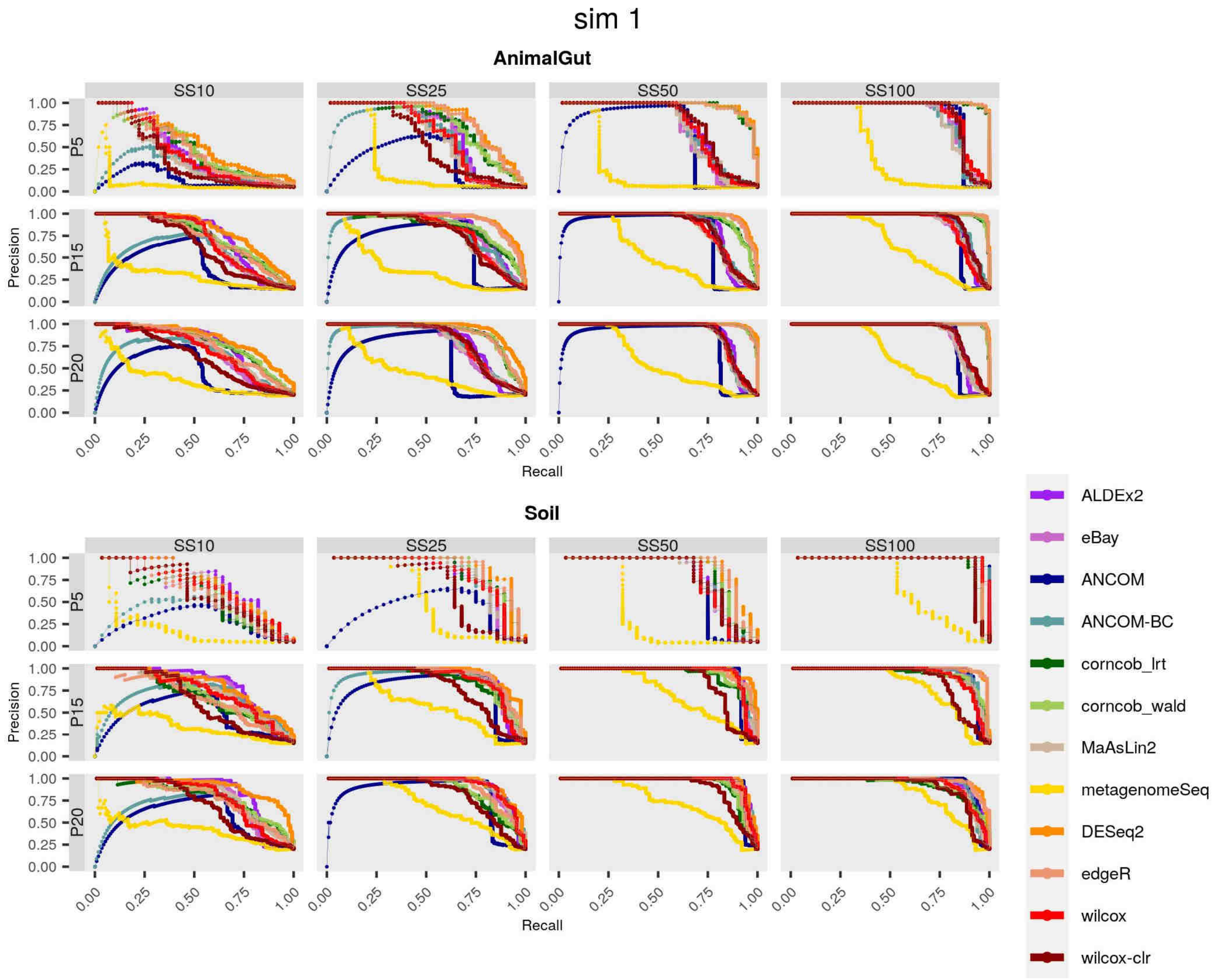

Figure 105. Simulation 1. Precision-Recall curves of each differential abundance method for AnimalGut and Soil dataset in the main scenario with simulated DA features. In each set of boxes corresponding to the dataset, different percentages (P) of simulated DA features are on rows, while different sample size (SS) values are on columns.

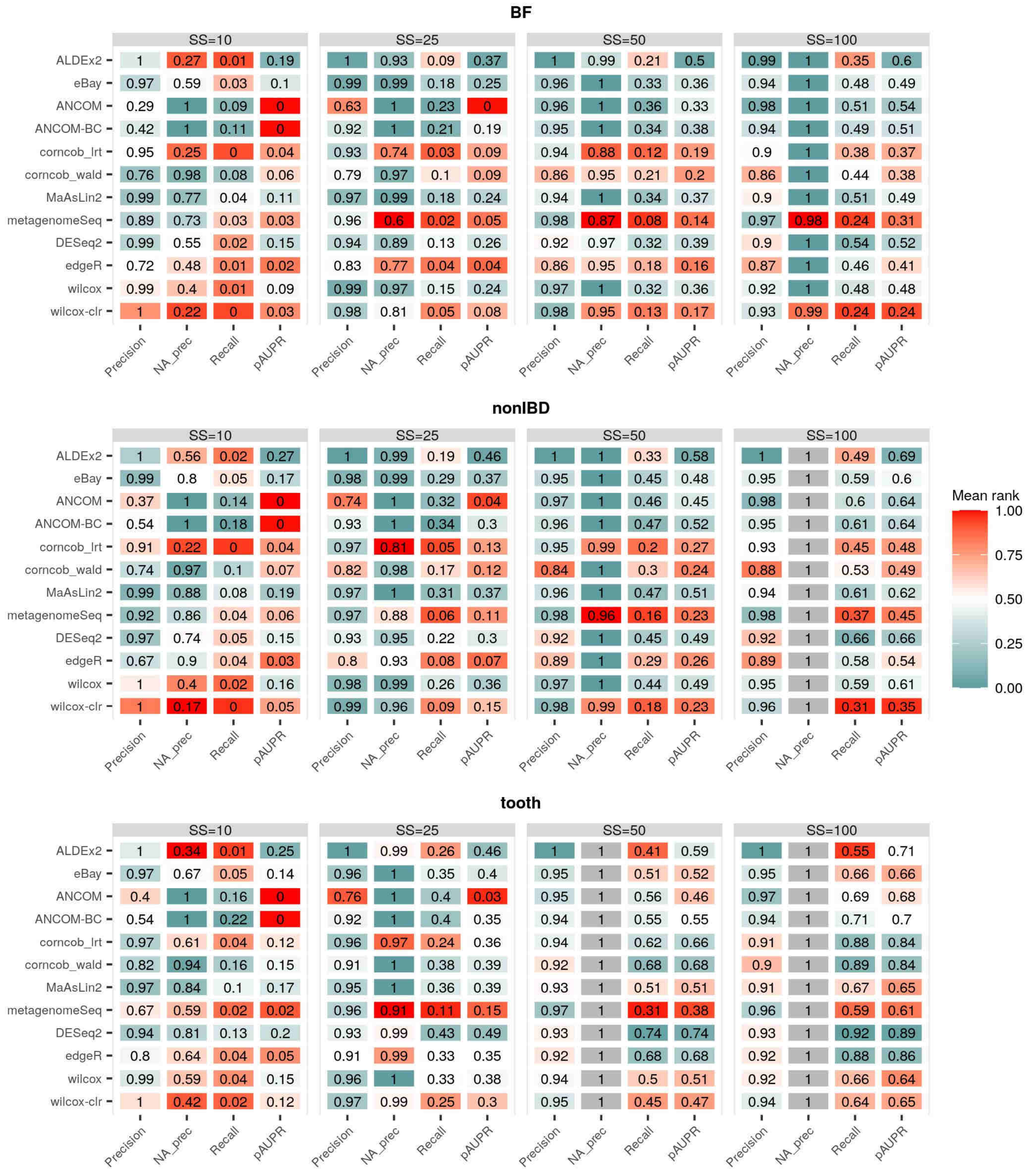

Figure 106. Average normalised ranks range from 0 to 1 (lower value means better performance) calculated on BF, nonIBD and tooth dataset for each performance metric on columns and on each method on rows. Different boxes correspond to different sample size scenario (SS). For each metric, Precision, NA_prec (percentage of simulations for which at least one taxon has been identified as DA), Recall, pAUPR, we report the mean values calculated over the 150 simulations in the tile (50 for P=5, 50 for P=15 and 50 for P=20). The rank is calculated for each simulation and then normalised to the range [0-1]. The average rank for each metric is used as a colour scale.

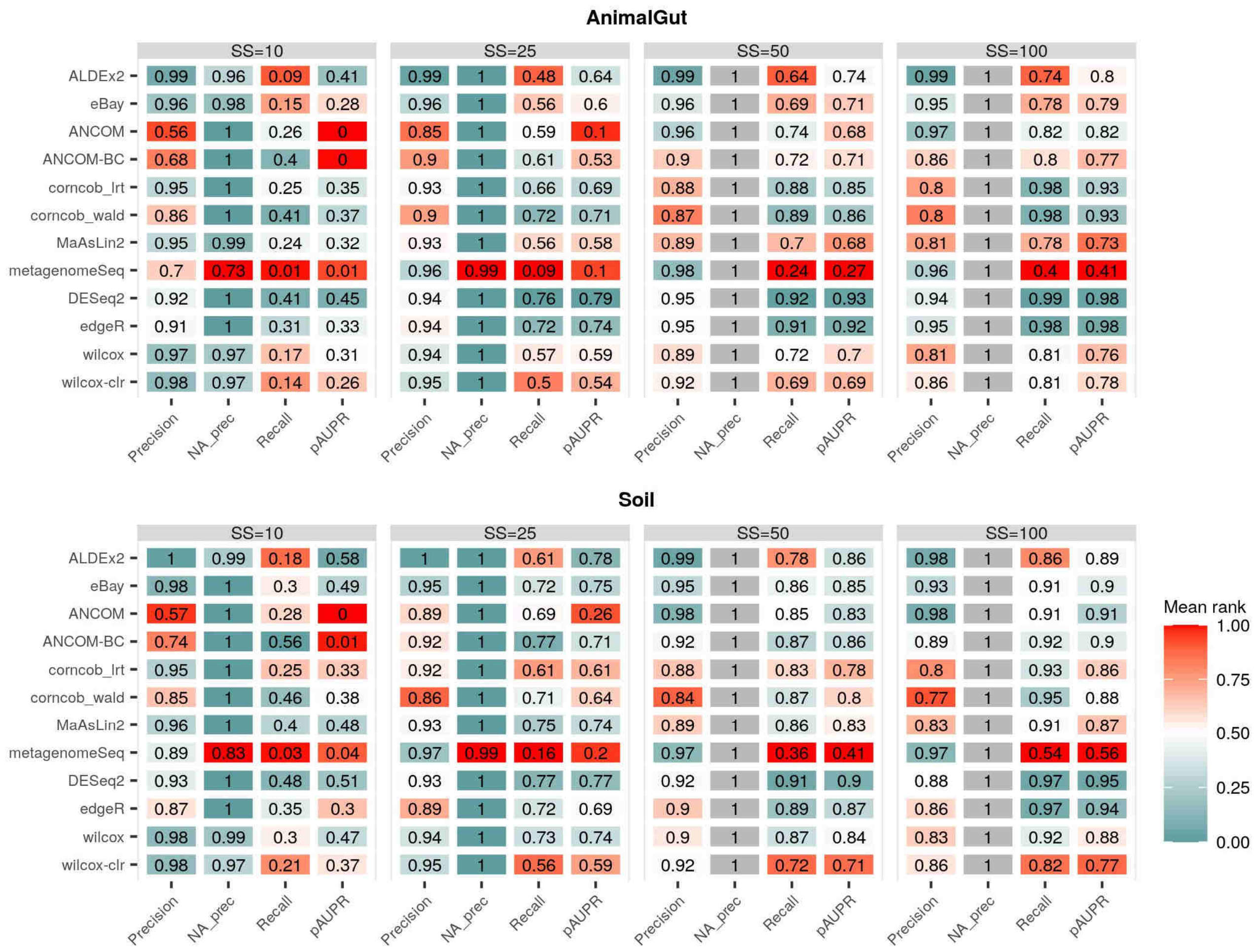

Figure 107. Average normalised ranks range from 0 to 1 (lower value means better performance) calculated on AnimalGut and Soil dataset for each performance metric on columns and on each method on rows. Different boxes correspond to different sample size scenario (SS). For each metric, Precision, NA_prec (percentage of simulations for which at least one taxon has been identified as DA), Recall, pAUPR, we report the mean values calculated over the 150 simulations in the tile (50 for P=5, 50 for P=15 and 50 for P=20). The rank is calculated for each simulation and then normalised to the range [0-1]. The average rank for each metric is used as a colour scale.

9 Comparison between ZIG and ZILG model in metagenomeSeq

In previously founding [26–28], the performance of metagenomeSeq is particularly unsatisfactory: i.e. high recall but also high FDR values. Since our simulations state the opposite, we carry out a more in-depth analysis. The authors of metagenomeSeq modified their originally procedure that exploited the zero-inflated Gaussian (ZIG) model and recommended using zero-inflated Log-Gaussian (ZILG) mixture model for DA analysis. Therefore, we run metagenomeSeq with both ZIG and ZILG model following the instruction in the Bioconductor vignette. We note that by default in the R package metagenomeSeq scales the normalisation factors (*nf*) as follows:

$$log_2\left(\frac{nf}{1000}+1\right) \tag{6}$$

Instead, in the vignette the authors scale the normalisation factors in a different way: $log_2\left(\frac{nf}{median(nf)}+1\right)$. We follow the example in the vignette, in analogy to what was done in Calgaro et al. [27]. Furthermore, the difference between the two models lies not only in the logarithmic scale used, but also in the normalisation factors scaling as shown in the following table.

Table D. Summary of difference between ZILG and ZIG implementation in metagenomeSeq R package.

Model	R function	Scale factor
ZILG	fitFeatureModel	log2(nf/median(nf)+1)
ZIG	fitzig	log(nf/median(nf))

We evaluate the differences in terms of FPR, FDR and Recall between the two models on the simulations in the scenario setting the θ threshold (see Figures 108-113).

We find that ZILG model shows good control of the FDR at the desired threshold, while the performance in terms of power decreases. This behaviour was also found in Lin et al. [28] which is the only one of the previously cited studies that compares both models.

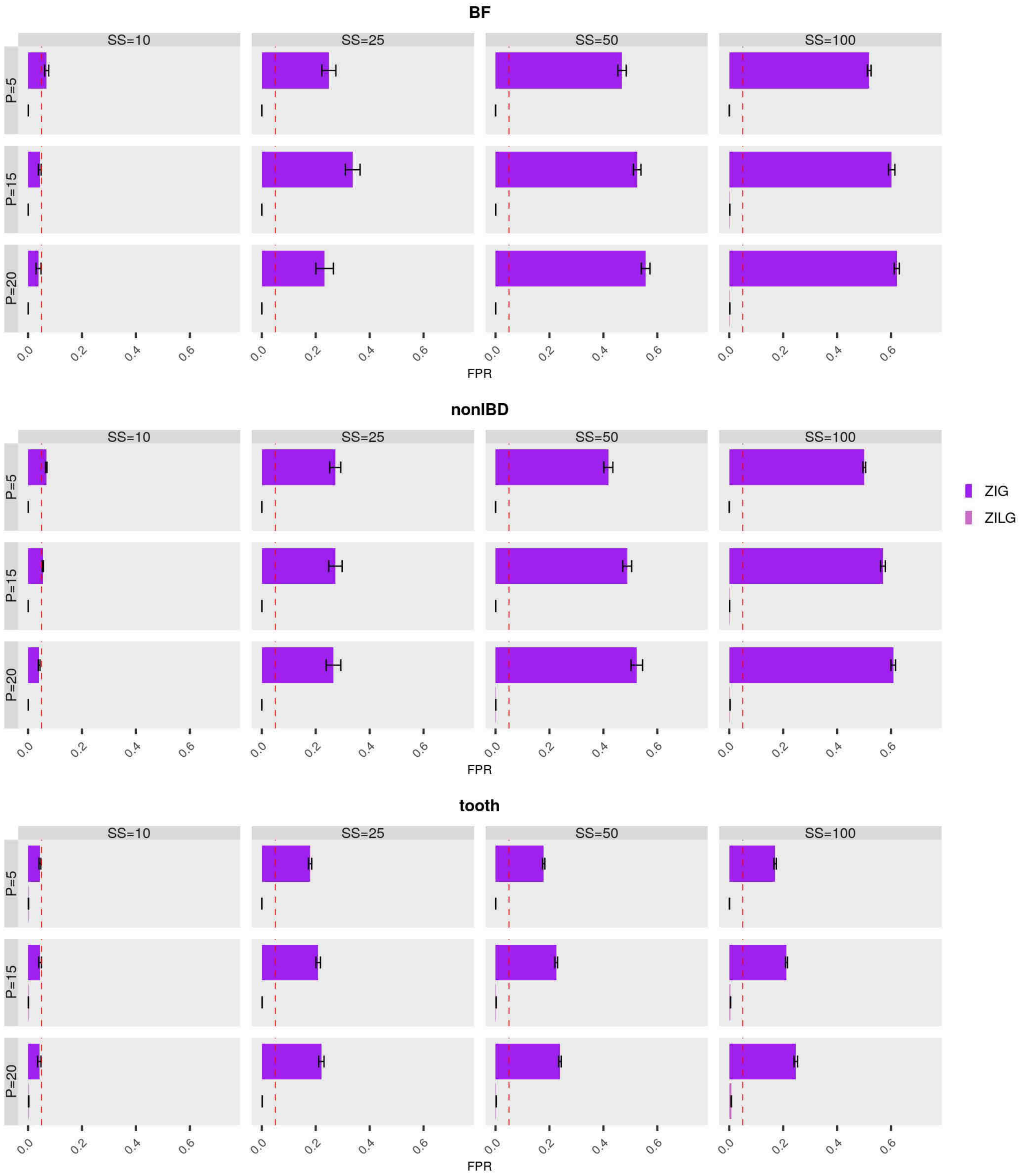

Figure 108. False Positive Rate (FPR) of ZIG and ZILG model in metagenomeSeq for BF, nonIBD and tooth dataset in the main scenario with simulated DA features. In each set of boxes corresponding to the dataset, tools are on rows, while different sample size (SS) values are on columns. The FPR values are averaged over the 50 simulations and the bars show the standard error.

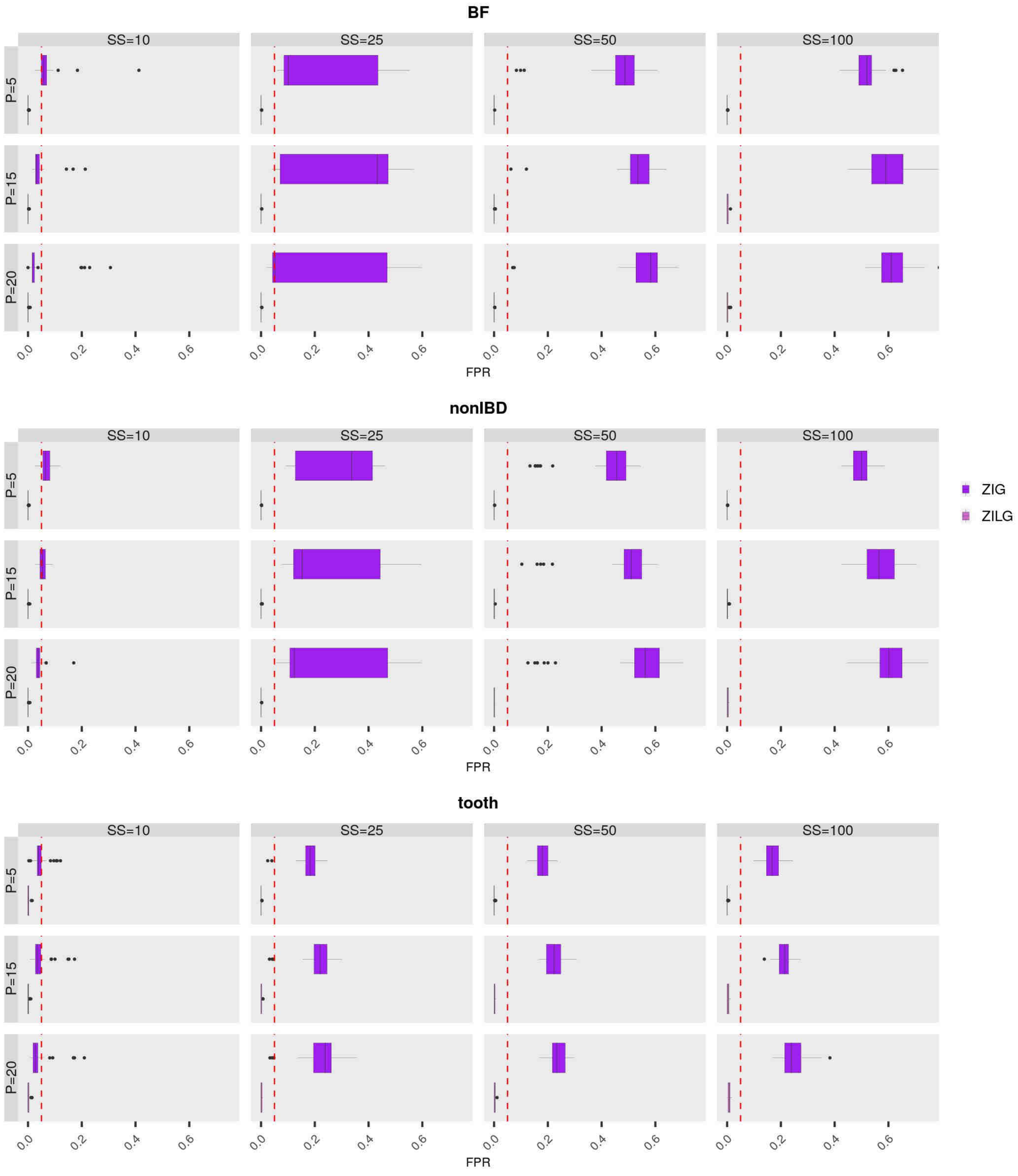

Figure 109. Boxplot of the False Positive Rate (FPR) distribution across the 50 simulations of ZIG and ZILG model in metagenomeSeq for BF, nonIBD and tooth dataset in the main scenario with simulated DA features. In each set of boxes corresponding to the dataset, tools are on rows, while different sample size (SS) values are on columns.

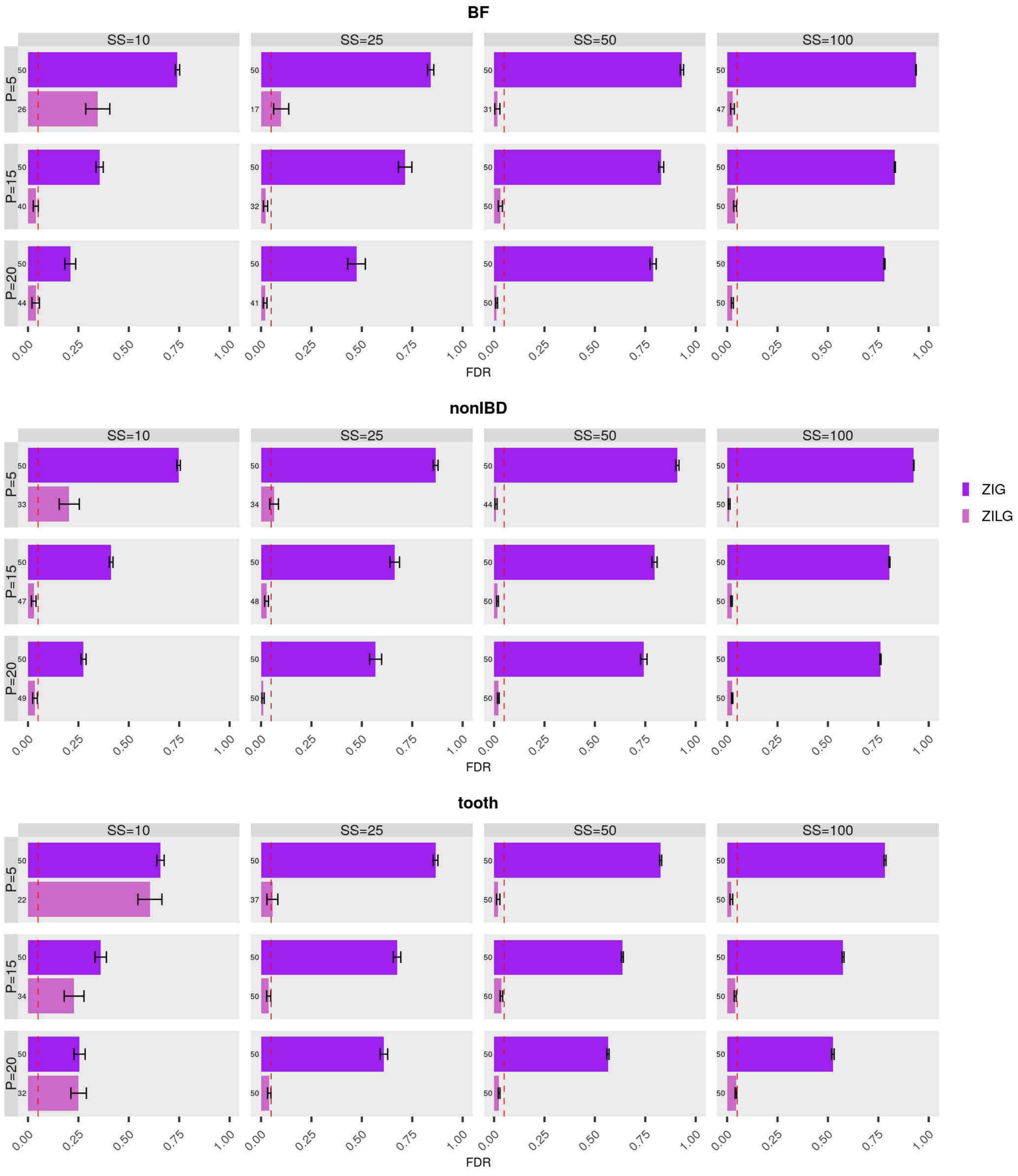

Figure 110. False Discovery Rate (FDR) of ZIG and ZILG model in metagenomeSeq for BF, nonIBD and tooth dataset in the main scenario with simulated DA features. In each set of boxes corresponding to the dataset, different percentages (P) of simulated DA features are on rows, while different sample size (SS) values are on columns. The FDR values are averaged over the 50 simulations and the bars show the standard error. The number of runs that provide a defined value of FDR is shown at the beginning of the bars.

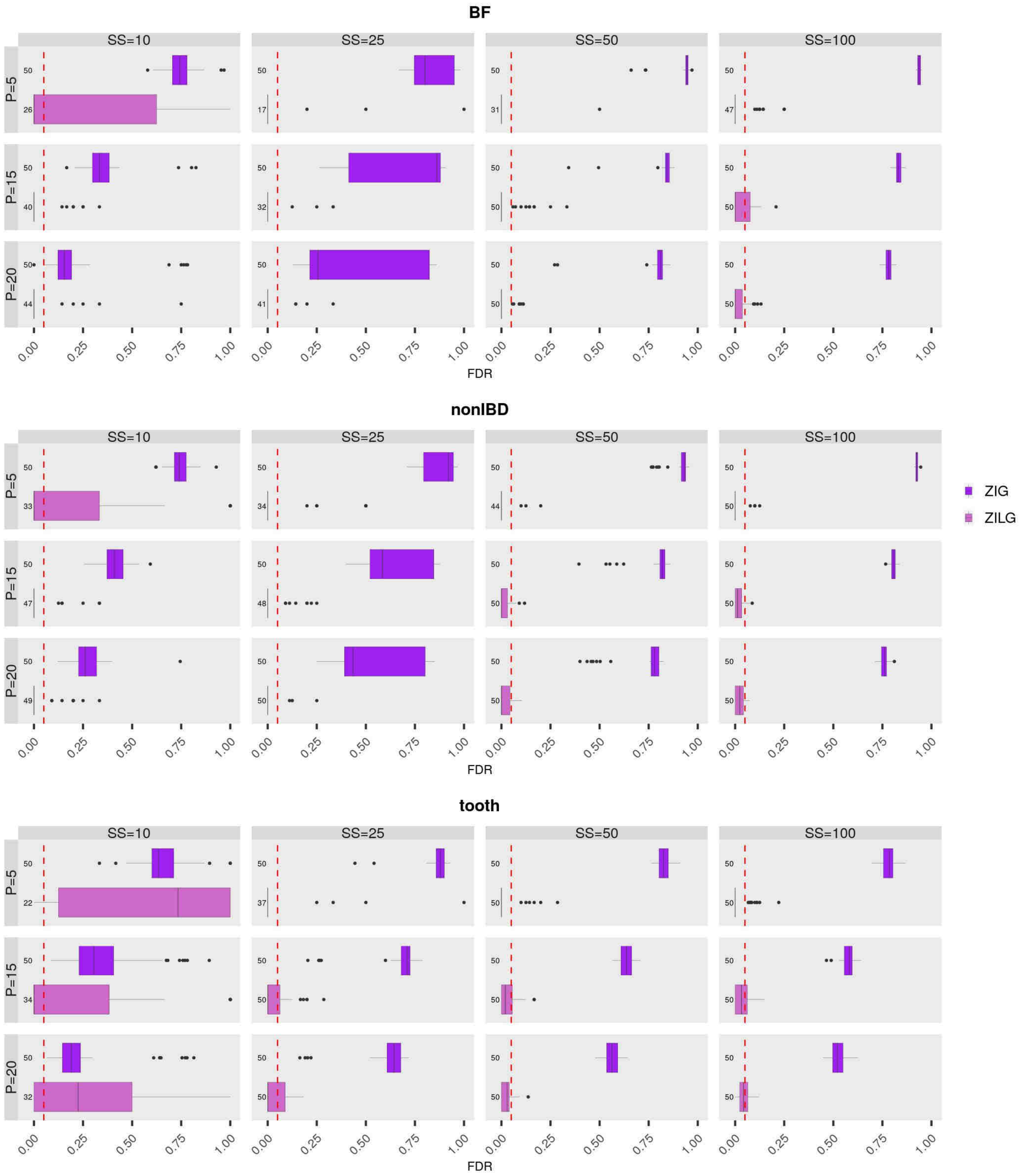

Figure 111. Boxplot of the False Discovery Rate (FDR) distribution across the 50 simulations of ZIG and ZILG model in metagenomeSeq for BF, nonIBD and tooth dataset in the main scenario with simulated DA features. In each set of boxes corresponding to the dataset, different percentages (P) of simulated DA features are on rows, while different sample size (SS) values are on columns. The number of runs that provide a defined value of FDR is shown on the left of each boxplot.

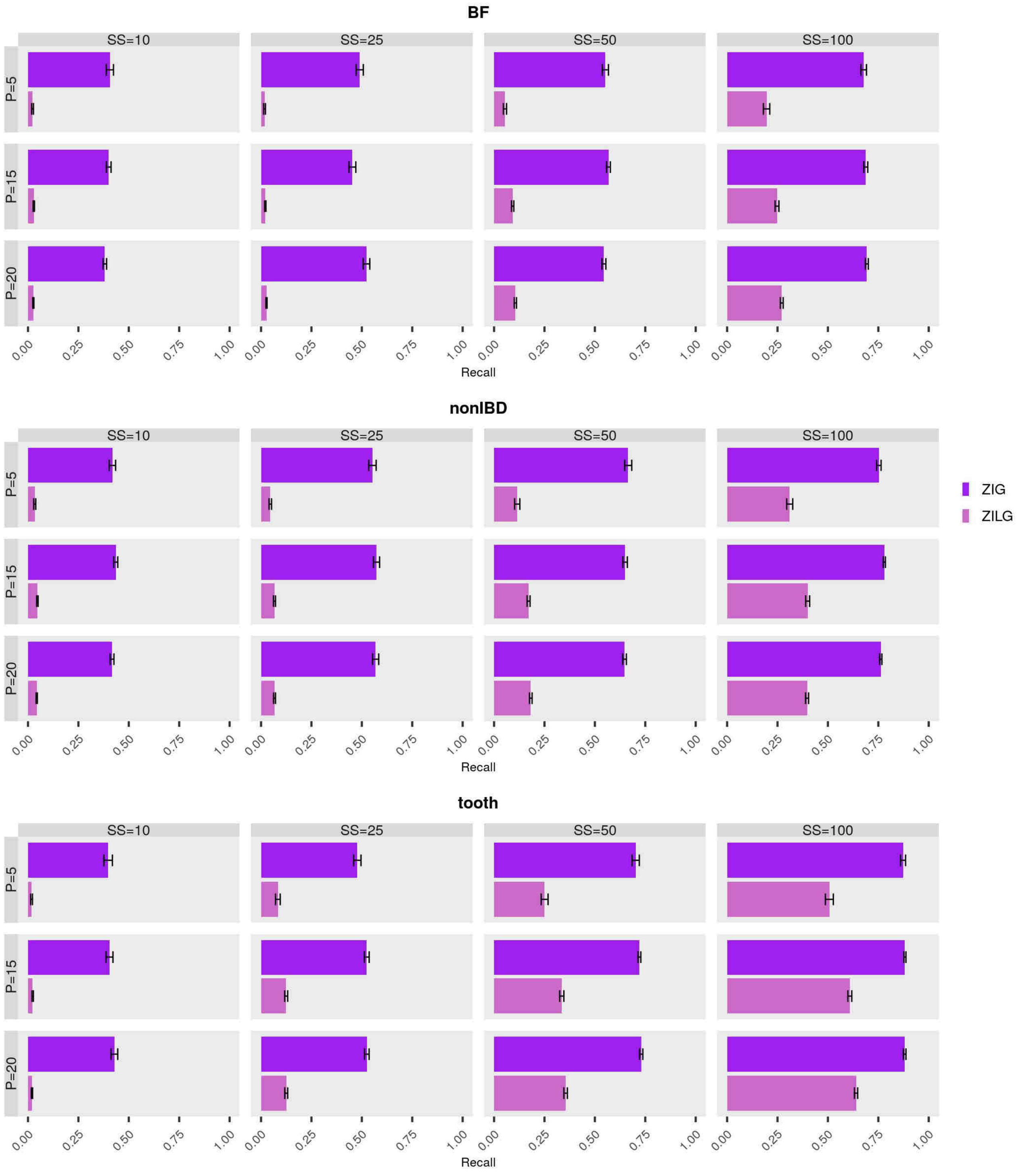

Figure 112. Recall of ZIG and ZILG model in metagenomeSeq for BF, nonIBD and tooth dataset in the main scenario with simulated DA features. In each set of boxes corresponding to the dataset, different percentages (P) of simulated DA features are on rows, while different sample size (SS) values are on columns. The recall values are averaged over the 50 simulations and the bars show the standard error.

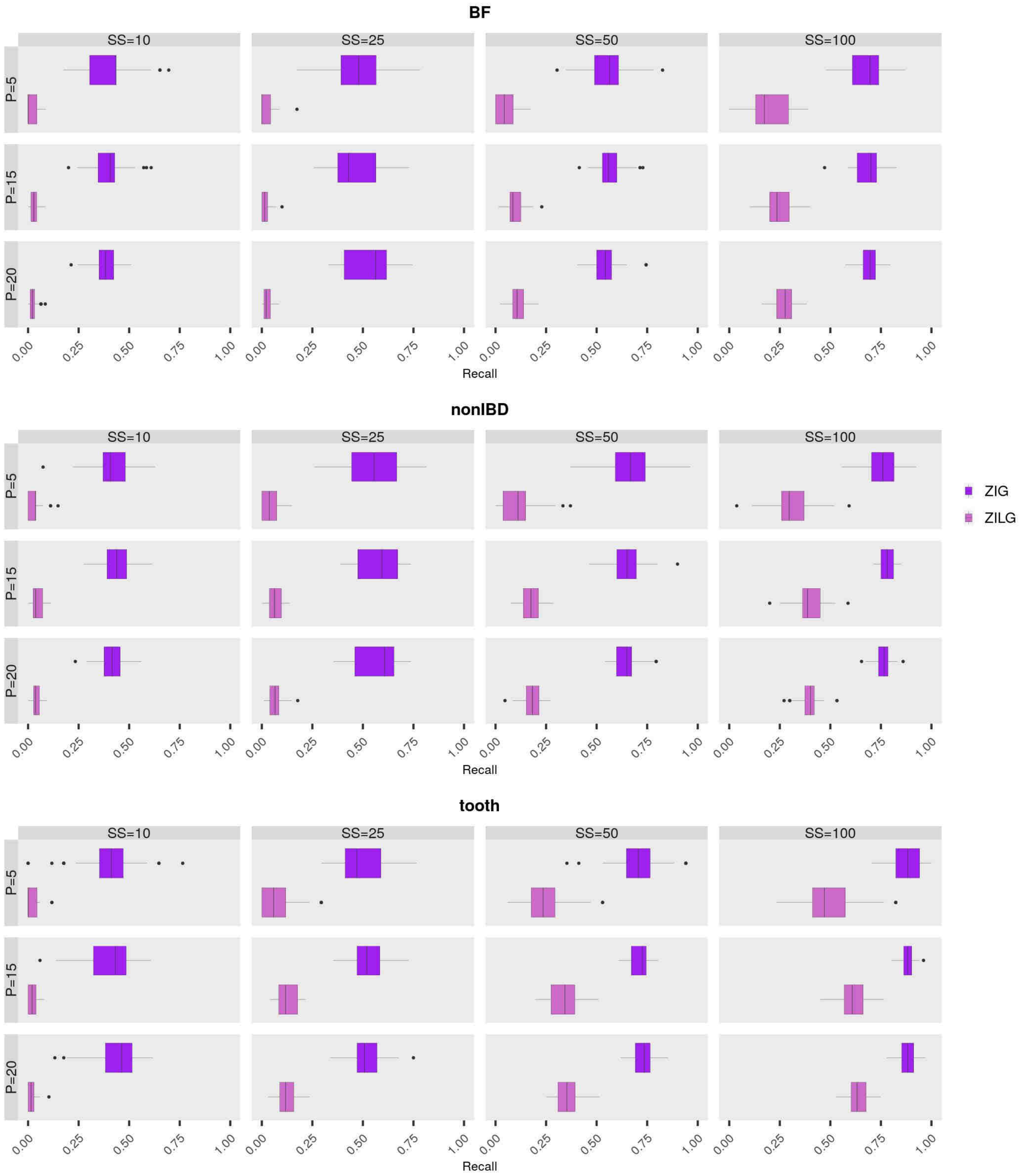

Figure 113. Boxplot of ZIG and ZILG model in metagenomeSeq for BF, nonIBD and tooth dataset in the main scenario with simulated DA features. In each set of boxes corresponding to the dataset, different percentages (P) of simulated DA features are on rows, while different sample size (SS) values are on columns.

10 References

1. Fernandes AD, Reid JNS, Macklaim JM, McMurrough TA, Edgell DR, Gloor GB. Unifying the analysis of high-throughput sequencing datasets: characterizing RNA-seq, 16S rRNA gene sequencing and selective growth experiments by compositional data analysis. Microbiome. 2014 May;2(1):15.

2. Aitchison J. The Statistical Analysis of Compositional Data. J R Stat Soc Ser B. 1982;44(2):139–77.

3. Liu T, Zhao H, Wang T. An empirical Bayes approach to normalization and differential abundance testing for microbiome data. BMC Bioinformatics. 2020 Jun;21(1):255.

4. Martin BD, Witten D, Willis AD. MODELING MICROBIAL ABUNDANCES AND DYSBIOSIS WITH BETA-BINOMIAL REGRESSION. Ann Appl Stat. 2020 Mar;14(1):94–115.

5. Fletcher R. Practical methods of optimization. 2000;450.

6. Sequential Quadratic Programming. Numer Optim. 1999 Jun 6;526–73.

7. Mallick H, Rahnavard A, McIver LJ, Ma S, Zhang Y, Nguyen LH, et al. Multivariable association discovery in population-scale meta-omics studies. PLOS Comput Biol. 2021 Nov 1;17(11):e1009442.

8. Paulson JN, Stine OC, Bravo HC, Pop M. Differential abundance analysis for microbial marker-gene surveys. Nat Methods 2013 1012. 2013 Sep;10(12):1200–2.

9. Gentleman R, Carey VJ, Huber W, Irizarry RA, Dudoit S, editors. Bioinformatics and Computational Biology Solutions Using R and Bioconductor. 2005;

10. Mandal S, Van Treuren W, White RA, Eggesbø M, Knight R, Peddada SD. Analysis of composition of microbiomes: a novel method for studying microbial composition. Microb Ecol Heal Dis. 2015 May;26:27663.

11. Kaul A, Mandal S, Davidov O, Peddada SD. Analysis of microbiome data in the presence of excess zeros. Front Microbiol. 2017 Nov 7;8(NOV):2114.

12. Lin H, Peddada S Das. Analysis of compositions of microbiomes with bias correction. Nat Commun.

2020;11(1):3514.

13. Robinson MD, McCarthy DJ, Smyth GK. edgeR: a Bioconductor package for differential expression analysis of digital gene expression data. Bioinformatics. 2010 Jan;26(1):139–40.

14. Love MI, Huber W, Anders S. Moderated estimation of fold change and dispersion for RNA-seq data with DESeq2. Genome Biol. 2014 Dec;15(12):550.

15. Robinson MD, Smyth GK. Moderated statistical tests for assessing differences in tag abundance. Bioinformatics. 2007 Nov;23(21):2881–7.

16. Risso D, Schwartz K, Sherlock G, Dudoit S. GC-Content Normalization for RNA-Seq Data. BMC Bioinformatics. 2011 Dec 17;12(1):1–17.

17. Hansen KD, Irizarry RA, Wu Z. Removing technical variability in RNA-seq data using conditional quantile normalization. Biostatistics. 2012 Apr;13(2):204–16.

18. van den Boogaart KG, Tolosana-Delgado R. “compositions”: A unified R package to analyze compositional data. Comput Geosci. 2008 Apr 1;34(4):320–38.

19. Patuzzi I, Baruzzo G, Losasso C, Ricci A, Di Camillo B. metaSPARSim: a 16S rRNA gene sequencing count data simulator. BMC Bioinformatics. 2019 Nov;20(Suppl 9):416.

20. Huttenhower C, Gevers D, Knight R, Abubucker S, Badger JH, Chinwalla AT, et al. Structure, function and diversity of the healthy human microbiome. Nature. 2012 Jun;486(7402):207–14.

21. Methé BA, Nelson KE, Pop M, Creasy HH, Giglio MG, Huttenhower C, et al. A framework for human microbiome research. Nature. 2012 Jun;486(7402):215–21.

22. He X, Parenti M, Grip T, Lönnerdal B, Timby N, Domellöf M, et al. Fecal microbiome and metabolome of infants fed bovine MFGM supplemented formula or standard formula with breast-fed infants as reference: a randomized controlled trial. Sci Rep. 2019 Aug;9(1):11589.

23. Lloyd-Price J, Arze C, Ananthakrishnan AN, Schirmer M, Avila-Pacheco J, Poon TW, et al. Multi-omics of the gut microbial ecosystem in inflammatory bowel diseases. Nature. 2019 May;569(7758):655–62.

24. Yurgel SN, Douglas GM, Dusault A, Percival D, Langille MGI. Dissecting community structure in wild blueberry root and soil microbiome. Front Microbiol. 2018 Jun 6;9(JUN):1187.

25. Nearing JT, Douglas GM, Hayes MG, MacDonald J, Desai DK, Allward N, et al. Microbiome differential abundance methods produce different results across 38 datasets. Nat Commun. 2022 Dec 1;13(1).

26. Hawinkel S, Mattiello F, Bijnens L, Thas O. A broken promise: microbiome differential abundance methods do not control the false discovery rate. Brief Bioinform. 2019 Jan;20(1):210–21.

27. Calgaro M, Romualdi C, Waldron L, Risso D, Vitulo N. Assessment of statistical methods from single cell, bulk RNA-seq, and metagenomics applied to microbiome data. Genome Biol. 2020 Aug;21(1):191.

28. Lin H, Peddada S Das. Analysis of microbial compositions: a review of normalization and differential abundance analysis. NPJ Biofilms Microbiomes. 2020 Dec;6(1):60.
