# Supplementary material for: Investigating differential abundance methods in microbiome data: A benchmark study
Source: PLoS Comput Biol. 2022 Sep 8;18(9):e1010467. doi: 10.1371/journal.pcbi.1010467 (PMC9488820; doi:10.1371/journal.pcbi.1010467)
Supplement: Attachment — Submitted filename: Review of Cappellato et al.pdf [file pcbi.1010467.s002.pdf]

Review of Cappellato et al.,

In this manuscript Cappellato et al. report various performance metrics for eleven different methods that aim to identify differentially abundant taxa within microbiome datasets. Within the manuscript they report the false positive rate, false discovery rate, recall, and AUPR of each tool tested at four different sample sizes and various percentages of differentially abundant features. All these reported values are based on synthetic datasets created using their recently developed tool metaSPARSim, which makes use of both a gamma distribution to model microbial counts and a multivariate hypergeometric distribution to model technical variability. The parameters used to generate each dataset tested within this manuscript have been pulled from three different studies examining the microbial communities present on teeth, or those present in the gut of 6 month of old breast-fed infants or healthy individuals in the IBDMD/HMP2 study. Overall, the manuscript is well written and shows a strong analysis of the various tools, however, there are several concerns that should be addressed.

Major concerns

While the authors do a good job within the introduction of introducing the issues of compositionality their classification of each tools ability to model these types of abundances seem too not be as straight forward. For example, while it is true that tools such as corncob, and metagenomeSeq model the counts of each feature directly or after normalisation it is not clearly pointed out that the feature counts these tools are modeling are relative to one another due to the compositional effects of sequencing depth. Therefore, while it is true these tools are modeling counts directly it does not mean they are better equipped for identifying changes in the actual cellular counts present within the observed communities which is often associated with the term absolute abundances. Another example of this occurs when, they indicate Aldex2 as examining relative abundances. This tool, however, uses a center-log-ratio transformation and is modeling the fold change of taxa between samples in reference to each samples geometric mean. It is unclear how it was decided that this examination it more akin to relative abundances than absolute abundances. Especially considering previous work that has argued that center-log-transformations may allow users to make better estimates at the true cellular count changes within a community (https://doi.org/10.1038/s41467-019-10656-5). As such I think better wording and careful considerations need to be taken around the construction of the last column in table 1. Furthermore, the authors need to consider how they use the term “absolute abundance” throughout the manuscript as in many cases its unclear whether they are referring to the cellular counts with the observed communities or simplify the raw or normalised count data that came off the sequencer.

While the authors do a great job at discussing the performance of tools when looking at differentially abundant features that are in the medium or high abundance range it is unclear how often this represents true scenarios in microbiome datasets. As such I think the authors might want to consider including some of the supplemental figures of their later examination of low abundance DA features in the main text.

The simulated datasets that the authors use to benchmark the tools in their report derive from human associated environments. It is unclear whether tool performance would differ if the simulation parameters were derived from environmental samples such as sediment or ocean water. Including some data on whether they would expect this to impact their simulations, or the benchmarks found in this paper would help readers who are interested in characterizing environments that are not human associated.

Other comments:

In the supplemental figures indicating computational time it is unclear whether each tool was run using a single core or multiple cores as many tools allow for the use of both. If possible, it would be best if the authors reported true cpu time (the time the cpu spent working on this task) rather than the change in system time between the start and end of their computation. This could be important if other software or OS updates were running during there testing time.

The supplemental information for this manuscript contains 73 figures and while they do a great job at presenting all their data, they might want to consider reorganizing the contents to highlight the more impactful figures such as those directly mentioned within the text.

Please indicate in the main text the reasoning behind why the mean values in condition B for a taxon were limited to the range of mean values in condition A. This is surely not something seen in all microbiome datasets and could impact the performance of various DA tools.

It was interesting to see the high FPR of metagenomeSeq and the low FPR of edgeR despite a recent publication finding the opposite (https://doi.org/10.1038/s41467-022-28034-z). It would be great if the authors discussed the opposite results as many of the other conclusions from these papers agree with one another.

The authors examine the performance of tools due to varying sequencing depths, however, it would be interesting to see tool performance when mean sequencing depths between conditions A and B differ from one another as this issue can often occur during microbiome analysis.

While it is not need it would strengthen the manuscript to see other popular DA microbiome tools included in their analysis such as MaAsLin2 or LEfSe.

Like many benchmarking studies the authors conclude that DA tools perform similarly to one another with no clear choice in which tool is best to use. Considering this information, it would be nice if the authors gave some recommendations on how to properly conduct DA analysis in their own datasets.

I was unable to locate a legend for figure 1.
