# Supplementary material for: Investigating differential abundance methods in microbiome data: A benchmark study
Source: PLoS Comput Biol. 2022 Sep 8;18(9):e1010467. doi: 10.1371/journal.pcbi.1010467 (PMC9488820; doi:10.1371/journal.pcbi.1010467)
Supplement: Attachment — Submitted filename: Cappellato_Baruzzo_DiCamillo_ReviewAnswer.pdf [file pcbi.1010467.s003.pdf]

Dear Editor,

We addressed all the points raised by all the three reviewers and reported all the changes in the main paper and supplementary material highlighting the text in red.

In details we:

- Performed a more in-deepth investigation of metagenomeSeq. In brief, we compare the two underlyng model, namely the zero-inflated Log-Gaussian (ZILG) mixture model and the zero-inflated Gaussian (ZIG) model. We show that ZILG perform better than ZIG. Then, we run ZILG model from metagenomeSeq package in all simulations. All figures in manuscript and supplementary material are updates with new metagenomeSeq results.
- Improved the description of DA tools, providing further details about methods' assumptions and adopted models. We also clarify the wording regarding the terms "absolute abundance" and "relative abundance", as well as provide a more complete and detailed description of which type of differential abundance testing is handled by each method.
- Clarified some aspects of the simulation process (i.e. Equation 4, and the simulation of differentially absolute/relative abundant features).
- Tested the effect of normalising data using GMPR normalisation (in addition to the tool suggested/built-in normalisation).
- Tested the effect of an unbalanced sequencing depth between the two experimental conditions.
- Added a new differentially abundance tool, i.e. MaAsLin2. We run this method in all the scenario tested; we updated all figures in the manuscript and supplementary file and finally we included comment on its performance.
- Added two not human associated datasets to study the effect of the ecological niche on tools performances.
- Included a supplementary figure about low abundance DA features in the main text (Fig 9).
- Checked the execution time of all methods.
- Further summarised the results to provide more clear suggestions and recommendations. For example, we commented what methods can control FDR and achieve an high recall at the same time.
- Further improved the discussion of the results adding a new section that focus on overall performance of the methods.
- Improved the readability of the manuscript, adding sub-headers and redesign some figures to be more readable and informative (e.g. new figure 5, 8, 10-12).

We think the paper is significantly improved thanks to the suggestions of the reviewers and hope it is now publishable on Plos Computational Biology.
I will refer you to the response to the reviewer for the details.

Best regards,
Barbara Di Camillo

Reviewer #1:

The authors compared different methods for testing differential microbial abundance using simulation studies. There are usually two types of tests, one for testing relative abundance difference and one for testing absolute abundance difference.

(1) The authors compared the methods in terms of testing absolute abundance difference between two groups. In addition to compare the methods using simulation studies, it will be great to discuss the statistical models used in different methods and whether they are appropriate for testing the changes in "absolute abundance"

Thank you for this suggestion that help clarifying the content of our ms.

You are right observing that statistical models used in the literature for DA analysis are designed for testing either the changes in “absolute abundance” [1–6] or the changes in “relative abundance” [7–9].

We added a more explicit definition of true/observed absolute and relative abundance (new Fig 1 and a paragraph in the introduction) and discussed more in details the focus of each method (paragraph in material and methods and S1 File, Section 2) already summarised in Table 1 of the main ms. The new figures and paragraphs are also reported here for reviewer convenience.

<u>Paragraph added in the Introduction</u>

As previously noted by Lin et al. [13], many authors use the terms relative abundance and absolute abundance differently, causing difficulties in understanding not only the method, but also in interpreting the results of other benchmarking studies. Therefore, an explicit notation would be desirable. We summarise all the terms used in the example above in Fig 1. In particular, "true absolute abundance" means the number of bacteria in the analysed specimen, whereas "true relative abundance" refers to proportions, i.e. the percentage of bacteria compared to the total number. If these quantities refer to the output of the sequencing process, we use the term "observed", i.e. “observed absolute abundance” is the number of counts measured by the sequencing process and “observed relative abundances” are the corresponding proportions relative to the total counts.

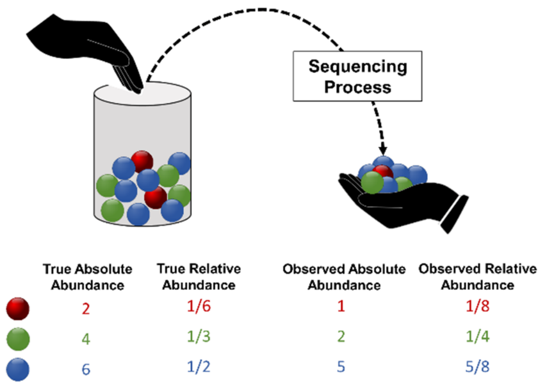

Fig 1. Summary of notations. The urn represents the real microbial population where different bacteria are represented by spheres of different colours. The sequencing process can be described by sampling without replacement with a limited number of extractions (i.e. the sequencing depth).

<u>Paragraph added in Material and Methods</u>

Table 1 summarises the methods considered in this benchmark study, providing information about software availability, underlying approach, preprocessing performed by the tool and adopted definition of DA taxa, i.e. if the methods aim at investigating significant differences in mean true absolute or relative abundance of each taxon. DESeq2, edgeR, MaAsLin2 and metagenomeSeq model the observed absolute abundance of each taxon using different statistical models with the goal of estimating the FC, that, if data are opportunely normalised, should reflect the FC between the true absolute abundances. ANCOM and ANCOM-BC are explicitly designed to target the true absolute abundances. All the other methods presented in Table 1 are classified as tools targeting the differentially relative abundance. A more detailed description of the methods is given in S1 File, Section 2.

<u>Paragraph added in S1 File, Section 2</u>

In Table 1 in the main text, we classify each method as addressing relative or absolute abundances based on the underlying definition of DA taxa adopted and the preprocessing adopted. DESeq2, edgeR and metagenomeSeq apply normalisation in order to overcome bias introduced by different microbial loads and sequencing depths across samples. Although these methods use different statistical models, in principle normalisation should correct the above biased in order to be able to estimate Fold Changes between conditions

without compositional bias. Therefore, we indicated them as addressing absolute differential abundance. Similarly, since we set TSS as a normalisation approach in MaAsLin2, we characterise this method as addressing absolute differential abundance. However, this tool can also run on observed relative abundances and exploiting different data transformation, thus, in principle, it can potentially be classified as addressing relative abundance.

The authors of ANCOM and ANCOM-BC demonstrate that their models allow to explicitly test the differences in terms of true absolute abundance [9,11].

On the other hand, although corncob models directly observed absolute abundance, its approach aim at modelling the expected true relative abundance of each taxon and how it is linked to covariates in the data. For this reason, we classified this method as addressing relative differential abundance.

Similarly to ALDEx2, eBay models observed absolute abundances using a Bayesian approach. The authors formulate a Posterior Bayesian estimation for the true relative abundances. In both methods the estimated values are transformed with clr transformation in order to project relative data from Simplex to Euclidean space. The transformation is necessary to then carry out the statistical test correctly. According to the definition of clr, the differences between two or more groups are evaluated in relation to the geometric mean abundance. However, the transformation is used to project the data into Euclidean space and make the proportions comparable, but it does not change the nature of the data. Consequently, these methods compare proportions and were classified as addressing relative differential abundance.

Moreover, to account for both absolute and relative abundance scenarios, we made explicit how our simulation framework accounts for both scenarios (new paragraph in the Introduction and a new sub-section, also reported here for reviewer convenience).

Paragraph added in the Introduction

The statistical models used in the literature for DA analysis are designed for testing either the changes in "absolute abundance" or the changes in "relative abundance" [13]. To account for both scenarios, we developed our simulation framework accounting for sequencing data compositionality, i.e. the fact that changes in abundance of one taxon affects the observed abundances of other taxa [7–11]. In particular, we balanced over and under abundant taxa so to create scenarios in which the simulated DA taxa are differentially abundant both in terms of true absolute abundance and true relative abundance. Consequently, our simulation can be used to assess both the methods that test differentially absolute abundant taxa and those that test differentially relative abundant taxa.

New sub-section added in Material and Methods (section: "Simulating 16S count data with differentially abundant taxa – DA taxa: differences between absolute and relative abundances")

It should be noted that potentially, as first pointed out in Weiss et al. [11], introducing a DA feature as a change in absolute abundance could affect all the other features by a variation in terms of their relative abundances. Therefore, different methods targeting relative abundance, might identify correctly as relevant many features not intentionally simulated as DA.

In previous studies [11,17,19], the balance of non-DA features after the application of FC has been investigated in different ways. Weiss et al. [11] have proposed an approach based on the duplication of all the simulated DA features, in which the FC alternately multiplies the two experimental groups. In this way, the authors create a balanced simulation, where the features are DA in terms of both absolute and relative abundance. In Hawinkel et al. [17], on the other hand, an approach called "compensation" is introduced. In short, the mean true relative abundance of a fraction $\frac{1}{FC+1}$ *of the chosen DA features (whose true relative abundances sum to a) is multiplied by FC, while the remaining* $\frac{FC}{FC+1}$ *features (whose true relative abundances sum to b) are multiplied by* $\frac{a}{b}(1-FC)+1$*. In this way, the introduction of the DA features does not change the mean relative abundances of taxa equally abundant. Unfortunately, this approach is only possible if the FC value is constant, a scenario that hardly comes close to reality.*

In our benchmark study, we balanced over and under abundant taxa so to create scenarios in which the simulated DA taxa are differentially abundant both in terms of true absolute abundance and true relative abundance (Fig 1). This property was verified a-posteriori as shown in Figures 1-3 in S1 File and explained in the following.
According to the use of a Fold Change approach to simulate differentially absolute abundant features (Equation 3), we use the same definition to define taxa differentially relative abundance (or Proportionally Differentially Abundant, PDA). A taxon i is PDA if:

$$P_i^A = FC_i \cdot P_i^B \quad (5)$$

Where P_i^A *is the mean true relative abundance in the experimental group A:* $P_i^A = \frac{\mu_i^A}{\sum_{j=1}^{N} \mu_i^A}$.
In Figures 1-3 in S1 File, the MVA plots, i.e the FC versus mean average in log scale (base 2) of the relative abundances in the two groups (i.e P_i^A *and* P_i^A*) in all the simulated scenarios are shown for a single run. The DA features (in red) are those defined as absolute DA based on Equation (3). As evident from Figures 1-3 in S1 File, absolute DA features are also PDA with fold change greater that 2 in the majority of cases, and always well distinguishable from not DA features (black dots).*

(2) Usually normalization has effects on the results of tests. Have the authors checked the influence of different normalization methods?

We agree that it's interesting to check the influence of normalisation on methods' performance. Following your suggestion, we have added this investigation as a further scenario and reported some general considerations in discussion. Results are shown in 2 new figures in the main manuscript (Figures 10-11) and in several new figures in the Supplementary Materials (Figures 86-91 in S1 File).
The new paragraphs and the two figures are also reported here for reviewer convenience.

Paragraph added in Material and Methods – Assessment scenarios

To investigate whether by changing the default normalisation the performance of the methods changes, we choose to test the methods with a normalization specifically developed for microbiome data that is not used by any DA method considered in this study: the geometric mean of pairwise ratios (GMPR [39]). It is fair to highlight that some methods, namely ALDEx2, ANCOM, ANCOM-BC, corncob, eBay, would require as input raw data, since normalisation or data transformation is an integral part of the model used (see S1 File, Section 2). However, in this simulation scenario, for sake of comparison, we gave normalised data as input to all methods.

New sub-section added in Results – Effect of normalization

Figures 86-91 in S1 File show results obtained using GMPR normalisation in the scenario with DA taxa in the mid-high range (i.e. by setting the θ threshold). Fig. 10-11 show an example of FDR and Recall for one of the 3 datasets under consideration where statistically significant differences between normalised and unnormalized version of each method (Wilcoxon test, nominal p-value not corrected for multiple testing lower than 0.05) are indicated by a star symbol on the left (see Figures 92-93 in S1 File for the other datasets). As can be seen, GMPR normalisation mainly affect method performance at low sample size and low P. The differences in FDR and Recall vary across methods and datasets, both in terms of sign and magnitude. However, most of the differences are not statistically significant, and no tool clearly benefits from GMPR normalisation compared to the base scenario. Overall, GMPR normalisation does not have a clear impact in the ranking of the methods, both in terms of FDR and Recall, compared to the base scenario (i.e. methods run with default normalisation).

Paragraph added in Discussion

In general, there is a disagreement in the literature regarding the effect of normalisation on Differential Abundance testing. Hawinkel et al. [17] have reported that there is no normalisation that greatly improves the performance of the methods; thus the default normalisation suggested by the authors of the tools or the simple

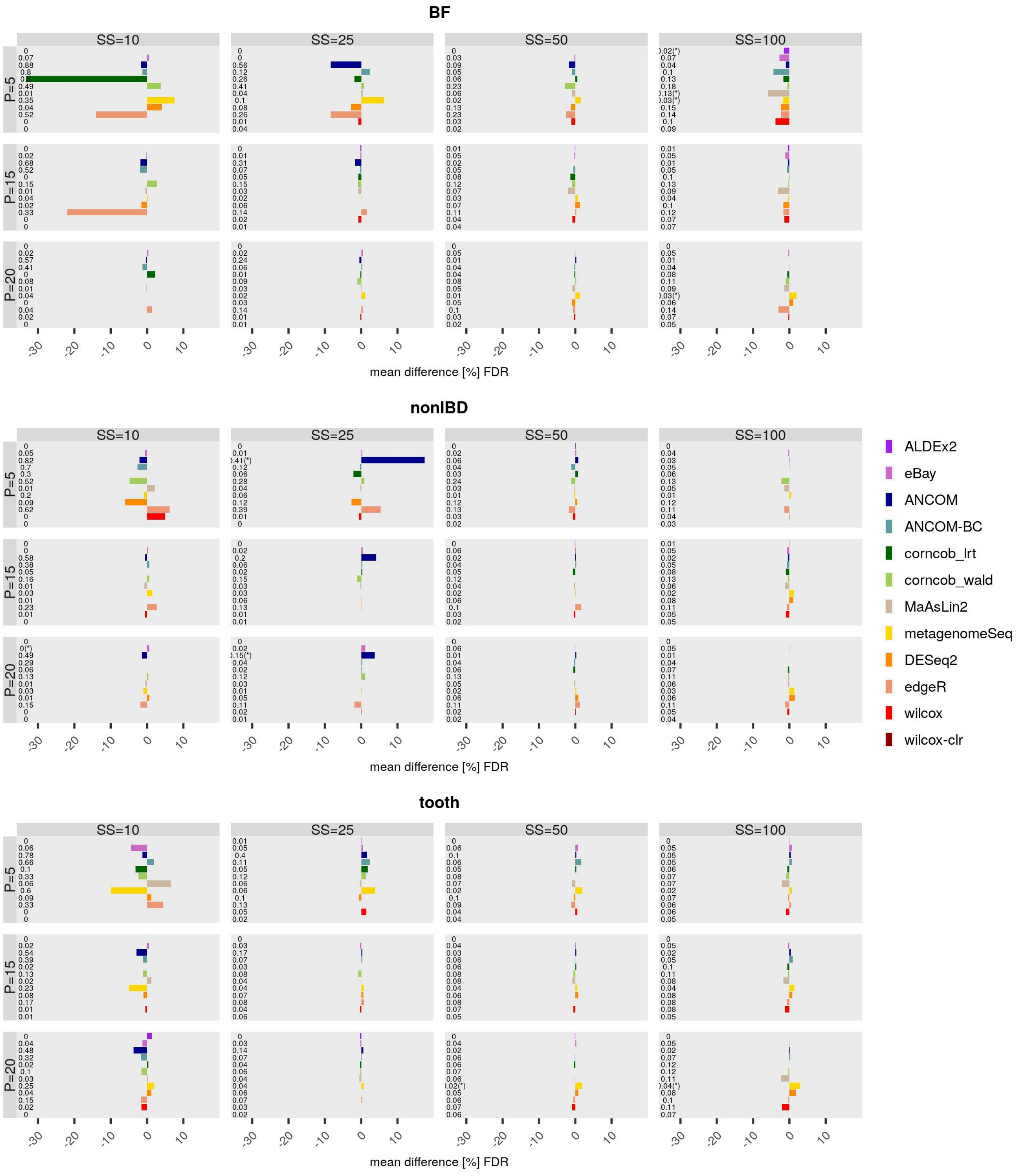

Fig 10. Mean FDR difference [%] between each differential abundance method and its GMPR normalised version for BF, nonIBD and tooth dataset in the scenario with simulated DA features. In each set of boxes corresponding to the dataset, different percentages (P) of simulated DA features are on rows, while different sample size (SS) values are on columns. Numbers at the beginning of each row correspond to the FDR values obtained with default normalization, while the symbol (*) identifies that the Wilcoxon unpaired statistical test is significant.

use of the sequencing depth correction are both reasonable choices. Recently, Baruzzo et al. [51] have observed that normalisation has a good impact on the differential testing, although with limited effect size. The performances shown in Lin et al. [13] also demonstrate that different normalisation methods have a limited impact on recall, but more evident on the precision. Indeed, in Lin et al. [13] the only method among the tested ones that seems to change the outcome of the FDR control in one of the three simulated scenarios is the Wilcoxon test, where the variant without normalisation has higher recall and lower FDR values compare to the ones obtained with TSS normalisation. In Mallick et al. [29] TSS is identified as the best normalisation

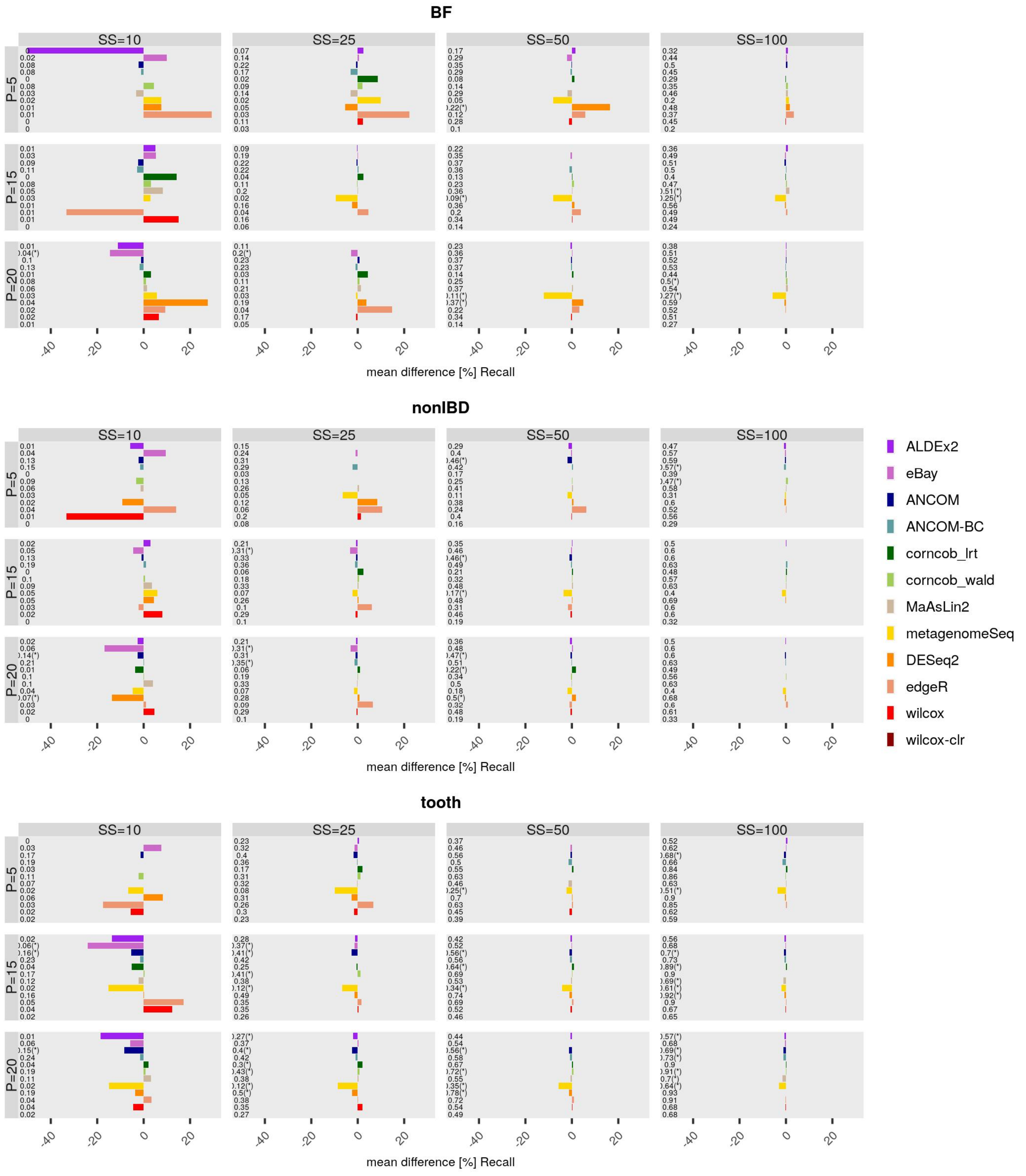

Fig 11. Mean Recall difference [%] between each differential abundance method and its GMPR normalised version for BF, nonIBD and tooth dataset in the scenario with simulated DA features. In each set of boxes corresponding to the dataset, different percentages (P) of simulated DA features are on rows, while different sample size (SS) values are on columns. Numbers at the beginning of each row correspond to the Recall values obtained with default normalization, while the symbol (*) identifies that the Wilcoxon paired statistical test is significant.

choice, in terms of effect on FDR control and good statistical power, compared to model-based normalisation schemes (such as RLE) or data-driven normalisation methods (e.g. TMM, CSS, or the log-ratio transformation in ANCOM, ALDEx2 and eBay). Finally, in Calgaro et al. [19] normalisation seems to have an impact on inferred results especially in term of consistency (i.e. the ability of each method to produce replicable results in independent data) and on differential abundance analysis framework. Results obtained in our benchmarking show that GMPR does not affect the overall ranking, in accordance with the results shown in Hawinkel et al. [17].

(3) For comparison of methods on testing differential abundance, in addition to controlling the FDR at a desired significance level such as 0.05, we would like to compare the power of tests. What methods would you recommend using the criterion of maximizing the power of the test while controlling the FDR?

As the reviewer rightly pointed out, analysts usually set the desired significance threshold for the FDR at 0.05 and are then interested in knowing which method reach higher Recall while controlling the FDR expected value. As suggested by the reviewer, to facilitate the reader to analyse the results, we generated the average FDR vs Recall plots for each simulated scenario and comment it in the main manuscript (new Figure 5 and

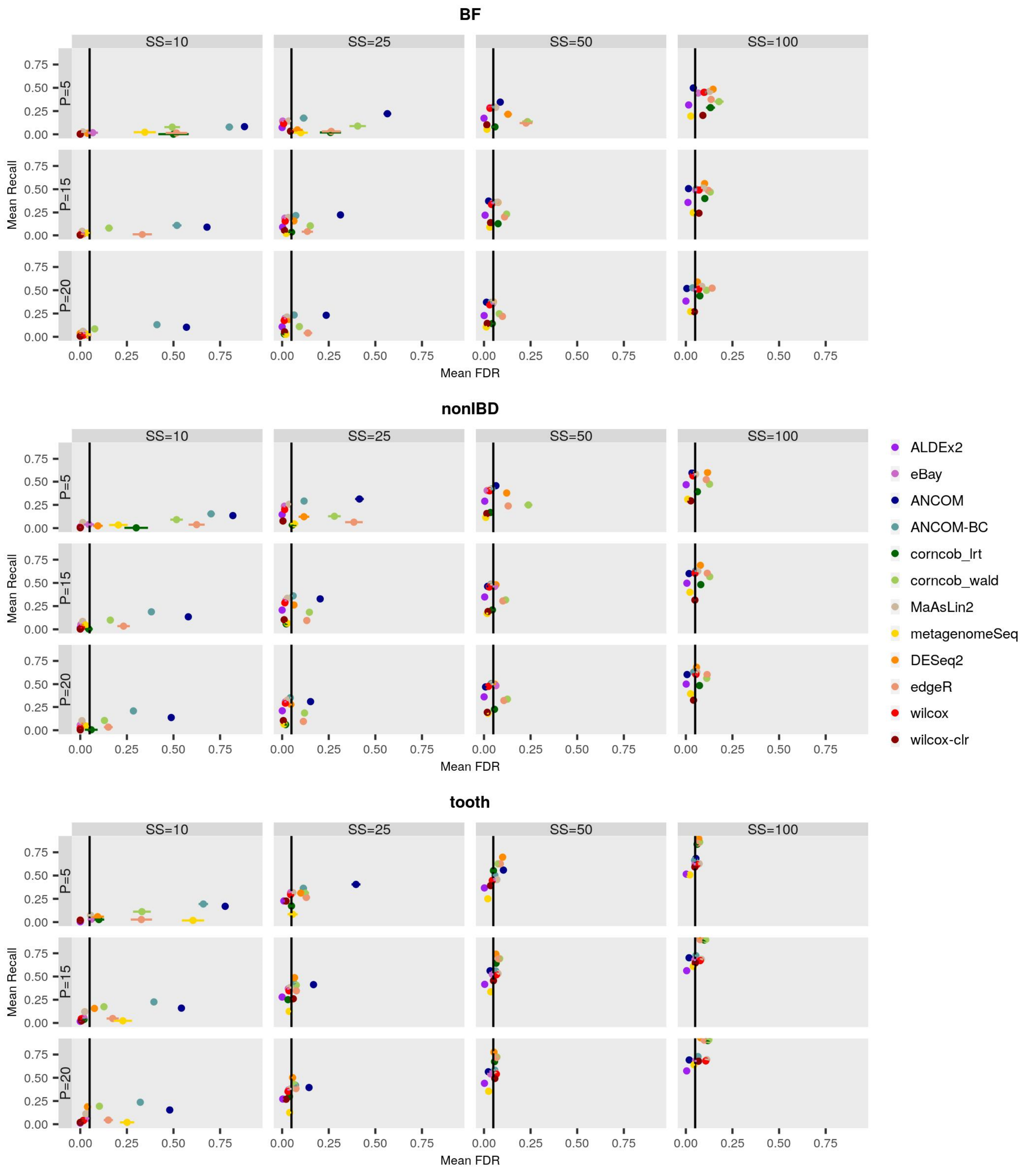

Fig 5. Mean Recall (on y axis) and FDR (on x axis) of each differential abundance method for each dataset considered in the comparison in the main scenario with simulated DA features. In each set of boxes corresponding to the dataset, different percentages (P) of simulated DA features are on rows, while different sample size (SS) values are on columns. The recall values are averaged over the 50 simulations and the bars show the standard error.

new paragraph "Results – Performance evaluation on simulation with DA taxa – Recall", both reported here for reviewer convenience).

Paragraph added in Results – Performance evaluation on simulation with DA taxa – Recall

Analysts usually set the desired significance threshold for the FDR at 0.05 and are interested in knowing which method reach higher Recall while controlling the FDR expected value. As shown in Fig 5, in tooth dataset at SS=100, DESeq2, corncob and edgeR are the best methods since it reaches the highest Recall values while controlling the FDR at the desired significance level (i.e. the black line) for most of the scenarios shown. However, looking at the other datasets there is no well-defined winner. In general, although at low sample size some methods still control the FDR, the performance in terms of recall is extremely low.

To further summarise the results and provide some guidance about what methods can control FDR and achieve high recall in the majority of the dataset tested, we add Fig 12 and a dedicated paragraph in the new sub-section Results – *Overall performance*. The figure shows the methods sorted first by highest precision (highest class contains the methods that control the FDR), and then by the highest recall. Figure 12 and the new sub-section are also reported here for reviewer convenience.

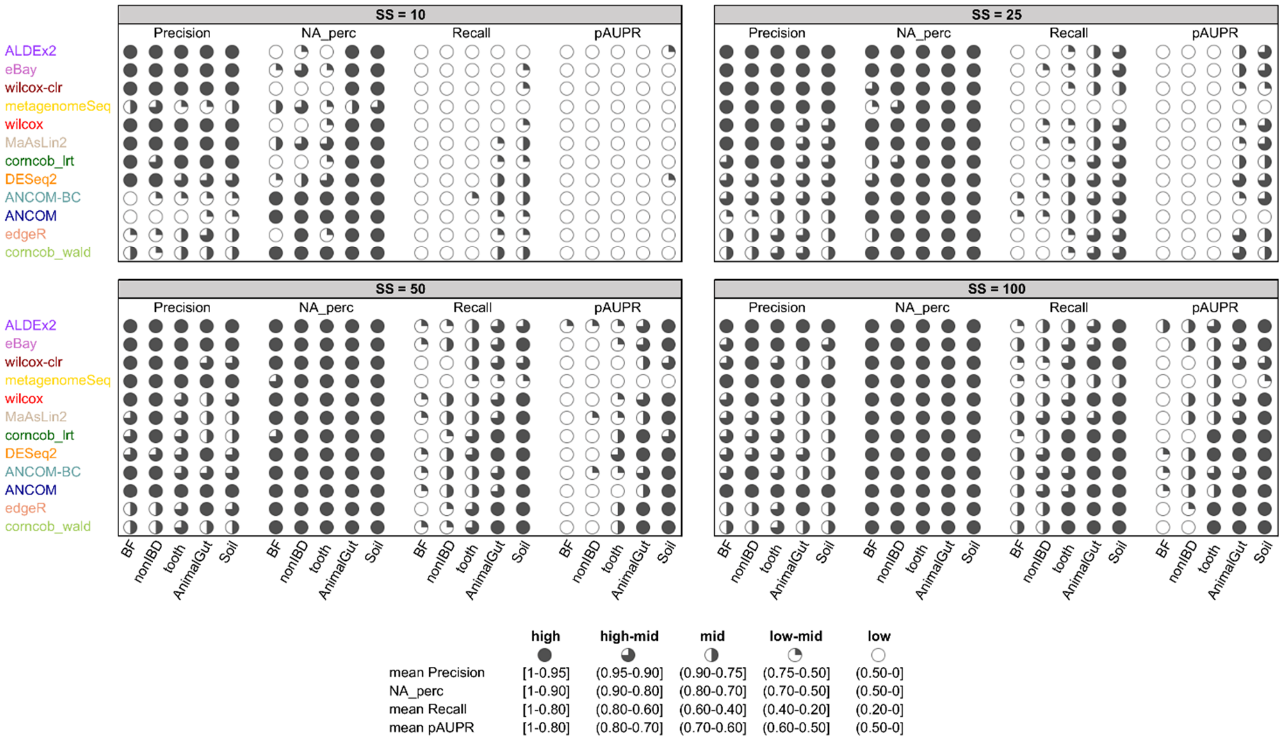

Fig 12. Overall performance of each DA method. In each set of boxes corresponding to different sample size (SS) values, Precision, NA_perc (percentage of available precision), Recall and pAUPR scores are shown for each dataset in columns. Methods (on rows) are ranked based on Precision values across all the SS scenarios and then based on Recall in case of ties. The legend below the boxes explains the threshold used to assign the overall score for each metric.

Paragraph added in Results – Summary of overall performance

However, the ranking shown in Figure 106-107 in S1 File does not take into account the average value of the metric. For example if all methods fail to control the FDR, the ranking still finds a winner among the methods. Consequently, we assign a score between 1 and 5 (1 = "low", 2 = "low-mid", 3 = "mid", 4 = "high-mid" and 5 = "high") to each mean value of the metrics by defining some thresholds on metrics values. In brief, methods having a high precision and controlling the nominal FDR of 0.05 (i.e. precision >= 0.95) were assigned to class "high". Method achieving a precision >= 0.90 were assigned to class "high-mid", representing tools that still provide a very high precision but slightly exceeding the chosen FDR threshold. Overall, methods in the above two classes represent robust alternatives when controlling type I error is a relevant constraint for the study. The remaining classes describes methods having a precision < 0.9, with the

class "low" containing the methods achieving a maximum precision of 0.5. Closely related to precision, we also classified the methods in terms of "NA_perc", i.e. percentage of datasets for which at least one taxon has been identified as DA. Methods able to identify at least one DA taxon in the great majority of datasets (i.e. > 75%) were assigned to the highest classes. In particular, methods able to identify at least one DA taxon in 100% or more than 90%, 75% of datasets were assigned to classes "high", "high-mid" and "mid", respectively. Performance in terms of recall were ranked in 5 classes representing the 5 quantiles in the recall range [0-1]. Since the recall is the metrics showing the largest difference across different number of samples SS, covering almost the entire range [0-1], a stratification in 5 quantiles provide a fair and straightforward way to summarise method performance. In terms of pAURP, the lowest class represents methods having an pAURP < 0.5, while a pAUPR > 0.7 could be a reasonable labeled as "high", "high-mid" and "mid" with 0.1 step.

Fig. 12 shows the methods ranked as described above, sorting them first by precision and then by recall, across all SS scenarios in each dataset. In SS = 10, MaAsLin2 always control the precision and reaches higher recall values in 2 out of 5 datasets. As the sample size increases, a pattern seems clearer. ALDEx2 is among the best in terms of FDR control on all datasets for SS = 25, 50, 100; similarly eBay shows good results for SS = 25-50; while ANCOM reaches top performance for SS = 50-100. The recall values of these methods are in the top ranking, although there are methods that achieve higher recall at the expense of not controlling the FDR in the majority of the datasets.

(4) In the simulation study, what is the motivation to require equation (4)?

$$m=min(\mu^A)<\mu_i^B<M=max(\mu^A)$$

As now reported in the main ms (section Simulating 16S count data with differentially abundant taxa – Simulation of DA taxa) *"It is worth noting that setting a range on the mean absolute abundances for condition B has the purpose of generating data in a plausible range. However, this does not imply that the two conditions have exactly the same maximum and minimum intensity in the resulting count table. In fact, μ is provided as input to the gamma distribution, and, after the application of the biological (Equation 1) and technical (Equation 2) noise, it is reasonable to expect that the two conditions have different average maximum observed abundances, although roughly in the range of values observed in the real experimental condition."*

Reviewer #2:

In this manuscript Cappellato et al. report various performance metrics for eleven different methods that aim to identify differentially abundant taxa within microbiome datasets. Within the manuscript they report the false positive rate, false discovery rate, recall, and AUPR of each tool tested at four different sample sizes and various percentages of differentially abundant features. All these reported values are based on synthetic datasets created using their recently developed tool metaSPARSim, which makes use of both a gamma distribution to model microbial counts and a multivariate hypergeometric distribution to model technical variability. The parameters used to generate each dataset tested within this manuscript have been pulled from three different studies examining the microbial communities present on teeth, or those present in the gut of 6 month of old breast-fed infants or healthy individuals in the IBDMD/HMP2 study. Overall, the manuscript is well written and shows a strong analysis of the various tools, however, there are several concerns that should be addressed.

Major concerns

While the authors do a good job within the introduction of introducing the issues of compositionality their classification of each tools ability to model these types of abundances seem too not be as straight forward. For example, while it is true that tools such as corncob, and metagenomeSeq model the counts of each feature directly or after normalisation it is not clearly pointed out that the feature counts these tools are modeling are

relative to one another due to the compositional effects of sequencing depth. Therefore, while it is true these tools are modeling counts directly it does not mean they are better equipped for identifying changes in the actual cellular counts present within the observed communities which is often associated with the term absolute abundances. Another example of this occurs when, they indicate Aldex2 as examining relative abundances. This tool, however, uses a center-log-ratio transformation and is modeling the fold change of taxa between samples in reference to each samples geometric mean. It is unclear how it was decided that this examination it more akin to relative abundances than absolute abundances. Especially considering previous work that has argued that center-log-transformations may allow users to make better estimates at the true cellular count changes within a community (https://doi.org/10.1038/s41467-019-10656-5). As such I think better wording and careful considerations need to be taken around the construction of the last column in table

As pointed out by the reviewer and reported by Lin et al. [10], "Some methods are designed for testing hypotheses regarding the relative abundance, while others are designed for testing hypothesis regarding absolute abundance". We also agree that, depending on data transformation and normalisation used, the focus of the methods can change from relative to absolute differential abundance.

To try disambiguating we:

- Added a more explicit definition of true/observed absolute and relative abundance (see answer to your question 1 in the next paragraph).
- Discussed more in details the focus of each method (S1 File, Section 2) as also requested by reviewer 1. Note that, although we agree that corncob models relative differential abundance (and not absolute as originally written), we kept the indication that Aldex2 examines relative abundances because , as also reported by the authors, they "*refer to clr-transformed values as relative abundance values…*" and apply statistical tests directly to clr transformed data (not to fold changes between samples) "*Each realization of the ci,j value between conditions is subjected to both a Welch's t-test and a Wilcoxon rank test giving two vectors of P values, and each P value instance is then corrected for multiple hypothesis testing using the false discovery rate (fdr) approach of Benjamini and Hochberg.*".
- We made explicit how our simulation framework accounts for both scenarios. We report in the following the added paragraph for the reviewer convenience:

Paragraph added in S1 File, Section 2

In Table 1 in the main text, we classify each method as addressing relative or absolute abundances based on the underlying definition of DA taxa adopted and the preprocessing adopted. DESeq2, edgeR and metagenomeSeq apply normalisation in order to overcome bias introduced by different microbial loads and sequencing depths across samples. Although these methods use different statistical models, in principle normalisation should correct the above biased in order to be able to estimate Fold Changes between conditions without compositional bias. Therefore, we indicated them as addressing absolute differential abundance. Similarly, since we set TSS as a normalisation approach in MaAsLin2, we characterise this method as addressing absolute differential abundance. However, this tool can also run on observed relative abundances and exploiting different data transformation, thus, in principle, it can potentially be classified as addressing relative abundance.
The authors of ANCOM and ANCOM-BC demonstrate that their models allow to explicitly test the differences in terms of true absolute abundance [9,11].
On the other hand, although corncob models directly observed absolute abundance, its approach aim at modelling the expected true relative abundance of each taxon and how it is linked to covariates in the data. For this reason, we classified this method as addressing relative differential abundance.
Similarly to ALDEx2, eBay models observed absolute abundances using a Bayesian approach. The authors formulate a Posterior Bayesian estimation for the true relative abundances. In both methods the estimated values are transformed with clr transformation in order to project relative data from Simplex to Euclidean space. The transformation is necessary to then carry out the statistical test correctly. According to the definition of clr, the differences between two or more groups are evaluated in relation to the geometric mean abundance. However, the transformation is used to project the data into Euclidean space and make the

proportions comparable, but it does not change the nature of the data. Consequently, these methods compare proportions and were classified as addressing relative differential abundance.

New sub-section added in Simulating 16S count data with differentially abundant taxa – DA taxa: differences between absolute and relative abundances"

It should be noted that potentially, as first pointed out in Weiss et al. [11], introducing a DA feature as a change in absolute abundance could affect all the other features by a variation in terms of their relative abundances. Therefore, different methods targeting relative abundance, might identify correctly as relevant many features not intentionally simulated as DA.
In previous studies [11,17,19], the balance of non-DA features after the application of FC has been investigated in different ways. Weiss et al. [11] have proposed an approach based on the duplication of all the simulated DA features, in which the FC alternately multiplies the two experimental groups. In this way, the authors create a balanced simulation, where the features are DA in terms of both absolute and relative abundance. In Hawinkel et al. [17], on the other hand, an approach called "compensation" is introduced. In short, the mean true relative abundance of a fraction $\frac{1}{FC+1}$ *of the chosen DA features (whose true relative abundances sum to a) is multiplied by FC, while the remaining* $\frac{FC}{FC+1}$ *features (whose true relative abundances sum to b) are multiplied by* $\frac{a}{b}(1\text{-}FC)+1$*. In this way, the introduction of the DA features does not change the mean relative abundances of taxa equally abundant. Unfortunately, this approach is only possible if the FC value is constant, a scenario that hardly comes close to reality.*
In our benchmark study, we balanced over and under abundant taxa so to create scenarios in which the simulated DA taxa are differentially abundant both in terms of true absolute abundance and true relative abundance (Fig 1). This property was verified a-posteriori as shown in Figures 1-3 in S1 File and explained in the following.
According to the use of a Fold Change approach to simulate differentially absolute abundant features (Equation 3), we use the same definition to define taxa differentially relative abundance (or Proportionally Differentially Abundant, PDA). A taxon i is PDA if:

$$P_i^A = FC_i \cdot P_i^B \qquad (5)$$

Where P_i^A *is the mean true relative abundance in the experimental group A:* $P_i^A = \frac{\mu_i^A}{\sum_{j=1}^{N} \mu_i^A}$.
In Figures 1-3 in S1 File, the MVA plots, i.e the FC versus mean average in log scale (base 2) of the relative abundances in the two groups (i.e P_i^A *and* P_i^A*) in all the simulated scenarios are shown for a single run. The DA features (in red) are those defined as absolute DA based on Equation (3). As evident from Figures 1-3 in S1 File, absolute DA features are also PDA with fold change greater that 2 in the majority of cases, and always well distinguishable from not DA features (black dots).*

1. Furthermore, the authors need to consider how they use the term "absolute abundance" throughout the manuscript as in many cases its unclear whether they are referring to the cellular counts with the observed communities or simplify the raw or normalised count data that came off the sequencer.

From your comment we have realised that the main manuscript lacks clarity regarding the terms used to indicate the concept of absolute and relative abundance. We have revised the manuscript to make the use of these terms clearer.

Paragraph added in the Introduction

As previously noted by Lin et al. [13], many authors use the terms relative abundance and absolute abundance differently, causing difficulties in understanding not only the method, but also in interpreting the results of other benchmarking studies. Therefore, an explicit notation would be desirable. We summarise all the terms used in the example above in Fig 1. In particular, "true absolute abundance" means the number of bacteria in the analysed specimen, whereas "true relative abundance" refers to proportions, i.e. the percentage of bacteria compared to the total number. If these quantities refer to the output of the sequencing process, we use the term

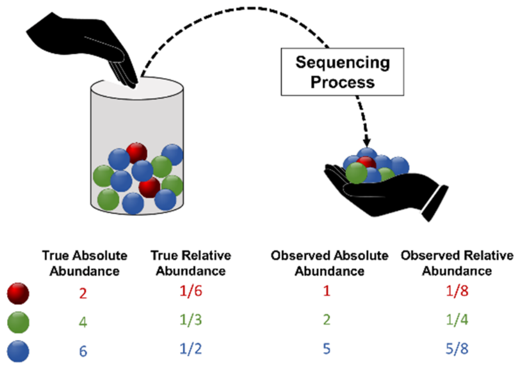

Fig 1. Summary of notations. The urn represents the real microbial population where different bacteria are represented by spheres of different colours. The sequencing process can be described by sampling without replacement with a limited number of extractions (i.e. the sequencing depth).

"observed", i.e. "observed absolute abundance" is the number of counts measured by the sequencing process and "observed relative abundances" are the corresponding proportions relative to the total counts.

Paragraph added in Material and Methods

Table 1 summarises the methods considered in this benchmark study, providing information about software availability, underlying approach, preprocessing performed by the tool and adopted definition of DA taxa, i.e. if the methods aim at investigating significant differences in mean true absolute or relative abundance of each taxon. DESeq2, edgeR, MaAsLin2 and metagenomeSeq model the observed absolute abundance of each taxon using different statistical models with the goal of estimating the FC, that, if data are opportunely normalised, should reflect the FC between the true absolute abundances. ANCOM and ANCOM-BC are explicitly designed to target the true absolute abundances. All the other methods presented in Table 1 are classified as tools targeting the differentially relative abundance. A more detailed description of the methods is given in S1 File, Section 2.

While the authors do a great job at discussing the performance of tools when looking at differentially abundant features that are in the medium or high abundance range it is unclear how often this represents true scenarios in microbiome datasets. As such I think the authors might want to consider including some of the supplemental figures of their later examination of low abundance DA features in the main text.

As the rewiever proposed, we included a supplementary figure about low abundance DA features in the main text, i.e. Fig 9. This figure shows the recall values of each differential abundance method for each dataset considered in the comparison in the scenario with simulated DA features at low abundance. We decide to include this figure since the presence of low abundant DA features highly impact recalls.

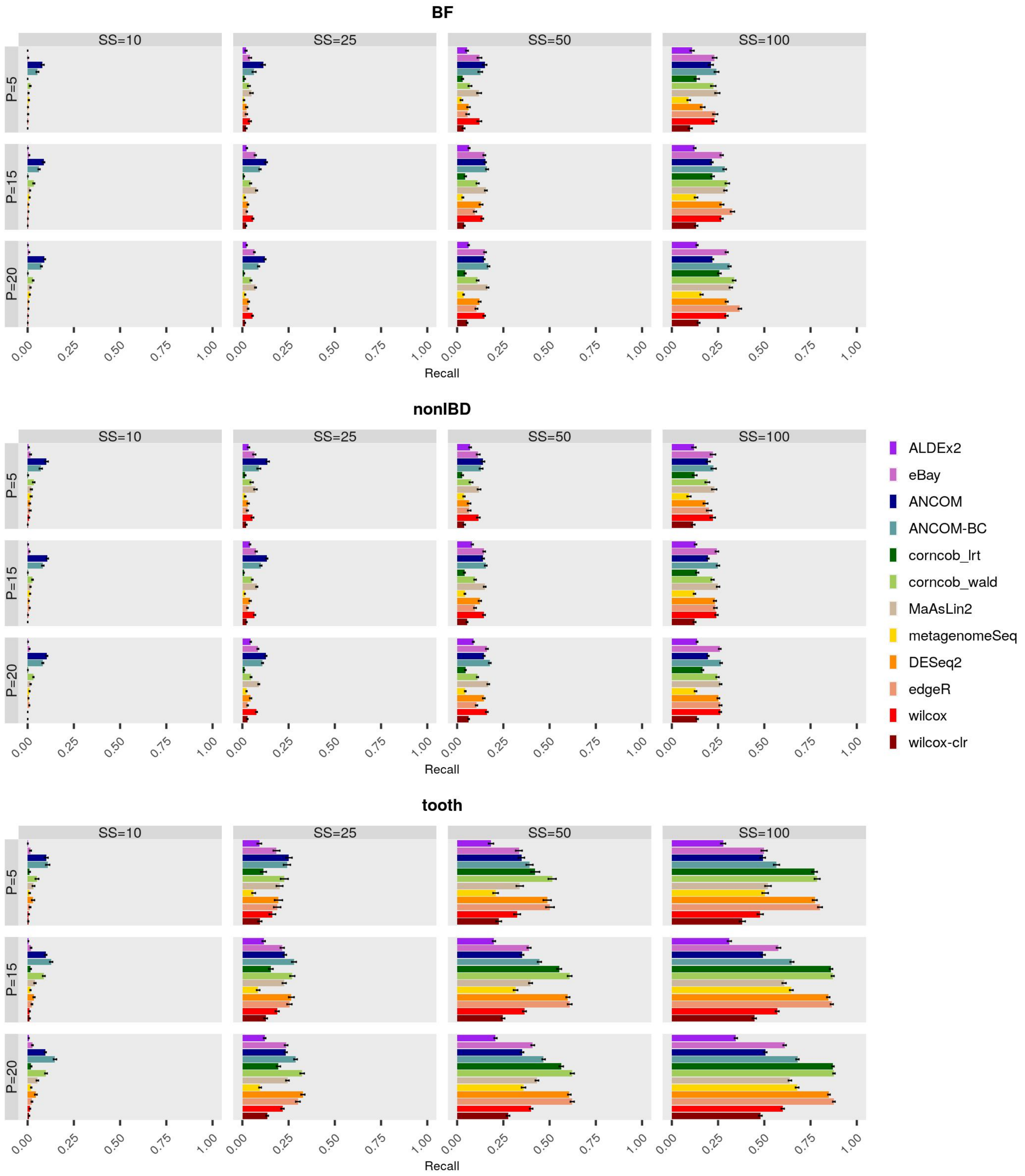

Fig 9. Recall of each differential abundance method for each dataset considered in the comparison in the scenario with simulated DA features and θ=0. In each set of boxes corresponding to the dataset, different percentages (P) of simulated DA features are on rows, while different sample size (SS) values are on columns. The recall values are averaged over the 50 simulations and the bars show the standard error.

The simulated datasets that the authors use to benchmark the tools in their report derive from human associated environments. It is unclear whether tool performance would differ if the simulation parameters were derived from environmental samples such as sediment or ocean water. Including some data on whether they would expect this to impact their simulations, or the benchmarks found in this paper would help readers who are interested in characterizing environments that are not human associated.

Thank you for your suggestion. We have added two new datasets describing chicken gut microbiota ("AnimalGut" dataset [11]) and soil communities ("Soil" dataset [12]). The description of the two datasets was added in S1 file, Section 4 and Table C.

The main findings are shown in a new section in Results (reported below for reviewer convenience) and in Figures 94-105 in S1 file.

Subsection added in Paragraph Results - Effect of non-human associated niches

Taking into account different ecological niches, different absolute performances are observed in terms of FPR, FDR and recall, although general trends remain valid (see Figures 94-105 in S1 file). In general, the tools are confirmed to be capable of maintaining FPR below 0.05 with some exceptions. For example, the two corncob variants show FPR slightly higher than 0.05 for SS = 100 and P = 15, 20. Similarly, edgeR and Wilcox have an high number of FP in the Soil dataset at P=20. MetagenomeSeq, on the other hand, still shows good performance in term of FPR although the recall is the lowest compared to all the other methods.
As for the human associated datasets, for certain methods FDR is more difficult to control than FPR. This is particularly evident, in the case of non-human associated environment, for SS=50 and SS = 100 where some methods reach an FDR up to 25%. However, the methods that are able to correctly control the FDR are the same for human and non-human datasets.

Differently form the human associated datasets, most of the methods are able to call at least one DA feature even at low sample sizes, except for metagenomeSeq in the AnimalGut dataset. Consistently, recall values increase with respect to human associated datasets, and the majority of methods show satisfactory values (around 75%) already at SS=25.

Other comments:

In the supplemental figures indicating computational time it is unclear whether each tool was run using a single core or multiple cores as many tools allow for the use of both. If possible, it would be best if the authors reported true cpu time (the time the cpu spent working on this task) rather than the change in system time between the start and end of their computation. This could be important if other software or OS updates were running during there testing time.

Following your suggestion, we checked the execution time of all methods. We found results in line with the previously submitted version of the ms.

The supplemental information for this manuscript contains 73 figures and while they do a great job at presenting all their data, they might want to consider reorganizing the contents to highlight the more impactful figures such as those directly mentioned within the text. Like many benchmarking studies the authors conclude that DA tools perform similarly to one another with no clear choice in which tool is best to use. Considering this information, it would be nice if the authors gave some recommendations on how to properly conduct DA analysis in their own datasets.

We agree that looking at all the figures in the ms could be hard. Therefore, we add a new sub-section in "Results - Summary of overall performance", where we summarised all the performance in the main scenario across the 5 datasets considered in our study. In this way we provide suggestion about which methods to use or focus on. This sub-section describes how we attempt to summarise the results. In the following we report for your convenience the new sub-section added, and also the overall performance figure shown in the main text, i.e. Fig 12. Additional figures that summarize the results are available also in the Supplementary materials (Figures 106-107 in S1 file).

Paragraph added in Results – Summary of overall performance

While the ecological niche is an information that is trivially available in any 16S rDNA-seq study, and a characteristic that affects DA methods performances in our study, feature variability, percentage of DA taxa, and amount of low abundant DA taxa are elements on which researches have typically no control or for which there is few a-priori information.
On the other hand, number of samples, sequencing depth and count normalisation are elements that can be controlled during a 16S rDNA-seq study, despite only the number of samples seems to have a considerable impact on DA analysis in our tests.

Therefore, based on the elements that seem to affect most the accuracy of a DA analysis, i.e. the ecological niche and the number of samples, we try to summarise methods results and provide some recommendations on which DA method may better fit a particular scenario. We decide to compare the values of the metrics in the 5 datasets for the scenario in which the simulated DAs are in the medium-high range, i.e. setting the θ threshold. Basically, performing this comparison by simulating DA mainly in the low abundance range would not be significant since we have already seen that the method performance drops dramatically.
We consider the means of FDR, Recall, percentage of datasets for which at least one taxon has been identified as DA (NA_perc) and pAUPR calculated on all simulations by combining the percentages of DA taxa (P), since it is an unknown covariate and, in most scenarios, it does not particularly affect the metrics. We do not consider computational times since in our settings they are negligible even running the tools using a single core (although some support parallel computing, see S1 File, Section 6).
Figure 106-107 in S1 File show all the metrics mean values, reporting with a colour scale the mean normalised ranking for each method. Although the absolute means change as the dataset changes, there are two different groups of datasets. BF, nonIBD and Soil have similar patterns; namely ALDEx2, ANCOM-BC, DESeq2, eBay are in the top-down ranking considering all the metrics. On the other hand, tooth and AnimalGut show a similar top-down ranking pattern for corncob_wald, corncob_lrt, DESeq2 and edgeR. In summary, the ecological niche does not seem to have an effect on the overall ranking of the methods, since similar patterns are observed between human and non-human associated datasets, while it affects the absolute performance.
However, the ranking shown in Figure 106-107 in S1 File does not take into account the average value of the metric. For example if all methods fail to control the FDR, the ranking still finds a winner among the methods. Consequently, we assign a score between 1 and 5 (1 = "low", 2 = "low-mid", 3 = "mid", 4 = "high-mid" and 5 = "high") to each mean value of the metrics by defining some thresholds on metrics values. In brief, methods having a high precision and controlling the nominal FDR of 0.05 (i.e. precision >= 0.95) were assigned to class "high". Method achieving a precision >= 0.90 were assigned to class "high-mid", representing tools that still provide a very high precision but slightly exceeding the chosen FDR threshold. Overall, methods in the above two classes represent robust alternatives when controlling type I error is a relevant constraint for the study. The remaining classes describes methods having a precision < 0.9, with the

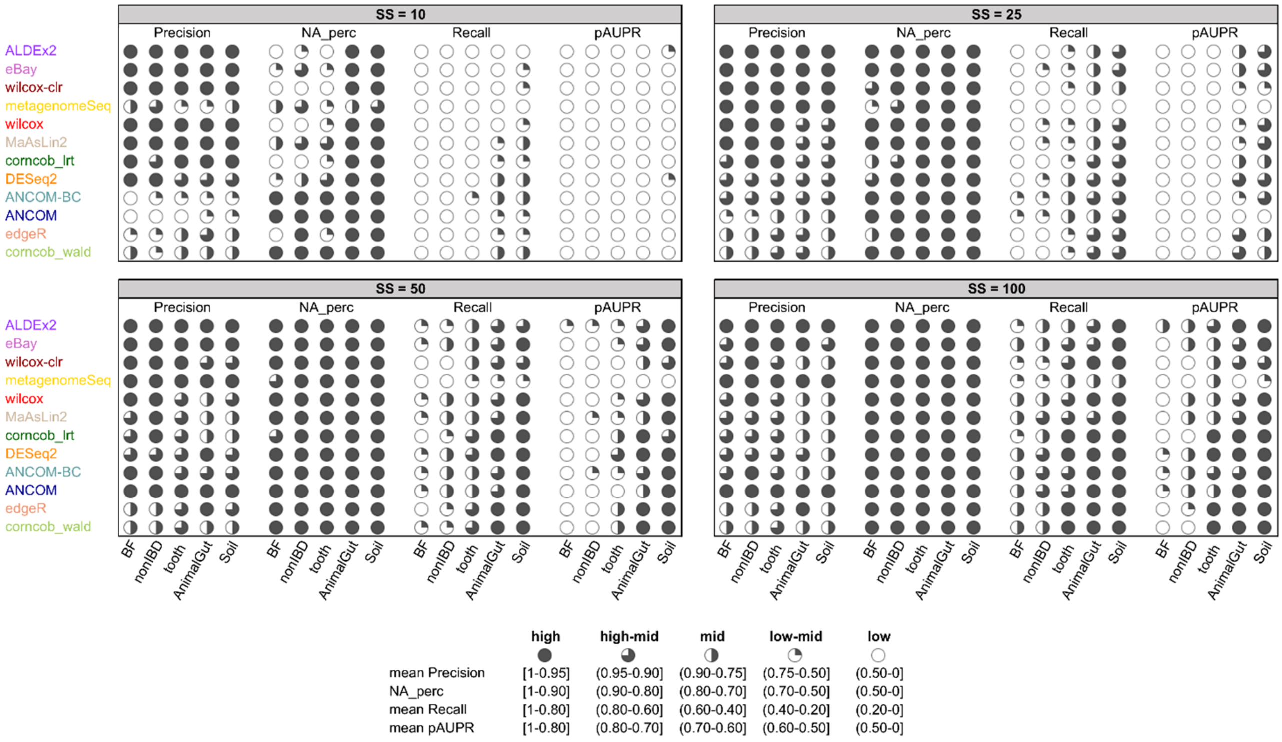

Fig 12. Overall performance of each DA method. In each set of boxes corresponding to different sample size (SS) values, Precision, NA_perc (percentage of available precision), Recall and pAUPR scores are shown for each dataset in columns. Methods (on rows) are ranked based on Precision values across all the SS scenarios and then based on Recall in case of ties. The legend below the boxes explains the threshold used to assign the overall score for each metric.

class "low" containing the methods achieving a maximum precision of 0.5. Closely related to precision, we also classified the methods in terms of "NA_perc", i.e. percentage of datasets for which at least one taxon has been identified as DA. Methods able to identify at least one DA taxon in the great majority of datasets (i.e. > 75%) were assigned to the highest classes. In particular, methods able to identify at least one DA taxon in 100% or more than 90%, 75% of datasets were assigned to classes "high", "high-mid" and "mid", respectively. Performance in terms of recall were ranked in 5 classes representing the 5 quantiles in the recall range [0-1]. Since the recall is the metrics showing the largest difference across different number of samples SS, covering almost the entire range [0-1], a stratification in 5 quantiles provide a fair and straightforward way to summarise method performance. In terms of pAURP, the lowest class represents methods having an pAURP < 0.5, while a pAUPR > 0.7 could be a reasonable labeled as "high", "high-mid" and "mid" with 0.1 step.
Fig. 12 shows the methods ranked as described above, sorting them first by precision and then by recall, across all SS scenarios in each dataset. In SS = 10, MaAsLin2 always control the precision and reaches higher recall values in 2 out of 5 datasets. As the sample size increases, a pattern seems clearer. ALDEx2 is among the best in terms of FDR control on all datasets for SS = 25, 50, 100; similarly eBay shows good results for SS = 25-50; while ANCOM reaches top performance for SS = 50-100. The recall values of these methods are in the top ranking, although there are methods that achieve higher recall at the expense of not controlling the FDR in the majority of the datasets.

Please indicate in the main text the reasoning behind why the mean values in condition B for a taxon were limited to the range of mean values in condition A. This is surely not something seen in all microbiome datasets and could impact the performance of various DA tools.

As now reported in the main ms (section Simulating 16S count data with differentially abundant taxa – Simulation of DA taxa) *"It is worth noting that setting a range on the mean absolute abundances for condition B has the purpose of generating data in a plausible range. However, this does not imply that the two conditions have exactly the same maximum and minimum intensity in the resulting count table. In fact, μ is provided as input to the gamma distribution, and, after the application of the biological (Equation 1) and technical (Equation 2) noise, it is reasonable to expect that the two conditions have different average maximum observed abundances, although roughly in the range of values observed in the real experimental condition."*

It was interesting to see the high FPR of metagenomeSeq and the low FPR of edgeR despite a recent publication finding the opposite (https://doi.org/10.1038/s41467-022-28034-z). It would be great if the authors discussed the opposite results as many of the other conclusions from these papers agree with one another.

We thank the reviewer to point us to this article. Since we change the model in metagenomeSeq (see the answer to reviewer 3 and S1 file section 9) now our findings are in line with results in Nearing et al. [13]. In addition, we found that ALDEx2 and ANCOM are the best looking at the overall performance, as also see in Nearing et al [13] in term of consistency results across studies. We revised the discussion in order to highlight the difference between the previous comparison. In the following we report only the paragraph in discussion where we cited the suggested study by Nearing et al [13].

Paragraph added in Discussion

Similarly, in Nearing et al. [52] ALDEx2 and ANCOM demonstrated not only consistent results between datasets, but also the best agreement with different approaches. Furthermore, given the variability of the performance on the datasets, the authors recommend a consensus approach. Our findings can help identify the best tools to focus on.

The authors examine the performance of tools due to varying sequencing depths, however, it would be interesting to see tool performance when mean sequencing depths between conditions A and B differ from one another as this issue can often occur during microbiome analysis.

As suggested, we added a scenario when mean sequencing depths between conditions A and B differ from one another. The results are shown in Figures 53-64 in S1 File and discussed in a new paragraph in *Results - Effect of sequencing depth.*

Paragraph added in Assessment scenarios

Furthermore, in microbiome analysis the mean sequencing depth between condition A e B could differ. Therefore, we test tool performances also simulating nonIBD scenarios by doubling L only for condition B, thus creating an unbalanced sequencing depth scenario.

Paragraph added in Paragraph Results - Effect of sequencing depth

In the unbalanced sequencing depth scenario, trends similar to the balanced scenario are still observed (see Figures 53-64 in S1 File). However, the FDR appears to be affected by the change in the percentage of DA at high sample size, i.e. SS = 50 and SS = 100. In particular, ALDEx2, ANCOM, eBay and ANCOM-BC fail to control the FDR for P = 20 and SS = 50-100 with eBay showing an increase in FDR also at SS=25 for all P. metagenomeSeq and MaAsLin2, on the other hand, is the tool with the best control of the FDR, which is also not affected by the percentage P (although recall remains the lowest). Looking at the AUPR values, no changes are observed with respect to the balanced scenario. However, the pAUPR values are lower for ALDEx2, eBay, ANCOM but only for SS = 100 and P = 5, while in the other scenarios the ranking of the methods is maintained and the values of pAUPR are also comparable.

While it is not need it would strengthen the manuscript to see other popular DA microbiome tools included in their analysis such as MaAsLin2 or LEfSe.

As the reviewer suggested, we included in our benchmarking MaAsLin2, since it's a popular DA microbiome tool. We run this method for each scenario and also comment the performance through the ms. We add a brief descript of the method in Table 1 and S1 file, Section 2, we update "Results" and "Discussion"sections, and update all the figures (Figures 3-12 in the main text, and figures 4-5, 18-107 in S1 file).
We decided to exclude LEfSe from the comparison since it's a discriminant analysis method as pointed out by Lin et al. [10] *"By its construction, LEfSe method is more a discriminant analysis method rather than a DA method. Unlike the DA analysis methods discussed earlier in this paper, LEfSe is more focused on investigating the relationship among microbial profiles and an outcome or phenotype (Step 3). More precisely, LEfSe tries to quantify the magnitude of the effect size of such associations between microbial profiles (e.g. a set of taxa) and the outcome of interest.".*

I was unable to locate a legend for figure 1.

Thanks for your advice. We checked that the legends of each figures are reported at the end of the section where cited for the first time.

Reviewer #3:

Cappellato et al. benchmarked several differential abundance methods in microbiome data using simulated data generated by the metaSPARSim. While there had been several comparative studies that assessed differential abundance approaches, the authors benchmarked with more metrics, and considered more scenarios and covariates than other studies.

The count generative model used a gamma distribution to model the absolute abundance level of a taxon in samples with the dispersion parameter representing the biological variability and the mean abundance parameter. In addition, the technical variability is modeled by multivariate hypergeometric distribution with a sequencing depth parameter. Parameters were estimated from the reference conditions of three different real datasets, and parameters for the other conditions were then simulated based on the fold changes of mean abundance with further settings of desired percentage of differential abundant features and linear interpolation of the dispersion. The scripts and simulated data were made available online.

Side comments:
It is good to see the use of Precision & Recall metrics used for few differential abundant taxa.
Please note that this is benchmarking results from one method of simulating the data.

Major

-While the authors pointed out other reports on the under performance of metagenomeSeq, have the authors examined if has anything to do with the normalization approach? Please also report the normFactor used. Also was the Zero-inflated Log-Normal mixture model tested as well? From their manual, “We currently recommend using the zero-inflated log-normal model as implemented in fitFeatureModel.” The Precision-recall curves of metagenomeSeq are way too striking, especially for large SS. It appears way worse than random, which is bothersome. Would the authors be able to double-check and investigate further and offer comments on why it performs so poorly in the discussion?

As the reviewers rightly noted, the performance of metagenomeSeq strongly depends on the model used. Despite our simulations validate what previously found regarding metagenomeSeq [10,14,15], we agree that a more in-depth analysis needs to be carried out. Therefore, we compare the results obtained using the zero-inflated Log-Gaussian (ZILG) mixture model and the zero-inflated Gaussian (ZIG) model. We added a new section in the S1 File that summarise the results of this comparison. For the sake of simplicity, we show here Figures 110 and 112 from S1 File, Section 9, regarding the performance comparison in term of FDR and Recall. We found that ZILG model shows good control of the FDR at the desired threshold, while the performance in terms of recall decreases with respect to those observed using ZIG. This behaviour was also observed in Lin et al. [10] which is the only one of the previously cited studies that compares both models.
We decided to consider the ZILG model in the new version of the manuscript. Now the "metagenomeSeq" label in all the figures refers to the implementation with the “fitFeatureModel” function (i.e. ZILG model) run as recommend by the authors.
Since we believe it is important to emphasize the different behaviour of these two model in metagenomeSeq, we added also a paragraph in Discussion, thus make the possible users aware of the difference in terms of performance.

Section 9 added in S1 File, Comparison between ZIG and ZILG model in metagenomeSeq

In previously founding [24–26], the performance of metagenomeSeq is particularly unsatisfactory: i.e. high recall but also high FDR values. Since our simulations state the opposite, we carry out a more in-depth analysis. The authors of metagenomeSeq modified their originally procedure that exploited the zero-inflated Gaussian (ZIG) model and recommended using zero-inflated Log-Gaussian (ZILG) mixture model for DA analysis. Therefore, we run metagenomeSeq with both ZIG and ZILG model following the instruction in the Bioconductor vignette. We note that by default in the R package metagenomeSeq scales the normalisation factors (nf) as follows:

$$log_2\left(\frac{nf}{1000}+1\right) \tag{6}$$

Instead, in the vignette the authors scale the normalisation factors in a different way: $log_2\left(\frac{nf}{median(nf)}+1\right)$

We follow the example in the vignette, in analogy to what was done in Calgaro et al. [25]. Furthermore, the difference between the two models lies not only in the logarithmic scale used, but also in the normalisation factors scaling as shown in the following table.

Table D. *Summary of difference between ZILG and ZIG implementation in metagenomeSeq R package.*

Model	*R function*	*Scale factor*
ZILG	*fitFeatureModel*	*log2(nf/median(nf)+1)*
ZIG	*fitzig*	*log(nf/median(nf))*

We evaluate the differences in terms of FPR, FDR and Recall between the two models on the simulations in the scenario setting the θ threshold (see Figures 108-113).
We find that ZILG model shows good control of the FDR at the desired threshold, while the performance in terms of power decreases. This behaviour was also found in Lin et al. [26] which is the only one of the previously cited studies that compares both models.

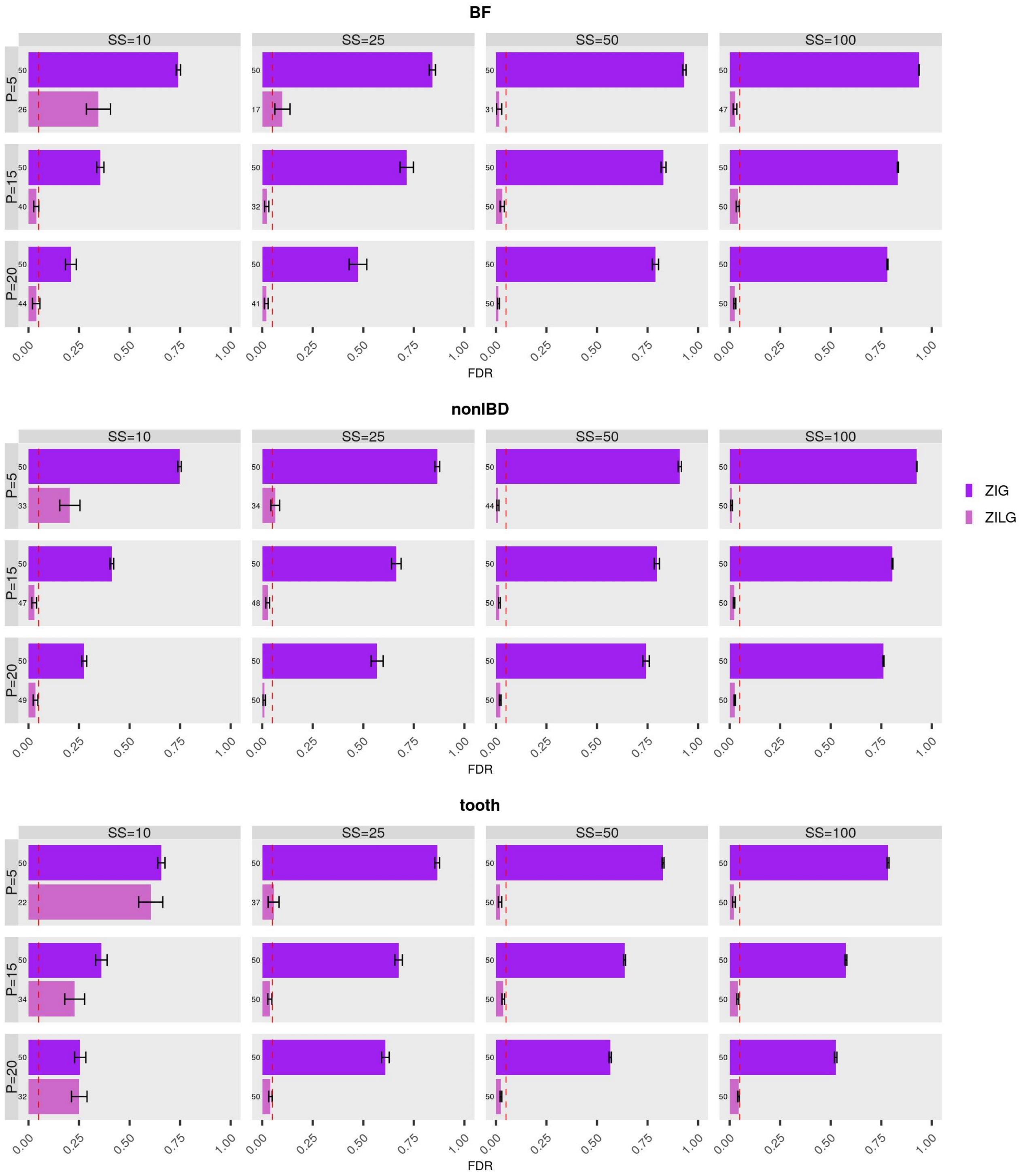

Figure 110. False Discovery Rate (FDR) of ZIG and ZILG model in metagenomeSeq for BF, nonIBD and tooth dataset in the main scenario with simulated DA features. In each set of boxes corresponding to the dataset, different percentages (P) of simulated DA features are on rows, while different sample size (SS) values are on columns. The FDR values are averaged over the 50 simulations and the bars show the standard error. The number of runs that provide a defined value of FDR is shown at the beginning of the bars.

Paragraph added in Discussion

It is worth noting that our results regarding metagenomeSeq do not match previous comparisons. Specifically, other works [13,17,19] found that metagenomeSeq is characterized by high recall but also high FDR values, while we observe opposite behaviour. In fact, metagenomeSeq implements two different models: the zero-inflated Log-Gaussian (ZILG) mixture model and the zero-inflated Gaussian (ZIG) model. We recommend following the authors' suggestions using the ZILG model since it shows extreme performance improvement with respect to the ZIG model (a comparison between the two approaches is shown in S1 file, section 9).

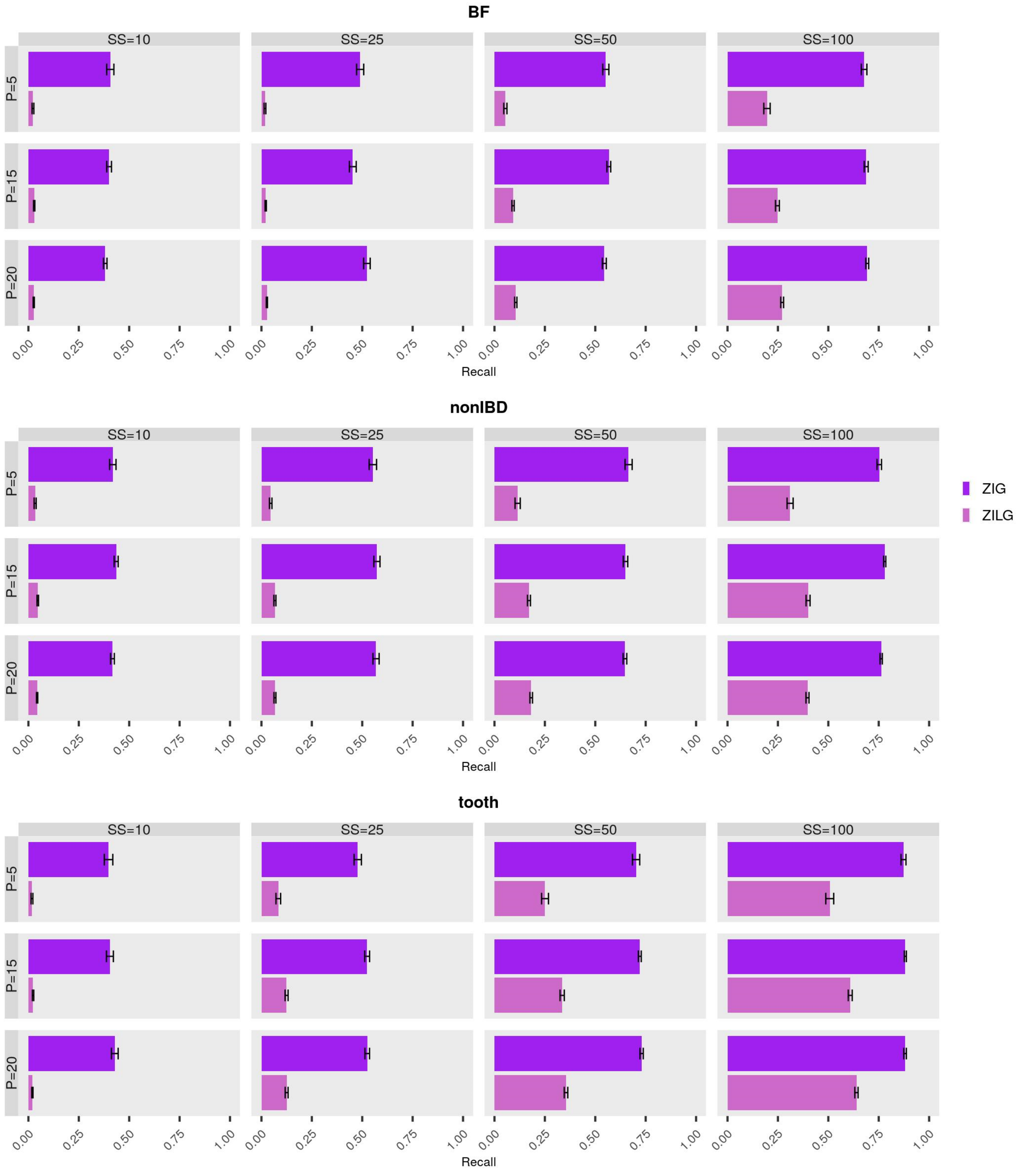

Figure 112. Recall of ZIG and ZILG model in metagenomeSeq for BF, nonIBD and tooth dataset in the main scenario with simulated DA features. In each set of boxes corresponding to the dataset, different percentages (P) of simulated DA features are on rows, while different sample size (SS) values are on columns. The recall values are averaged over the 50 simulations and the bars show the standard error.

Minor

- Sub-headers could make the "Simulating 16S count data with differentially abundant taxa" section in Methods easier to read.

Thank you for this suggestion, we also think that adding sub-sections could improve the readability of the *Simulating 16S count data with differentially abundant taxa* section. Therefore, we divided the section following this structure:

- 16S count data modelling
- Simulation of DA taxa
- DA taxa: differences between absolute and relative abundances

- I find the figures a challenge to follow at times. Eg. It is very challenging to compare Figure 7 to Figure 3 to see how they differ. It may be a better representation simply with a scatter plot of the values between (a subset of) the two figures.

We added a new version of Fig 7 (now it is Fig 8). In particular, at the beginning of each bar we reported the difference in the mean FDR between the scenario with reduced variability shown in Fig 3 (now it is Fig 4) and the main scenario with simulated DA features. Adding this information could help the readers to follow the comparison between these scenarios.

References

1. Mandal S, Van Treuren W, White RA, Eggesbø M, Knight R, Peddada SD. Analysis of composition of microbiomes: a novel method for studying microbial composition. Microb Ecol Heal Dis. 2015 May;26:27663.
2. Lin H, Peddada S Das. Analysis of compositions of microbiomes with bias correction. Nat Commun. 2020;11(1):3514.
3. Robinson MD, McCarthy DJ, Smyth GK. edgeR: a Bioconductor package for differential expression analysis of digital gene expression data. Bioinformatics. 2010 Jan;26(1):139–40.
4. Love MI, Huber W, Anders S. Moderated estimation of fold change and dispersion for RNA-seq data with DESeq2. Genome Biol. 2014 Dec;15(12):550.
5. Mallick H, Rahnavard A, McIver LJ, Ma S, Zhang Y, Nguyen LH, et al. Multivariable association discovery in population-scale meta-omics studies. PLOS Comput Biol. 2021 Nov 1;17(11):e1009442.
6. Paulson JN, Stine OC, Bravo HC, Pop M. Robust methods for differential abundance analysis in marker gene surveys. Nat Methods. 2013 Dec;10(12):1200.
7. Fernandes AD, Reid JNS, Macklaim JM, McMurrough TA, Edgell DR, Gloor GB. Unifying the analysis of high-throughput sequencing datasets: characterizing RNA-seq, 16S rRNA gene sequencing and selective growth experiments by compositional data analysis. Microbiome. 2014 May;2(1):15.
8. Martin BD, Witten D, Willis AD. MODELING MICROBIAL ABUNDANCES AND DYSBIOSIS WITH BETA-BINOMIAL REGRESSION. Ann Appl Stat. 2020 Mar;14(1):94–115.
9. Liu T, Zhao H, Wang T. An empirical Bayes approach to normalization and differential abundance testing for microbiome data. BMC Bioinformatics. 2020 Jun;21(1):255.
10. Lin H, Peddada S Das. Analysis of microbial compositions: a review of normalization and differential abundance analysis. NPJ Biofilms Microbiomes. 2020 Dec;6(1):60.
11. Patuzzi I, Baruzzo G, Losasso C, Ricci A, Di Camillo B. metaSPARSim: a 16S rRNA gene sequencing count data simulator. BMC Bioinformatics. 2019 Nov;20(Suppl 9):416.
12. Yurgel SN, Douglas GM, Dusault A, Percival D, Langille MGI. Dissecting community structure in wild blueberry root and soil microbiome. Front Microbiol. 2018 Jun 6;9(JUN):1187.
13. Nearing JT, Douglas GM, Hayes MG, MacDonald J, Desai DK, Allward N, et al. Microbiome differential abundance methods produce different results across 38 datasets. Nat Commun. 2022 Dec 1;13(1).
14. Hawinkel S, Mattiello F, Bijnens L, Thas O. A broken promise: microbiome differential abundance

methods do not control the false discovery rate. Brief Bioinform. 2019 Jan;20(1):210–21.

15. Calgaro M, Romualdi C, Waldron L, Risso D, Vitulo N. Assessment of statistical methods from single cell, bulk RNA-seq, and metagenomics applied to microbiome data. Genome Biol. 2020 Aug;21(1):191.
