# Supplementary material for: Investigating differential abundance methods in microbiome data: A benchmark study
Source: PLoS Comput Biol. 2022 Sep 8;18(9):e1010467. doi: 10.1371/journal.pcbi.1010467 (PMC9488820; doi:10.1371/journal.pcbi.1010467)
Supplement: Attachment — Submitted filename: Cappellato_Baruzzo_DiCamillo_ReviewAnswer_2.pdf [file pcbi.1010467.s004.pdf]

Dear Editor,

We tackled all the matters raised by Reviewer #2 and reported all the changes in the main paper and supplementary material highlighting the text in red.

In details we:

- Add information on taxon bias not considered by the tools and update Table 1.
- Clarify through the manuscript the term “relative” specifying with respect to what.
- Update the classification for MaAsLin2 as relative abundance-based method when TSS normalisation is involved.
- Add experimental covariates investigation as a perspective in Discussion.

We think the Reviewer's suggestions have served to improve the clarity of some key points of our work. We revised the manuscript to eliminate possible misunderstandings and hope it is now publishable on Plos Computational Biology.

The details of our response are attached below.

Best regards,

Barbara Di Camillo

Reviewer #2:

I believe that the authors should at least address or clarify within table 1 that these methods still do not account for specific taxon biases (highlighted in Clausen et al., 2022, and McClearn et al., 2019). While I do not think the manuscript needs to go in depth on this point, I believe that is it important to note these biases when estimating absolute abundances and discussing what these tools are trying to measure within the samples they are provided.

We thank the Reviewer for the suggestion. We clearly stated that only few methods implement some strategies to consider taxon-specific biases, and that none of these strategies are included in the default setting (so not included in our comparison). We also updated the description of the DA methods in the supplementary materials (S1 File, Section 2) to include such information.

We added these sentences in paragraph “Material and Methods – Assessed differential abundance methods” and we updated Table 1 accordingly.

16S and metagenomics data are affected by experimental bias throughout the entire experimental workflow given that DNA extraction, PCR, and sequencing steps preferentially measure and amplify certain taxa over others, therefore distorting the measurements with taxon- and protocol-specific biases [33,34]. Usually, this aspect is only partially addressed by normalisation methods that aim primarily at making comparisons across samples within the same taxon and experiment. However, the comparison across different taxa or using data from different protocols is not possible without addressing this issue. Although this aspect goes beyond the scope of our work, we report in Table 1 a column specifying if the different tools could address taxon bias (see S1 File, Section 2 for more details).

While I think the authors have a point that ALDEx2 and eBays may not be measuring absolute abundance directly it is also incorrect to say they are measuring relative abundances at least in the

traditional sense (measured as proportions of the whole). The center-log-transformation that they apply is not only to project data into Euclidean space as indicated by the authors but is also there to provide a reference for how that taxon increases or decreases in comparison to the geometric mean abundance. While this could be thought of as a type of relative abundance (as you are comparing the abundance of a taxon relative to something else) it is not similar to what is generally referenced as relative abundances in microbiome data (i.e., measuring taxa as proportions of the whole). This later approach is commonly used in conjunction with Wilcoxon or t-tests for differentially abundance screening. I believe this difference should be highlighted within the table and made clear to the reader because it can have major differences in how the results of these tools should be interpreted. I.e., knowing that a taxon is increased across samples within a group compared to the geometric mean abundance of all taxa within that group is different then knowing that the proportion of that taxon is increased across the samples within that group. Overall, I think this really highlights that a clearer definition of what relative abundances mean in the context of this table should be discussed.

We see the Reviewer point and agree that when we state "relative" we might specify with respect to what.

We corrected Table 1 following the Reviewer suggestion and updated both the main text and the S1 File, Section 2 accordingly.

Table 1. Summary of the methods included in this benchmark study in alphabetical order.

**Method**	**Software**	**Approach**	**Type of filtered features**	**Absolute/relative differential abundance**	**Support to taxon bias correction**
ALDEx2	R package[1]	Bayesian estimation of true relative abundance by Monte Carlo sampling. Centered log ratio (clr) transformed data are tested and mean p-values and q-values are provided after statistical test.	All zero features in both groups.	Relative abundances with respect to the geometric mean	No
ANCOM-II	R script[2]	Test the difference between true absolute abundances using the log ratios of the observed absolute abundances (i.e. the count matrix). Each log ratio between the features of the subjects belonging to the same experimental group is described with an ANOVA model. The model's coefficients serve to perform the statistical test for each log-ratios.	• Features that have a percentage of zeros across all samples/subjects greater than a predefined *zero_cut* threshold.<br>• Structural zeros are considered as differentially abundant and removed from the dataset.	Absolute abundances	No
ANCOM-BC	R package[3]	Estimate the unobservable sampling fraction c_j, correcting the bias introduced by the possible extreme variation between the subjects. A linear model with a sample-specific offset term estimated	• Features that have a percentage of zeros across all samples/subjects greater than a predefined *zero_cut* threshold.<br>• Structural zeros are considered as	Absolute abundances	No

		from the observed absolute abundances (i.e. the count matrix) is used to describe the log true absolute abundance of feature *i* in subject *j*.	differentially abundant and removed from the dataset.		
Corncob	R package[4]	It estimates expected true relative abundances from the observed absolute abundances (i.e. the count matrix) fitting a beta-binomial model. The parameters are estimated through maximum likelihood and two different testing procedures are implemented: Likelihood-ratio (LRT) or Wald tests.	• All zero features in both groups.<br>• Features for which the method fails to estimate the model parameters.	Relative abundance	No
DESeq2	R package[5]	Negative binomial distribution is exploited to model observed absolute abundances. Relative log expression (RLE) is the default normalisation applied to observed absolute abundance (i.e. the count matrix). After model fitting, Wald test is used to evaluate taxa difference between groups.	Rare or outlier features identified using a procedure based on the Cook's distance.	Absolute abundances	Yes
eBay	R package[6]	Empirical Bayesian estimation of mean posterior distribution of true relative abundance. Centered log ratio (clr) transformed proportions are tested to obtain the p-values and q-values.	None	Relative abundances with respect to the geometric mean	No
edgeR	R package[7]	It is assumed that observed absolute abundances (i.e. the count matrix) follow a negative binomial distribution. Trimmed mean of M values (TMM) is the default normalisation applied to observed absolute abundance. After estimating model parameters, likelihood-ratio test (LRT) is used to test differentially abundant features.	None	Absolute abundances	Yes
MaAsLin2	R package[8]	Log-transformed linear model on Total Sum Scaling (TSS)-normalised observed abundances.	Features with minimum prevalence at 10%.	Relative abundances	No
metagenomeSeq	R package[9]	Observed absolute abundances (i.e. the count matrix) are modelled through zero-	None	Absolute abundances	No

		inflated Log-Normal mixture model using the built-in normalisation cumulative sum scaling (CSS). The estimated FC parameter is involved in the formulation of the statistical test.			

It is still unclear to me how MaAsLin2 is identifying absolute abundances when it models TSS normalized data. I do believe TSS normalization inherently turns the data into proportions or relative abundances. If the authors do believe that this tool is measuring absolute abundances a longer comment on their stance would be useful.

We thank the Reviewer for highlighting this point about TSS normalisation.

MaAsLin2 accepts as input different kind of data (count and non-count), and it supports different data normalisations and transformations. Therefore, it could be used to assess differences in relative or absolute abundances depending on the adopted combination of input data, normalisation and transformation. We better clarify this point in the description of the tool in the supplementary materials (S1 File, Section 2).

We agree with the Reviewer that MaAsLin2 is measuring relative abundances when input data are count and TSS normalisation is used. We updated the classification for MaAsLin2 as relative abundance-based method when TSS normalisation is involved.

In the manuscript it is concluded that performance of tools does not depend on ecological niche as performance of tools tended to cluster separately from non-human vs human. While I find this interesting tool performance does differ based on the underlying datasets in someway. Is it possible to compare some characteristics of these two clustering's to try and identify what is driving performance differences? However, I do understand that this analysis may not be possible due to the smaller number of datasets included within these groupings and may be out of scope of this manuscript.

As the Reviewer rightly observed performance of tools does not depend on ecological niche. Indeed, we observed two groups of datasets independently from non-human and human associated data. The fact that different datasets result in different tools' performance it's something that has been observed also in other benchmarking studies [**1-2**]. However, we found that relative ranking seems quite stable. We agree that it could be interesting go deep into the data characteristics that drive the performance difference. However, the number of datasets under consideration does not allow us to draw robust conclusion. Looking at the dataset metadata shown in the manuscript maybe the different grouping depends on the sequencing platform used since it's the only covariates that cluster data. In literature, there are some studies that confirm this behavior on bacterial composition and diversity analysis [**3-4**]. However, also the targeted region sequenced [**5-7**] and the bioinformatics pipeline used could influence the analysis results [**8-10**].

We decided to answer to this and the next review adding a paragraph in "Discussion" highlighting the experimental covariates inspection as future study (see answer to next review).

Finally, while the authors do a great job at addressing the limitations within their study. I believe they should include a sentence or two about the limitations of their simulations to be reflective of all 16S rRNA datasets. It is clear that even within this manuscript DA tools tend to perform better based on the underlying datasets that simulated data is parameterized on (as indicated by the two clusters they

found). Its highly possible that further differing results between DA tools could be obtained if more datasets were explored that came from various types of sample preparations, libraries, sequencing platforms, or bioinformatic processing.

As said in the previous answer, experimental covariates investigation is out of the scope of our work but could be an interesting suggestion for future work. We added the following sentences in "Discussion":

Finally, as pointed out by the comparison between different microbiota niches, although the relative ranking of different DA methods seems quite stable, results might depend on the type of dataset and on characteristics rising from different types of sample preparations, libraries, sequencing platforms, and read preprocessing. Although a more extended comparison addressing experimental covariates is out of the scope of this paper, an interesting development aspect for future studies might focus on analysing how DA methods cluster based on their performance and on the experimental characteristics of the analysed datasets.

References

[**1**] Nearing JT, Douglas GM, Hayes MG, MacDonald J, Desai DK, Allward N, Jones CMA, Wright RJ, Dhanani AS, Comeau AM, Langille MGI. Microbiome differential abundance methods produce different results across 38 datasets. Nat Commun. 2022 Jan 17;13(1):342.

[**2**] Calgaro M, Romualdi C, Waldron L, Risso D, Vitulo N. Assessment of statistical methods from single cell, bulk RNA-seq, and metagenomics applied to microbiome data. Genome Biol. 2020 Aug 3;21(1):191.

[**3**] Allali I, Arnold JW, Roach J, Cadenas MB, Butz N, Hassan HM, Koci M, Ballou A, Mendoza M, Ali R, Azcarate-Peril MA. A comparison of sequencing platforms and bioinformatics pipelines for compositional analysis of the gut microbiome. BMC Microbiol. 2017 Sep 13;17(1):194.

[**4**] Tremblay J, Singh K, Fern A, Kirton ES, He S, Woyke T, Lee J, Chen F, Dangl JL, Tringe SG. Primer and platform effects on 16S rRNA tag sequencing. Front Microbiol. 2015 Aug 4;6:771.

[**5**] Kerrigan Z, Kirkpatrick JB, D'Hondt S. Influence of 16S rRNA Hypervariable Region on Estimates of Bacterial Diversity and Community Composition in Seawater and Marine Sediment. Front Microbiol. 2019 Jul 16;10:1640.

[**6**] Soriano-Lerma A, Pérez-Carrasco V, Sánchez-Marañón M, Ortiz-González M, Sánchez-Martín V, Gijón J, Navarro-Mari JM, García-Salcedo JA, Soriano M. Influence of 16S rRNA target region on the outcome of microbiome studies in soil and saliva samples. Sci Rep. 2020 Aug 12;10(1):13637.

[**7**] Bukin YS, Galachyants YP, Morozov IV, Bukin SV, Zakharenko AS, Zemskaya TI. The effect of 16S rRNA region choice on bacterial community metabarcoding results. Sci Data. 2019 Feb 5;6:190007.

[**8**] Antich A, Palacin C, Wangensteen OS, Turon X. To denoise or to cluster, that is not the question: optimizing pipelines for COI metabarcoding and metaphylogeography. BMC Bioinformatics. 2021 Apr 5;22(1):177.

[**9**] Prodan A, Tremaroli V, Brolin H, Zwinderman AH, Nieuwdorp M, Levin E. Comparing bioinformatic pipelines for microbial 16S rRNA amplicon sequencing. PLoS One. 2020 Jan 16;15(1):e0227434.

[**10**] Chiarello M, McCauley M, Villéger S, Jackson CR. Ranking the biases: The choice of OTUs vs. ASVs in 16S rRNA amplicon data analysis has stronger effects on diversity measures than rarefaction and OTU identity threshold. PLoS One. 2022 Feb 24;17(2):e0264443.
